# Coleoptera genome and transcriptome sequences reveal numerous differences in neuropeptide signaling between species

**DOI:** 10.7717/peerj.7144

**Published:** 2019-06-17

**Authors:** Jan A. Veenstra

**Affiliations:** INCIA UMR 5287 CNRS, University of Bordeaux, Bordeaux, Pessac, France

**Keywords:** Neuropeptide, Genome, Transcriptome, Beetle, Neuropeptide F, Gene duplication, Gene loss, CCHamide

## Abstract

**Background:**

Insect neuropeptides are interesting for the potential their receptors hold as plausible targets for a novel generation of pesticides. Neuropeptide genes have been identified in a number of different species belonging to a variety of insects. Results suggest significant neuropeptide variation between different orders, but much less is known of neuropeptidome variability within an insect order. I therefore compared the neuropeptidomes of a number of Coleoptera.

**Methodology:**

Publicly available genome sequences, transcriptomes and the original sequence data in the form of short sequence read archives were analyzed for the presence or absence of genes coding neuropeptides as well as some neuropeptide receptors in seventeen beetle species.

**Results:**

Significant differences exist between the Coleoptera analyzed here, while many neuropeptides that were previously characterized from *Tribolium castaneum* appear very similar in all species, some are not and others are lacking in one or more species. On the other hand, leucokinin, which was presumed to be universally absent from Coleoptera, is still present in non-Polyphaga beetles.

**Conclusion:**

The variability in neuropeptidome composition between species from the same insect order may be as large as the one that exists between species from different orders.

## Introduction

Many neuropeptide signaling systems are commonly found in both protostomian and deuterostomian species, showing that most neuropeptides originated very early (Elphick, Mirabeau & Larhammar, 2018). Indeed, it is well established that genes coding neuropeptides and their receptors are well conserved during evolution, and this is not surprising as they are important regulators of a variety of physiological processes.

Neuropeptide evolution consists of different phenomena, on the one hand are gains of novel neuropeptides and losses of existing ones, on the other hand are structural changes of the neuropeptides themselves, which are usually minor, but sometimes significant. When one compares the neuropeptidomes of decapod crustaceans with those of insects, it becomes apparent that few new neuropeptides have evolved since the existence of their last common ancestor, but that in insects a relatively large number of neuropeptide genes has been lost (Veenstra, 2016a). It would be interesting to have a better understanding of neuropeptide loss in order to get a perspective on how it is possible that very ancient and well conserved regulatory systems can be lost in some species but remain apparently essential for others.

*Tribolium castaneum* was one of the first insect species for which a complete genome sequence was published (Richards et al., 2008). As the genes coding neuropeptides and their receptors were identified it became clear that at least three neuropeptide genes that seemed to be universally present in insects, those coding for corazonin, leucokinin, and allatostatin A, were absent from this species (Li et al., 2008). An observation that was confirmed by the absence of genes coding for the receptors of these neuropeptides (Hauser et al., 2008). The genes for two other well-known insect neuropeptides, pigment dispersing factor (PDF) and neuropeptide F (NPF) were neither found in this genome (Li et al., 2008), although receptors for such peptides were identified (Hauser et al., 2008). It thus appeared that the sequences of the latter two peptides might have evolved so much, that they can no longer be easily identified based on sequence homology using the BLAST program. This raises the question as to whether these peculiarities—the absence of three common insect neuropeptides and the apparent structural modification of two others—are characteristic of all Coleoptera and thus characteristic of this insect order, or whether they are more limited and specific for this particular species or family.

The public genome sequences for sixteen Coleoptera species (Richards et al., 2008; Keeling et al., 2013; Cunningham et al., 2015; Vega et al., 2015; McKenna et al., 2016; Meyer et al., 2016; Tarver et al., 2016; Ando et al., 2018; Fallon et al., 2018; Gautier et al., 2018; Kraaijeveld et al., 2018; Schoville et al., 2018; Van Belleghem et al., 2018a, 2018b; Wu, Li & Chen, 2018) should make it possible to identify their complete neuropeptidomes and answer this question. Given that *Tenebrio molitor* once was the most studied beetle, as still evidenced by the number of publications that can be retrieved for this species on PubMed, I have added it to the list, even though only transcriptome data are available for this species.

Neuropeptides act through receptors and these may also be lost or amplified. In Chelicerates several neuropeptide G-protein coupled receptors (GPCRs) are amplified multiple times (Veenstra, 2016c). Yet I have not systematically checked whether neuropeptide receptors might be duplicated or lost. In the absence of a neuropeptide gene duplication, receptor duplication is likely to fine-tune the effects of its ligand, but this is difficult to establish. The fruitfly is no doubt the best studied insect species and while it is known to have two different allatostatin C receptors, the physiological significance of having two instead of one, like most insect species, is unknown. Therefore, receptors were only studied when the ligand appeared to be absent and in those cases where a neuropeptide gene was duplicated.

## Materials and Methods

### Definition of neuropeptide

The definition of neuropeptide is sometimes ambiguous as in principle any peptide from the nervous system could be called a neuropeptide. In this manuscript neuropeptide is defined as a peptide or protein, that is, either released into the hemolymph, directly on a target tissue, or within the nervous system to regulate cellular activity by interaction with a specific cell surface receptor, usually a GPCR. A large number of such neuropeptides has been identified by biological activity on target tissues and/or by directly activating their receptors, while others been identified only by their homology to known neuropeptides. Some neuropeptides have been identified solely on the basis of being produced after proteolytic processing of proteins of unknown function or even only on the basis of the strong likelihood that their putative precursors could be processed by neuroendocrine convertases into neuropeptides. The latter are hypothetical neuropeptides only and are more properly called putative neuropeptides. Indeed, a recent analysis of the precursor of the salivary gland salivation stimulating peptide from *Locusta migratoria* suggests that it is not a neuropeptide after all (Veenstra, 2017). These putative neuropeptide precursors have been included here, even though no physiological effects have been described for these peptides and their receptors are unknown. On the other hand, I have not included the putative antidiuretic peptide identified from *Tenebrio* (Eigenheer et al., 2002). The definition given above does not exclude it from analysis, but it is almost certainly derived from a cuticle protein (CAA03880). Although there is a one amino acid difference between the C-terminus of the reported sequence of this cuticle protein (Mathelin et al., 1998) and the antidiuretic peptide that was sequenced, when constructing a *Tenebrio* transcript with Trinity using the various RNAseq sequence read archives (SRAs) from this species the C-terminus of this proteins was found to be completely identical to the antidiuretic peptide. There are no structure activity data with regard to its antidiuretic activity and it is unclear which protease is responsible for cleaving it from the rest of the protein. This makes it difficult if not impossible to reliably predict which other proteins might be the precursors of similar antidiuretic peptides.

### Sequence data

Genome assemblies were downloaded from NCBI (https://www.ncbi.nlm.nih.gov/genome) using the following direct links: https://www.ncbi.nlm.nih.gov/genome/?term=Tribolium; https://www.ncbi.nlm.nih.gov/genome/?term=Pogonus; https://www.ncbi.nlm.nih.gov/genome/?term=Nicrophorus; https://www.ncbi.nlm.nih.gov/genome/?term=Aleochara; https://www.ncbi.nlm.nih.gov/genome/?term=Oryctes; https://www.ncbi.nlm.nih.gov/genome/?term=Coccinella; https://www.ncbi.nlm.nih.gov/genome/?term=Harmonia; https://www.ncbi.nlm.nih.gov/genome/?term=Dendroctonus; https://www.ncbi.nlm.nih.gov/genome/?term=Hypothenemus; https://www.ncbi.nlm.nih.gov/genome/?term=Anoplophora; https://www.ncbi.nlm.nih.gov/genome/?term=Leptinotarsa; https://www.ncbi.nlm.nih.gov/genome/?term=Aethina), with the exception of the genomes of *Hycleus cichorii and Hycleus phaleratus*, which were obtained from the *GigaScience* repository (http://gigadb.org/dataset/100405) and those of *Photinus pyralis, Aquatica lateralis*, and *Ignelater luminosus*, which were downloaded from http://fireflybase.org/firefly_data.html. When available, protein sequences predicted from the various genomes were also downloaded from NCBI or the two websites mentioned using the same direct links indicated above. Predicted proteins for *Hypothenemus hampei* were obtained from https://genome.med.nyu.edu/coffee-beetle/cbb.html, and those for *Aleochara* the Animal Ecology department of the Free University of Amsterdam (http://parasitoids.labs.vu.nl/parasitoids/aleochara/data.php). For *Pogonus chalceus* the published transcriptome was useful (Van Belleghem et al., 2012). To facilitate reading, species will be identified by their genus name throughout this paper. In the case of the *Hycleus* species, this will refer to *Hycleus phaleratus*. There are also two *Harmonia* genomes, but these are from the same species and they showed no differences in the genes coding neuropeptides. Four other Coleopteran genomes are publicly available, however, they are not yet officially published and for this reason were not fully analyzed here. Those are *Sitophilus oryzae, Diabrotica viriginfera, Onthophagus taurus*, and *Agrilus planipennis*.

*Pogononus* belongs to the Adephaga, all the other species to the Polyphaga suborder. The genera *Coccinella, Harmonia, Hypothenemus, Dendroctonus, Anoplophora, Leptinotarsa, Aethina, Hycleus, Tenebrio*, and *Tribolium* all belong to the infraorder Cucujiformia. As will be seen this group shares certain neuropeptidome characteristics that are absent from the other Polyphaga as well as *Pogonus*.

The quality of these genomes is quite variable. Some have excellent assemblies and in addition numerous RNAseq SRAs making it possible to have high quality assemblies, others are much more limited. For example, the *Aleochara* assembly has no RNAseq data and only a limited amount of genomic sequences. In the case of *Aleochara* there is RNAseq data from a different species, *Aleochara curtula* (SRR921563, from the 1KITE project, Misof et al., 2014), which was helpful and it allowed in some case to reconstruct exons missing from the assembly using a combination of raw genome sequences and trinity. Nevertheless, it is still possible to ascertain the presence or absence of neuropeptide genes from this assembly.

In several instances the predicted complete coding sequences of some neuropeptides are incomplete. When there is little RNAseq data to deduce precursor sequences and a draft genome contains large and small gaps in the assembly such sequences are often incomplete and may well be incorrect in the parts that have been deduced. The *Oryctes* and *Aleochara* draft genomes suffer the most from these problems.

A complete list of all SRAs used is available in Table S1.

### Presence of neuropeptide and receptor genes

Predicted neuropeptide precursors were preferentially obtained from the annotated genomes, but this was not always possible. On the one hand, small neuropeptide genes are often overlooked by automated annotation programs, even though progress has been quite impressive in that respect, on the other hand there are quite a few transcripts that are probably wrong. Thus many neuropeptide precursors were corrected or predicted de novo from RNAseq data by using the tblastn_vdb command from the SRA Toolkit (https://www.ncbi.nlm.nih.gov/sra/docs/toolkitsoft/) on one or more SRAs using the *Tribolium* neuropeptide precursors as query to extract reads that could potentially encode a homologous protein. Those reads were then assembled using Trinity (Grabherr et al., 2011) and transcripts that might encode neuropeptides or other proteins of interest were then identified using BLAST+ (ftp://ftp.ncbi.nlm.nih.gov/blast/executables/blast+/). Trinity produced transcripts were judged complete when the N-terminal of the predicted neuropeptide precursors had a signal peptide that could be identified as such by SignalP (Petersen et al., 2011) as implemented on the Web (http://www.cbs.dtu.dk/services/SignalP/) and had an in-frame stop codon at the C-terminus. For GPCRs the identification of the N-terminus is often more ambiguous as some sequences did not have an in-frame stop codon upstream from the putative ATG start codon. In those cases, perceived similarities with homologous GPCRs were used as criterium for completeness. However, for the GPCRs analyzed here the aim was not to obtain absolutely perfect sequences for each receptor, but rather to show whether or not it is present in a particular species.

When in a first round of analysis with the tblastn_vdb command incomplete sequences were obtained, partial transcripts were then used as query for the blastn_vdb command to obtain, where possible, the remainder of the putative transcripts. This process sometimes needed to be repeated multiple times. Transcripts could also be completed by using the assembled genomes, and in several instances no transcripts were obtained at all and only the genome was available. Although many genes were located on single genomic contigs, this was not always the case. In those cases either Trinity produced transcripts and/or individual RNAseq reads, or homology with other precursors from other species were used to confirm the continuity of these transcripts.

It may be noted here that not all trinity produced transcripts are copies of mRNA species of the genes their sequences seem to indicate. In a previous paper on the RYamide gene in *Drosophila melanogaster* we showed that the very large majority of RNAseq reads that correspond to the coding sequence of this neuropeptide in the various SRAs are not due to the transcription of the RYamide gene, but rather parts of 3′-ends of mRNA produced from genes located upstream (Veenstra & Khammassi, 2017). The RYamide gene is very little expressed in *Drosophila melanogaster* and mostly in only two neurons in the adult. This causes the RYamide transcript to be so rare that virtually every RNAseq read that contains part of the coding sequence of this gene is in fact an artifact and is not derived from an authentic RYamide mRNA. It is likely that RNAseq reads from other genes that are neither extensively expressed, such as is the case for many neuropeptide genes, may similarly be the product from a heavily expressed upstream gene, rather than from the neuropeptide gene in question. This problem was, for example, encountered with the PTTH gene from *Hycleus* and the *Leptinotarsa* periviscerokinin gene. Such RNAseq reads attest to the existence of the neuropeptide gene in question, but have not necessarily undergone the same splicing as the one that is imposed on the neuropeptide mRNA. This phenomenon explains why certain Trinity produced transcripts predict mRNA sequences that contain introns that have not been excised. Whereas in some cases such “false” transcripts can be discarded easily due to the presence of an in-frame stop codon, in other instances such stop codons are absent. Even though obviously such data reveal alternatively splicing, it is not at all clear that this alternatively splicing actually occurs in mRNAs produced from the neuropeptide gene. In other words, what at a first impression may look like very sloppy intron processing, may in fact reflect sloppy stop codon processing in a gene upstream where such sloppiness has no consequences. It is for this reason that I have made no effort to carefully analyze all alternative splice forms for neuropeptide precursors and only recorded those that seem authentic and physiologically relevant.

The presence in RNAseq data of sequences that represent 3′-ends of primary mRNA sequences in which the polyadenylation signal has been ignored may lead to Trinity transcripts that are longer and extend into downstream genes. Thus many Trinity produced transcripts appear at first sight to lack a signal peptide, such transcripts were corrected by removing sequences judged to be extraneous based on sequence homology with other species and in the case of neuropeptide precursors on the presence of a credible signal peptide.

### Absence of neuropeptide and receptor genes

The methodology described above allows one to demonstrate the presence of particular neuropeptides. However, when a particular gene is not identified in this way, it does not necessarily mean its absence from the species in question. When a gene is absent from the transcriptome, it may be simply because its expression levels are very low, as, for example, in the case of the previously mentioned RYamide gene from *Drosophila melanogaster*. If the gene is also absent from the genome assembly, it is possible that it is located in a part of the genome that did not make it into the genome assembly.

Neuropeptides act via receptors, most of which are GPCRs. In many cases, but certainly not all, GPCRs are specific for a particular neuropeptide. So if a neuropeptide gene is genuinely missing from a species, one should expect its receptor to have lost its function and no longer be subject to positive selection. Hence, its receptor is expected to be lost as well. So, when both a neuropeptide and its unique receptor(s) are absent from a genome assembly, it is a good indication that the particular neuropeptide signaling system has been lost from the species in question.

For receptors that may be activated by neuropeptides derived from different genes, this argument can not be used. For example, a *Bombyx* myosuppressin receptor can be activated by both myosuppressin and FMRFamides (Yamanaka et al., 2005, 2006), hence if either the myosuppressin or FMRFamide were lost, this receptor could still be present. Similar situations likely exists for other neuropeptides, for example, the CCHamides 1 and 2 (Hansen et al., 2011; Ida et al., 2012) or short NPF (sNPF) and NPF (Reale, Chatwin & Evans, 2004). Thus missing neuropeptide receptors can only be used to validate the absence of a neuropeptide ligand, if these receptors are activated exclusively by that ligand.

The loss of a gene can in principle only be demonstrated by flawless genome assemblies (they don’t exist), however, there is an alternative that is almost perfect. It exists in the analysis of the original genomic reads obtained for the assembly. When those reads are very numerous short reads, the chance that there is not a single read that covers the gene in question becomes extremely small and thus negligible. The only remaining problem than is the question, whether or not the gene in question can be reliably identified from a single short read. For the most GPCRs the answer to this question is yes, as the sequences of the seven transmembrane regions are strongly conserved and there are always a couple of them that one can unambiguously identify as being part of a particular receptor. Obviously, this might not work if all the individual transmembrane regions of a GPCR were coded by two exons interrupted by an intron. But this is not the case for the GPCRs analyzed here. An illustration of this method is provided as a in Fig. S1.

For the analysis of the absence of neuroendocrine convertase PC1/3 a similar procedure was applied. This was relatively easy, as this protein has a very well conserved primary sequence.

To demonstrate the absence of a particular neuropeptide gene in this fashion is much more difficult. First, many neuropeptide genes code for a single neuropeptide and the remainder of the precursor is often too poorly conserved to be recognized reliably in short genomic reads from species that are not closely related. Secondly, in some cases the sequence coding the peptide or its most conserved parts, may be interrupted by an intron. For example, the genomic sequences coding the NPF family all have a phase 2 intron in the triplet coding the Arg residue of the C-terminal Arg-Phe-amide, making identification of genomic sequences coding this peptide more difficult. A similar intron is present in the elevenin gene. Thirdly, some neuropeptides are not only small but are also made up of amino acids that have very degenerate codons, this is the case for sNPF. Finally, sometimes conserved amino acids in a particular neuropeptide are no longer conserved, as is the case for allatotropin in honeybees and other Hymenoptera (Veenstra, Rodriguez & Weaver, 2012). On the other hand, when dealing with a larger peptide, that is, structurally well conserved during evolution this would provide an additional argument for its absence.

### Sequence comparisons

For comparing the sequences of various neuropeptides I have used Seaview (Gouy, Guindon & Gascuel, 2010) and the figures it produces. The different colors that are used to identify amino acid residues with similar chemical characteristics (acidic, basic, aromatic, aliphatic, etc.) provide good visualization of conserved amino acid sequences when absolute conservation of residues is limited.

## Results

### General comments

Neuropeptides have previously been identified and sequenced for some of the species analyzed here. The sequences of the two sNPFs and the two AKHs that were identified from the potato beetle (Gäde & Kellner, 1989; Spittaels et al., 1996a) are exactly the same as those predicted from the genome. On the other hand there is no *Leptinotarsa* gene coding an allatotropin with the same structure as the allatotropin ortholog from *Locusta migratoria*, in spite of suggestions otherwise (Spittaels et al., 1996b) and this species has a proctolin gene that predicts a classical proctolin and not the previously reported [Ala^1^]-proctolin (Spittaels et al., 1995a). Two other putative neuropeptides that were reportedly isolated from this species could neither be identified in any of the genomic or transcriptomic sequences analyzed here (Spittaels et al., 1991, 1995b).

The sequences of *Tenebrio* AKH, myosuppressin, three pyrokinins, DH37 and DH47 as predicted here from the transcriptome are identical to those reported previously, the only difference being that the transcriptome suggests a C-terminal amide for DH47 instead of the reported C-terminal acid (Gäde & Rosiński, 1990; Furuya et al., 1995; Furuya, Schegg & Schooley, 1998; Weaver & Audsley, 2008; Marciniak et al., 2013). The AKH sequences from *Coccinella, Harmonia*, and *Nicrophorus* (Neupert, 2007; Gäde, Šimek & Marco, 2015) are also identical to those deduced from the genome sequences.

The majority of the neuropeptide precursors seem quite similar in structure between the different species. Those will not be commented upon, but their sequences can be found in Table S2 and the Supplementary Figures. To facilitate interpretation of the data several figures include a simplified phylogenetic tree of the species analyzed. This tree is based on the extensive phylogenetic tree recently published for Coleoptera (Zhang et al., 2018). When I use the term closely related species in the text, this is short hand for species that are neighbors on the simplified phylogenetic trees.

### Significant changes in peptide sequences

#### Pigment dispersing factor

**Figure 1:**
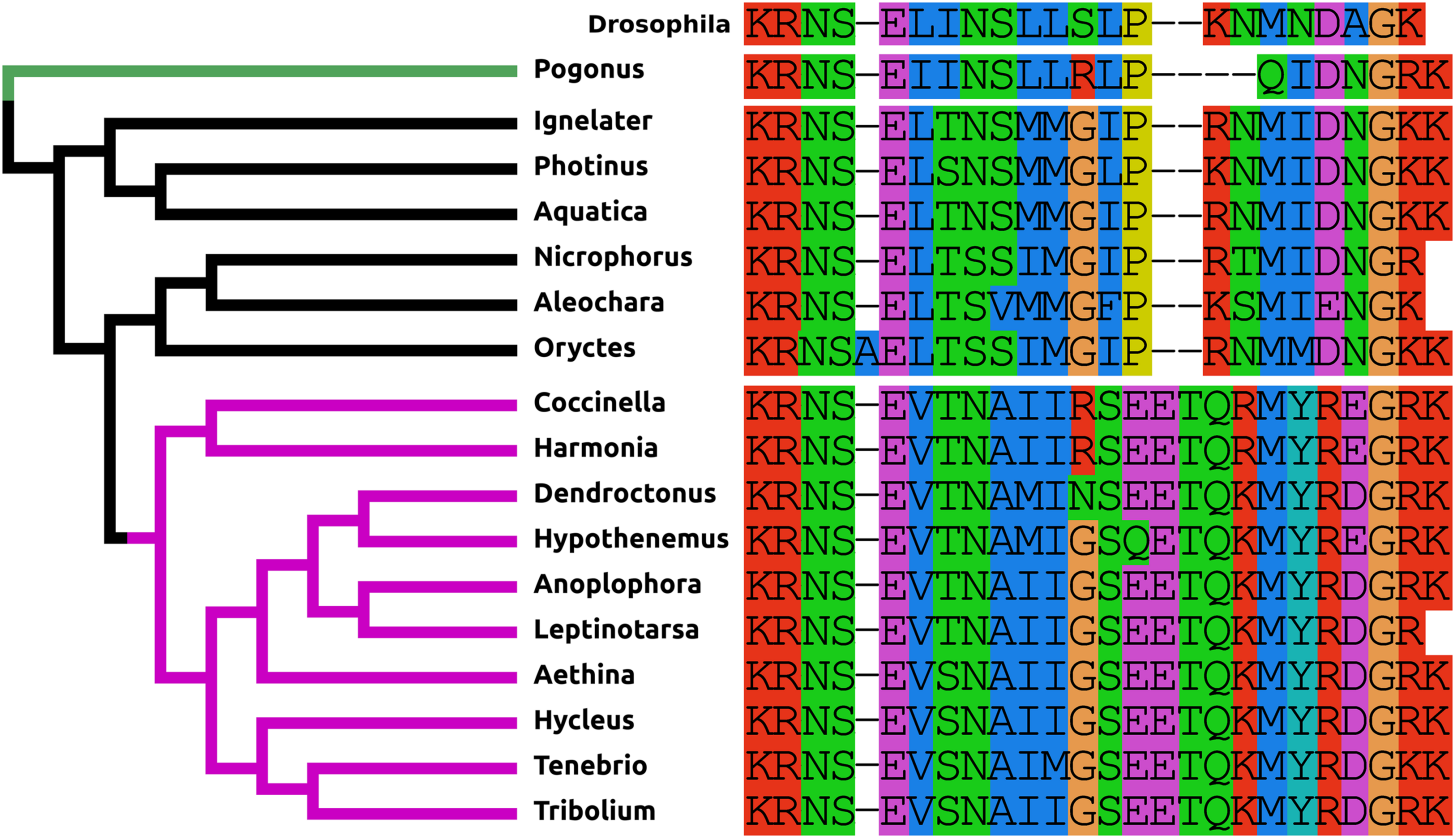
Coleoptera pigment dispersing factors. Alignment of the predicted PDFs from the 17 Coleoptera species as obtained by conceptual translation of their putative transcripts. *Drosophila* PDF has been added for comparison. The sequences include the processing sites on both side of the mature peptide. The species are arranged according to their position on the phylogenetic tree as established by Zhang et al. (2018). Note the differences between the predicted PDF from the single Adephaga species, green on the tree, the Cucujiformia, purple part of the tree, and the remaining Polyphaga species.

The PDF present in *Tribolium* and other Cucujiformia has two more amino acid residues than the *Drosophila* peptide and differs from it especially in its C-terminal half (Fig. 1). This explains why it wasn’t identified in a previous study (Li et al., 2008). In the other Polyphaga it is more similar to the *Drosophila* peptide, but in *Polygonus* it is two amino acids shorter than in *Drosophila*.

#### Neuropeptide F

**Figure 2:**
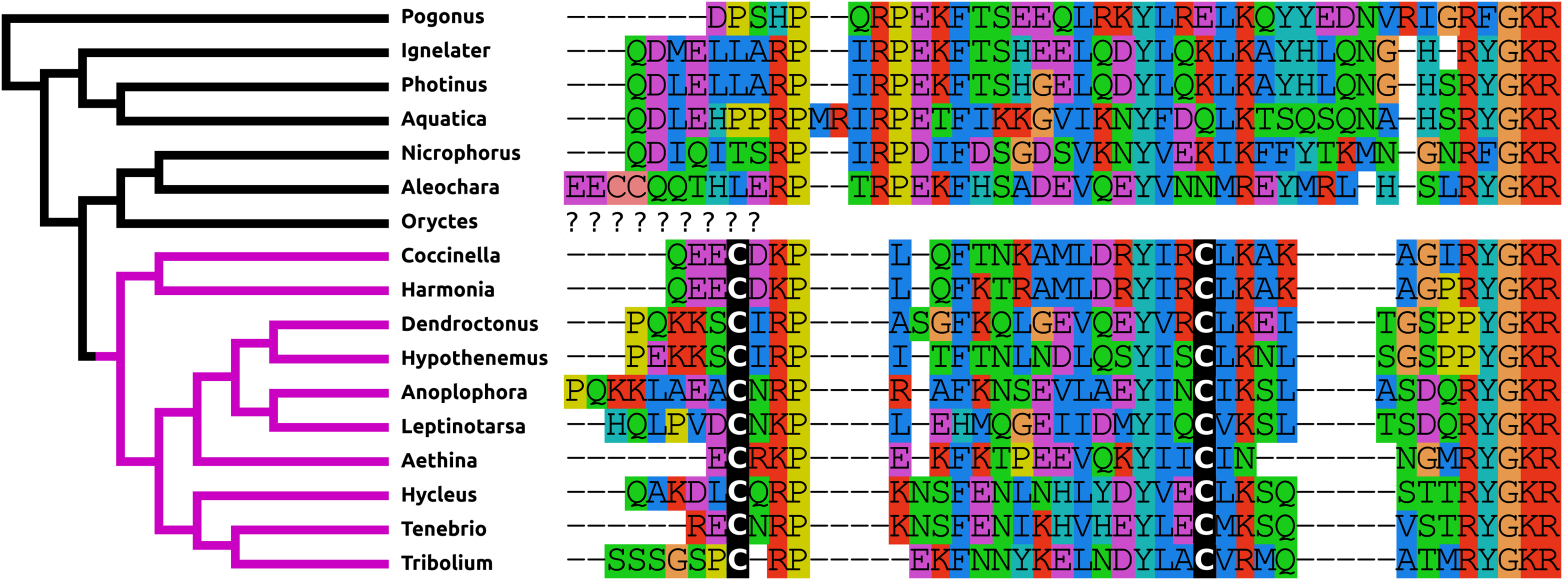
Coleoptera neuropeptide F. Alignment of the predicted NPFs from 16 species as obtained by conceptual translation of their putative transcripts. The sequences include the processing sites on C-terminal site of the mature peptide. The species are arranged according to their position on the phylogenetic tree as established by Zhang et al. (2018). Note the differences between the predicted NPF from Cucujiformia where the peptide has acquired a cysteine bridge with those from the other species. An NPF gene was not found in the *Oryctes* genome, even though this species does have an NPF receptor gene.

The structure of NPF has changed even more than that of PDF. It is relatively common for a Phe to residue change into a Tyr and vice versa and so the mutation of the C-terminal Arg-Phe-amide into Arg-Tyr-amide in most species studied here, is not unusual. More drastic is the presence of disulfide bridge in the N-terminal of NPF in the Cucujiformia and the mutation of the C-terminal Arg-Tyr-amide into a Pro-Tyr-amide in the two Curcuclionids. The primary sequence similarity of the predicted peptides to each other and other insect NPFs, as well as the characteristic phase 2 intron in the Arg residue (Pro in the Curcuclionids) of the C-terminal of these peptides confirm that these are true NPF orthologs (Fig. 2). I was unable to find an *Oryctes* NPF gene, although an NPF receptor seems to be still present in this species. Given the enormous structural variability of this peptide in Coleoptera it is not clear whether this is because the NPF gene was lost, or whether in this species the peptide has undergone even larger sequence changes.

#### ACP

**Figure 3:**
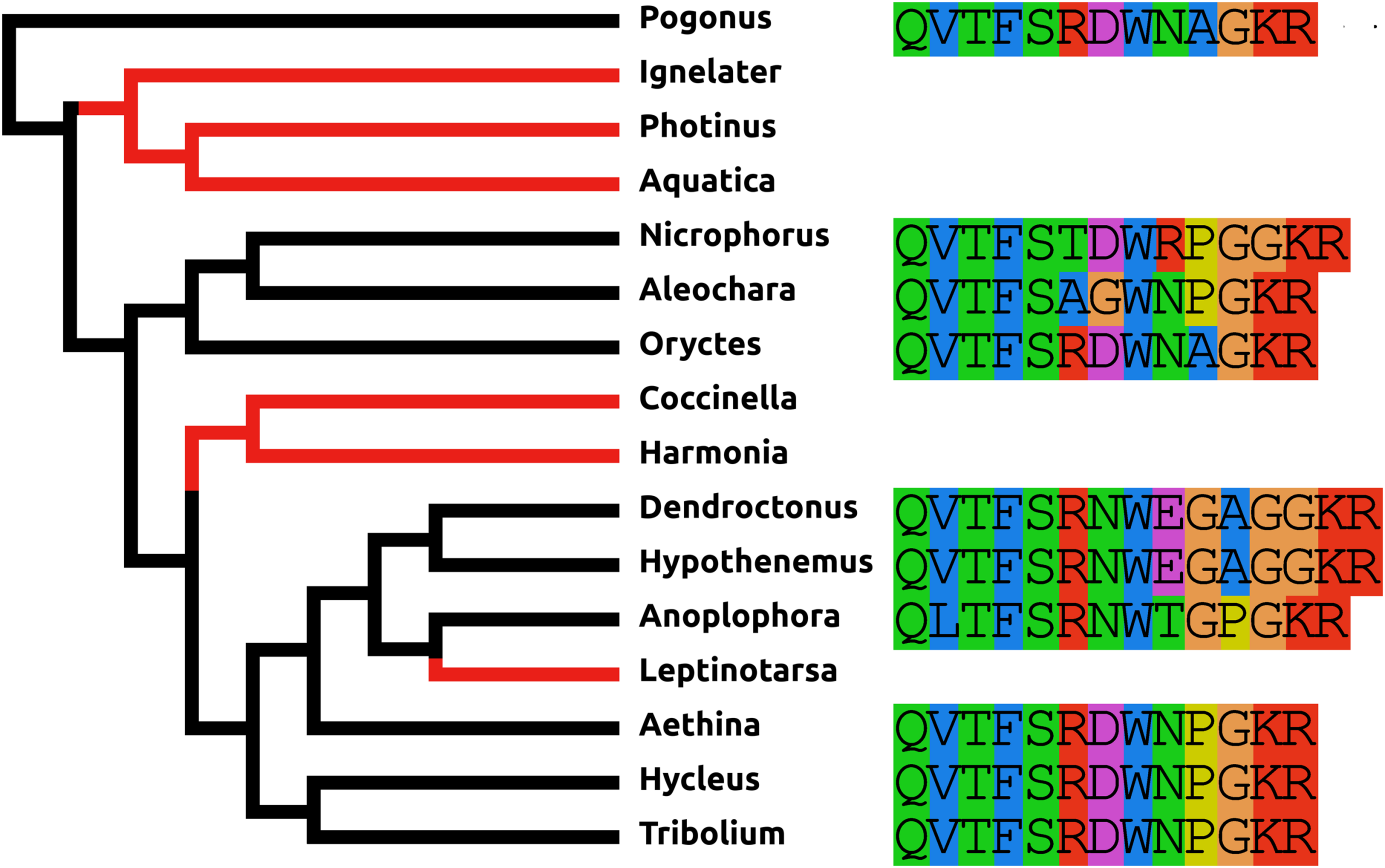
Coleoptera ACPs. Alignment of the predicted Coleoptera ACPs as obtained by conceptual translation of their putative transcripts. The sequences include the processing sites on C-terminal site of the mature peptide. Cleavage of the N-terminal is performed by a signal peptidase. The species are arranged according to their position on the phylogenetic tree as established by Zhang et al. (2018). Tree branches have been made red where the peptide and its receptor were lost from the genome, which must have occurred on at least three occasions. Note that the peptide sequence is not very well conserved.

AKH/corazonin-related peptide (ACP) is a peptide that has been lost independently at least three times and in those species where the gene is still present the predicted peptide sequences are quite variable (Fig. 3).

#### Baratin or NVP-like precursor

Baratin is a small neuropeptide initially isolated from the cockroach *Leucophaea maderae* (Nässel, Persson & Muren, 2000) that has been shown to be produced from a large neuropeptide precursor that has been called NVP-like in *Tribolium* (Li et al., 2008). This neuropeptide precursor is well conserved in Coleoptera (Fig. S2), except that in *Dendroctonus* it is lacking the last part as deduced from both the genome and transcriptome sequences (note there is another baratin precursor at NCBI, supposedly also from *Dendroctonus*, however, analysis of the various SRAs from which this transcriptome is made shows that one of them (SRR2044898) contains in addition to *Dendroctonus* a second unidentified species from which this baratin precursor transcript originates).

#### Calcitonin B

**Figure 4:**
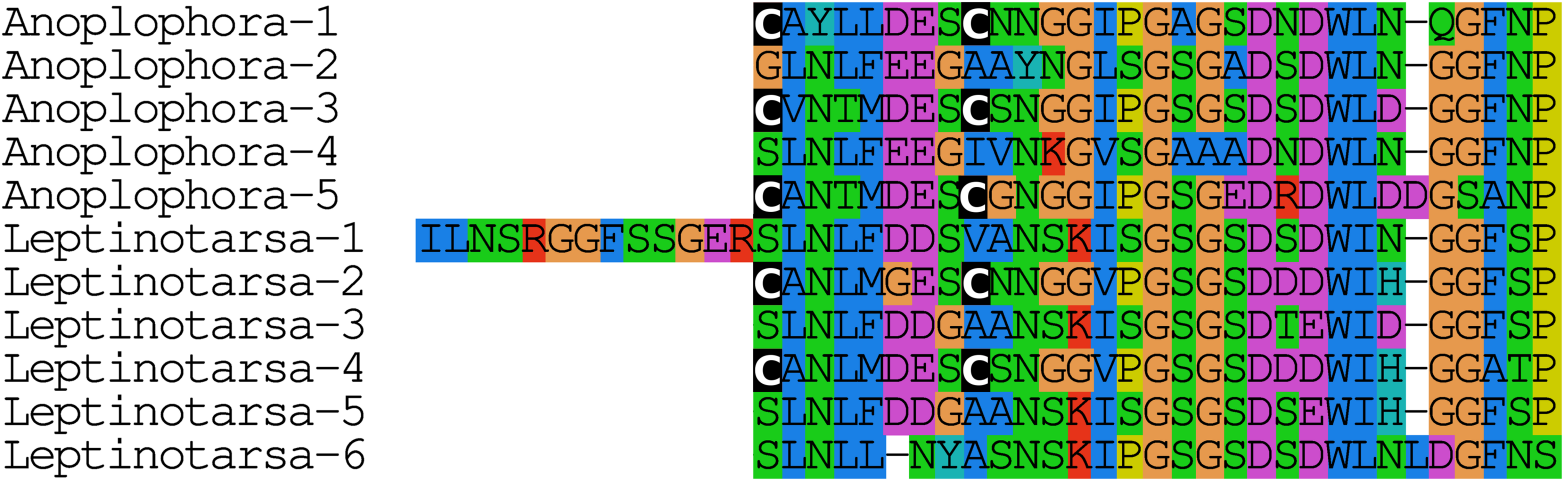
Unusual calcitonin sequences. Alignment of calcitonin B sequences encoded by the *Leptinotarsa* calcitonin B gene and the first such gene from *Anoplophora*. Note that with the exception of the sixth peptide from *Leptinotarsa*, these peptides have well conserved amino acid sequences, but that some of them have a cysteine bridge in the N-terminal of the molecule, while others have not. All peptides are predicted to have a C-terminal amide.

The *Leptinotarsa* and one of the *Anoplophora* calcitonin genes encode not only typical calcitonin peptides, but also a number of structurally very similar peptides that lack the disulfide bridge in the N-terminal portion of the molecule (Fig. 4).

#### Elevenin

**Figure 5:**
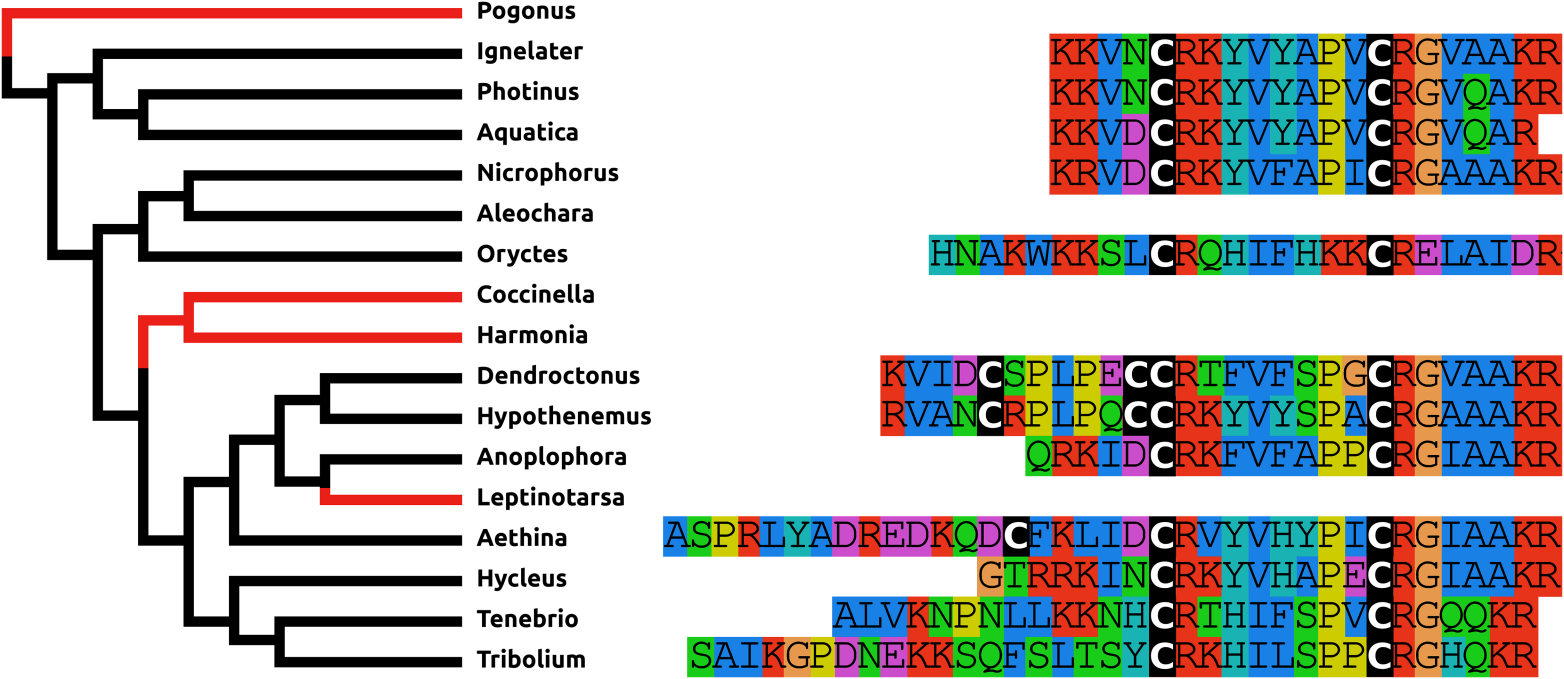
Coleoptera elevenins. Alignment of the predicted Coleoptera elevenins as obtained by conceptual translation of their putative transcripts. The sequences include the processing sites of peptides; where these are lacking on the N-terminal, cleavage is obtained by a signal peptidase. The species are arranged according to their position on the phylogenetic tree as established by Zhang et al. (2018). Tree branches have been made red where the peptide and its receptor were lost from the genome, which must have occurred on at least three occasions. Note that the peptide sequence is not very well conserved and that in both *Dendroctonus* and *Hypothenemus* an additional cysteine bridge has been added to the peptide.

Like ACP, elevenin has been lost independently at least three times and in those species where this gene is still present the predicted elevenin sequences are also very variable (Fig. 5).

#### Myosuppressin

Myosuppressin is always located at the very end of its precursor and in virtually all insect species after the Gly residue that will be transformed in the C-terminal amide the precursors ends with two, three, or occasionally four dibasic amino acid residues. Surprisingly this is not so in Coleoptera, where all myosuppressin precursors terminate with a few additional amino acid residues after those dibasic amino acid residues (Fig. S3).

#### Orcokinin convertase cleavage sites

In those species where the organization of the exons of the orcokinin gene could be established, it was similar to the one described previously for other insects (Veenstra & Ida, 2014). Due to the presence of numerous copies of orcokinin B peptides, sequences of this gene are usually very difficult to assemble using short reads and this explains the problems with the orcokinin genes of *Oryctes* and *Aleochara*, although in both cases the presence of orcokinin was established. What makes these genes interesting is the convertase cleavage sites in the orcokinn B precursors. Proteolytic processing of neuropeptide from their precursors occurs at specific dibasic amino acid residues, usually a Lys-Arg pair. When processing occurs at singe Arg residues, as is the case for most orcokinin B precursors, empirical rules describe that other dibasic amino acid residues need to be located nearby in the precursor (Veenstra, 2000). However, orcokinin B precursors do not conform, which suggests that they are processed by a different convertase than the one processing the majority of insect neuropeptide precursors. Interestingly, in the two Coccinellids studied here, *Harmonia* and *Coccinella*, as well at least another, *Serangium japonicum* (GGMU01110504.1), the convertase cleavage sites have been replaced by the more classical Lys-Arg sites. In *Aethina* a few single Arg cleavage sites are still present, but the majority are also Lys-Arg pairs (Fig. S4). This suggested that this second convertase may have been lost. The two most common neuroendocrine convertases are PC1/3 and PC2; both are commonly present in insects (Veenstra, 2017), but PC1/3 is absent from *Drosophila*, a species in which the orcokinin B precursor also has Lys-Arg convertase cleavage sites (Veenstra & Ida, 2014). When looking for these two convertases in Coleoptera, it became clear that PC1/3 is similarly lacking in Coccinellids but present in the other species, including *Aethina*.

#### Periviscerokinin (Capa peptides)

The periviscerokinins have often the typically the C-terminal sequence FPR(V/L/I)amide, but although some of the Coleoptera peptides have this sequence (Fig. S5), others have not. Detailed analysis of three receptors activated by pyrokinins, tryptopyrokinins, and periviscerokinins in *Tribolium* shows that a periviscerokinin with a C-terminal LTPSLRVamide is as good a ligand as the MVSFPRIamide (Jiang et al., 2014). This analysis also reveals that none of these receptors preferentially recognizes the tryptopyrokinins, which in *Drosophila* and mosquitoes have a dedicated receptor (Cazzamali et al., 2005; Olsen et al., 2007). Unfortunately, I was unable to reconstruct a complete periviscerokinin transcript for neither *Leptinotarsa* nor *Harmonia* from either the genomic or the transcriptomic data.

#### Proctolin

The predicted proctolin sequences of *Harmonia, Coccinella*, and *Oryctes* deviate from the classical peptide. This is described in more detail in the following section on gene losses.

### Gene losses

#### Unambiguous gene losses

**Figure 6:**
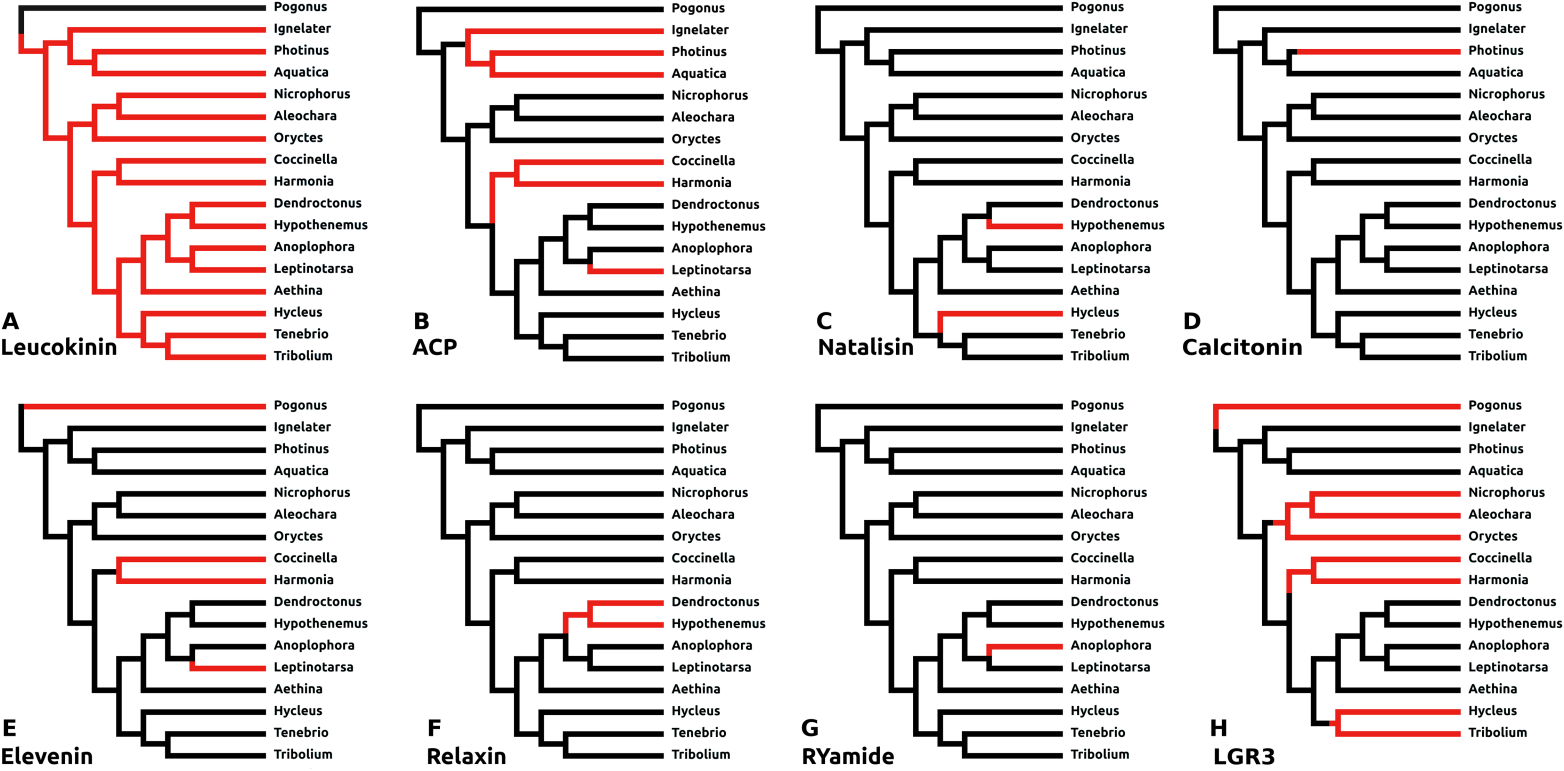
Neuropeptide losses. Loss of eight neuropeptide signaling systems in at least one of the 16 Coleoptera species for which a genome is available. Black branches on the tree indicates the presence of the neuropeptide gene, while red branches indicate its absence. In the case of LGR3 only the receptor could be studied, but for ACP, elevenin, RYamide, natalisin, leucokinin, and relaxin both ligand and receptor genes were absent from the indicated genomes. Relaxin and calcitonin are neuropeptides for which the receptor has not been formally deorphanized in insects. Their identities have been deduced from sequence similarity between the insect ligands with their well-known vertebrate homologs, sequence similarity between their putative receptors and their vertebrate homologs and the systematic co-occurrence and co-absence in the same genomes of each ligand with its putative receptor (cf Veenstra, 2014). (A) Leucokinin, (B) ACP, (C) natalisin, (D) calcitonin, (E) elevenin, (F) relaxin, (G) RYamide, and (H) LGR3.

There are a number of instances in which genes could not be found in the assembled genome of a species. In six cases this concerns neuropeptides with a known and unique receptor which is also absent from the same genomes that lack the genes for the ligands. It was previously reported that the *Tribolium* lacks both ligand and receptor genes for allatostatin A, corazonin and leucokinin (Li et al., 2008; Hauser et al., 2008). The first two were found to be absent from all Coleoptera studied here, while both leucokinin and its receptor were found in *Pogonus*, the only species outside the Polyphaga suborder for which a genome is available. However, neither leucokinin nor its receptor was found in any of the other species. Leucokinin is also present in other species that do not belong to the Polyphaga suborder. As noted above both ACP and elevenin were lost independently at least three times, while natalisin was lost at least twice in Coleoptera (Fig. 6). Interestingly in *Photinus* there is still a remainder of the original calcitonin gene. It is clearly defective as it misses essential parts and it is no longer expressed, while the putative receptor (*cf* Veenstra, 2014) is completely gone. A similar situation occurs with the relaxin gene in *Sitophilus oryzae*; there also a remainder of non-functional relaxin gene is still present, but its putative receptor (*cf* Veenstra, 2014) is absent.

#### Dilp8 orthologs

The structure of dilp8, *Drosophila* insulin-like peptide 8, is very poorly conserved and it has so far not been detected outside flies. LGR3 (Leucine Rich Repeat GPCR-3) has been identified as the receptor for this peptide (Vallejo et al., 2015) and this receptor, although absent from *Tribolium*, was found in a number of species (Fig. S7), suggesting that it got independently lost on at least four occasions (Fig. 6).

#### Eclosion hormone

Most Coleoptera have two Eclosion hormone genes (Fig. S8), but the second gene appears to be missing in *Coccinella, Harmonia*, and *Dendroctonus*, while in *Hycleus* there is still a sequence that can be recognized as once have being part of such a gene, but it is probably no longer functional as (1) there are no expression data for the second gene, while such data is abundant for the first one, and (2) it seems to lack an essential intron donor site. In many genomes the two genes are located on the same contig. All four possible configurations (head to head, tail to tail, one upstream from two, two upstream from one) are present, but there is no clear pattern.

#### Elevenin

The presence of elevenin in *Oryctes* and *Aleochara* is not clear. On the one hand, there are genomic sequences in *Oryctes* that code for what looks like parts of an elevenin precursor, even though the predicted elevenin peptide deviates even more from the elevenin consensus sequence than the average Coleopteran elevenin. On the other hand, no traces were found of a putative elevenin GPCR. Therefore, elevenin may well be also absent from *Oryctes*. A similar but different problem occurs with *Aleochara*, here a putative elevenin GPCR is present in the genome, but the elevenin precursor could not be found. This is not so surprising as its precursor is hardly conserved outside the sequence of the neuropeptide itself and even that sequence is so poorly conserved within the Coleoptera (Fig. 5) that the characteristic intron splice site inside the neuropeptide sequence is often needed to confirm that it is indeed elevenin. However, the same intron splice site makes finding homologous sequences much more difficult.

#### sNPF

In all species an sNPF GPCR can be identified, but the sNPF precursor (Fig. S9) was found in neither the *Photinus* nor the *Aquatica* genome. These two Coccinellid species are relatively closely related and the absence of the sNPF precursor from both suggests that it was already lost in their last common ancestor. It seems unlikely to be a case of genome assembly problems, as despite several efforts not a single transcriptome read could be found that could represent an sNPF mRNA. There are two possible explications. The first one is that the sNPF precursor has been lost in these two species but its receptor is still being used by a different peptide, for example, another N-terminally extended RFamide. The second possibility is that the sequence of the peptide has undergone so many structural changes, that it is now impossible to find it using the BLAST program for homology searches.

#### Proctolin

Proctolin was the first insect neuropeptide for which a complete chemical structure was determined (Starratt & Brown, 1975). It is commonly present in insects, although it seems to be absent from at least some Lepidoptera and Hymenoptera (Roller et al., 2008; Kanost et al., 2016; Hummon et al., 2006; Hauser et al., 2006, 2010; Schmitt et al., 2015). Its pentapeptide sequence (Arg-Tyr-Leu-Pro-Thr) has been well conserved during evolution and is exactly the same in Chelicerates, Myriapods, Decapods, and insects (Veenstra, 2016a, 2016c). It is therefore interesting to see that in Coccinellids the predicted sequence of this peptide has mutated to [Ser^4^]-proctolin. In *Oryctes* the proctolin precursor also predicts a non-classical proctolin, in this case [Ala^5^]-proctolin. In all three species these sequences are deduced from both the genome and transcriptome sequences. On the other hand, the overall structures of these putative proctolin precursors are well conserved (Fig. S10). Nevertheless, no proctolin receptors could be found in either *Oryctes* or the two Coccinelid species.

#### Other peptides that are absent from Coleoptera

Calcitonin A and CCRFamide have never been found in Holometabola, and they were neither found here. In Coleoptera tryptopyrokinin coding sequences were only found as part of the periviscerokinin and pyrokinin genes and hence a specific tryptopyrokinin gene as exists in termites and locusts (Veenstra, 2014) was not found in Coleoptera. Of the three allatostatins Cs (Veenstra, 2016b) only CC and CCC were found and neither did we find any evidence for a second NPF gene. EFLamide is difficult to find, because its conserved sequence is so short (Veenstra, 2019). Insect species that have an EFLamide gene also have an ortholog of the *Platynereis* EFLamide GPCR (Bauknecht & Jékely, 2015), but such an ortholog is missing from all the Coleoptera genomes studied here.

The recently described putative neuropeptide precursor RFLamide (Liessem et al., 2018) is easily detectable in most Coleoptera (Fig. S11), but was not found in either of the two Curculionids, *Hypothenemus* and *Dendroctonus*. Hence, it is likely that this gene is missing from those two species as well as from other Curculionidae.

### Gene duplications

#### AKH

**Figure 7:**
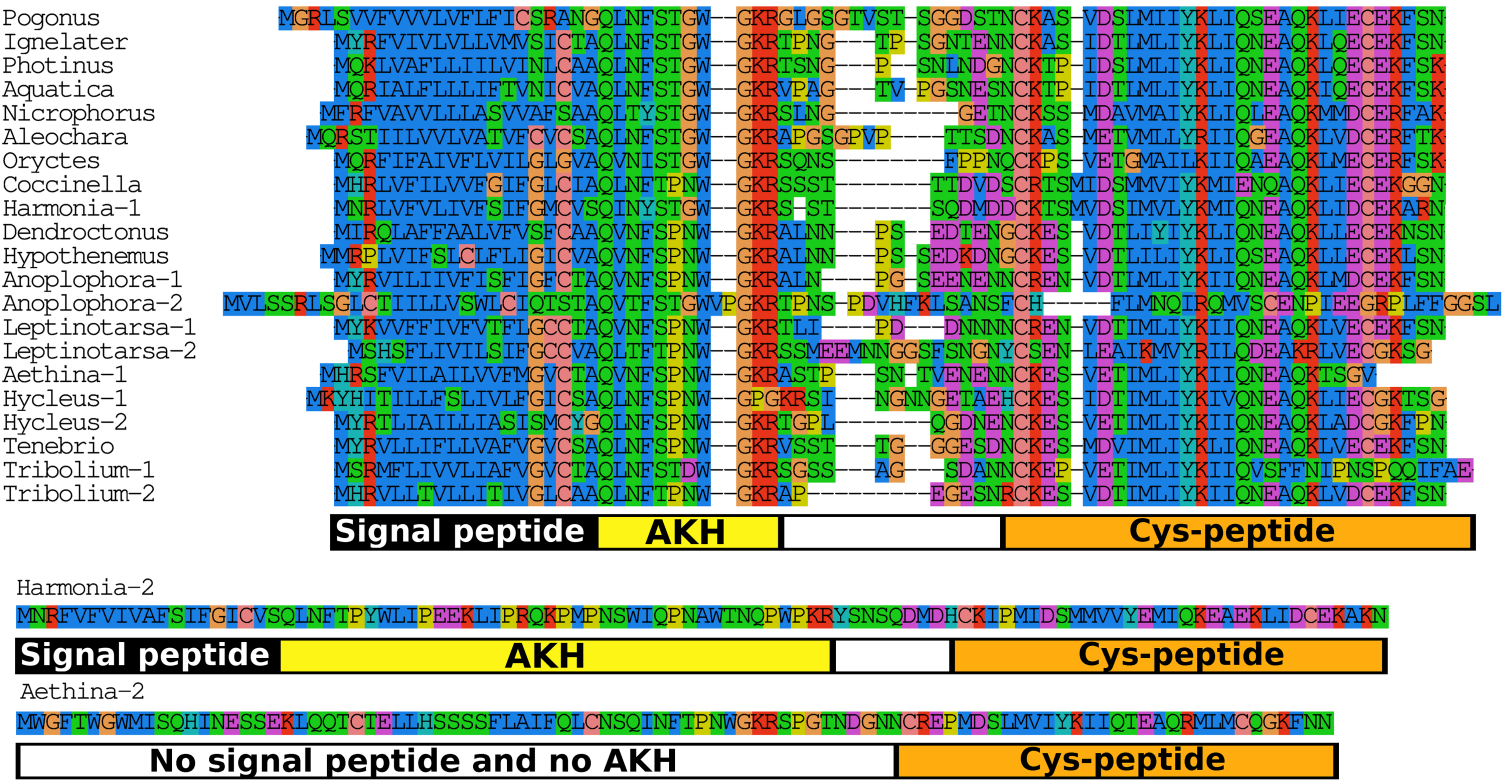
Adipokinetic hormones. Putative AKH precursor sequences found in the 16 genomes and the *Tenebrio* transcriptome. Most of the sequences are relatively short and are aligned in the top part of the figure. Those sequences typically consists of four different parts: the signal peptide, followed immediately by the AKH sequence and a glycine residue, that is, transformed into the C-terminal amide and a convertase cleavage site, a variable region, and at the end the sequence of a well conserved peptide forming a disulfide bridge. These different regions are indicated below the alignment. Two sequences that show homology to AKH precursors deviate significantly from this pattern. The second *Harmonia* AKH-like precursor is predicted to produce a very long AKH-like peptide, while the second *Aethina* precursor lacks a signal peptide and hence can not produce AKH.

When the putative Coleopteran AKH precursors are aligned it is evident that they consist of four different regions, the signal peptide, the AKH peptide sequence with its processing site consisting of the GKR triplet, a hydrophilic connecting peptide (C-peptide) and a more hydrophobic disulfide bridge containing sequence (Cys-peptide). It is noticeable that the sequences of the signal peptides, AKHs and the Cys-peptides are very well conserved (Fig. 7), albeit that there are a number of exceptions. The most glaring examples are the second putative *Harmonia* AKH gene, which obviously can not encode an AKH, and the putative *Aethina* AKH precursor that is predicted to have no functional signal peptide.

#### Bursicon

The bursicon sequences are all very similar, as expected from this well conserved and essential insect hormone (Figs. S12 and S13). *Oryctes* is the only species, that is, noteworthy in that it has two bursicon A genes the start ATGs of which are 4,184 nucleotides apart on the same contig. When one compares the predicted mature protein sequences, it is clear that the second one has several amino acid changes which in all the other proteins are well conserved (Fig. S12). It is impossible to know which of these two genes is most expressed, as all the Bursicon A intron splice sites in both genes are ignored by the various RNAseq reads. The only Trinity transcript generated from this genomic region that has an intron reveals that it was generated from the opposite DNA strand. So possibly all the RNAseq reads present in the only public transcriptome SRA (SRR2970555) that cover the Bursicon A genomic region of this species are generated from the opposite strand and thus originate from a different gene.

#### Calcitonin

Two genes coding calcitonin B are present in *Anoplophora, Hycleus, Tribolium*, and *Tenebrio*, they are described in greater detail in the section on paracopy duplication.

#### CCHamide 2

**Figure 8:**
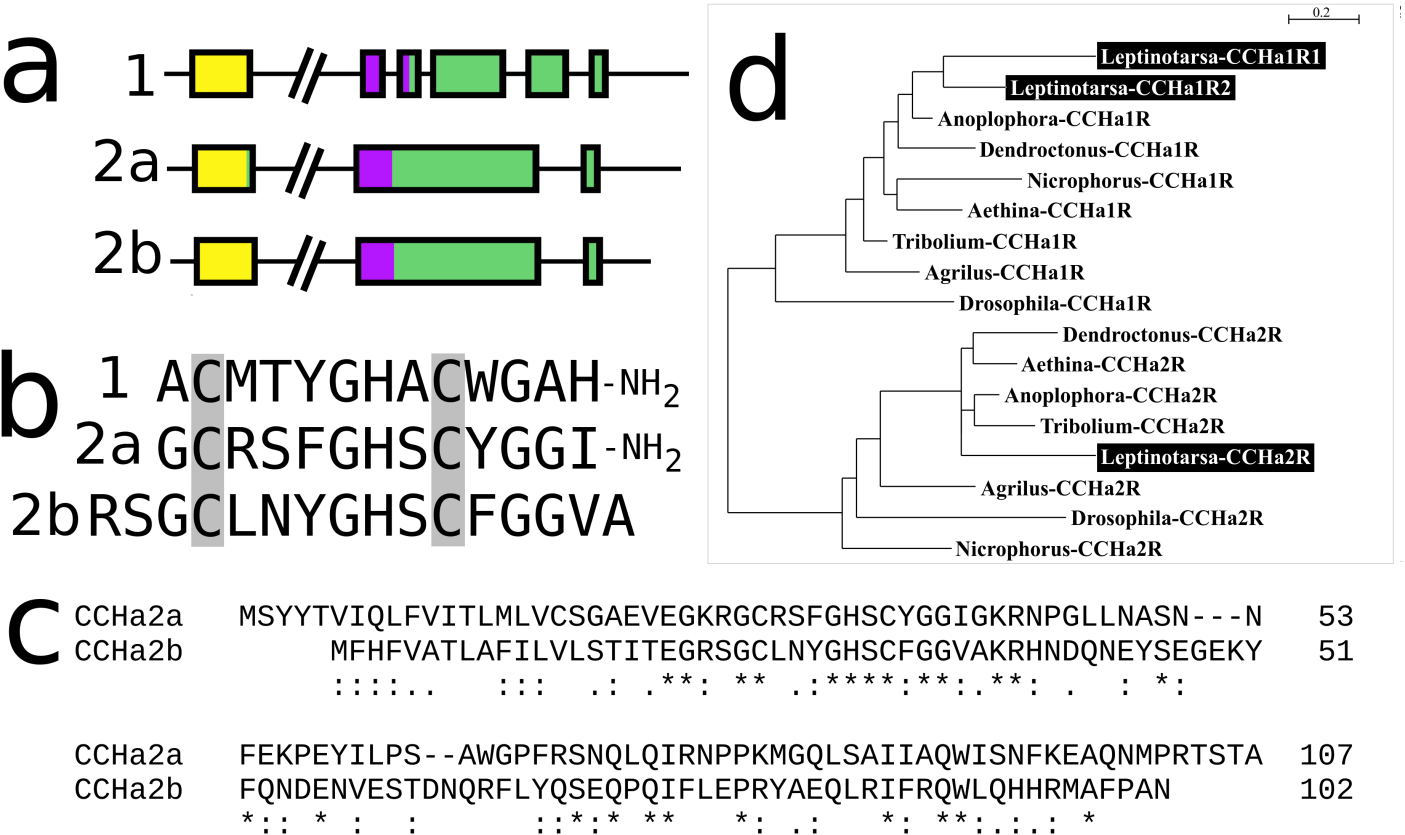
*Leptinotarsa* CCHamides. Duplication of the CCHamide 2 neuropeptide and the CCHamide 1 receptor genes in *Leptinotarsa*. (A) Schematic organization of the three CCHamide genes in *Leptinotarsa*. Horizontal lines indicate introns and other untranslated DNA sequences, the boxes correspond to translated exons. Yellow indicates sequences corresponding to the signal peptides, purple correspond to the mature peptide sequences and green the remainder of the precursors. Note that the gene organizations of CCHamides 2a and 2b are very similar. (B) Direct comparison of the predicted mature peptides. Note that CCHamide 2b lacks a C-terminal amide, that is, present in all other CCHamides. (C) Direct comparison of the predicted precursors for CCHamides 2a and 2b. Note that, although similar, these sequences are significantly different. Asterisks (*) indicate identical amino acid residues, double dots (:) and single dots (.) indicated conserved and semi-conserved substitutions of amino acid residues, respectively. (D) Simple phylogenetic tree for CCHamide receptors from *Leptinotarsa*, other Coleoptera and *D. melanogaster*. Note that the two *Leptinotarsa* CCHamide 1 receptors are more similar to one another than to any of the other Coleoptera CCHamide 1 receptors, including the one from *Anoplophora*. Nucleotide sequences for these receptors are: ACZ94340.1, XP_023021283.1; XP_017768833.1, XP_019758999.1; XP_025836439.1, XP_008197479.1, XP_023310960.1, XP_019880954.1; XP_018332710.1, ERL86066.1, XP_019880542.1, XP_023313148.1, XP_015838444.1; XP_017779615.1, AAF57819.3, XP_023023025.1, and the *Leptinotarsa* CCHamide 2 receptor which is present in the Supplementary Excel File.

In *Leptinotarsa* the CCHamide 2 gene is duplicated (Fig. S14) and so is the CCHamide 1 receptor. One of the two CCHamide 2 gene codes for a typical CCHamide 2 neuropeptide, the second CCHamide 2 peptide lacks the typical C-terminal amide. Phylogenetic tree analysis of CCHamide receptors shows that the two *Leptinotarsa* CCHamide 1 receptors are more similar to one another than to the *Anoplophora* ortholog, thus suggesting that the duplication of this receptor is relatively recent (Fig. 8).

#### Insulin-related peptides

Insects have three different types of insulin, two of which act, predominantly or exclusively, through GPCRs, these are relaxin and the dilp8 orthologs. The third type acts through a classical tyrosine kinase receptor and in most insect species the latter insulin genes are amplified. In Coleoptera their numbers range from two in *Aquatica* and *Pogonus* to ten in *Anoplophora*. The primary amino acid sequence is in general not very well conserved, making it difficult if not impossible to make reliable trees of insect insulin genes. However, it is clear that genes were lost and added on multiple occasions. The strong sequence divergence of these proteins implies that one can only make phylogenetic trees for relatively closely related species. Such a tree for *Hycleus, Tribolium*, and *Tenebrio* (Fig. S15) shows that almost all of their insulin genes are direct orthologs from one another. A similar tree made for the insulin sequences from *Dendroctonus, Hypothenemus, Anoplophora, Leptinotarsa*, and *Aethina* similarly shows shared ancestors for several of their insulin genes as well as recently amplified insulin genes in *Leptinotarsa*, *Anoplophora*, and *Aethina* (Fig. S16).

#### Myosuppressin

In *Leptinotarsa* the myosuppressin gene has been amplified and its genome now has four such genes, one that produces a classical myosuppressin and three others that at first sight seem to code for a smaller analog of myosuppressin. On the basis of the described specificity of neuropeptide convertase (Veenstra, 2000), it is also possible that they produce N-terminally extended myosuppressins, as Lys-Arg cleavage sites followed by an aliphatic amino residue are rarely cleaved, and this even more unlikely for the precursor in which the putative Lys-Arg cleavage site has mutated into a Lys-Lys site (Fig. S3). All four genes are expressed as shown by the various RNAseq SRAs. Interestingly, this gene is also amplified in the Scarabaeid *Onthophagus taurus*, where there are at least three genes that express a myosuppressin precursor. Thus the myosuppressin gene was amplified independently at least twice in Coleoptera.

#### Neuroparsin

The neuroparsin gene is present as a single copy in all species, except *Oryctes* where it is duplicated (Fig. S17) and perhaps even triplicated, as the second gene is present in two copies in the genome assembly; those may represent either two quite divergent alleles of the same gene or perhaps more likely a gene duplication. In the RNAseq SRA of this species (SRR2970555) these three sequences are represented by 44, 19, and 11 half spots, respectively.

#### Pyrokinin

The basic beetle pyrokinin gene has three coding exons, the two introns in between are phase 1 and phase 0, which makes amplification of the intermediate coding exon, such as occurred in the periviscerokinin gene, very difficult. The first coding exon contains the signal peptide, the second a copy of tryptopyrokinin and the last one three pyrokinin paracopies. The *Pogonus* gene has three coding exons, but the precursor only codes for two pyrokinin paracopies. The tryptopyrokinin has also been lost from the Coccinelid precursors, one of two pyrokinin precursors in *Nicrophorus, Dendroctonus, Hypothenemus, Anoplophora*, and *Leptinotarsa*, all *Photinus, Aquatica*, and *Ignelater* precursors and probably three out of five in *Aethina*. It thus looks like that in several Coleoptera species evolution favored the production of pyrokinins over that of tryptopyrokinin from these genes. The pyrokinin gene is amplified in five of the species studied here; most of these are segmental amplifications, but in *Oryctes* there is one gene that has no longer any introns and may have originated from retroposition (Fig. S18).

#### Relaxin

The gene coding for relaxin (the ortholog of *Drosophila* insulin-like peptide 7) is duplicated in *Aethina* (Fig. S19). Both copies look like they can produce functional proteins and both genes are expressed (there are 170 and 89 reads for the coding sequences of relaxin-1 and -2, respectively, in SRR1798556).

#### Vasopressin

Genes encoding vasopressin-related peptides were found in all species analyzed (Fig. S20). In all of them, except *Leptinotarsa*, these genes code for CLITNCPRG-amide, the peptide that was identified from *Locusta migratoria* (Proux et al., 1987). In the Colorado potato beetle two such genes were found and they code for two different vasopressin-like molecules CLITNCPKG-amide and its analog CLITNCPIG-amide. Interestingly, various vasopressin antisera that were used to label vasopressin-immunoreactive neurons in this species labeled the two vasopressin-specific neurons only weakly while the adjacent pyrokinin containing neuroendocrine cells that have a C-terminal PRLamide sequence intensely (see, e.g., fig. 3 from Veenstra, Romberg-Privee & Schooneveld, 1984), while the same antisera stain the vasopressin specific cells in the *Locusta* just as intensely as the ones producing pyrokinin (Veenstra, 1984). Each of the two *Leptinotarsa* peptides could explain these results, the Lys-analog, as the Lys residue is likely to be cross-linked by formaldehyde and hence no longer immunoreactive, and the Ile-analog because it lacks the basic amino acid residue that is likely important for immunoreactivity. Counts of RNAseq reads in *Leptinotarsa* SRAs reveals twice as many reads for the Ile-analog as for the Lys-analog (652 vs 341).

Although there are two vasopressin genes, there is only a single vasopressin receptor present in the genome.

### Exon duplications

#### Allatostatin CCC

**Figure 9:**
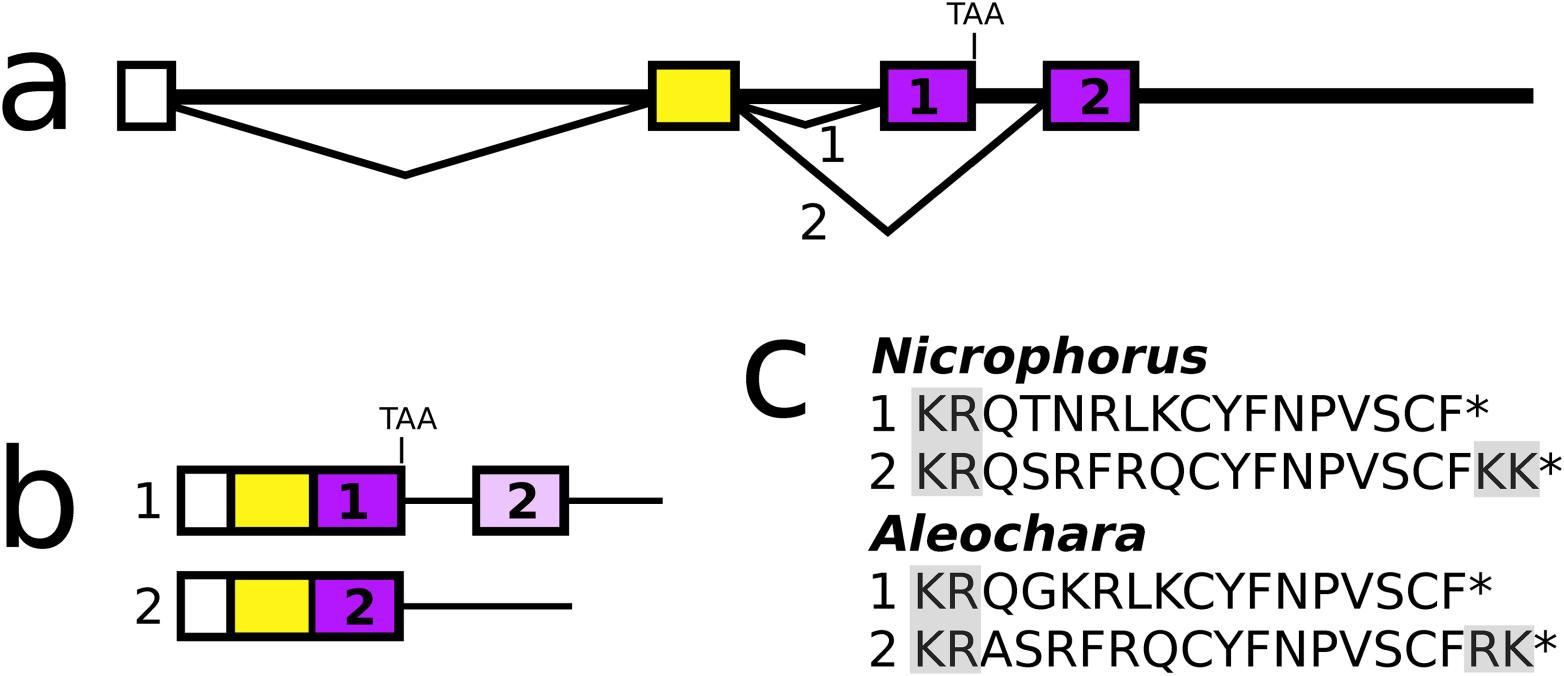
*Nicrophorus* allatostatin CCC gene. (A) Schematic representation of the allatostatin CCC gene in *Nicrophorus*. Boxes indicate exons and horizontal lines introns. The first exon (white) is untranslated, the second (yellow) codes for the signal peptide and few additional amino acid residues. The last coding exon has two acceptor splice sites. (B) When the first acceptor splice site is used the mRNA is larger and leads to the production of an mRNA that contains coding sequences for two allatostatin CCC-like peptides, however, an in-frame stop codon prevents translation of the second one. When the second acceptor splice site is used, it is the second allatostatin CCC peptide that will be produced. (C) The last amino acid residues coded by the two types of mRNA. Convertase cleavage sites and C-terminal dibasic amino acid residues that will be removed by carboxypeptidases are highlighted. The *Aleochara* gene has a very similar structure, although the untranslated first exon could not be identified. Note that the sequences of these peptides are fairly similar between the two species, and that in both cases the peptides produced from the first transcript lack the C-terminal dibasic amino acid residues that are typically present in allatostatin CCC peptides (Veenstra, 2016b).

In both *Aleochara* and *Nicrophorus*, but not in closely related *Oryctes*, the second and last coding exon of the allatostatin CCC gene has been duplicated allowing the production of two different allatostatin CCC transcripts (Fig. 9) predicted to produce slightly different allatostatin CCC peptides that both conform to the consensus sequence of this peptide (Veenstra, 2016b). In the other species only a single allatostatin CCC precursor was found (Fig. S21).

#### Allatotropin

**Figure 10:**
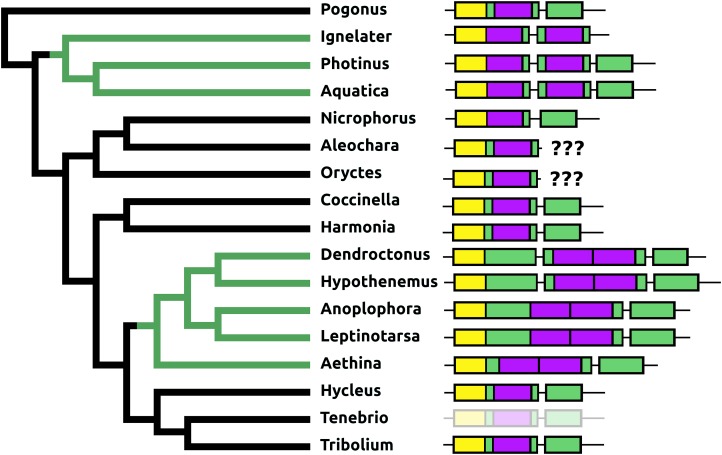
Structure of allatotropin genes. Horizontal lines indicate non-translated DNA and the boxes coding exons. Yellow shows the location of the coding sequences for the signal peptide, purple those for allatotropins and green those for the remainder of the precursors. On two occasions the number of allatotropin paracopies was increased during evolution; green branches in the tree. Once by adding a coding exon, and once by adding a paracopy inside the original allatotropin coding exon. The last coding exons for the *Aleochara* and *Oryctes* allatotropins could not be established. The structure of the *Tenebrio* gene is translucent to indicate that it is only inferred from those of *Hycleus* and *Tribolium* based on the very similar sequences of their respective allatotropin transcripts.

The *Pogonus* allatotropin precursor is almost indistinguishable from the Hemimetabola sequences; it shares with them the N-terminal Gly-Phe-Lys and the remainder of its precursor is also very similar. However, in the other Coleoptera allatotropin sequences these characteristics have not been conserved (Fig. S22). On two occasions, the allatotropin precursor has acquired a second allatotropin paracopy, once by adding a second exon and a second time by adding an additional allatotropin paracopy directly next to the existing one (Fig. 10).

#### Calcitonin

The calcitonin precursor is one of the most variable neuropeptide precursors in Coleoptera (Fig. S23). A functional calcitonin gene is absent from *Photinus*, where a remainder of the gene for the peptide can still be found, but where the putative receptor has completely disappeared, while in four of the other species, *Anoplophora, Hycleus, Tribolium*, and *Tenebrio*, there are two calcitonin genes. The *Leptinotarsa* and one of the *Anoplophora* genes encode not only typical calcitonin peptides, but also a number of structurally very similar peptides that lack the disulfide bridge in the N-terminal portion of the molecule (Fig. 5). The number of paracopies predicted from each precursor varies from one to seven. The sequences of several of these precursors suggests that they have lost one or more calcitonin paracopies during evolution (Fig. S23).

#### DH31

**Figure 11:**
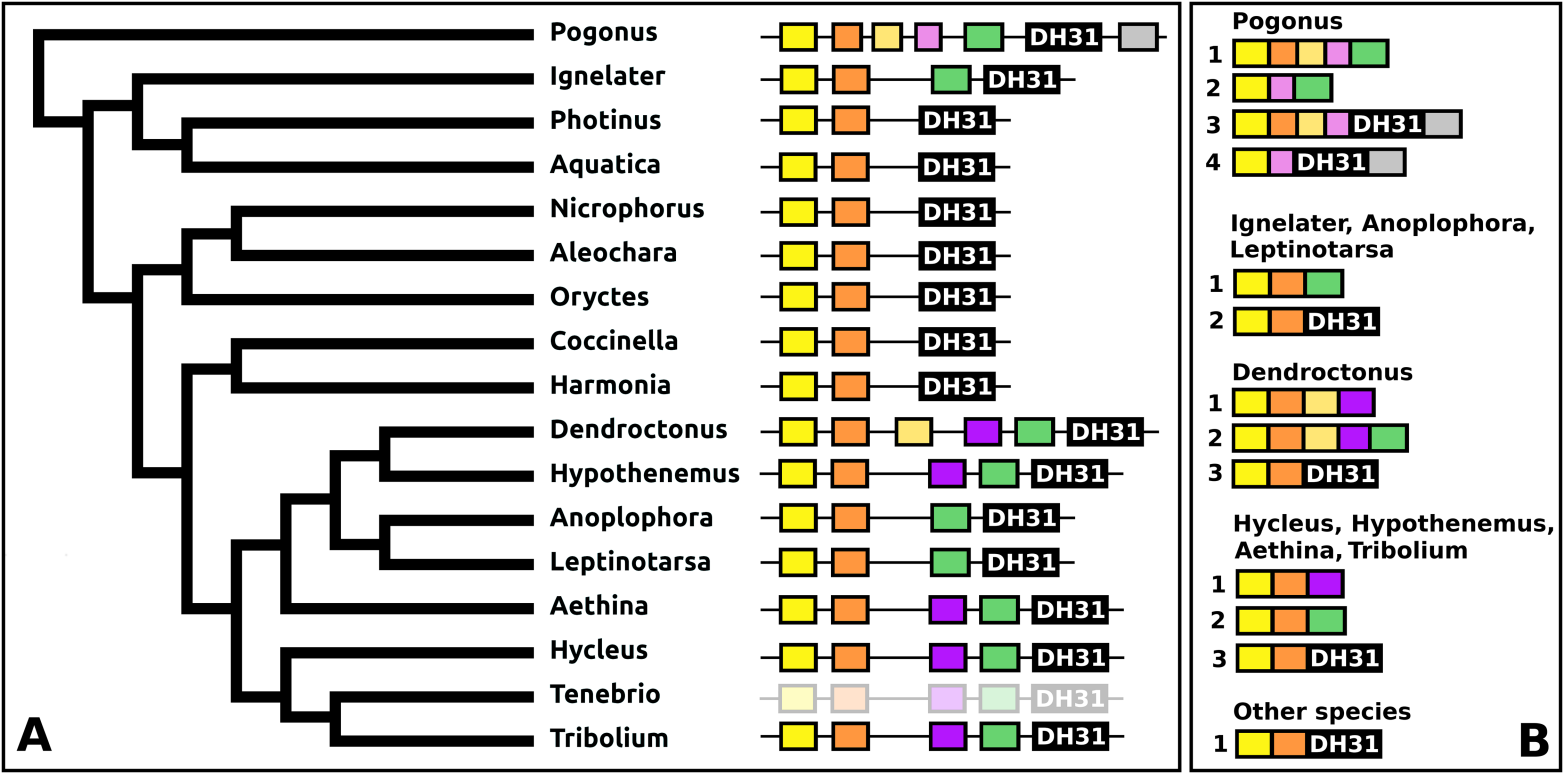
Structure of DH31 genes. (A) DH31 gene structures. (B) D31 transcript structures. Horizontal lines indicate non-translated DNA and the boxes coding exons. Yellow shows the location of the coding sequences for the signal peptide, orange those for a coding exon common to all transcripts, black coding exons for DH31 itself and green and purple those for other putative neuropeptides. The structure of the *Tenebrio* gene is translucent to indicate that it is only inferred from those of *Hycleus* and *Tribolium* based on the very similar sequences of their respective DH31 transcripts. To the left is a phylogenetic tree in order to facilitate comparing sequences with evolution, to the right are the various transcripts that are produced from these genes by alternative splicing.

The DH31 gene shows considerable variation in its structure and the peptides it produces. Although in all species, it codes for the classical DH31 that is very well conserved (Fig. S24), in several species additional neuropeptides are encoded on alternatively spliced mRNAs that do not encode DH31. In its most basic form the gene produces a single transcript from three coding exons containing, respectively, the signal peptide, a conserved peptide that does not look like a neuropeptide, and DH31. In several species, one or two coding exons that code for alternative neuropeptides have been inserted between exons for the conserved peptide and DH31. This leads to alternative splicing in which different neuropeptides are made (Fig. 11). In *Hycleus, Tenebrio*, and *Tribolium* at least three different mRNAs are produced enabling precursors sharing the same N-terminal sequence but that have different C-termini encoding an Arg-amide, a short Pro-amide and the typical DH31 peptide, respectively. In contrast to DH31 itself, that has a very well conserved amino acid sequence, these alternative DH31 gene products lack well defined consensus sequences and are neither very similar to DH31 (Fig. S25).

In *Pogonus* there are two additional exons predicted from the trinity assembly of RNAseq sequences (Fig. 11). In two other Adephaga species, *Gyrinus marinus* and *Carabus granulatus*, the transcriptome assembly sequences corresponding to DH31 sequences lack sequences homologous to those two exons (GAUY02019591.1; GACW01024447.1).

#### DH37–DH47 or CRF-like diuretic hormones

**Figure 12:**
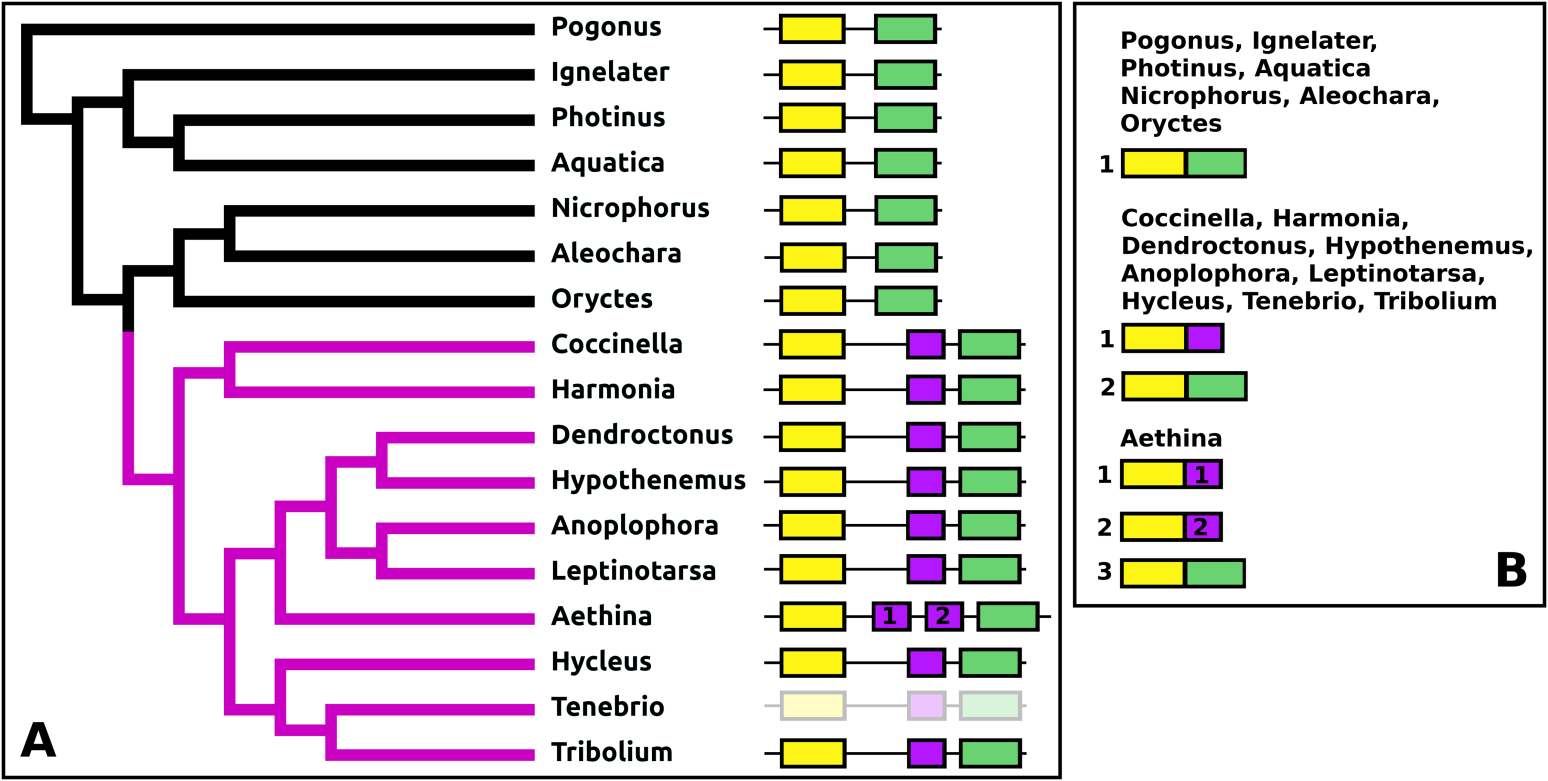
Structure of D37-D47 genes. (A) DH37–DH47 gene structures. (B) DH37–DH4747 transcript structures. Horizontal lines indicate non-translated DNA and the boxes coding exons. Yellow shows the first coding exon containing sequences coding the signal peptide and parts of the precursor, the purple coding exon contains the complete sequence for DH37, its convertase cleavage sites and few additional amino acid residues on each site, and the green coding exon contains the same for DH47. Note that the DH37 exon is only present in the Cucujiformia, corresponding to the magenta part of the tree. The DH37 coding exon has been duplicated in *Aethina* and allows the DH37–DH47 gene to produce three different transcripts and three different putative diuretic hormones. The structure of the *Tenebrio* gene is translucent to indicate that it is only inferred from those of *Hycleus* and *Tribolium* based on the very similar sequences of their respective DH37 and DH47 transcripts. To the right are the various transcripts that are produced from these genes by alternative splicing.

The *Tribolium* DH37–47 gene has previously been reported to have three coding exons (Li et al., 2008), in which the first of those three is alternatively spliced to the second or the third one. This leads to the production of two CRF-like diuretic hormones, DH37 and DH47 which both been isolated and sequenced from *Tenebrio*, a species of the same family. Given the sequence similarity of DH47 (Fig. S26) and DH37 (Fig. S27) it seems likely that the last two exons arose by exon duplication. This gene structure seems to be common to the Cucujiformia, but in the other Polyphaga and *Pogonus* there are only two coding exons in which the last codes for a CRF-like hormone. In *Aethina* the DH37 coding exon has been duplicated once more, such that there are four coding exons in total and three different mRNAs are produced, each encoding different CRF-like peptides (Fig. 12).

#### Periviscerokinin (Capa)

The periviscerokinin genes are quite variable in Coleoptera. They can consist of several coding exons that all use phase 1 introns. This allows alternative splicing to produce a variety of different precursors from these genes. Although in some species RNAseq data confirm such alternative splicing, in many cases the number of total RNAseq reads for these genes is far too small to demonstrate alternative splicing. An important site of periviscerokinin synthesis is in the abdominal ganglia, from which RNAseq reads are generally only obtained when whole insects are used for RNA extraction. Thus while in many species only a single transcript can be documented, alternative splicing may well be common.

**Figure 13:**
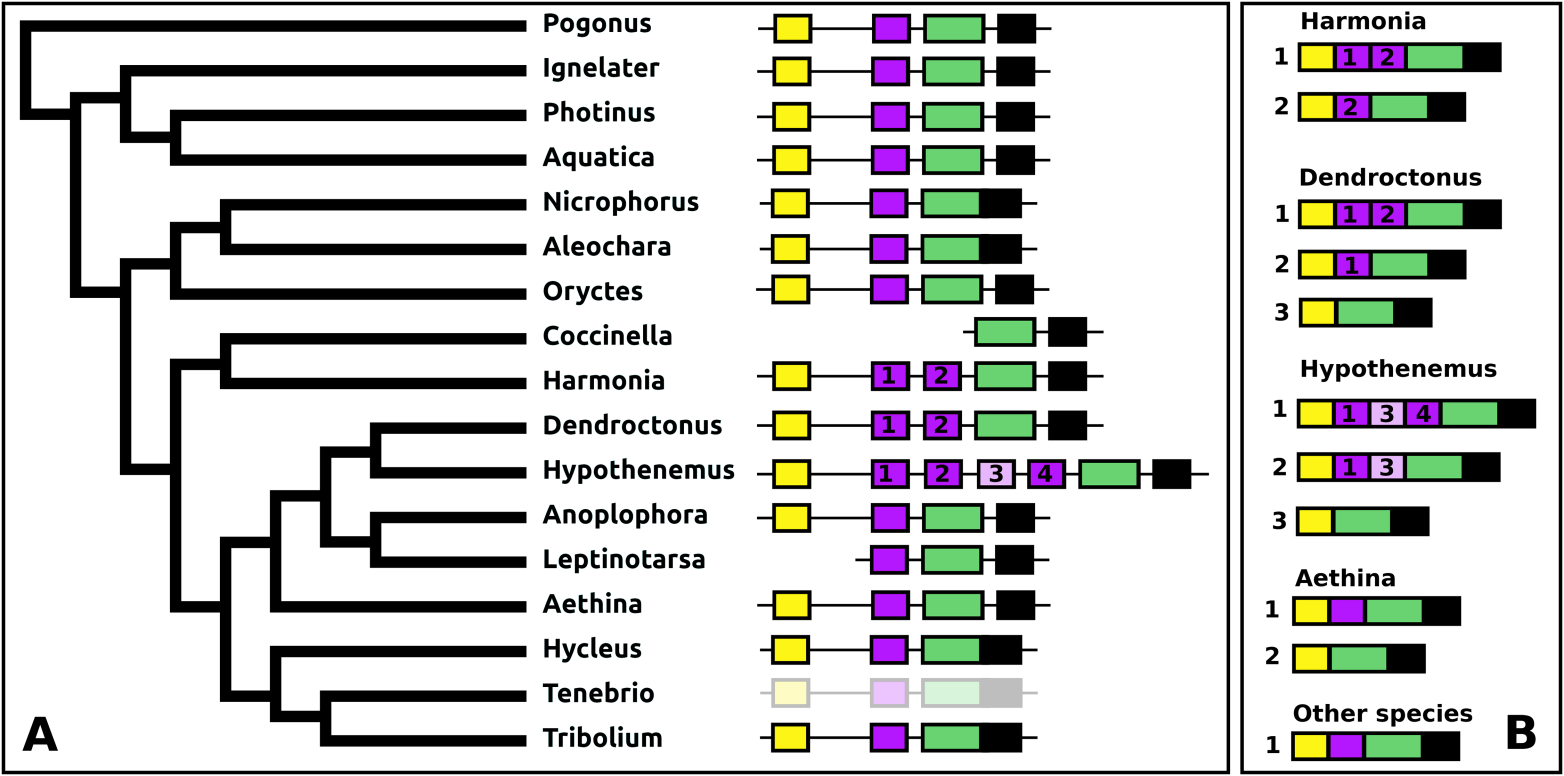
Structure of periviscerokinin genes. (A) Periviscerokinin gene structures. (B) Periviscerokinin transcript structures. Horizontal lines indicate non-translated DNA and the boxes coding exons. Yellow shows the first coding exon containing the sequence coding the signal peptide and parts of the precursor, the purple coding exons contain sequences for a periviscerokinin, and the green coding exon sequences for a periviscerokinin, a tryptopyrokinin and another periviscerokinin. The final exon, black in the figure, characteristically codes for several acidic amino acid residues. In general there are relatively few RNAseq reads for this gene and when there are gaps in the genome assembly, as is the case in *Leptinotarsa* and *Harmonia*, it is not possible to reconstruct the complete gene. The structure of the *Tenebrio* gene is translucent to indicate that it is only inferred from those of *Hycleus* and *Tribolium* based on the very similar sequences of their periviscerokinin transcripts. To the right are the various transcripts that are produced from these genes by alternative splicing. Note that there may well be additional transcripts that could not be identified due to the scarcity of RNAseq reads for this gene.

The number of coding exons for this gene in the species studied varies between four and seven (Fig. 13). The first coding exon contains the sequence for the signal peptide, the last for a hydrophilic C-terminal sequence of the precursor that is usually rich in acidic amino acid residues. The penultimate coding exon tends to be the largest and codes for subsequently a periviscerokinin, a tryptopyrokinin and a hydrophobic sequence. The variable number, one to four exons between the first and pentultimate coding exon, contain sequences for a pyrokinin-like peptide, although in *Hypothenemus*, the third one has lost this sequence.

#### sNPF

**Figure 14:**
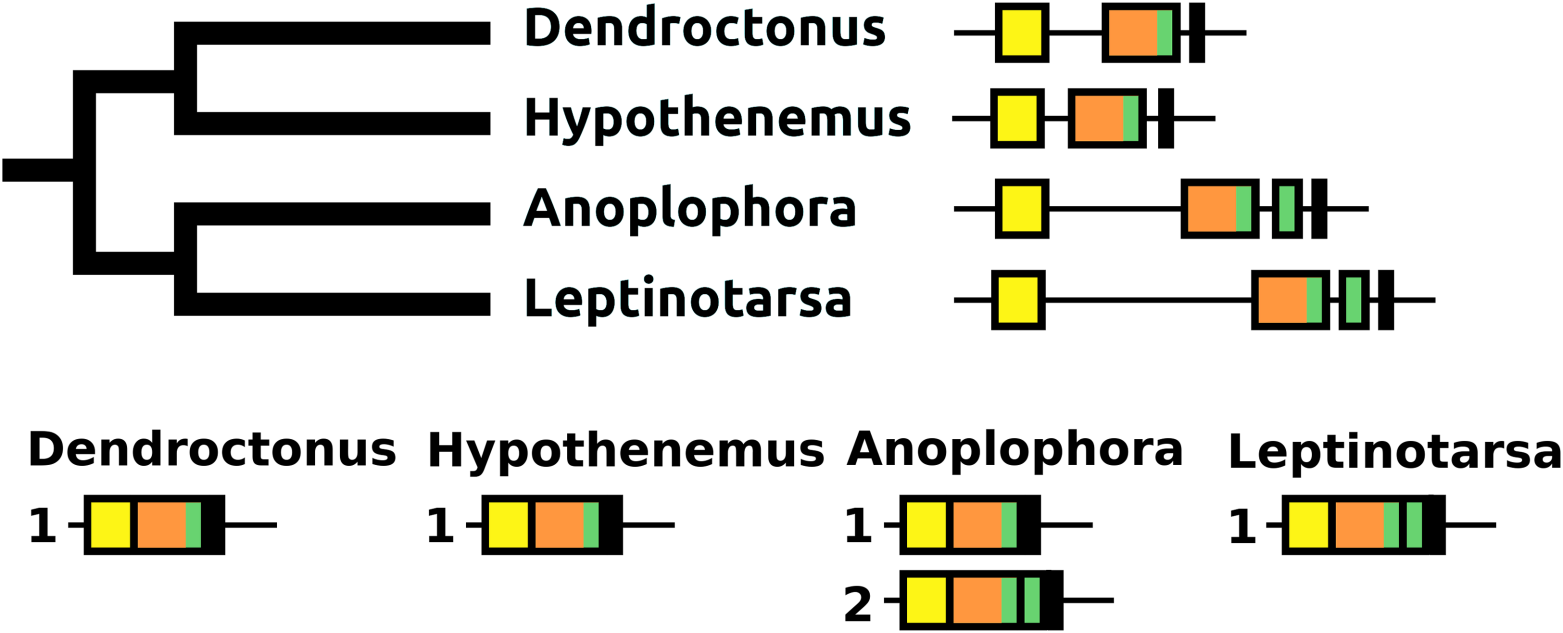
sNPF precursors. Partial tree of four species with the structure of their sNPF genes. In all species except *Anoplophora* and *Leptinotarsa* the sNPF gene consists of three coding exons. The first one (yellow) codes coding for the signal peptide and a few additional amino acid residues, the second one (orange) codes for a well conserved sequence and near the end of the exon has the sequence for sNPF (indicated in green), while the last ones codes for a peptide, that is, not very well conserved. In both *Anoplophora* and *Leptinotarsa* there is an additional exon between the second and third that codes for an additional sNPF paracopy. In *Leptinotarsa*, RNAseq data suggests a single mRNA encompassing all four of these exons in *Leptinotarsa*, but alternative splicing allowing the production of sNPF precursors that have either one or two sNPF paracopies in *Anoplophora*.

The sNPF precursor is very well conserved in Coleoptera (Fig. S9). In *Anoplophora* and *Leptinotarsa*, but not in closely related *Dendroctonus* and *Hypothenemus* or any of the other species studied here, partial duplication of the exon coding sNPF led to a gene having an additional coding exon. In *Anoplophora* the RNAseq data suggest the production of two alternatively spliced transcripts that code for either one or two sNPF paracopies. Although there is much more RNAseq data from *Leptinotarsa*, in this species there is only evidence for a single mRNA encoding two sNFP paracopies (Fig. 14).

### Paracopy numbers

Several insect neuropeptide precursors contain multiple copies of identical or very similar peptides. These typically include allatostatins A and B, calcitonin B, leucokinin, FMRFamide, pyrokinin, periviscerokinin, ETH, orcokinin A and B, RYamide, sulfakinin, and tachykinins. The number of such paracopies can vary between and even within species (Veenstra, 2010). The genes coding calcitonin B, leucokinin, pyrokinin, and periviscerokinin have already been discussed above. Allatostatin A has so far never been found in Coleoptera. ETH has usually two paracopies, but in the three species from the Elateroidea—*Ignelater*, *Photinus*, and *Aquatica*—the first copy has been lost (Fig. S28) and this is also the case in *Aleochara* and *Hypothenemus*. In all five of these species, the genome still contains coding sequences for both splice variants of the ETH receptor. The RYamide gene codes for two RYamide peptides in all Coleoptera (Fig. S29), except *Anoplophora* that lost this gene and its receptor, while the sulfakinin gene codes also for two paracopies in all species studied here (Fig. S30). The number of FMRFamide paracopies varies from four to six (Fig. S31), and from 5 to 9 for the NPLP1 precursor (Fig. S32) and for allatostatin B (Fig. S33) the numbers are from seven for the Curcuclionids *Dendroctonus* and *Hypothenemus* to eight for the other species.

#### Tachykinin

The calcitonin B and tachykinin genes are those that show significant changes in the number of neuropeptides encoded. The ancestral tachykinin gene in Coleoptera likely coded for eight paracopies, the number found in the majority of species. *Oryctes* and *Harmonia* each lost one paracopy, but in *Anoplophora* there are only five paracopies and in *Leptinotarsa* there are just two left. In the latter species, the well conserved N-terminus of the precursor has also disappeared. This may well be a general phenomenon in Chrysomelidae as the transcriptome from *Oreina cacaliae* (GDPL01001642.1) reveals a very similar sequence (Fig. S34). *Leptinotarsa* does have an ortholog of the tachykinin receptor gene that looks normal.

### Genes that seem very stable

It is fair to state that the number of changes in neuropeptide genes in Coleoptera is significant. This might obscure the fact that many other genes seem, as least as far as their sequences are concerned, remarkably stable, such is the case for CCAP (Fig. S35), SIFamide (Fig. S36), Sulfakinin (Fig. S30), GPA2 (Fig. S37), GPB5 (Fig. S38), FMRFamide (Fig. S31), hansolin (Fig. S39; Liessem et al., 2018), CNMamide (Fig. S40), ITG (Fig. S41), and PTTH (Fig. S42). The mRNA from the gene coding ion transport peptide (ITP) is generally alternatively spliced in two forms, ITP-A (Fig. S43) and ITP-B (Fig. S44). It has been reported that in *Tribolium* there is a third splice product (Begum et al., 2009), but such a form could not be detected for any of the other species studied here, including *Hycleus* or *Tenebrio*, two species closely related to *Tenebrio*.

## Discussion

A previous study on neuropeptides in twelve *Drosophila* species found their neuropeptidomes to be remarkably similar (Wegener & Gorbashov, 2008). This is the first time that the neuropeptidomes of several species of the same insect order that are not as closely related have been compared. The results clearly show considerable variation within Coleoptera, variation that seems to be almost as large as that seen between species from different orders. By using a variety of species some surprising findings, such as, for example, the very evolved structures of NPF and PDF or the loss of certain neuropeptide genes, are confirmed in related species, and they can thus not be attributed to experimental error.

In the same way that there are differences between the different neuropeptides, there are also differences between the different species. *Leptinotarsa* is perhaps the species in which the neuropeptidome has evolved the most. It has two vasopressin genes, its allatotropin and sNPF genes encode two paracopies each, it lost both elevenin and ACP and it duplicated the CCHamide 1 receptor and CCHamide 2 neuropeptide genes. *Anoplophora* is another member of the Chrysomeloidea superfamily with a neuropeptidome with significant changes. Although *Anoplophora* still has elevenin and ACP, it lost RYamide and it has a large number of insulin genes. Both these species are specialist herbivores, like many Cucujiformia. Always eating the same or almost the same food might eliminate some physiological uncertainties that no longer need to be regulated. Variation in protein, carbohydrate and water content in food should be much more limited in specialists than in generalists. For example, if RYamide is indeed an antidiuretic hormone as suggested (Veenstra & Khammassi, 2017) it might become obsolete in a species that is always exposed to the necessity to conserve water. It will no longer be necessary to increase water conservation during times of water shortage and decrease it when the insect is fully hydrated and thus there may be no longer a need for the acute regulation of antidiuresis; it always has to be optimal.

### Significant peptide sequence changes

In those Coleoptera where elevenin and ACP are (still) present neither the sequences of the peptides nor of those of their precursors are well conserved. Other neuropeptides have not only maintained the sequences of the neuropeptides themselves, but often those of the entire precursors, suggesting that those parts of the precursor that do not code for the biologically active peptides must have other important functions. It has previously been noted that as expression of the RYamide gene in *Drosophila* species decreases, the structure of both the neuropeptides themselves and their precursors are no longer well conserved (Veenstra & Khammassi, 2017). This could mean that the function of the peptide is becoming obsolete, which would facilitate its subsequent loss; use it or loose it. However, it is also possible that it is no longer needed in the large quantities that are necessary for discharge into the hemolymph. This might well be the case for *Drosophila* RYamide where the rectal papillae in *Drosophila melanogaster* are innervated by RYamide neurons. While the same neurons are present in *Drosophila virilis*, in that species—and many others—RYamide is also released from enteroendocrine cells, presumably likewise to stimulate the rectal papillae. The amount of RYamide that needs to be released from the midgut to reach sufficiently high hemolymph concentrations will be much larger than that made by the neurons that directly innervate the rectal papillae. This likely not only puts selection pressure on the peptide sequences but also on an efficient processing of their precursors. It is the latter that may explain why some insect neuropeptide precursors seem to be so well conserved. In *Rhodnius* and *Tribolium* ACP appears to be expressed exclusively in neurons (Hansen et al., 2010; Patel et al., 2013). This suggests that its large structural variability indicates a loss of physiological relevance in Coleoptera which may explain its loss from the genome on at least three occasions in this insect order. The same could also be true also for elevenin and it is tempting to speculate that when neuropeptide structures are no longer well conserved it either indicates the loss of their physiological relevance as a hormone or as both a hormone and a neuromodulator. For small neuropeptides that act on GPCRs replacement of a single critical amino acid may be enough to impair receptor binding. Insulin is larger and needs to bind simultaneously to two tyrosine kinases it may therefore be less sensitive to single amino residue replacements as long as the three-dimensional structure of the molecule is maintained and interaction with its receptor is still possible.

It is interesting to see that some changes in Coleoptera neuropeptide precursors are similar to the those observed in other Holometabola. The allatotropin genes in Hemimetabola code for a single allatotropin paracopy, but in Lepidoptera the gene encodes various allatotropin-like peptides produced on alternatively spliced mRNAs (Taylor, Bhatt & Horodyski, 1996; Nagata et al., 2012). Whereas, the *Pogonus* allatotropin gene is quite similar to those of the Hemimetabola, in the Polyphaga suborder allatotropin genes coding for two paracopies emerged on two occasions. The sNPF gene in Hemimetabola is also very simple, but in Lepidoptera and Diptera, the gene codes for several paracopies. Again this evolved independently in *Anoplophora* and *Leptinotarsa*. If proctolin is indeed absent from *Oryctes* and the Coccinellids, this would be similar to what occurred in Hymenoptera and Lepidoptera. Thus in at least some cases neuropeptide evolution in different holometabolous insect orders seems to follow what look to be similar pathways, that is, increasing paracopies in neuropeptide genes that look like they never changed from the ancestral arthropod to cockroaches, decapods and chelicerates, or independently eliminating others, such as elevenin and ACP. This raises the question whether somehow complete metamorphosis is responsible for these changes.

Several neuropeptides contain a cysteine bridge structure constraining the structure of the peptide. This could have important effects on receptor binding and/or provide it protection against proteases degradation. It is surprising to see NPF gain a cysteine bridge in the Cucujiformia and some, but not all, calcitonins in *Leptinotarsa* and *Anoplophora* loose theirs. It would be very interesting to see the interactions of these peptides with their receptors in order to know what effects, if any, these structures have on receptor activation.

### Gene losses

It appears that loss of a neuropeptide systems is not a very rare event and some neuropeptides are more easily eliminated than others. Thus, in Coleoptera some neuropeptides got lost repeatedly, that is elevenin and ACP each at least three times, natalisin twice and the dilp8 ortholog likely four times. Indeed, elevenin, ACP, calcitonin, corazonin, natalisin, dilp8, and relaxin were also found missing in other insect species (Huybrechts et al., 2010; Veenstra, 2014, 2019).

Although the loss of a neuropeptide signaling system may well have its origin in the degeneration of the peptide gene, for the reasons of gene sizes, it is as likely to start in the receptor gene. Not only are the receptor coding regions generally much larger than those of neuropeptides, but more often than not the total size of these genes is enormous. Thus the accidental elimination of a large piece of DNA after chromosome breakage and rearrangement may well be limited to sequences coding a receptor without altering any adjacent genes, while the elimination of a piece of the same size that touches a neuropeptide gene is more likely to affect also neighboring genes, thus increasing the likelihood of selection against such an event. Indeed in at least some instances, one can still find remnants of the ligand gene, while the receptor has vanished. Apart from calcitonin B in *Photinus* described here, the same phenomenon is observed with relaxin in *Sitophilus* and sulfakinin in the tsetse fly *Glossina morsitans*. In the latter species a highly degraded sulfakinin pseudogene is still recognizable, while both sulfakinin receptor genes have been lost (BLAST Data).

As discussed above if the sequence of a neuropeptide is no longer maintained it may indicate the loss of physiological relevance. This might be a useful criterium to look at duplicated neuropeptide genes as well. When the putative Coleopteran AKH precursors are compared it is evident that not all these precursors are well conserved. If the bulk of AKH precursor sequences is so well conserved, why are the others not? We have a good idea about what signal peptides and authentic AKHs should look like, and we can thus discard the truly aberrant genes from *Aethina* and *Harmonia* as obviously no longer functional AKH genes (Fig. 7). However, we do not know what the requirements are for a good Cys-peptide, as its function is unknown. Similar Cys-peptides have been found in other insect neuropeptide precursors, such as those of SIFamide and RYamide (Verleyen, Huybrechts & Schoofs, 2009; Veenstra & Khammassi, 2017). The conservation of the structure of such peptides implies that they are important—perhaps for assuring correct intracellular transport to the secretory granules of the neuropeptide precursors—and thus that those precursors that no longer have such a conserved Cys-peptide may be functionally impaired. Predicted AKH precursors that look like they might be defective are only found in species that also have an AKH gene with a well conserved AKH precursor. This suggests that AKH precursors that are not well conserved may have largely lost their functional significance and/or may be evolving into pseudogenes. It is interesting in this context that of the two *Tribolium* AKHs only the one that has the best conserved precursor sequence could be detected by mass spectrometry (Li et al., 2008). Similar arguments suggests that the copy of the *Oryctes* Bursicon A gene may well be on its way to become a pseudogene.

The predicted signal peptides of the proctolin precursors from *Oryctes, Harmonia*, and *Coccinella* seem to be perfectly normal as do other parts of the precursor that have been conserved since the last common ancestor of chelicerates and mandibulates. However, the predicted proctolin molecules have been mutated and these species all seem to have lost their proctolin receptors. It is a very puzzling and unresolved matter; as if proctolin is not the only biologically active peptide produced from the proctolin precursor or as if there is yet another proctolin receptor that remains to be identified.

### Gene duplications

Gene duplications are a common phenomenon during evolution and most of these duplicated genes are subsequently lost (Lynch, 2007). Genes coding insulin-related peptides and adipokinetic hormones are repeatedly amplified in insects and in Coleoptera this also includes pyrokinin genes. Why is it that some neuropeptide genes regularly show increased numbers while others do so only rarely? When there are paralogous genes in a genome, this is the result of two independent processes, first duplication of the original gene and then maintaining both the original and its copy.

Just like elimination of receptor gene is likely facilitated by its large size, the initial duplication of a neuropeptide gene is probably more easily accomplished due to the smaller the sizes of the gene. It is striking in this context that both the insect AKH and insulin genes—two that are commonly amplified in insects—are generally very compact genes, have small introns and plausibly small regulatory regions (of that we only have some information from *Drosophila*, which is not necessarily a model for all insect species). The Coleoptera pyrokinin genes similarly look very compact, as the sizes of the introns between the coding regions are small. This is also true for the strongly amplified vasopressin genes in *Locusta* (Veenstra, 2014).

Such small sizes may not only favor the original duplication, but also make it much more difficult to eliminate the gene by gross chromosome reorganizations and this may explain the presence of amplified genes in a genome that are not as well conserved as others. However, in order to permanently maintain two paralog genes, there also needs to be an advantage to maintaining both copies. This is often achieved through neo- or subspecialization (Lynch, 2007). In *Drosophila* the different insulin genes do not all have the same temporal and spatial expression (Brogiolo et al., 2001; Liu et al., 2016), suggesting that subspecialization may be at least part of the reason these genes are maintained. I have previously suggested that in some cases it may be the need for massive amounts of neuropeptides that facilitates the maintenance of paralog neuropeptide genes (Veenstra, 2014). In the case of the Coleoptera pyrokinin genes this may well apply also, but there is maybe something else at play as well.

Pyrokinins, tryptopyrokinins, and periviscerokinins are structurally similar arthropod peptides that each act on specific receptors (Iversen et al., 2002; Rosenkilde et al., 2003; Cazzamali et al., 2005; Homma et al., 2006; Paluzzi et al., 2010; Paluzzi & O’Donnell, 2012; Jiang et al., 2014). The tryptopyrokinins are only present in insects, where they seem to play an important physiological role and they are absent from basal arthropods. In most insect species they are coded by two different genes, the pyrokinin and periviscerokinin genes. The pyrokinin genes codes for pyrokinins, often also for a tryptopyrokinin and rarely even a periviscerokinin. The periviscerokinin gene codes for periviscerokinins, often a tryptopyrokinin and rarely a pyrokinin. Whatever their exact roles or physiological functions, there appears to be a physiological need to be able to produce these three types of peptides, pyrokinins, tryptopyrokinins, and periviscerokinins independently from one another. In termites, crickets, stick insects, locusts, and cockroaches separate tryptopyrokinin genes have evolved that code for a tryptopyrokinin precursors containing multiple tryptopyrokinins. However, this has not happened in holometabolous insects.

The tryptopyrokinins are produced predominantly, if not exclusively, by neuroendocrine cells in the labial neuromere of the suboesophageal ganglion from either a pyrokinin or periviscerokinin precursor by mechanisms that are not understood. Although receptor ligand interactions in *Tribolium* suggests that at least in that species there may be no tryptopyrokinin specific receptor (Jiang et al., 2014), in the closely related *Zoophobas atratus* the periviscerokinin precursor is still differentially processed to produce predominantly a tryptopyrokinin and pyrokinin in the suboesophageal ganglion and periviscerokinins in the abdominal ganglia (Neupert et al., 2018). Interestingly, in *Tribolium* the tryptopyrokinin from the pyrokinin precursor is less active on the pyrokinin receptors than the one from the periviscerokinin precursor (Jiang et al., 2014) and so it may be no coincidence that the tryptopyrokinin from was lost from a number of Coleoptera pyrokinin genes.

Whereas the pyrokinin genes are often amplified in Coleoptera, the periviscerokinin genes are often sometimes partially amplified, that is, a periviscerokinin coding exon is duplicated. Adding an extra exon does not change the reading frame, since the introns defining the duplicated exons are of the same phase. This makes alternative splicing relatively easy. Duplication of this exon may be further facilitated by the presence of much larger introns than those that are found in the pyrokinin gene.

The size of receptor genes should make their segmental duplication a relatively rare event. It is thus interesting that in *Leptinotarsa* the CCHamide 1 receptor is duplicated and that both copies seem to be well expressed. Surprising and unexpected is that in the same species the CCHamide 2 neuropeptide gene is also duplicated. Although this does not constitute final proof for the evolution of a novel insect neuropeptide system in *Leptinotarsa*, it certainly is as close as one can get from sequence data alone. Both receptor and peptide have evolved significantly since their respective duplications (Fig. 8) and this very much suggests that *Leptinotarsa* has three separate CCHamide neuropeptide systems.

## Conclusion

Beetle species show very significant differences in their neuropeptidomes. Thus neuropeptidome variation may be (almost) as big within insect orders as it is between them.

## Supplemental Information

10.7717/peerj.7144/supp-1 Supplemental Information 1 Raw sequence data used.

10.7717/peerj.7144/supp-2 Supplemental Information 2 Deduced amino acid sequences of Coleoptera neuropeptides.

10.7717/peerj.7144/supp-3 Supplemental Information 3 Supplementary Figures for Coleoptera Neuropeptidomes.

## Additional Information and Declarations

### Data Availability

The following information was supplied regarding data availability: Genome assemblies were downloaded from NCBI (https://www.ncbi.nlm.nih.gov/genome) using the following direct links: (https://www.ncbi.nlm.nih.gov/genome/?term=Tribolium; https://www.ncbi.nlm.nih.gov/genome/?term=Pogonus; https://www.ncbi.nlm.nih.gov/genome/?term=Nicrophorus; https://www.ncbi.nlm.nih.gov/genome/?term=Aleochara; https://www.ncbi.nlm.nih.gov/genome/?term=Oryctes; https://www.ncbi.nlm.nih.gov/genome/?term=Coccinella; https://www.ncbi.nlm.nih.gov/genome/?term=Harmonia; https://www.ncbi.nlm.nih.gov/genome/?term=Dendroctonus; https://www.ncbi.nlm.nih.gov/genome/?term=Hypothenemus; https://www.ncbi.nlm.nih.gov/genome/?term=Anoplophora; https://www.ncbi.nlm.nih.gov/genome/?term=Leptinotarsa; https://www.ncbi.nlm.nih.gov/genome/?term=Aethina), with the exception of the genomes of *Hycleus cichorii* and *Hycleus phaleratus*, which were obtained from the GigaScience repository (http://gigadb.org/dataset/100405) and those of *Photinus pyralis*, *Aquatica lateralis*, and *Ignelater luminosus*, which were downloaded from http://fireflybase.org/firefly_data.html. When available, protein sequences predicted from the various genomes were also downloaded from NCBI or the two websites mentioned using the same direct links indicated above. Predicted proteins for *Hypothenemus hampei* were obtained from https://genome.med.nyu.edu/coffee-beetle/cbb.html, and those for Aleochara the Animal Ecology department of the Free University of Amsterdam (http://parasitoids.labs.vu.nl/parasitoids/aleochara/data.php).
