# Supplementary material for: Coleoptera genome and transcriptome sequences reveal numerous differences in neuropeptide signaling between species
Source: PeerJ. 2019 Jun 17;7:e7144. doi: 10.7717/peerj.7144 (PMC6585902; doi:10.7717/peerj.7144)
Supplement: Supplemental Information 1 [file peerj-07-7144-s001.pdf]

Supplementary Table 1.
Listing of short read archives used

Jan A. Veenstra

Coleoptera Genome and Transcriptome Sequences reveal numerous Differences in Neuropeptide Signaling between Species

Manuscript submitted to PeerJ

Transcriptome files
Aethina: SRR1798556.
Aleochara curtala: SRR921563.
Anoplophora: SRR5867415, SRR5867416, SRR5867417, SRR5867418, SRR5867419, SRR5867420, SRR5867421, SRR5867422, SRR5867423, SRR5867424, SRR1986051, SRR1986052, SRR1986053, SRR1986054, SRR1986055, SRR1986056, SRR1986057, SRR1986058, SRR1799851, SRR1799852.
Aquatica: DRR119264, DRR119265, DRR119266, DRR119267, DRR119268, DRR119269, DRR119270, DRR119271, DRR119272, DRR119273, DRR119274, DRR119275, DRR119276, DRR119277, DRR119278, DRR119279, DRR119280, DRR119281, DRR119282, DRR119283, DRR119284, DRR119285, DRR119286, DRR119287, DRR119288, DRR119289, DRR119290, DRR119291, DRR119292, DRR119293, DRR119294, DRR119295.
Coccinella: ERR1145724, ERR1145727, SRR2971089, SRR2971092, SRR2971095, ERR1145725, ERR1145728, SRR2971090, SRR2971093, SRR2971096, ERR1145726, ERR1145729, SRR2971091, SRR2971094, SRR2971097
Dendroctonus: SRR3323584, SRR3340437, SRR3340441, SRR3342470, SRR3342473, SRR6279151, SRR6279154, SRR6279157, SRR6279160, SRR6279173, SRR3340435, SRR3340439, SRR3342468, SRR3342471, SRR3342474, SRR6279152, SRR6279155, SRR6279158, SRR6279171, SRR3340436, SRR3340440, SRR3342469, SRR3342472, SRR3342475, SRR6279153, SRR6279156, SRR6279159, SRR6279172, SRR867441, SRR867440, SRR867439, SRR867438, SRR867436, SRR867434, SRR867433, SRR867432, SRR867188, SRR867186, SRR867183, SRR867179, SRR867176, SRR867161, SRR867162, SRR867160, SRR2044910, SRR2044909, SRR2044908, SRR2044907, SRR2044906, SRR2044905, SRR2044904, SRR2044903, SRR2044902, SRR2044901, SRR2044900, SRR2044899, SRR2044898, SRR2044897, SRR2044896, SRR2044895, SRR1703019, SRR1703018, SRR1703016, SRR1703014, SRR1703012, SRR1703010, SRR1703009, SRR1702992, SRR1702988, SRR1702987, SRR1702979, SRR1702966, SRR1702950, SRR1702934, SRR1702933, SRR1702932, SRR1702930, SRR1702929, SRR1702927, SRR1702925, SRR1702923, SRR1702919, SRR1702916, SRR1702913, SRR1702910, SRR1702904, SRR1702901, SRR1702898, SRR1702894, SRR1702891, SRR1702890, SRR1702878.
Harmonia: ERR1309558, ERR1309559.
Hycleus: SRR5408725, SRR5757329, SRR5408726, SRR5757330.
Ignelater: SRR6339830, SRR6339831, SRR6339832, SRR6339833, SRR6339834, SRR6339835, SRR6339838.
Leptinotarsa: SRR5024423, SRR5024424, SRR5024429, SRR5024430, SRR5024431, SRR1820736, SRR5024432, SRR1820750, SRR5024433, SRR1820763, SRR5024435, SRR1820770, SRR5024436, SRR1820776, SRR5024437, SRR1820785, SRR5024438, SRR1820807, SRR5024439, SRR1820827, SRR5024440, SRR1820839, SRR5024441, SRR1820843, SRR6238873, SRR1820867, SRR6238874, SRR1820877, SRR6238875, SRR1827565, SRR6238876, SRR1827566, SRR6238877, SRR1948057, SRR6238878, SRR1948059, SRR6238879, SRR2556962, SRR6238880, SRR2600374, SRR6238881, SRR2600376, SRR6238882, SRR2600438, SRR6238883, SRR2600681, SRR6238884, SRR2600974,

SRR6238885, SRR2600998, SRR6238886, SRR2600999, SRR6238887, SRR2601000, SRR6238888, SRR3999901, SRR6238889, SRR3999902, SRR6238890, SRR3999903, SRR7127649, SRR4069274, SRR7127650, SRR4069275, SRR7127651, SRR4069276, SRR7127652, SRR5024410, SRR7127653, SRR5024411, SRR7127654, SRR5024412, SRR7127655, SRR5024413, SRR7127656, SRR5024414, SRR7127657, SRR5024415, SRR7127658, SRR5024416, SRR7127659, SRR5024417, SRR7127660, SRR5024418, SRR7127661, SRR5024420, SRR7127662, SRR5024421, SRR7127663, SRR5024422, SRR7127664
Nicrophorus: SRR5307970, SRR5307971, SRR5307972, SRR5307973, SRR5307974, SRR5307975, SRR5307976, SRR5307977, SRR5307978, SRR5307979, SRR5307980, SRR5307981, SRR5307982, SRR5307983, SRR5307984, SRR5307985, SRR5307986, SRR5307987, SRR5307988, SRR5307989, SRR5307990, SRR5307991, SRR5307992, SRR5307993, SRR5307994, SRR5307995, SRR5307996, SRR5307997, SRR5307998, SRR5307999, SRR5308000, SRR5308001, SRR5308002, SRR5308003, SRR5308004, SRR5308005, SRR5308006, SRR5308007, SRR5308008, SRR5308009.
Oryctes: SRR2970555.
Photinus: SRR3883756, SRR3883757, SRR3883758, SRR3883759, SRR3883760, SRR3883761, SRR3883762, SRR3883763, SRR3883764, SRR3883765, SRR3883766, SRR3883767, SRR3883768, SRR3883769, SRR3883770, SRR3883771, SRR3883772, SRR3883773, SRR6345454, SRR6345453, SRR6345452, SRR6345449, SRR6345447, SRR6345446, SRR6345445
Pogonus: SRR424340, SRR424342, SRR424344.
Tenebrio: SRR1636025, DRR002380, SRR1291244, SRR1023012, SRR1023013, SRR1023014, SRR1023015, SRR1023016, SRR1023017, SRR1023018, SRR1023019, SRR1023020, SRR1023021, SRR1023022, SRR1023023.

SRR2044898 : This SRA is contaminated, it contains in addition to *Dendroctonus ponderosae* sequences also those from another beetle.
SRR1827565 : Although advertised as being from *Leptinotarsa decemlineata*, this SRA appears to contain sequences from *Eurytemora affinis*, a copepod.

Genome files
Aethina: None.
Aleochara: abili_mapping.bam from http://parasitoids.labs.vu.nl/parasitoids/aleochara/data.php
Anoplophora: SRR941724; SRR941725; SRR941727.
Aquatica: DRR119296; DRR119297; DRR119298.
Coccinella: DRR140188.
Dendroctonus: SRR6279141; SRR6279142; SRR6279143; SRR6279144; SRR6279145; SRR6279146.
Harmonia: DRR140185.
Hycleus: SRR5710157.
Hypothenemus: SRR1986438; SRR1986395.
Ignelater: SRR6339837; SRR6339836; SRR6760567.
Leptinotarsa: SRR1055545; SRR1055546; SRR1055547; SRR1055548; SRR1055549.
Nicrophorus: None.
Oryctes: SRR2968118; SRR2967080; SRR2967097; SRR2968866; SRR2968867; SRR2968913; SRR2968914.
Photinus: SRR6345450.
Pogonus: SRR5427964; SRR5427963; SRR5427962; SRR5427961; SRR5427960; SRR5427959.
Tenebrio: None.
Tribolium: None.
