# Supplementary material for: Coleoptera genome and transcriptome sequences reveal numerous differences in neuropeptide signaling between species
Source: PeerJ. 2019 Jun 17;7:e7144. doi: 10.7717/peerj.7144 (PMC6585902; doi:10.7717/peerj.7144)
Supplement: Supplemental Information 3 [file peerj-07-7144-s003.pdf]

Supplementary Data for:

Coleoptera Genome and Transcriptome Sequences reveal numerous Differences in Neuropeptide Signaling between Species

Jan A. Veenstra

PeerJ – 2019

Note: In the supplementary figures depicting neuropeptide precursor alignments, putative neuropeptides in those precursors have been underlined in red.

Supplementary figures

Figure S1.	Example of sequencing reads demonstrating GPCR absence.	Page 3
Figure S2.	Alignment of baratin precursors.	Pages 4-5
Figure S3.	Alignment of myosuppressin precursors.	Page 6
Figure S4.	Alignment of orcokinin-B precursors for convertase cleavage sites.	Page 7
Figure S5	Alignment of Coleoptera Periviscerokinin precursors.	Pages 8-9
Figure S6.	Alignment of Coleoptera leucokinin precursors.	Page 10
Figure S7.	Alignment of LGR3s.	Page 11
Figure S8.	Alignment of eclosion hormone precursors.	Page 12
Figure S9.	Alignment of sNPF precursors.	Page 13
Figure S10.	Alignment of proctolin precursors.	Page 14
Figure S11.	Alignment of RFLamide precursors.	Page 15
Figure S12.	Alignment of bursicon-A precursors.	Page 16
Figure S13.	Alignment of bursicon-B precursors.	Page 17
Figure S14.	Alignment of CCHamide-2 precursors.	Page 18
Figure S15.	Phylogenetic tree insulins Hycleus, Tenebrio and Tribolium.	Page 19
Figure S16.	Phylogenetic tree insulins selected Cucujiformia.	Page 20
Figure S17.	Alignment of neuroparsin precursors.	Page 21
Figure S18.	Alignment of pyrokinin precursors.	Pages 22-23
Figure S19.	Alignment of relaxin precursors.	Page 24
Figure S20.	Alignment of vasopressin precursors.	Page 25
Figure S21.	Alignment of allatostatin CCC precursors.	Page 26
Figure S22.	Alignment of allatotropin precursors.	

Figure S23.	Calcitonin-B precursors.	Pages 28-29
Figure S24.	Alignment of DH31 precursors.	Page 30
Figure S25.	Alignment of peptides encoded by DH31 genes.	Page 31
Figure S26.	Alignment of DH47 precursors.	Page 32
Figure S27.	Alignment of DH37 precursors.	Page 33
Figure S28.	Alignment of ETH precursors.	Page 34
Figure S29.	Alignment of RYamide precursors.	Page 35
Figure S30.	Alignment of FMRFamide precursors.	Page 36
Figure S31.	Alignment of sulfakinin precursors.	Pages 37-38
Figure S32.	Alignment of NPLP1 precursors.	Pages 39-41
Figure S33.	Alignment of allatostatin-B precursors.	Page 42
Figure S34.	Alignment of tachykinin precursors.	Pages 43-44
Figure S35.	Alignment of CCAP precursors.	Page 45
Figure S36.	Alignment of SIFamide precursors.	Page 46
Figure S37.	Alignment of GPA2 precursors.	Page 47
Figure S38.	Alignment of GPB5 precursors.	Page 48
Figure S39.	Alignment of Hansolin precursors.	Page 49
Figure S40.	Alignment of CNMamide precursors.	Pages 50-51
Figure S41.	Alignment of ITG-like precursors.	Page 52
Figure S42.	Alignment of PTTH precursors.	Page 53
Figure S43.	Alignment of ITP-A precursors.	Page 54
Figure S44.	Alignment of ITP-B precursors.	Page 55

Hypothenemus hampei

```
Tribolium Natalisin-R     MSTTESWKEEEGNGS---FQGFDYGLLVPISSQTPVVVDSGENFMPIWVKVLWTVIFMVM      57
Sequence-1                -----------------------------------------------------------M      1
Sequence-3                ------------------------------------------------------ILYAGM      6
Tribolium Tachykinin-R    MNFTQEFLYTTGYSIMDNSSEYDYTTNIS---YNDTEEGGNQFILPVWRQVLWSILYAGM      57

Tribolium Natalisin-R     ILIATGGNCIVIWIVTAHRRMRTVTNYFLVNLSLADLLLTTFNCIFNFSYMIQRDWPFGS      117
Sequence-1                VIVATGGNLIVIYIVLAHKRMRTVTNYFL-------------------------------      30
Sequence-2                ---ATGGNLIVIYIVLAHKRMRTVTNYFLRELKIA-------------------------      32
Sequence-3                VIVATGGNLIVIYIVLAHKRMRTVTNY---------------------------------      33
Tribolium Tachykinin-R    VIVATGGNLIVIWIVFSHKRMRTVTNYFLLNLSVADTMVSTLNVTFNFVYMLNSHWPFGE      117

Tribolium Natalisin-R     LYCIISNFIANATVAASVFTLTGISCDRYLAIVHPLQPRMSKRASLITITFIWLASMTVA      177
Tribolium Tachykinin-R    LYCKISQFIAVLSVCASVFSLMSISIDRYMAIMTPLRPRMGRTVTVLLAVTTWLLGVIIG      177

Tribolium Natalisin-R     FPCLLYSTTITNKYK-GVERTGCILIWPDGKVVGSHFDFAYQMFFLIITYVIPVTLMSFS      236
Sequence-5                --------------------------------------------------------MTYT      4
Tribolium Tachykinin-R    SPSLMFFRTYTMPYKDGEERVICYPEWPDGTTNESMMEYAYNVGFLFVTYVVPIGSMTYT      237

Tribolium Natalisin-R     YTIMGKELWGSRSIGEMTQRQIDSIRSKRKVVKMFIFVVFIFAICWLPYHGYFLYVYYDT      296
Sequence-5                YARIGIELWGSQSIGECTQRQMDNIKSKR-------------------------------      33
Tribolium Tachykinin-R    YARIGIELWGSQSIGECTQRQMENIRSKRRVVKMMMVVVIIFAVCWLPYHLYFIVISYFP      297

Tribolium Natalisin-R     DIIFSKYTQHVYLAFYWFAMSNAMVNPLIYYWMNARYVKT--------------------      336
Sequence-4                ----SAYIQETFLAIYWLAMSNSMYNPIIYCWMNARY-----------------------      33
Tribolium Tachykinin-R    EITNSTYIQETYLAIYWLAMSNSMYNPIIYCWMNARFRRGFKQFFSCLPFIHVSPGALTR      357

Tribolium Natalisin-R     ------------------------   336
Tribolium Tachykinin-R    REVLTSRRRSYSGSPDHNRIIRNG   381
```

Hycleus phaleratus

```
Tribolium_Natalisin-R     MSTTESWKEEEGNGS---FQGFDYGLLVPISSQTPVVVDSGENFMPIWVKVLWTVIFMVM      57
Sequence-1                ---------------------------------------GNQFILPIWRQALWSILYAGM      21
Tribolium_Tachykinin-R    MNFTQEFLYTTGYSIMDNSSEYDYTTNIS---YNDTEEGGNQFILPVWRQVLWSILYAGM      57

Tribolium_Natalisin-R     ILIATGGNCIVIWIVTAHRRMRTVTNYFLVNLSLADLLLTTFNCIFNFSYMIQRDWPFGS      117
Sequence-1                VIVATGGNLIVIWIVLAHKRMRTVTNYFL-------------------------------      50
Sequence-4                ------------------------------NLSIADTMVSTLNVTFNFVYMLNSHWPFGE      30
Tribolium_Tachykinin-R    VIVATGGNLIVIWIVFSHKRMRTVTNYFLLNLSVADTMVSTLNVTFNFVYMLNSHWPFGE      117

Tribolium_Natalisin-R     LYCIISNFIANATVAASVFTLTGISCDRYLAIVHPLQPRMSKRASLITITFIWLASMTVA      177
Sequence-4                LYCKITQFIAVLSVCASVFS----------------------------------------      50
Tribolium_Tachykinin-R    LYCKISQFIAVLSVCASVFSLMSISIDRYMAIMTPLRPRMGRTVTVLLAVTTWLLGVIIG      177

Tribolium_Natalisin-R     FPCLLYSTTITNKYK-GVERTGCILIWPDGKVVGSHFDFAYQMFFLIITYVIPVTLMSFS      236
Sequence-2                ----------------------------------------YNVAFLFVTYVVPIGSMTYT      20
Tribolium_Tachykinin-R    SPSLMFFRTYTMPYKDGEERVICYPEWPDGTTNESMMEYAYNVGFLFVTYVVPIGSMTYT      237

Tribolium_Natalisin-R     YTIMGKELWGSRSIGEMTQRQIDSIRSKRKVVKMFIFVVFIFAICWLPYHGYFLYVYYDT      296
Sequence-2                YARIGLELWGSQSIGECTQRQIENIKSKR-------------------------------      49
Sequence-3                ---------------------------------------------WLPYHLYFIVTSYFP      15
Tribolium_Tachykinin-R    YARIGIELWGSQSIGECTQRQMENIRSKRRVVKMMMVVVIIFAVCWLPYHLYFIVISYFP      297

Tribolium_Natalisin-R     DIIFSKYTQHVYLAFYWFAMSNAMVNPLIYYWMNARYVKT--------------------      336
Sequence-3                EITNSPYIQETYLAIYWLAMSNSMYNPIIYCWMNA-------------------------      50
Tribolium_Tachykinin-R    EITNSTYIQETYLAIYWLAMSNSMYNPIIYCWMNARFRRGFKQFFSCLPFIHVSPGALTR      357
```

Figure S1. When the *Tribolium* natalisin receptor is used as query in a tblastn search of genomic short reads from either *Hypothenemus* or *Hycleus*, the predicted protein sequences after translatation of the reads with the smallest e-values are more similar to the *Tribolium* tachykinin receptor. Yellow highlighting indicates identical amino acid residues between the short sequences and the two *Tribolium* receptors, red highlighting indicates different residues. Black highlighting indicates conceptual translation of residues that are part of an intron. Note that in all cases the short sequences are more similar to the tachykinin receptor. Hence, one can conclude that neither of these species has a natalisin receptor.

```
             1
Pogonus      MHRRW-AVVCVVYAAWASLATALPVTLLEDAKAAEAHLENKVKRSHMDESLNLNAESNSGAPVAYFSN--PSASKRA
Ignelater    ---MRSRLIWLGVVSYLTLVSSLPTSLVEDVKANEIK-DNKVKRAHSDT------EENPRISTAYYNN--PTAIKRG
Photinus     ---MPSKLAWLGVISYLTFASTLPTSLVEDVKANSIK-DSKVKRARSDT------EENPHLPAIYYKN--PSAIKRG
Aquatica     ---MRSNFAWLSIISYLTFANALPTSLVEDVKANSLK-DNKVKRAHSDS------QESSHMPVVYYKN--PTAIKRG
Nicrophorus  MKHTLRALP--FLLYFAALALALPASVVEDVKSSDIK-NSKVKRAPVNAE--------S----HGESR--PTAVKRG
Aleochara    MHPAHTGRTLAVLLTVIVVSSAIPASVLEDLISHDVA-NKKVKRAQVAVSK---ENPQPAQ--KELFY--PSAVKRS
Oryctes      MTLSRLHLPLLTVFSCILLISGLPTSLVEDVKDNEVR-NIKVKRAHDTLSR---SEKAPHDSTPYLSN--PTAIKRG
Coccinella   ---MGVQTRLLFLIFCSYGCSAIPSSMIDQIKAREMR-PNKVKRVSIAAEE---PEESLGIGNHYYGN--PTAIKRG
Harmonia     ---MGLQSRLLILIFCLYDASAIPSSMIEEIKARELR-PTKVKRVSMIAEG---PEESMKIGNHYYGD--PTAIKRG
Dendroctonus -MAKW-AFSSLLGAYLVAVSLALPSSLVQEIKNSEMK-SNKVKRAQSD----SNEPEENIGKMSYFTNRPTSAIKRG
Hypothenemus MNSKW-SFNCLLVASMIVLTWALPSSLIEELKNNDVNSGHKVKRGQPDSGTSGPSAEENMINTNYFDK-PTSAIKRG
Anoplophora  MGLRHVILDFLIVASSIVLVLGIPTSLVDEIKSSELR-DNKVKRAHPSDLL---TSEENGGDVPYYNK--PSAIKRG
Leptinotarsa MGLRQVYLDLLIVATLVASAAAIPTSLVEEIKSSELR-NNKVKRAHPPNLA---SSEENEGIIPYYTK--PSAIKRG
Aethina      MD-SHFGARLFVLTSCLALVCSIPASLVEEIKSSELR-DSKVKRTPS--------NGEQNSEIQYFTK--PSAAKRG
Hycleus      MEVIWLCFKWIVWTAYLTTVLAIPGSLVDEIKANELEANNKVKRAHSI-------VEETVKDVPYYGK--PTAIKRG
Tenebrio     MELRC-SLKWATLASCMVLTLAIPASLIEEIKASELR-NNKVKRAHPPM-N---NVEERSRDVPYYSK--PTAIKRG
Tribolium    MELRW-SIRWATLASCLALSFAIPASLVEEIKTNELR-NNKVKRAHPQL-N----VGEHGREVPYYSK--PTAIKRG

             78
Pogonus      SNLNTDV---SDEAINEWAEQQQQQQQQQQQQPKIDPLKMIGYDDNPFE-DKTIAEYEKGFHYGTNKAKLDEQMENA
Ignelater    TSQ----------TLGGWEDQS------KESLANLAPS-LYSSDDGQFD-DKSLLDYEKAYRYGTNKEKLDEALENA
Photinus     TSQ----------PFDNWEDLN------KETFINLAPS-LYSN-DGKID-DKSIVDYEKAYQYGTNKEKLDEALENA
Aquatica     TNQ----------PMENWEDLN------KESLLNIAPS-LYSTYDGKFD-DKSLLDYDKGYQYGTNKDKLDEALENA
Nicrophorus  LNLKNGQ----LPLLTEWDQQRFRFPS--EEEPFAASP-LYTSED-ELGNDKTVAEYEKGFRYGVNKDKLDEALENA
Aleochara    EGNRLKS--SQLPLLTEWDQQRYR-----EQAERVSPS-LYSSF------DDSPLEYAKALRYASNRDQLDEKLQNA
Oryctes      TNFND--------QIGGWDQDQ-SIFQNT----DIPSS-LYDSD--QI-DDKSIAEYEKGYRYGTNKDKLDEALENA
Coccinella   TEMKNEAADDESSDDNDWSPSEQAIYQNVDN--DFQST-VYNDE--SSQSANDMDDYDKGLQYGN-REKFDEAIENA
Harmonia     TEMKNEVPEDESSSDTDWVQSQQGIYQDVDN--DFQST-SYNDE--SSQSAKSVDDYDKGFQYGENREKFDEAIENA
Dendroctonus TNLKEAD-------MGEWNRDQL-YGGRDFSLDNIQSG-LYDFPNLPMD-DKTIAEYEKGYQYGTNKEKLDAALQNA
Hypothenemus TNFQDKY-------LGDDLLDFG-N------GDDIQSG-LFN---SPLE-DKSLNEYEKGYRYGTGKEKLDEALENA
Anoplophora  TNINLKSPLPDQQSLNEWEREQ-SIFRNQAVLPNIPSS-LYNSPDDSYD-DKTIAEYEKGFRYGTNKDKIDEALENA
Leptinotarsa ANIDLKSLP-----DLDWEREQ-ADFINRNTLPNISSS-LFQYPENNFDDDKTIAEYEKGYRYGTNKEKLDEALENA
Aethina      AN--LKNANAEQQSLSDWTQDQ-VLT---DSLSSIQSS-LYNNPNQ-YD-DKTVEEYEKGFQYGASKDKLDEALENA
Hycleus      TNQ---LPLIILSPLSDWEHEQQTFYENPENLANIQSS-LYNSD--NFD-DKTIAEYEKGYHYGTNKEKLDEALENA
Tenebrio     TN-SLKNPTPDQQSLNEWEQEQ-SLYQSPEGLANLQSG-LYNNADAPFD-DKSVAEYEKGFRYGTNKEKLDEALENA
Tribolium    AN-NLKNPSPEQQSLSDWEQEQ-SLYQNPDSLADIQSS-LYN-AENPFD-DKTIAEYEKGFHYGTNKEKLDEALENA

             155
Pogonus      ILKSEMYGDPASI-NQYRYYGGSDDRRRKRSN----------LKRSASFANRYKREVELSPEDILTLLALWDEDRLR
Ignelater    VLKSELYGDPGAL-NQYRYFDGSSDERRR-KR---------RAIKNLRLNSRYKREIELTPEEVLTLLTLWENERRR
Photinus     VLKSELYGSPGSA-NQYRYFEGGNDEKRK-KR---------AAF--KRLNSRYKRDIELTPEEFLTLLSLWENERRH
Aquatica     VLKTELYGNPASF-NQYRYFDADER--RK-KR----------GT--YKLKDRFKRDIELTPEEFLTLLSVWENERRL
Nicrophorus  ILKSELYGEPAAV-NQYRYYGMEGD--KKRKR---------RNAQKTRMDNRYKREVDLSPEDILAILTLWENERQH
Aleochara    ILKSEMYDLPATLPNQYQYYGNDDRRRKRRSTNGKLAKKAARVQKARSITNRLKRSADMSPEQILAILTLWENERMN
Oryctes      VLKSELYGDPVSI-NQYRYYGGVADEKRRRRR---------SNAQKMRFDTRIKRDVDLSPEDILTLLSLMENERQR
Coccinella   VLKSEFYGNLEGD-------------KKKRKK---------RNSRQYSYGNS-AASDDLSPEEVLDLLTLYESERQK
Harmonia     VLKSEFYGNLDND-------------KKKRKK---------RSSRLNGYGNS-IASDDLSPEEVLELLNLYEGERQK
Dendroctonus ILKSELYGDNY---GPSIWPYEDRRRR---KR---------ETVSNKRNTLRYKRNVDLTPDEVLRLLQFYEKNQRE
Hypothenemus ILKSDLLLDPY---RRYN--------------------------------NRSKRTVDLTPEGMAALLSLYEKNHR-
Anoplophora  VLKSELYGEPGAI-NQYRYYGADD--KKR-KR---------REVKKLRFDGRIKREVDLTPEEILALLSLYESNRQR
Leptinotarsa VLKSEIY-EPTSI-NQYRYFGAEAA-KKR-KR---------RDAKKLRLESRMKREVDLSPEEILTLLSLYENNHPR
Aethina      VLKSEYYGEPGAV-NQYRFYGGDD--RKR-KR---------RSAKKLRLSQRLKRDVELTPEEILTLLNLYEKSRQN
Hycleus      VLKSELYGEPGAI-NQYRYYGNDDK-KRRRRR---------RDVQKSSRTNRLKRDTDLTPEEILSILTLYENEHQN
Tenebrio     VLKSELYGDPAPL-NQYRYYENDDR-RRR-RR---------RDARKIRPDNRIKREVDLTPEEILTILTLYENERQS
Tribolium    VLKSELYGDPAPL-NQYRYYGNDDQ-RRR-KR---------RDARKIRLDSRMKREVDLTPDEIFTILTLYENER--

             232
Pogonus      Q---PAGYRTNWPRYNPDIDLDNDREE---NEIDEPEESWLESPV-YPGIRNP-VHAS--PF---ARE--YAHNQVQ
Ignelater    A---QDDYRPTWSRYEPNLDLDDSSEN---D-VEQEDENWLDTPVVYPHATNR-INQLSGEFVRGNPNYIYEDK---
Photinus     T---SDDYRPTWSRYEPNVDLDDRNEN---E-LDEDDENWLDTPVVYPHATNR-----------ASPNYMYEDK---
Aquatica     S---QDDYRPTWSRYEPSVDLDDRNEN---D-VDQDEENWLDTPVVYPHATNR-----------GNPNYIYEDK---
Nicrophorus  GPP--ANFHPSWQRFD-NFDMDSISGDKNEPEIEQNDEDWLDSPV-YPHISEH-LSPM-------NPNYYY-DS---
Aleochara    K------DATPWLNYDEDGYGD--------MSSTDQDEDWLEAPV-HPHATDE-H------MMPQSPNYYYDVSPQA
Oryctes      P-----SNYRNWQRYDSNLDLDKDNQD---SYEEEDEGTWLDTPV-YPHVGPH-TNSM-------PSSN-YYYE---
Coccinella   P----------------EDTL---------SNLEQDNSIWLDVPV-RPGAILPSTSNF-------GPAYHLDRSPSN
Harmonia     P----------------EMDI---------GNFDQDHAVWLDVPV-RPGPVVPNTSNL-------GPSYSLDRSPLD
Dendroctonus LK---------------NGEFY--------PVEDNNDETWFNEPMRYG-SV-D--RP----------ANNRFHQ---
Hypothenemus -S---------------D-PYE--------IADEDEDGAWLNEPVVRPHSSIE--NS----------VYNTRNL---
Anoplophora  QQE-SNNYRQPWNRYEPNFDVDINQDD---S-QDQDEENWLDNPV-FPHATGY-DKEL-------GPKYMIDQS---
Leptinotarsa QQPSTENYRYPWNRFEPNLDLDINQDD---S-QDQDGENWLDNPV-YPHATAF-EKDL-------GPKYMYEPS---
Aethina      D------NYRPWQNRLEN-SFDNFEDG---E-LNDNDESWLETPV-YPHVSRD-NTDI-------GPQYMIDE----
Hycleus      ---------RPWRLDDF--------DK---S-NEQDDETWLDGPV-YPQTNG---NDI-------GPGYMFD-----
Tenebrio     N------GYRPWSGEP-D-----NDQN---N-NIEEEENWLDAPV-YPHAAGH-TNDI-------GPSYLLDE----
Tribolium    N------GYRPWGLEP-E-----PS-G---D-NLEEEENWLDAPV-YPHATGH--NDL-------APSYLMDE----
```

```
               309
Pogonus        FEQPPIFAQKRQWAGF-SD-KKRKRFMVARKR-SDPTRELRYLNGPAQSHEDLYSLTQLLGPPPARD---GNFPMYH
Ignelater      --RS----QLGQWGGF-SD-NKKKRFMVARKR-NDPTRELRYFNGPAQ-RNDFYTLSQLLA--SQRE---PNVPLYH
Photinus       --R-------GEWGRF-PE-NKKKRFMVARKR-NDPTRELRYINGPPQ-RSDFYTLSQLLA--AQRE---PNAPLYR
Aquatica       --R-------GQWGRF-SE-NKKKRFMVARKR-NDPTRELRYINGPPQ-RNDFYTLSHLLA--NQRE---PNAPLYH
Nicrophorus    --KPQVYEKRRMWGEY-PD--KKKRFMVAKKS-NDPTREIRFLNGPT--RNNYYTLSELLGA-AQQE---QNVPLYQ
Aleochara      PPVPQFYEKRPRWGGF-NE-NHRKRFMVAKKR-GDPTRELRYLNGPS--HSDYYTLSELLA--NQRD---PDVPLFQ
Oryctes        --KPQSYEKHNQWNEV-AE-SKKKRFMVAKKR-NDPTRELRYLNGPN--VNDYYTLSQLLG--NQRE---PNIPVYH
Coccinella     FEK-------SRWS----DTRNNKRFMVPKKR-NDPTREIRYINGPT--KHDYYFLSQLLN--NRRE---TNVPVFH
Harmonia       YER-------SKWL----ENK-NKRFMVAKKK-NDPTREIRYLTGPA--KHDYYFLSQLLN--NRRE---ANVPVFH
Dendroctonus   --INSQPDYKPRWGAFDPATFKKKRLFY-------------------------------------------------
Hypothenemus   --LDDIYHLKPRWGSN-IDFNKYKRFPMAKKRTTDPTRIIRYLNGPS--QNDFNTLSSILN--SQKEI-EIGVPVYR
Anoplophora    --NSQMFDKKGRWGGF-GD-NRKKRFMVSKKRTNDPTREIRYLNGPN--KNDFYTLSQLLS--NQRE---PNVPVYH
Leptinotarsa   --GPQVFENKEKWRGF-GGDSRNKRFMVAKKRNVDPTHELRYINGPN--KNDFYTLSQLLS--NQRE---PNLPVYH
Aethina        ---PQLYDKRGKWGNF-AD-NKKKRFMVAKKR-NDPTRELRYINGPN--QNDYYTLSRLLS--NQQE---PDLPLYR
Hycleus        --------DKRRWGNL-VDNERKKRFMVAKKR-----------DDPA--RNNFYTLTQLLS--NQRE---PNVPVYH
Tenebrio       ----KPFEKRGRWGGF-AD-NRKKRFMVAKKR-NDPTRELRYLNGPN--KNDYYTLSQLLS--NQRE---PNVPLYH
Tribolium      --------KRGRWGGF-AD-SRKKRFMVAKKR-NDPTRELRYLNGPN--KNDYYTLSQLLS--NQRE---PNVPLYH

               386
Pogonus        RMVL
Ignelater      RFVL
Photinus       RMVL
Aquatica       RMVL
Nicrophorus    RYVL
Aleochara      RYVL
Oryctes        RRVL
Coccinella     RYVL
Harmonia       RYVL
Dendroctonus   ----
Hypothenemus   RPIL
Anoplophora    RLVL
Leptinotarsa   RLIL
Aethina        RLVL
Hycleus        RLVL
Tenebrio       RLVL
Tribolium      RLVL
```

Figure S2. Sequence comparison of Coleoptera baratin precursors.

```
                1
Pogonus         --------MHSCT-LILITFGIIAVLMTCSSAS----AIPQPPCSPSVLMT-ADAKYRQICS
Ignelater       --------MHQCT-ILIASVGILAAFLTSSSVT-AS-VDTTNLCPPDASP-----KMRQLCL
Photinus        --------MHQYK-IIIASICIFAGLIS-STVN-LS-VETTNLCSSDASP-----KLRQLCL
Aquatica        --------MHRYT-ILVASVGVLVALLTS-SVA-IN-VETTNLCPSDASP-----KMRQLCL
Nicrophorus     --------MNQCT-VYAVAF-IFVAMMASSNLS-MASNLPLIYCQPSALD---GLQKRQLCF
Aleochara       --------MHRESSYFALGVGLLVVILAARTTTTSAAHIPVIVCPPNPNSADVPSQLRQLCF
Oryctes         --------MNYST-DIIATFGIISIFLISSTSS---TTIPMINCEPNSLS-EAPTKIRQLCY
Coccinella      --------MHLYI-TSALLWGVLSILMH-------SSSASVISCPPNNLP-EAGVYLRQLCS
Harmonia        --------MHLNF-VAIFFFGVLATSML-------STSASVVSCPPNSLP-EAGIYLRHLCS
Dendroctonus    --------MQQST-VYF-FFGMLVFVLLC-----SMTSATVISCPPNSIQQEINPKLSQLCF
Hypothenemus    --------MHQSV-VCF-FFGMVVFAFFY-----SVATASVISCPPNSVQQEINPKISELCY
Anoplophora     --------MQTYT-VFSLIFGVVTLILTS-----SISKASVISCPPSSFQEQSNPALRQLCY
Leptinotarsa-1  --------MHSST-IVSCVVGTIIVILFS-----SVARASIISCPPNPLQEQVHPALRQVCD
Leptinotarsa-2  MFTHTNSQMNSFM-MVSFIFWM--AVLS------STSQAYVINCSPVSVQDQMNPGVRQLCA
Leptinotarsa-3  --------MHFSA-VISFVVSLVSAVLS------SEVNAYIISCPLSSLQDTSNSGLRQLCS
Leptinotarsa-4  --------MDFLL-KIYFLVSL--MFGI------NVSNAYIINCSPSTLQIQANSRERQLCA
Aethina         --------MQQHT-FAIFVLGVVSVFLSC-----STSTASVISCPPNNL--EVAPNLRQLCY
Hycleus         --------MHQYT-FFII-FGFVAVFVSN-----SLITASIISCPPSNLQ-EANPNLRQLCY
Tenebrio        --------MHHYT-LVAV-AFAAVAALLT-----SSATATAISCPPNPL--EASPYVRHLCY
Tribolium       --------MQQYA-FVAIVFGAVAVFLAN-----ASTTAYYVSCPPNDAL-EASTSLRHLCS

                63
Pogonus         LLSQ--FARDLEFSSDDRV------PDMSELI-------DNGIKRKDVDHVFLRFGRPRM--
Ignelater       LASLEQFLAENPP-RPNAD------VLDTQLY-------DRGTKRQDVDHVFLRFGRRLGL-
Photinus        LASLEQFLSENPS-RSNAD------VQDTQLY-------DRGTKRQDVDHVFLRFGRRLGL-
Aquatica        LASLEQFLAENPP-HSNAE------PMDTQLY-------DRGTKRQDVDHVFLRFGRRLGL-
Nicrophorus     AL-LER--MDAPQ-EVSND------VMDNQLY-------ERGIKRQDVDHVFLRFGRRMGL-
Aleochara       VL-LDKNDIQIPLEDGDLAAAGIDPAQAAQLYERHLVEGSRGTKRQDVDHVFLRFGRRSGM-
Oryctes         YV-MEQLRSEQ-P-QDNGE------MIDSKLY-------ERGIKRQDVDHVFLRFGRRFGF-
Coccinella      AI--KQAINDNSPPEEPYY--------F-----------GGNSKRQDVDHVFLRFGRRLGFE
Harmonia        VI--KQAINDNAPPEEQFY--------F-----------GGNSKRQDVDHVFLRFGRRHGFE
Dendroctonus    AI--EQALQDSSPQNDAYT--------ARLVDERNT---NLNAKRQDVDHVFLRFGRRFGM-
Hypothenemus    AI--QQTLQDSSPQNDEYI--------GQLLDERTASNLRTNAKRQDVDHVFLRFGRRYGL-
Anoplophora     AI--EQAVDEVVPQQTQFP--------GRLEE----RNTNLNAKRQDVDHVFLRFGRRFGL-
Leptinotarsa-1  AI--EQAIVDI-SQQQAFV--------GRLEE----RNTNLNTKRQDVDHVFLRFGRRFGV-
Leptinotarsa-2  VL--DSTID-LTS-PPAYS---------GLLG-----ESISLPNDKKLDHSFLRIGKRFHL-
Leptinotarsa-3  IL--EQAVDDLPQ-QAVYS---------GIVG-----DSNTLSSGKRVDHSFLRIGKRFNF-
Leptinotarsa-4  LF--DPAID-LVA-QTAYS---------GILG-----ESVTLPNDKRVDHNFLRIGKRFNL-
Aethina         AI--EQAISEVQNNQAEYH--------FDERN----APGQVEKKRQDVDHVFLRFGRRLGY-
Hycleus         AI--EQAISENAISQDQ-F--------GRMDE----RNVNPNAKRQDVDHVFLRFGRRFGL-
Tenebrio        AI--EQAISENAITDDQ-Y--------RRVEE----RNVNGNAKRQDVDHVFLRFGRRLGL-
Tribolium       FI--EQAVNDNVVIQDEPF--------RRIVG----RNVNPNTKRQDVDHVFLRFGRPFGL-
```

Figure S3 Sequence comparison of Coleoptera myosuppressin precursors.

```
Aquatica      HLNGRTLDELGGGHLIG-RTLDELGGGHLNG-RTLDELGGGHLNG-RTVDELGGGHLNGRNAY
Nicrophorus   NLNGRALDGIGGGNLNG-RALDGIGGGNLNG-RALDGIGGGNLNG-RALDGIGGGNLNGRSYL
Harmonia      NLRKRALDSIGGGALLGKRSLDSIGGGALLGKRALDSIGGGALLGKRALDSIGGGALLGKRAL
Coccinella    MLRKRALDSIGGGALLGKRALDSIGGGALLGKRALDRIGGGALLGKRALDSIGGGALLGKRSQ
Dendroctonus  LASRRRLDSIGGGGILSARGLDSIGGGGLL--RSIDDIGGGGILSARSMDGIGGGGILEARSM
Anoplophora   TLMGRGLDGIGGGNLMG-RSLDGIGGGSLIG-RGLDGIGGGTLMG-RGLDGIGGGNLMG-RSI
Leptinotarsa  SLIGRSLDNIGGGALIG-RSLDNISEGSRIG-RSLDPIGGDSLIG-RNLESIGGAVKRTIDNI
Aethina       LYRAPTMDDIGGD-VL-KRSMDDIGGD-VL-KRSMDDIGGD-IL-KRSMDDIGGDVLRSLYRA
```

Figure S4. Sequence alignment of parts of the orcokinin B precursors from the species indicated. Note that the putative convertase cleavage sites consists of a single Arg residue in most species, but that in the two Coccinellids, Harmonia and Coccinella, all have been replaced by a Lys-Arg pair, while in Aethina several single Arg convertase cleavage sites have similarly been mutated into Lys-Arg doublets.

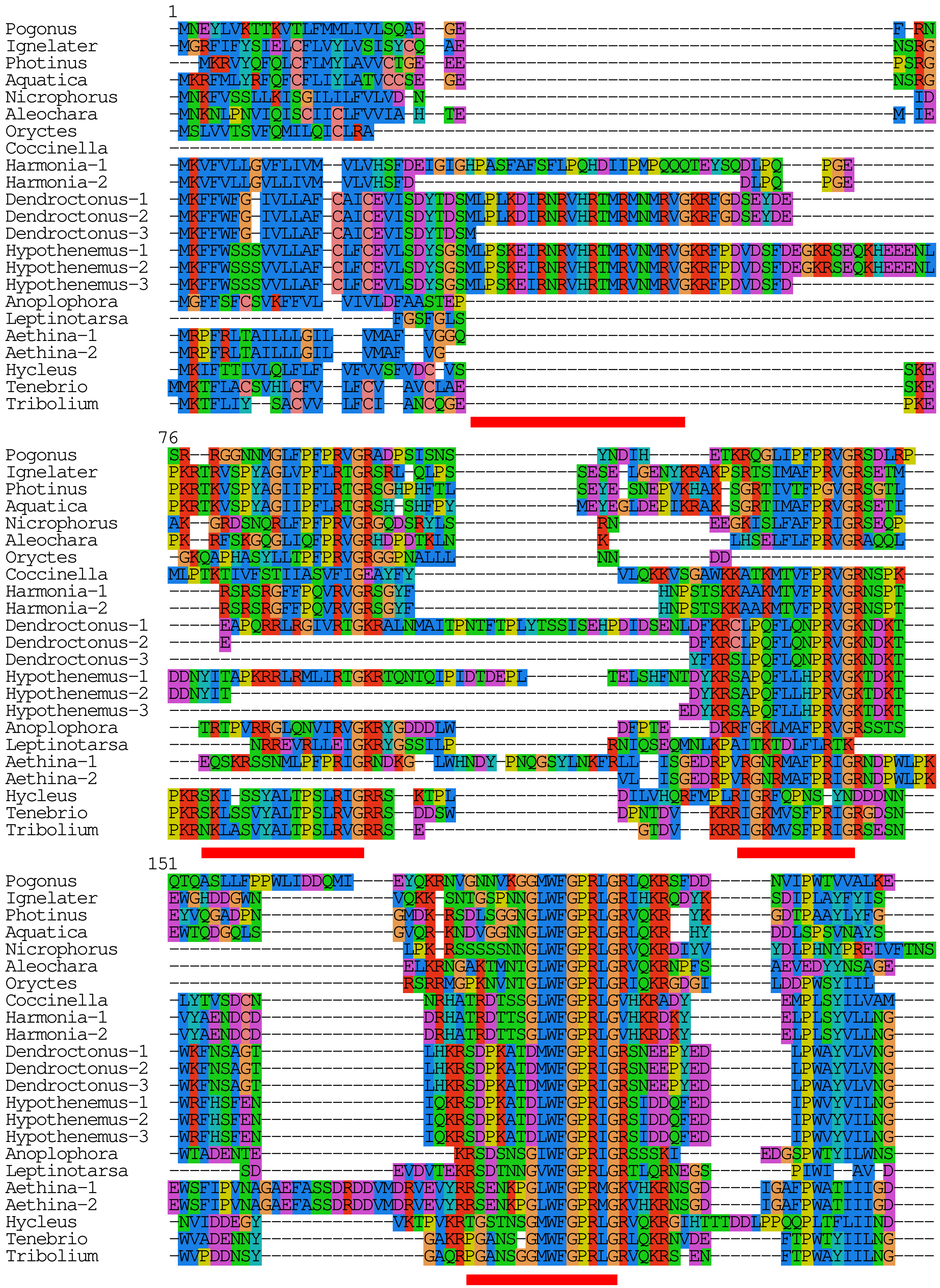
1
76
151
Pogonus
Ignelater
Photinus
Aquatica
Nicrophorus
Aleochara
Oryctes
Coccinella
Harmonia-1
Harmonia-2
Dendroctonus-1
Dendroctonus-2
Dendroctonus-3
Hypothenemus-1
Hypothenemus-2
Hypothenemus-3
Anoplophora
Leptinotarsa
Aethina-1
Aethina-2
Hycleus
Tenebrio
Tribolium

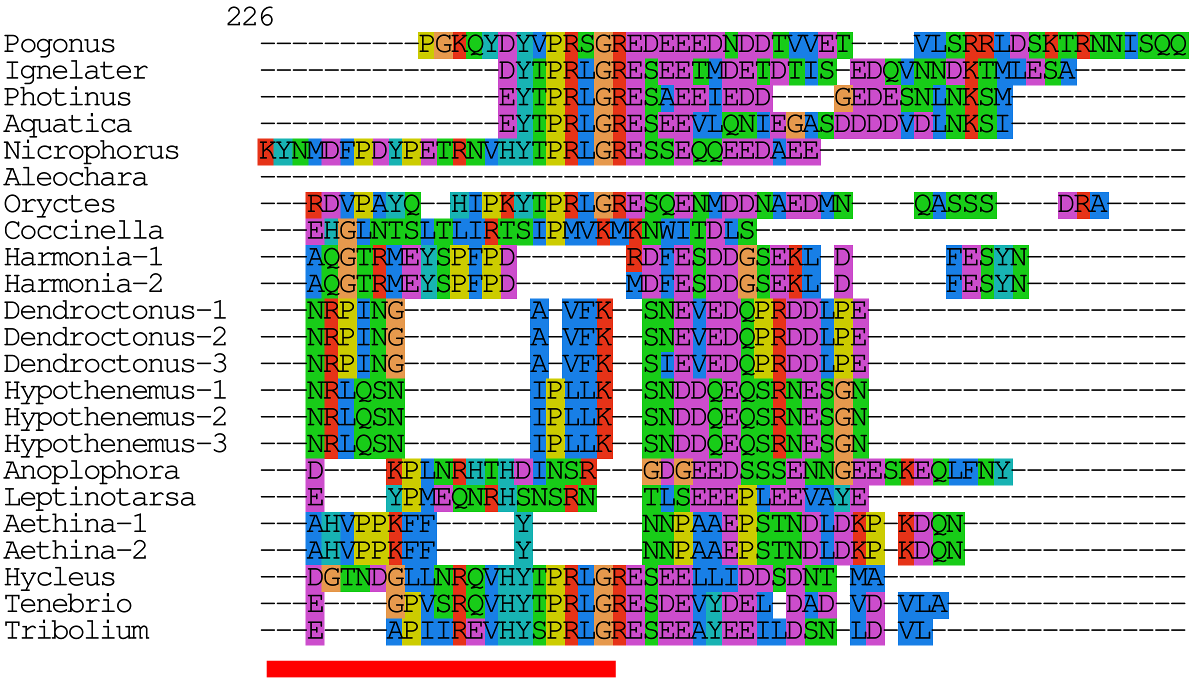

Figure S5. Alignment of periviscerokinin precursors.

ARCHOSTEMATA

Micromalthus debilis (GDOQ01020265.1)
MALLWALVLFPAAILASGTEQEPGIYPQYYSRYPDREEPPFAFPEEFQSDQRKTVQKSYEEIAEDLSKLSNNLQ
QKLRFASALLKQENPYETDPADRIEISNLQNSLCILNAEIDNYFRQKAGVFSKGNTQAKRSVWDSKKSTFDSAG
RTSPIELANKSPNIYKSNSNLANAYAQPLFTMKFDATPTDGRTPVQRKRREITKAQLENLLQDEIEKQEEEEWE
RIEGEDDFKKRSQNPPTVVLKSGEVDIPKRVPFNPWGGKRLEQFPVNRDLLGVWESKRDRMPFDPWGGKRARVP
FDSWGGKRDRTAFDSWGGKRDRLPFDSWGGKRSSRVKLSPWAEGKRPKQRKSQFYSWGGKRSKYDATLV*

Priacma serrata (GACO01006445.1)
-----GGDDTDQKRTKFFSWAGKRTNDGTEPSFEFLPSFKRSHSSPNIVVKPREMALPKWTPFHSWGGKRAVQV
ESERLPISRVPFNAWGGKRSSDHEDNEFLPLHQEMTSLPHNPEGEFFKSVQTIETEVEVSQLGWKEKSVLTLRN
IKMLPL*

ADEPHAGA

Pogonus chalceus (JU438197.1)
MYYKNSVIQTSKILLQIILYILIQFANTNCMTIPNDNSNMLQTKSIRNRLNLLEDDNIFGTETPTDTKTYNSGN
DMENNNNGDIFTKETDNENIFTEDSDIDDKKLNVNKIVSLLLDPNEVTPKMIEKLSQFKTFYLSNSNDKTLINL
CTLSRNYINDVCLLIQSSNRSNGLYNPYIYQRCVKLLPLLDFYHCVNEKQNYDETAKENEFKRVPFHAWGGKRF
RQVATEKVQSSKVPFNAWGGKRQMNYDDGDEYCKRSKSKFHSWGGKRTVRETANVL*

Gyrinus marinus (GAUY02012896.1)
-----QMLMDASKQVDDEDAKHVKFLGWIGKRFSNANGNNIPKRIPFHAWGGKRSNQVGTEKIQASRVPFNAWG
GKRTYNMNSDESTPDNMYEINTPSSVLPYLVYKRSRTKFHSWGGKRNY*

Figure S6. Predicted partial or complete Coleoptera leucokinin precursors as obtained from conceptual translation of TSA transcripts found at NCBI. The nucleotide identifiers are indicated in parentheses. Lys-Arg and Gly-Lys-Arg processing sites are indicated in purple and the amino acid sequences of the mature peptides are in green.

```
                  1
Ignelater         NLEDLASLNLEQIEMDNVDVSIFSRLNKLESVYFKKYHYCAFAPTVPRCRPLTDGVSSAHHLLEKPVLRYATWI
Photinus          KMENLQSLNLDGIEIENIDLSIFSTTPNLKSVYFKKYFYCTFAPRVRRCRPLSDGISSVDQLLAKPILRYAIWI
Aquatica          NLKNLESLNLEGIEIHNIDVSIFSSLKRLKSIYFKKYFYCTFAPSVKRCRPLSDGISSVDELLAKPILRYALWI
Dendroctonus      NLQNLLSLHLEEIDITNIHTDIFASLTNLQWIYFKTYKYCTYVPTVPRCRPLSDGISSINQLLFKPIFRYSNWI
Hypothenemus      NLPNLLSLHLEDIEILNIDTKMFGGLDGLKWIYFKTYTYCTFAPEVPRCRPLSDGISSLNQLLYKPIFRYSNWL
Anoplophora       NLINLNLLNLEDIEIRNIHTLMFINLFSLESVYFKKYSYCLFVTSVERCRPLSDGVSSNAQLLYKPIFRYTNWI
Leptinotarsa      NLTQLQHLNLGDIDILKIDASMFDSLKELKRIELKNL-ICRYN-KVLSCR-ASDGISSANNILNKGIFRFWNWV
Aethina           NTINVRSLNMEGIEITNIDLGMFEHLSDLQSVYFLKYSYCSYSPMVPRCRPLSDGISSTKQLLYKPILRYSNWI
Drosophila-LGR3   EPLNISFLNLTGIRYDHIDFEAINSMRNLTYIIYDRFFYCSMTPRVRMCKPSTDGVSSFQDLLSKPVLRYSAWV
Drosophila-LGR4   CLKNLSSLLLTGNRFQTLDSRVLKNLTSLDYIYFSWFHLCSAAMNVRVCDPHGDGISSKLHLLDNQILRGSVWV

                 75
Ignelater         TCLIACAGNISVIWGRLTV-RDENRVLSFVVRNLAASDLLMGIYLLIIGFHDVKFRNIYNKVSHQWMSSWLCTT
Photinus          TCFVTCIGNAMVIYERMTS-RDENKVLSLVIKNLAASDFIMGIYMVIIAGYDLKFRNIYNKVAHEWISSWPCTF
Aquatica          TSLFTCVGNAMVVCGRVMY-RDENKVLNLVIRNLAVSDFLMGVYMIIIAFHDFKFRNIYNKI-HIWMSSWFCTA
Dendroctonus      MCIITIGGNSLVLFGRFLF-RDENKNLSVVIKNLAVSDGLMGIYLLIIAYHDLKYRDNYNEIARKWMSSWNCVI
Hypothenemus      MCVITIGGNSLVLFGRFLF-RDENKNLSIVIKNLAVSDGLMGIYLLSIAYHDLKFRDNYNEMARKWISSWTCVF
Anoplophora       ICFATCAGNIMVLFGRYLF-RDENKVLSLIIKNLAVSDLLMGIYLLLIGIHDMKFRNIYNLEAQNWISSWGCTI
Leptinotarsa      ISSAICISNFLVIFSRLFY-RDENKILNLVIRNLAASDFLMGVYLIIIGIYDVKFRGVYGEMSEQWLSSWTCTF
Aethina           ICFITISANTLVLFGRYMF-RDENKALSLVIRNLAISDLGMGIYLLIIAVQDWRFRNAYNQYAHAWISSWGCTV
Drosophila-LGR3   MATLTIAGNVLVLWGRFIY-RDENVAVTMVIRNLALADMLMGFYLVTIGVQDYRYRNEYYKVVLDWITSWQCTL
Drosophila-LGR4   MASIAVVGNLLVLLGRYFYKSRSNVEHSLYLRHLAASDFLMGIYLTLIACADISFRGEYIKYEETWRHSGVCAF

                149
Ignelater         AGILAMTSSEVSVLLLVFMSVERCLLITAPFGHLASLTLKPAFSILFSIWTVGIGLAVIPVIEFYS-STR-FYG
Photinus          AGVLAMVSSEVSVLLLVFMSVERFLIIAVPFGRFSSIDVKKCALILGCIWILGVGLAIIPVLQFYS-STK-FYG
Aquatica          AGVLAMVSSEVSVFLLVFMSVERLLIIAVPFGPFSSMDLTKTVTVLXSIWILGIGLAVFPILQFYS-STR-FYG
Dendroctonus      TGIIGMTSLEVSVMILVFLSVDRYFVITMPYKKYGSLTLKETWRVILCIWCFGICISVIPALGLLS-STK-FYG
Hypothenemus      TGVVGMISLEVSVMFLVFLSVDRYFVITMPYKKYGVLSLKETWRIVFFIWCFGISISVIPVLGFSS-STK-FYG
Anoplophora       TGIIAMLSCEVSVLLLVFMSVDRFLLIAIPYGKYSALTMREAVIALLFIWCLGIILAVFPALAYLS-STR-FYG
Leptinotarsa      TGITAMISSEVTVFILLFISIDRYLVIVTSYGKYKSLNEKDTVIVLTIIWCLGIAISVIPVIRYAD-DTK-FYG
Aethina           TGMIGMLSSEVSMLLLLFMSIDRYLLIAVPFGRYHALTLRKTTLILTGIWLVGVFITVLPVIEYHN-STR-FYG
Drosophila-LGR3   IGTLAVSSSEVSMLILAFMSLERFLLIADPFRGHRSIGNRVMWLALICIWITGVGLAVAPVLLWRT-STLPYYG
Drosophila-LGR4   AGFLSTFSCQSSTLLLTLVTWDRLMSVTRPLKPRDTE-KVRIVLRLLLLWGISFGLAAAPLLPNPYFGSH-FYG

                223
Ignelater         -VNSLCFPLHISDPFFLGWKYSAIIMLGVNSASLIIIAVVYCVMFISIWKTRHATSLP--LRDFEFAIRFFFIV
Photinus          -LNALCLPLHTTQAYHVGWEYSAFIVLGINFVSLITITLVYCIMFISILRTRSATTLP--RKDYEFAIRFFFIV
Aquatica          -LN-LCVPLYITEPFFVGWKYSAFIILGINFISLIVITVVYSVMFISIAKTRNATTLP--IKDYEFAVRFFFIV
Dendroctonus      -VNGLCLPLYIDEPYLIGWEYSAIIFFGINASSLLIIIYTYGGMFINIKNTRNATSMP--MNDCQFAIRFFFIV
Hypothenemus      -VNGLCLPLYINDPYFIGWQYSALIFFGVNASSLIIISYTYARMFINIKNTRKATSVT--LNDGQFALRFFFIV
Anoplophora       -VNGLCFPLHIDDPYLEGWEYSAVIFFGINATGLIIISVVYIGMFISIWRTRNATNLP--ANDYQFAIRFFFIV
Leptinotarsa      -INGLCFPLHIAYPYYSGWQYTAFIIFGINATSLIIIAFVYIGMFISIRKTREATPLP--KKVYEFVLRFFFIV
Aethina           -VNGLCFPLHIDDPYFIGWEFSVTIFVGLNAFCMIVTTVVYIGMFVSIWRTRNATTLP--SKDYEFAVRFFFIV
Drosophila-LGR3   SYSGTCFPLHIHEAFPMGWLYSAFVFLGVNLLLLVMIAMLYTALLISIWRTRSATPLT--LLDCEFAVRFFFIV
Drosophila-LGR4   -NNGVCLSLHIHDPYAKGWEYSALLFILVNTLSLIFILFSYIRMLQAIRDSGGGMRSTHSGRENVVATRFAIIV

                297
Ignelater         LTDASCWFPIILVKVFALTGMEISADLYAWLVVFVLPVNSAVNPLLYTFTTPKYRSKINKVAVPLHCIRDYGNM
Photinus          FTNGCCWFPSVLVNVLLLLGKEISGDLHAWLVVFILPINSAINPILYTFTTPKYRFNSKKVLVFFNMISQSQRS
Aquatica          LTNGCCWFPIILVKCLVFFETEIPNDLYAWLVVFVLPINSAINPILYTFTTSKYRSKLQKVNIMLHWILKNERS
Dendroctonus      LANIACWVPIITAKMLVYLKVDISGDFYAWLVVFVLPINSSLNPILYTFTTRKYRRRILKVPSCLKKFTTVNNL
Hypothenemus      FTNVACWLPIILAKMLVYFTVEVSADFYAWLVVFILPINSSLNPILYTFTTRKYREKIVNMPQCLRKCAYQSRP
Anoplophora       LTDVTCWLPIVTIKAVAFLGVAVSDDLYGWLVVFILPINSALNPILYTFTTPKYRTQILKKVPSLIKEAAVNLS
Leptinotarsa      FIYGLCWLPIIVFKTAAYAGADISDELYGWLVVFILPINSASNPILYTFTTRKYRAKMKISCIFEGVMDRTHHE
Aethina           LTELFCWLPIITIKVIVLFQFSVSDDTYGWLVVFILPINSGLNPILYTFTTPKYRREIYRVPMSVRESTSNKTR
Drosophila-LGR3   LTDFLCWVPIIVMKIWVFFNYNISDDIYAWLVVFVLPLNSAVNPLLYTFTTPKYRNQIFLRGWKKITSRKRAEA
Drosophila-LGR4   TTDCACWLPIIVVKLAALSGCEISPDLYAWLAVLVLPVNSALNPVLYTLTTAAFKQQLRRYCHTLPSCSLVNNE

                371
Ignelater         VLSDNIRRQSPQSHL--------------------------
Photinus          SLEKTTQGQTVPSSGGAFLTSSSI-----------------
Aquatica          NLKKNSRHPPVPSSAGAILNSSSL-----------------
Dendroctonus      TETHLPGAIFKITQKDIKTVNN-------------
Hypothenemus      SETQLSREIFTISQRDIEREKYF------------
Anoplophora       KNNGSKICSGEIDHLKLTCAKENNLNKTNVKIVLNKF
Leptinotarsa      DPSLTDPSDVESDCGVSKSKSTTEKRF------------
Aethina           TYDKGSPGTYHSVFDLKKFLQYQTPFVQLI----
Drosophila-LGR3   GNGNVATTTTGTATGSSQHPDDFTIFAKAAMRCH----
Drosophila-LGR4   TRSQTQTAYESGLSVSLAHLGGGVGGGSGRKRMSHRQMSYL
```

Figure S7. Alignment of N-terminal parts of Coleoptera LGR3's; for comparison the LGR3 and LGR4 from *Drosophila* have been added.

```
                  1
Pogonus-EH1       -------MQTKDSYHKIVLIFFV-LFVMIIMTSANPAIDYFPKPFIYTKNYGICIRNCAQCKKMFGPYFEGQL
Ignelater-EH1     ---------MKI----FLQICILCF-STVLLTEANPI--------------GVCIRNCAQCKKMFGPYFEGQL
Photinus-EH1      -----MACSMKT----IFRIYFFCI-AALPFIEANPI--------------GICIRNCVQCKKMFGPYFEGQV
Aquatica-EH1      -----MSRTLKT----VLRLYLLCF-ATFLLTDANPI--------------GICIRNCVQCKKMFGPYFEGQL
Nicrophorus-EH1   -----MVSSRKFIVLTGAALLLLLVVSQSPVVEANPI--------------GICIRNCAQCKKMFGPYFEGQL
Aleochara-EH1     -----MGFLLRRGFEST-FCLVVLFAALVLQAQANPI----------GILHGVCIRNCAQCKKMFGPYFEGQL
Oryctes-EH-1      -------MAKTVYLG-----IFLSTLLLATFVNSSP------------LFTGICIKNCAQCKEMYGKYFYGQL
Coccinella-EH     ------------MLRKV--ILLVFICCMIVHLEAYNP------VTKLSRPIGICIRNCAQCKKMYGPYFDGEL
Harmonia-EH1      ------------MLRIT--VLFVFVCCMFVHLEAYKT------GSRNTKPIGICIRNCAICKKMYGPYFDGEL
Dendroctonus-EH1  -------MAEYQRIT---LRILMVVVLAASCIDANLI--------------GVCIRNCAQCKKMFGRYFEGQL
Hypothenemus-EH1  ------------MLP---TFILTVLMFSAVCIEANPI--------------GVCIRNCAQCKKMFGPYFEGQL
Anoplophora-EH1   -----MVCQRNKMLG---VVAFLLLASMVLLAEANPI--------------GVCIRNCAQCKKMFGPYFEGQL
Leptinotarsa-EH1  -----MEGGVKMYSG---VAVVLIFTCFVFCAEANPI-------------VGICITNCAQCKKMFGPYFEGQL
Aethina-EH1       -----MVRVAKNLLG---ASFLFLIASTILFADANPI--------------GVCIRNCAQCKKMFGPYFEGQL
Aethina-EH1       -----MVRVAKNLLG---ASFLFLIASTILFADANPI--------------GVCIRNCAQCKKMFGPYFEGQL
Hycleus-EH1       -----MVNTTRT----SLIICLLLINNYLILAGANPI--------------GVCIRNCAQCKKMFGPYFEGQL
Tenebrio-EH1      -----MACRTRNLLG---AALFVLLTSAFLLTEANPI--------------GVCIRNCAQCKKMFGPYFEGQL
Tribolium-EH1     -----MDSGSRN----FLVLLLLFA-SSLLVVDANPI--------------GVCIRNCAQCKKMFGPYFEGQL

Pogonus-EH2       -----MVNFQWK-KI--FFSVLIILLTNYICTC--------------SSSVAVCITNCVQCKQMFGQYFEGRA
Ignelater-EH2     ---------MNK-FC--GFLCLLILLWERSVVC--------------NGSLGVCITNCVQCKKMFGPYFQGKA
Photinus-EH2      MTNKDVGPGYNR-LY--GVLYVLVLLWKVPAMG--------------HCSFDICITNCVQCKKMFGPYFQGRV
Aquatica-EH2      --NCAVYFPIMN-VN--VFLCILVLVWKSSTVC--------------YCAFDICITNCVQCKKMFGPYFKARA
Nicrophorus-EH2   -----------M-QT--ILRSMLLIMWLSAYAV--------------EATIPVCLTNCVQCKQMFGRYFQGRA
Aleochara-EH2     -------------MT--SFISTLLLLQLVRVAS--------------TESIPVCLSNCIQCKKMFGRYFQGRI
Oryctes-EH-2      ---------E-K-FG--LYTCYLIFMLLFADFC--------------NASIPVCITNCVQCKQMFGVYFEGKA
Hypothenemus-EH2  ----------MR-IH--LGTLCLLVILMGKHFA--------------KSSIGICLTNCVQCKQMYGPHFEGKI
Anoplophora-EH2   ----------------------MIIVAIKEDLA--------------NASLAVCITNCVQCKQMFGPYFQGRA
Leptinotarsa-EH2  --------MK-C-LH--VFTLSIILVIIEKDLV--------------NASVEVCITNCVQCKQMFGPYFQGKA
Aethina-EH2       --------------------MVIILISNEYYLI--------------TASIPVCITNCVQCKKMYGPYFQGKI
Hycleus-EH2*      --------------------------------------------------------NCVQCKQMYGRYFQGRA
Tenebrio-EH2      ----------------------------------------------------TCLLNCVQCKQMFGPYFQGKA
Tribolium-EH2     -----------------MLKLLFILLAVDTFFS--------------NASIPVCITNCVQCKQMFGPYFQGRA

                 74
Pogonus-EH1      CADACVKFKGKLIPDCEDLSSISAFLNKFE---
Ignelater-EH1    CADACVKFKGKMIPDCEDITSIAPFLSKFE---
Photinus-EH1     CADACVKFKGKMIPDCEDITSIAPFLSKFD---
Aquatica-EH1     CADACVKFKGKMIPDCEDITSIAPFLNKFD---
Nicrophorus-EH1  CADACVKFKGKIVPDCEDIVSIAPFLTKFE---
Aleochara-EH1    CADACVKFKGKIIPDCEDIGSIAPFLTRFD---
Oryctes-EH-1     CADSCVKYKGSIIPNCEDIASIAPFLILNDDLS
Coccinella-EH    CGDACIKFKGKIIPDCDDIASVAPFLTRLD---
Harmonia-EH1     CGDACIKFKGKIIPDCDDISSVAPFLNKLD---
Dendroctonus-EH1 CADACVKFKGKIIPDCEDFASIAPFLSKFDNY-
Hypothenemus-EH1 CADACVKFKGKMIPDCEDIASIAPFLSKFDHY-
Anoplophora-EH1  CADACVKFKGKIIPDCEDIASIAPFLNKFD---
Leptinotarsa-EH1 CADTCVKFKGKIIPDCEDLASIVPFLNKFE---
Aethina-EH1      CADACVKFKGKIIPDCEDIASIAPFLSKFD---
Aethina-EH1      CADACVKFKGKIIPDCEDIASIAPFLSKFD---
Hycleus-EH1      CADACVKFKGKIIPDCEDIGSIAPFLSKFE---
Tenebrio-EH1     CADACVKFKGKIIPDCEDIASIAPFLNKFE---
Tribolium-EH1    CADACVKFKGKIIPDCEDITSIAPFLNKFE---

Pogonus-EH2      CGDACMATGGLVTPDCNNPLTLRSFLKRLY---
Ignelater-EH2    CGDACVLSKGLIKLDCNNPDTFLSFLKRFNY--
Photinus-EH2     CADACVSTNGKIILDCNIPETVVTFLKRFH---
Aquatica-EH2     CADACVQSDGLINLDCNIPETIVTFLKRLY---
Nicrophorus-EH2  CGEACISSNGRDMPDCNNPGTLGNYLKRLY---
Aleochara-EH2    CGNDCIISNGRAAPDCNNPGTIGHYLKRFKIYM
Oryctes-EH-2     CGDACISSNGQISPDCNNPGTLGIFLKRFH---
Hypothenemus-EH2 CGDTCVASYGRLIPDCNNPGTFNGYLKRLYRK-
Anoplophora-EH2  CGDACLASNGRVTPDCNNPGTLGSFLKRLY---
Leptinotarsa-EH2 CGDACLSNNGQLVPDCNNPGTLGNFLKRLY---
Aethina-EH2      CGEACIESDGRLTPDCNNPGTLGTYLKRLY---
Hycleus-EH2*     CGDACISSNGRVTPDCNNPITFGNFLKRYY---
Tenebrio-EH2     CGDACITSNGNLMPDCNNAGTLGNFLKRLY---
Tribolium-EH2    CGDACVSTNGRLVPDCNNAGTLGNFLKRLY---
```

Figure S8. Alignment of Coleoptera eclosion hormone sequences. The asterisk for the second *Hycleus* eclosion hormone indicates the conceptual translation of a pseudogene.

```
                   1
Pogonus            MTGKSTLVCVCATLCVAILVAEMVTAAPTYA-DYE-NSIRDLYELMLQKEA---LEERL---G-HQIVRKSSR
Ignelater          MQGYSALKCFCATLCVIIVVAEMVSAAPTYA-DYE-NSIRDLYELLLQKDA---LEERL---GAHQVVRKSSR
Nicrophorus        ---MLNIKYCCLFLLVLIVATEFTSAAPAYN-DYE-NSIRDLYELLLQKEA---IEERI---GAHQVARKSGR
Aleochara          MQGYPRMKIVCAVLCLIVVMAEVATAAPSYA-DYE-SSIRDLYELLMQKEA---LEERL---GAHQMVRKASR
Oryctes            MQKYSALRCLCAVLCVMVIVADMATAVPTYA-DYE-NSIRDLYELLLQKEA---LEDRL---GAHQVTRKSNR
Coccinella         MQFPVKMRYFFSISCIFLILAALANTAPAYG-DID-NNIRDLLDALYQRDM---LYDRF--NGVHQVVRKAGR
Harmonia           MCSQLKMKYICSIACIFFILAALANSAPAYG-DMN-NNIRELLDALYQRDM---LYDRF--NGIHQVERKAGR
Dendroctonus       MNINNAMKVFSLFVALMLFIASATSA-PSYSGEYE-SSIRELIEMLIQREKENGLEDSR--VGYHTVSRKAGR
Hypothenemus       MNLNSLMKVFAFLASLMLFMACVTAAPPSYPSEYDGASIRELVELLMQRERESGLDDTR--AAYHTVSRKAGR
Anoplophora        MNPSSAIKFFCVIFSILIALAVVSAA--PYSSDYD-NNIRDLFEILMQRDN---LEDRM---GLHQVVRKS-R
Anoplophora-s.v.   MNPSSAIKFFCVIFSILIALAVVSAA--PYSSDYD-NNIRDLFEILMQRDN---LEDRM---GLHQVVRKS-R
Leptinotarsa       MHTNTAVKFFCVIFSILITIAAVSPA--PYS-DYD-NINDLYELLLQQEK---LNDE-L---GRHQVERRA-R
Aethina            MQSVTMKKYLLAVVCIAVLAATISSAAPSYS-DYD-NNIRDLFEILMQRDG---VEDGR--LGLHQVVRKQSR
Hycleus            MQGVAIMKCLCAVTCIMVLFATVSTAPSSYA-DYD-SNIRDLWEMLLQREA---LDEKY--GGGHQLVRKSGR
Tenebrio           MQGYSAMKCLCAVTCIMIVVATVTSAAPSYA-DYD-NNIRDLWEMLLQKDA---LEDKF---GGHQMVRKSSR
Tribolium          MQRYSAMKCLCAVTCIMIVVATVTSAAPSYA-DYD-NNIRDLWEILLQKEA---MDDKFAPGGPHQMVRKSGR

                  74
Pogonus            SPSLRLRFGRRSDPSLLAG-------------------------PPFMLPQSGEPERLEN-----
Ignelater          SPSLRLRFGRRSDPSLLPA-------------------------SQYILAQEAEHGDN-------
Nicrophorus        SPSLRLRFGRRSDPSLIQA-------------------------SPYMLSAAQDAAEIAN-----
Aleochara          SPSLRLRFGRRSDPSLLPG-------------------------GPFMYQAAAAQEAAERAAGDN
Oryctes            TPSLRLRFGRRSDPNMLAS-------------------------PYLLAQESTDHSDN-------
Coccinella         SPSLRLRFGRRSDPELSPG-------------------------EAFLMAQQAEEN---------
Harmonia           SPSLRLRFGRRSDPELSPG-------------------------VAFLMAQQPDDN---------
Dendroctonus       SPQLRLRFGKRMDSQFPVP-------------------------AAYITGLNDITNDN-------
Hypothenemus       SPQLRLRFGKRLDTQYAVP-------------------------SAYMAALNDIPTEK-------
Anoplophora        GPQLRLRFGKRPDAGYEP-------------------------ISAYMSGISADGEN--------
Anoplophora-s.v.   GPQLRLRFGKRPDAGYEPV-----VKRPRSLRLRFGKRSDDLPISAYMSGISADGEN--------
Leptinotarsa       GPQLRLRFGKRAGS-FDPVMKPIPVERAPSLRLRFGKRSEESPISDYLSGLSSEGAN--------
Aethina            SPQLRLRFGKRQDPSLLPV-------------------------SAYMMGEQDN-----------
Hycleus            SPSLRLRFGRRSDPSMTPE-------------------------AAFMMAQAIDHNNN-------
Tenebrio           SPSLRLRFGRRSDSSMSPE-------------------------AAFMMAQAVDHDN--------
Tribolium          SPSLRLRFGRRSDASMTPE-------------------------AAFMMAQAVDHETN-------
```

Figure S9. Alignment of Coleoptera sNPF precursors. Note that these sequences are well conserved. The apparent exceptions of *Leptinotarsa* and *Anoplophora* are due to the appearance of an extra exon. *Anoplophora* s.v., *Anoplophora* splice variant.

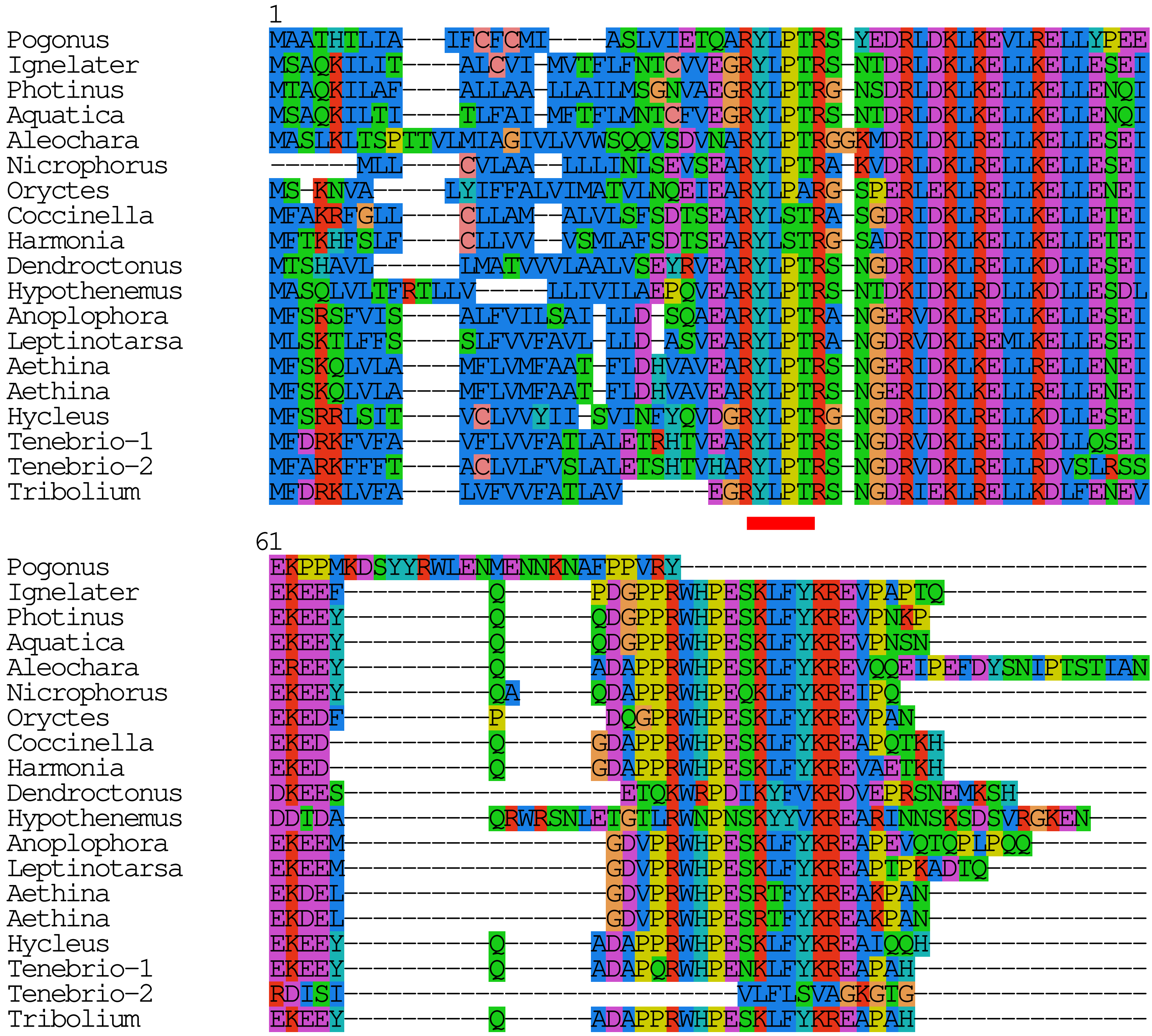

Figure S10. Alignment of proctolin precursors.

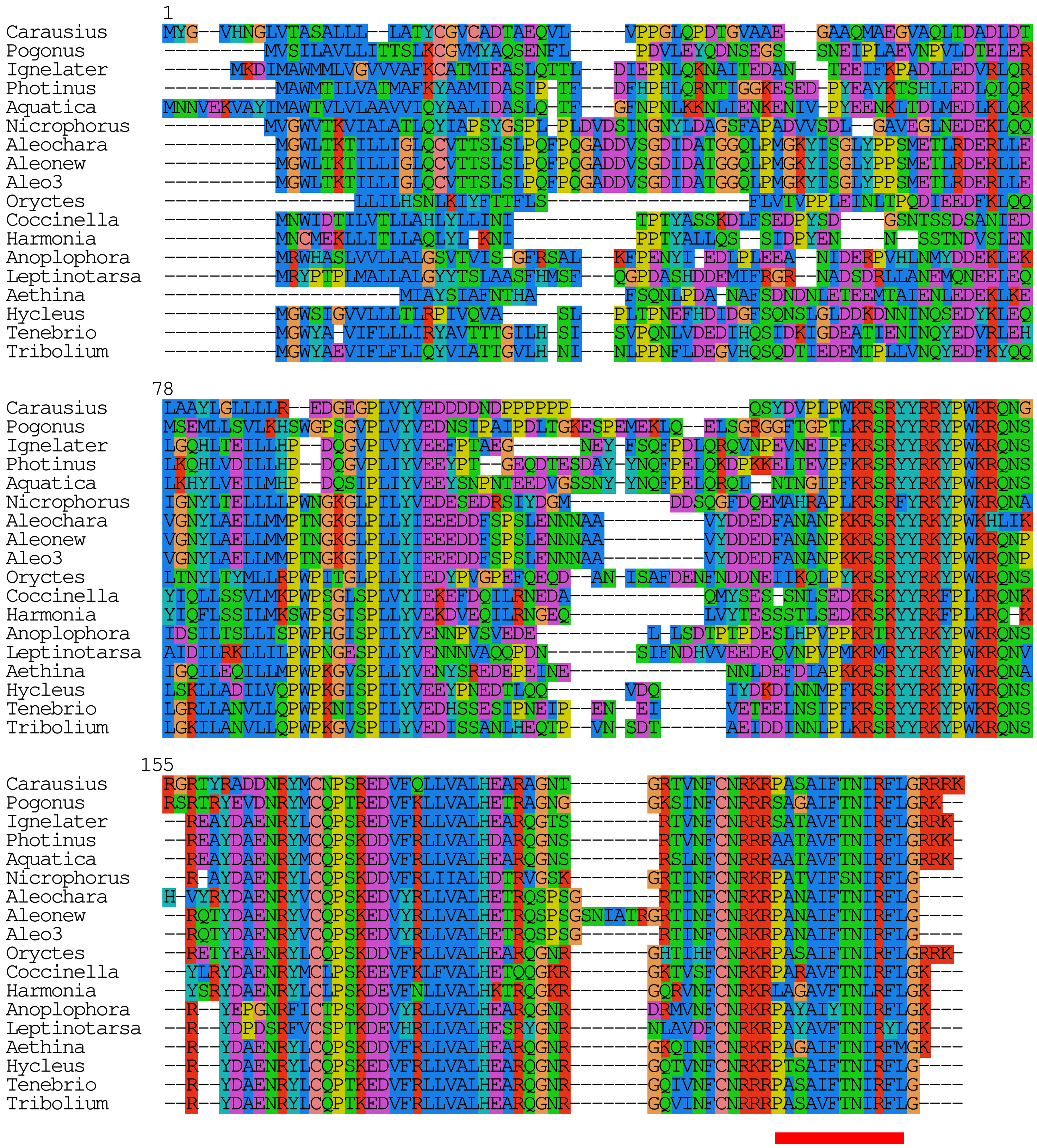

Figure S11. Alignment of RFLamide precursors.

```
              1
                                                                       *     *
Oryctes-2     -MK---YITLLIFIYFFIYLWRTALILMLLEGRSLDLLYHN----AIAEASSPTNECRLTPAIR
Oryctes-1     MFKLLFKSVNIEVLLVTLLAWKIAIIFISLDTICTDSKFKS----LQVQAVGSLDECQLTPVIH
Aethina       -------MLRQILDIVPQRLWQITTIFIILGNMCMDPRLSRG--TLEVSGTSTTDECQVTPVIH
Leptinotarsa  -------MFSRSCVWVISKLWYVTLVFIILGNMCMDPRLKTA--TSQVEAASTTDECQVTPVIH
Aquatica      -------MFTNIFQI---KTWF-TILLIMISSMCMLPSKN----KMITEAASTADECQVTPVIH
Ignelater     -------MFTQIFQSATQQLWTTTVLLTILANMCFDSRLSSLNNKIAVEGASSADECQVTPVIH
Photinus      -------MFGK-FVQIRMQTWLILVAVTLIANMCMSSSKV----KTRVAAANAADECQVTPVIH
Pogonus       -----MTHLGF-----SYSFEFCATIIFLLASMCMDPRFSKS---SAVDASSSGDECQVTPVIH
Anoplophora   -------MLPQVANLVLTKVWKLGTLFVIIATMSMDPRLTNIK-NLQAEAASSVDECQVTPVIH
Aleochara     -------MIGDFQFSKFVCLLQYAFLLIVLSGMCLDPKYAK--------GMISTDECQVTPVIH
Dendroctonus  -----MVVLENVCRLVGSALWNSTLVLIFIASMCMDPRLSAA-PKISAEAASLTDECQVTPVIH
Hypothenemus  -----MVLLVKVSNLVYPKIWNQTLVLVLIASMCMDPRLNSN-SK-PVEAASVTDECQVTPVIH
Nicrophorus   -------MYGNILFKVKSFMEKSLIVLIIIGNMCMDPHFQG---KTLVQAANAADECQVTPVIH
Coccinella    -------MIIAAIIHLSCNILTKSIMFMIIGSMCIDPRYIQ----YEVSAASKTDLCEVTPVIH
Harmonia      ------------------NIFCKAIMFLLIGSMCIDPRYIK----YEVAAASKTDLCEVTPVIH
Hycleus       --------MNSPTRCLFQNLWKIVLLL-IINGTCIDPRLN------KVSAASSTDECQVTPVIH
Tenebrio      --------MKSLITLVPWKLWRLTLLFAVLSSMCLDPRLTSN---IKATAASTTDECQVTPVIH
Tribolium     --------MKPLLSLV--TLWKLTI---FLSSMCLDPRLNSK---IQVSGASTTDECQVTPVIH

            65*** *  **** ** *     *             *                * * *  ****
Oryctes-2     RIKYEGCSSNLIHTFGCTGRCSSYLQVSGSKIWQLIRSCMCCQESGEREASVIINCINPVSEYK
Oryctes-1     VLQYPGCIPKPIPSFACMGRCASYLQVSGSKIWQMERSCMCCQESGEREASVSIFCPQAKPGEK
Aethina       VLQYPGCVPKPIPSFACIGRCASYIQVSGSKIWQMERSCMCCQESGEREASVSLFCPKAKPGER
Leptinotarsa  VLQYPGCVPKPIPSFACIGRCASYIQVSGSKIWQMERSCMCCQESGEREASVSLFCPKAKPGER
Aquatica      VLQYPGCVPKPIPSFACTGQCASYIQVSGSKIWQVERSCMCCQESGEREASVSLFCPKAKPGER
Ignelater     VLQYPGCVPKPIPSFACTGRCASYIQVSGSKIWQMERSCMCCQESGEREASVSLFCPKAKPGER
Photinus      VLQYPGCVPKPIPSFACTGRCASYIQVSGSKIWQMERSCMCCQESGEREASVSLFCPKAKPGER
Pogonus       VLQYPGCVPKPIPSFACIGRCASYLQVSGSKIWQMERSCMCCQESGEREASVSLFCPKAKPGER
Anoplophora   VLQYPGCVPKPIPSFACIGRCASYLQVSGSKIWQMERSCMCCQESGEREASVSLFCPKAKPGER
Aleochara     VLQYPGCVPKPIPSFACIGRCASYLQVSGSKIWQMERSCMCCQESGEREASVSLFCPKAKPGEK
Dendroctonus  VLQYPGCVPKPIPSFACTGRCASYIQVSGSKIWQMERSCMCCQESGEREASVSLFCPKAKPGER
Hypothenemus  VLQYPGCVPKPIPSFACTGRCASYLQVSGSKIWQMERSCMCCQESGEREASVSLFCPKAKPGER
Nicrophorus   VLQYPGCVPKPIPSFACIGRCASYLQVSGSKIWQMERSCMCCQESGEREASVSLFCPKAKPGEK
Coccinella    VLQYPGCVPKPIPSFACIGRCGSYLQVSGSKIWQMERSCMCCQESGEREASVSLFCPKAKPGER
Harmonia      VLQYPGCVPKPIPSFACIGRCGSYIQVSGSKIWQMERSCMCCQESGEREASVSLFCPKAKPGER
Hycleus       VLQYPGCVPKPIPSFACTGRCASYIQVSGSKIWQMERSCMCCQESGEREASVSLFCPKAKPGER
Tenebrio      VLQYPGCVPKPIPSFACIGRCASYIQVSGSKIWQMERSCMCCQESGEREASVSLFCPKAKPGER
Tribolium     VLQYPGCVPKPIPSFACIGRCASYIQVSGSKIWQMERSCMCCQESGEREASVSLFCPKAKPGER

           129  *   *    *       *  * *           * *      *
Oryctes-2     RFQKVTIKAPLSCMCRPCNDVEKHNVIPQELAGYSEKSRLSGHFLL----
Oryctes-1     KFRKVTTKAPLDCMCRPCTGIEESAVIPQEIAGYTDEGPLSNHFLKSTTQ
Aethina       KFIKVTTKAPLECMCRPCTGVEESAIIPQEIAGYADEGPLSNHFLKSHQQ
Leptinotarsa  KFIKVTTKAPLECMCRPCTGVEESAVIPQEIAGYSDETPLSGHFLKSHQL
Aquatica      KFKKVTTKAPLECMCRPCTGVEESAVIPQEIAGYTDEGQFSNHFIKSQTQ
Ignelater     KFKKVTTKAPLECMCRPCTGVEESAVIPQELVGYADEGPLSNHFLKSQTQ
Photinus      KFRKVTTKAPLECMCRPCTGVEESAVIPQEIAGYADEGPLSNHFLKSQSQ
Pogonus       KFRKVSTKAPLECMCRPCTGVEESAVIPQEIANYGEESPLSHHFRKSQNQ
Anoplophora   KFIKVTTKAPLECMCRPCTGVEESAVIPQEMAGYSDE-ALSGHFLKSHQL
Aleochara     KFRKVSTKAPLECMCRPCTGVEESAVIPQEIAGYTDEGPLSNHFLKSHVQ
Dendroctonus  KFIKVTTKAPLDCMCRPCTGIEESAIIPQEIAGYTDEGPLSGHFMRSHVQ
Hypothenemus  KFIKVTTKAPLDCMCRPCTGVEESAVIPQEIAGYTDEGPLSGHFMRSHIQ
Nicrophorus   KFRKVTTKAPLECMCRPCTGVEESAIIPQEIAGYADEGPLSNHFLKSHAH
Coccinella    KFLKVTTKAPLNCMCRPCTSIEESAVIPQEIAGYADEGPLSDHFLKSQPQ
Harmonia      KFIKVTTKAPLSCMCRPCTSIEESAVIPQEIAGYADEGPLSDHFLKSTPQ
Hycleus       KFIKVTTKAPLECMCRPCTGVEESAIIPQEIAGYADEGPLSNHFLKSHSQ
Tenebrio      KFIKVTTKAPLECMCRPCTGVEESAVIPQEIAGYADEGPLSNHFLKSHSQ
Tribolium     KFIKVTTKAPLECMCRPCTGVEESAVIPQEIAGYADEGPLSNHFLKSHSQ
```

Figure S12. Sequence comparison of Coleoptera bursicon A sequences. Note that the first sequence, that of the second Oryctes bursicon A has a large number of amino acid residues that are different from the consensus sequence; those are indicated by the asteriks on top of the sequences.

```
             1
Pogonus      --MARIL-WQLILVLV---SILFIVRGQ-LSGDETCETLPSEIHLIKEEYDELGRLQRTCNGEV
Ignelater    MMGFISIFLTTLIAIT---THYSVQCLN-ELGEENCETLPSEINIIKEEYDELGRLQRTCHGEI
Photinus     -MDAVAIFSKLFLTII---TISRIHTMN-DFGEETCETLPSEINIIKEEYDELGRLQRTCHGEI
Aquatica     -MGFAHNFSKLFLTII---TILHVHSLS-DISEENCETLPSEINIIKEEYDELGRLQRTCHGEI
Nicrophorus  -MGIA-RLFFCCF--ILNLYIYTVNSLNEIPGEENCETLPSDIHLIKEEYDELGRLQRTCNGEV
Aleochara    -MATS-RVHTLCLRLLFAILFASIAALNEIPGEENCETLPSEIHLTKEEYDELGRLQRTCNGEV
Oryctes      -MGNTRTYFNVLVIVI--ISLYKANGYSNIPGEETCETLQSEINLLKEEFDELGRLQRSCSGEI
Coccinella   -MALACTRIGLSFFLLY--TIFTLSYCKPDMPEETCETIPAEIHLVKEEYDELGHLQRTCSGDV
Harmonia     -----MAVVRFDIFLLF--AILCVGF-GKEMPEETCETIPNELHLVKEEYDELGHLQRTCSGDV
Dendroctonus ----MFTKGLLLIFAI---FASYGYCVS-ELPDESCETLPSDIHITKEEFDELGRLQRTCNGEV
Hypothenemus ----MSSKEFLVFLVL---ISTYVYCVS-ELTEETCETLPSEIHITKEEFDELGRLQRTCNGEI
Anoplophora  ------MLYTFLVSLL---CFSYIYCVS-DMSDETCETLPSEIHLTKEEYDELGRLQRTCNGEV
Leptinotarsa -----MLFYTVFFGAM---AVSFVYCLN-DMSEETCETLPSEIHLTKEEYDELGRLQRTCNGEV
Aethina      --MAITVFHRIVFACM---MFYYVCCVS-DLAEETCETIPSDLHLTKEEYDELGRLQRTCNGEV
Hycleus      -----MYNYSVFFLIL---YCTYVSSLS-DLSEETCETLPSDVNLIKEEYDELGRLQRTCNGEI
Tenebrio     -----MYN-KILLCLV---YVSYVSSVS-EISEETCETLMSDINLIKEEFDELGRLQRICNGEV
Tribolium    -----MFD-KIILCLV---YATCVYSVS-EISEETCETLMSDINLIKEEFDELGRLQRICNGEV

            65
Pogonus      AVNKCEGACNSQVQPSVITPTGFLKECYCCRESFLRERTVTLTHCYDPDGMRLTNEDMHSMDIK
Ignelater    AVNKCEGTCNSQVQPSVITPTGFLKECYCCRESFLRERVVTLSHCYDPDGMRLTNEDMHSMDIK
Photinus     AVNKCEGSCNSQVQPSVITPTGFLKTCYCCRESFLRERVVTLSHCYDPDGMRLNAEELKAMDIK
Aquatica     AVNKCEGMCNSQVQPSMITPTGFLKECYCCKESFLRERVVTLPHCYDPDGLRLMTADMHTMDIK
Nicrophorus  AVNKCEGTCNSQVQPSVITPTGFLKECYCCRESYLRERIITLTHCYDPDGIRLIGEGKQTMDIK
Aleochara    AVNKCEGTCNSQVQPSVITPTGFLKECYCCRESYLRERTITLTHCYDPDGMRLTSDAMKQMDIK
Oryctes      AVNKCEGSCNSQVQPSVITPTGFLKDCYCCRESYLKERVITLTHCYNPDGIRLTENDMQTMDIK
Coccinella   AVNKCEGSCRSQVQPSVITPTGFLKECYCCRESFMRERIITLNHCYDIDGVKLIDEGNSSLDVK
Harmonia     AVNKCEGSCRSQVQPSVITPTGFLKECYCCRESFMRERIITLNHCYDIDGVKLVEEGTSSLDVK
Dendroctonus AVNKCEGSCKSQVQPSVITPTGFLKECYCCRESYLRERIVTLTHCYDPDGLRLIGEGENSMDIK
Hypothenemus SVNKCEGSCKSQVQPSVITPTGFLKECYCCRESYLRERIITLTHCYDPDGVRLAAEGENSMDVK
Anoplophora  AVNKCEGSCKSQVQPSVITPTGFLKECYCCRESFLRERIITLSHCYDPDGVRLTNEGTNSMDVK
Leptinotarsa AVNKCEGSCKSQVQPSVITPTGFLKECYCCRESFLRERIITLSHCYDPDGVRLTGEGDNAMDIK
Aethina      AVNKCEGSCKSQVQPSVITPTGFLKECYCCRESYLRERIITLTHCYDPDGVRLTGEHNNAMDIK
Hycleus      AVNKCEGSCKSQVQPSVITPTGFLKECYCCRESFLRERIITLSHCYDPDGVRLTAAGVNSMDVK
Tenebrio     AVNKCEGSCKSQVQPSVITPTGFLKECYCCRESFLRERTITLTHCYDPDGVRLTTENANSMDVK
Tribolium    AVNKCEGSCKSQVQPSVITPTGFLKECYCCRESFLRERTITLTHCYDPDGVRLTAETVNSMDVK

           129
Pogonus      LREPSDCKCFKCGDFSR
Ignelater    LKEPSDCKCFKCGDFSR
Photinus     LREPSDCKCFKCGDFSR
Aquatica     LREPSDCKCFKCGDFSR
Nicrophorus  LKEPAECKCYKCGDFSR
Aleochara    LKEPAECKCYKCGDFSR
Oryctes      LREPIECKCSKCGDFSR
Coccinella   LKEPAECKCYKCGDLGR
Harmonia     LKEPAECKCFKCGDLGR
Dendroctonus LREPSECKCYKCGDFSR
Hypothenemus LREPSECKCYKCGDFSR
Anoplophora  LREPSDCKCYKCGDFSR
Leptinotarsa LREPSECKCFKCGDFSR
Aethina      LREPTECKCYKCGDFSR
Hycleus      LREPAECKCYKCGDFSR
Tenebrio     LREPAECKCYKCGDFSR
Tribolium    LREPAECKCYKCGDFSR
```

Figure S13. Sequence comparison of Coleoptera bursicon B sequences.

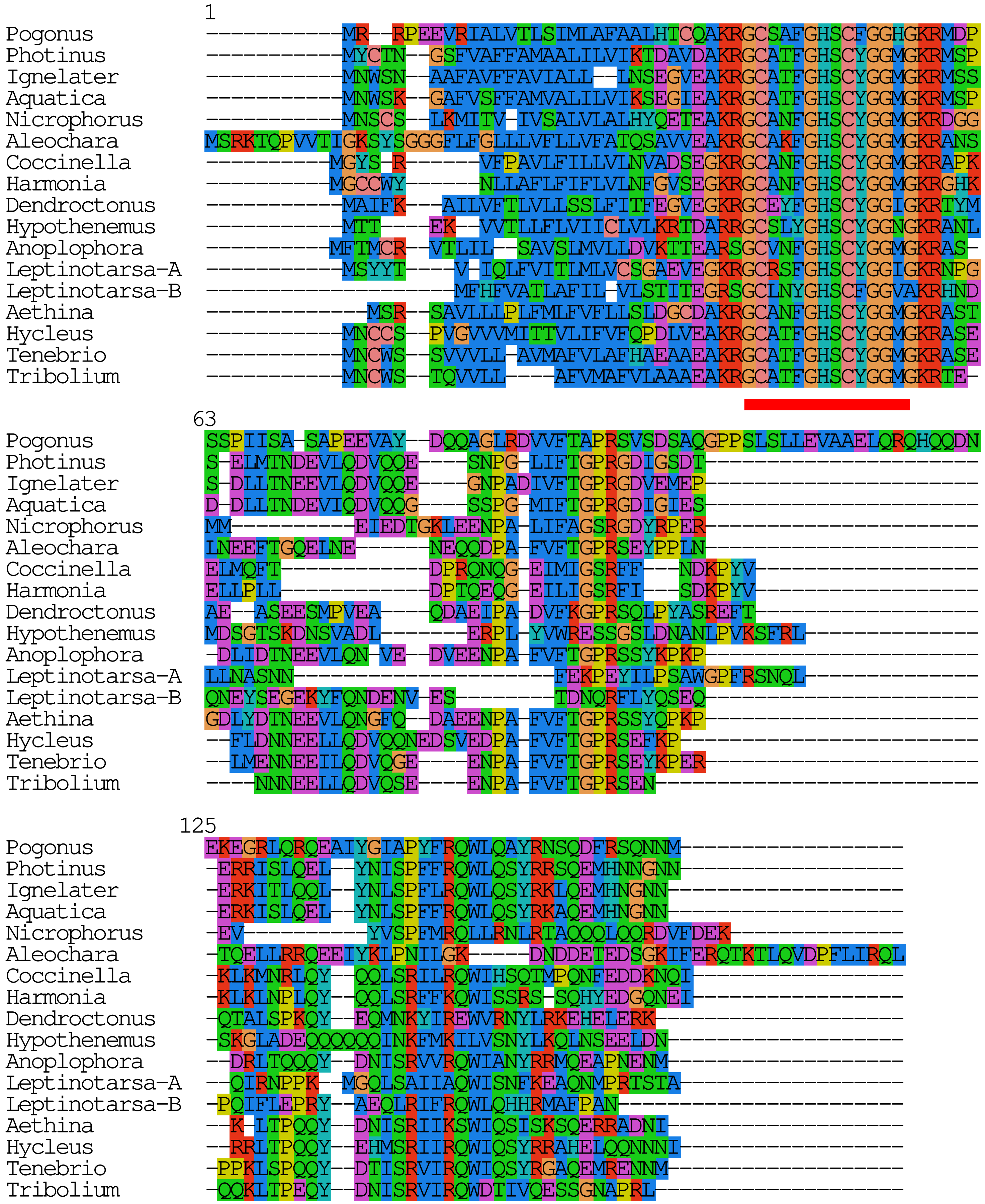

Figure S14. Alignment of CCHamide-2 precursors.

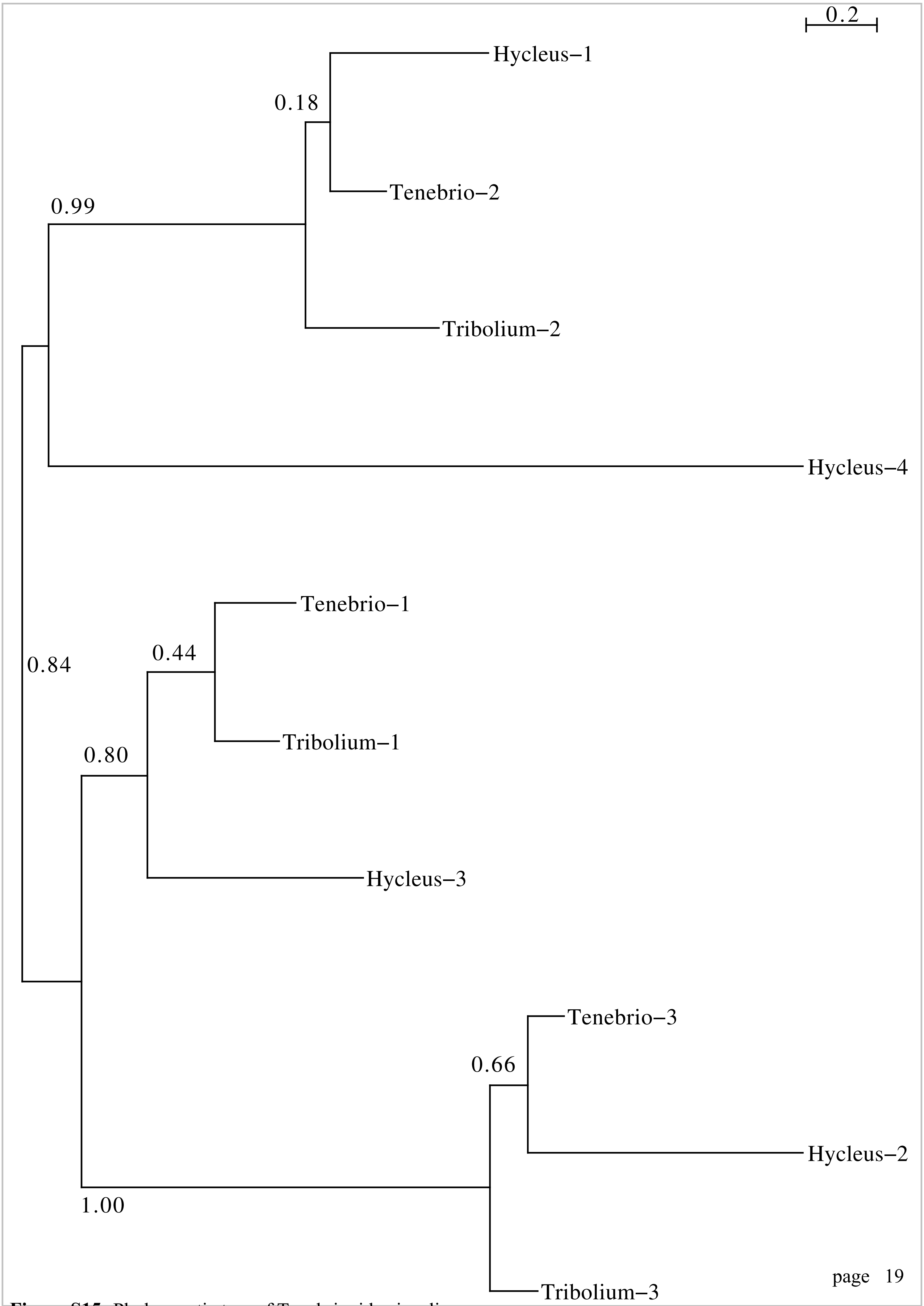

Figure S15. Phylogenetic tree of Tenebrionidea insulins.

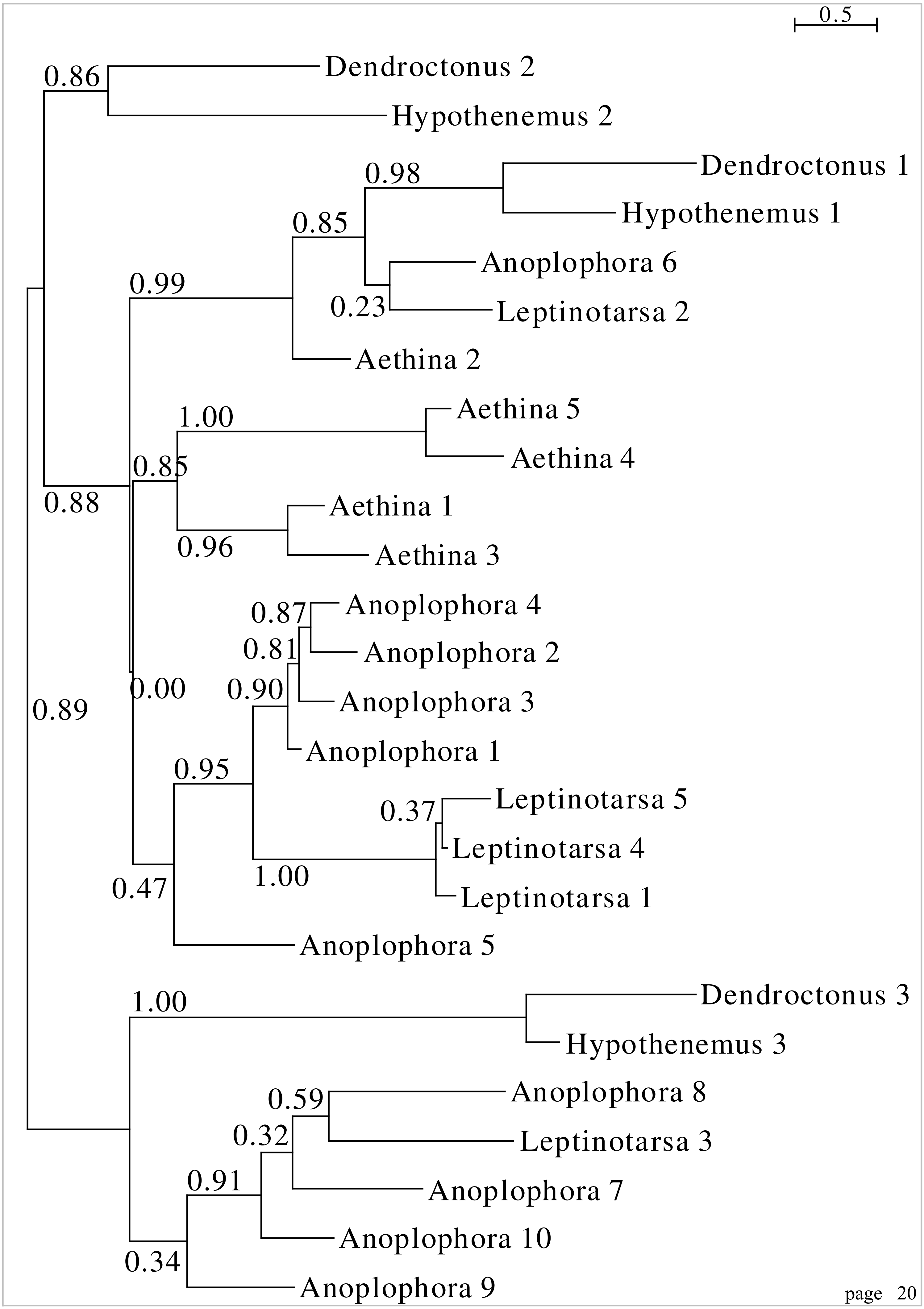

Figure S16. Phylogenetic tree of insulins from *Dendroctonus, Hypothenemus, Anoplophora, Leptinotarsa, Aethina*

```
             1
Pogonus      ----MHSTVLLYAFIG--VVFTTTLCNLGEAHVLSLHCTPCLNDFENGCDDPPA-EICVFGEVR
Ignelater    ----MRATPILPLLF---ALVVTTFSYMSD---ARSLCTPCFT--EEECDKEPE-HFCMWGVAR
Photinus     ----MWVSSIVSLSV-----ILITFTHMTD---ARWVCNPCAT--EDECEREPI-EFCMWGEAR
Aquatica     ----MWGSSLLPLTF---VFIILTFFYSTD---ARWVCNPCVT--EDECEREPI-EFCMWGEAR
Nicrophorus  ----MQTISIISLLFAVT---LSFVITNMAEARAPWICQPCDM--FEECNKEPE-SYCMWGETR
Aleochara    MRSAPPPLSCV-LLVAATICYVSLFGVTSVDARAAWTCQPCVS--AEDCNTEPV-ELCLWGETR
Oryctes-1    ----MDSTMAFIIIGF--ILSSNLV-YL-SSARRFHFCTPCMS--LEDCNQEPN-EVCQWGESR
Oryctes-2    ------------MRKF--IMIVSML-YNTGFGRRNYECRPCMS--MQECNEEPS-DYCPFGETR
Coccinella   ----MKYFSSLLLIIT--IVIL------SDYVQWSAACRWCQG---EECNVAPP-TACPFGEYI
Harmonia     ----MRFFPSILLIVA--IFIL------SDYVRWSTACTWCQG---EECNAPPPQYPCPYGEYT
Dendroctonus --MNPSTGIILVLCIA-LTLASSIDAMSIT---ERWICTSCVT--QQECDQDPP-SVCIWGEAR
Hypothenemus ----MHSSLVLMLVFT-LTFIVSVHSMSIT---ESWVCKPCAT--QQECDEKPN-NICVWGEAR
Anoplophora  ----MRSTLVTLLLAV-AIALILLCDLSLA---GKWVCRPCDS--QAECDEDPP-EYCVWGEVR
Leptinotarsa ----MSSTLVYVVLAL-TVFFILCPGTSIA---GLWICKPCEN--QAECDSQPK-EYCVWGEVR
Aethina      ----MYRFPLVSIILT-VAITLSVFHEASCGSISIKPCRPCI----NDCDDNPP-DECPWGESR
Hycleus      ----MHTSPQILAAILIVVVSFAVFTEQY-NPYRILPCRRCQT--AAECEEEPP-QICMWGEGR
Tenebrio     ----MCPSYNFATIVLVLTITVIIFSDKG-TTM-SLPCRRCFT--SDECNSPPP-DFCPYGENK
Tribolium    ----MCPFHNFITIILVLTVTVIIFSDKG-TAMIHLPCKRCAT--IQECNADPP-QLCVFGENR

            65
Pogonus      DKCGRRVCGKGPGEKCG----GHFGVCGEGMSCTTSFLGANVPRCHGCTLY--ANMECFPVL--
Ignelater    DSCNKKVCAKGPGERCG-GPLGVLGQCGAGMMCYS-D-----EICHGCSS---KTFECYP----
Photinus     DSCNRRVCAKGPGERCG-GPLGILGQCGDGMMCKK-D-----ERCHGCST---QTFQCHP----
Aquatica     DSCNRRVCAKGPGERCG-GPLGILGQCGEGMMCKT-D-----ERCHGCST---QTFQCHP----
Nicrophorus  NSCNRRVCAKGPGERCG-GLRDIFGRCGTGMWCQKED-ESNTYTCHGCYD---NSLECHQSTRY
Aleochara    NFCGRRVCAKGPGERCG-GMKDVLGKCGAGMWCQREEMDSMTFTCHGCYD---NSLDCHRDRRY
Oryctes-1    DKCGRRVCAKGPSERCG-GPMNIYGNCGEGLRCDS-D-----ERCHGCIAVRDSNFVCHPSDDY
Oryctes-2    DMCGRRTCAKGPADRCG-GPNGIYGRCAEGLRCRS-D-----ERCHGCADKGNSDFECYPKF--
Coccinella   NRCRRRACLKGPGEKCGGPTRNSFGDCATGLYCAS-E-----GRCYGCFL---GNLDCYPPQPE
Harmonia     NRCRRRECLKGPGEKCGGPIRNSFGDCATGLFCSS-E-----GRCYGCFL---GNLECYPPQPN
Dendroctonus NNCGRRICAKGPGERCG-GSLDILGICGEGMMCKS-D-----ERCHGCSM---QTLECSHN---
Hypothenemus DACGRRICAKGPSERCG-GSLNVLGVCGEGMMCKA-D-----EHCHGCSI---QTMECSHN---
Anoplophora  DACNRRMCAKGPGERCG-GTLNILGQCGEGLMCKS-D-----EICHGCSI---QTMKCYND---
Leptinotarsa DACNRRACAKGPGERCG-GSLNILGQCGEGMMCKS-D-----EKCHGCSL---QTMQCYNE---
Aethina      DDCGRRVCSKGPGERCG-GYSNAFGSCGEGLTCRPYD-----EKCHGCSL---ITLDCF-----
Hycleus      DACNRRVCLKGPGEKCG-GKFRIMGTCGDGMMCANSD-----SRCHGCYI---PTMTCYPEITD
Tenebrio     NYCGRRVCSKGPGEKCSNDQYAILGTCGEGMWCSNKD-----NRCHGCFI---ATMTCYE----
Tribolium    DYCNRRVCSKGPGEKCG-DRFNILGTCGEGLWCSNKD-----NRCHGCYI---PTMACYPDD--

           129
Pogonus      ------
Ignelater    ------
Photinus     ------
Aquatica     ------
Nicrophorus  ------
Aleochara    A-----
Oryctes-1    ------
Oryctes-2    ------
Coccinella   RKITQY
Harmonia     KNVNQY
Dendroctonus ------
Hypothenemus ------
Anoplophora  ------
Leptinotarsa ------
Aethina      ------
Hycleus      ------
Tenebrio     ------
Tribolium    ------
```

Figure S17. Alignment of Neuroparsin precursors.

```
Pogonus          ----MNNQQ-------VISSLVFVVLIVGINAGLEKLD---SNT-KISYDM---------VGHRL---KR--ST-
Ignelater-1      ----MPSYRLECCAAFIVISCVITAAIAEDHNENDKLA---NTAIPQKYQWYV--------SPRLGRKKKHVSPF
Ignelater-2      ----MGRSGLECCAVFLIVSCVFALIIAEEHNGNDQLT---NTAVSRDLQWYVRRKPGVWLTPRVGRKKRSASPY
Ignelater-3      MSTSSRLYKNVLTFRSVHKNHDIIVAIAEDHNENDKLA---NTATPQKYQWYV--------SPRLGRKKKNVSPL
Photinus-1       ----MGQFCLKCCVLLT---FLVVVTIANQYNGNHHI--------LMAVQLYSNKKPTVLLSSNAQRQKRN-SQY
Photinus-2       ----MKQPCATCTVLVAIVI----MAVSQVHTEVS----------------------------------------
Photinus-3       ----MKQFYATCAILLTVVS----MAIA-----------------------------------------------
Aquatica         ----MGQSSLKCCAVIFVLNLLFAITTADQSNGDHRK--------VIGLQWYVNKKPNTVLSSHILRKKRN-SNY
Nicrophorus-1    ---------MVRQAIFVLAVLILV---AICHSA-------D-NDERSIDSYSKRKMSALWFGPRLGRKKRNPSS-
Nicrophorus-2    ----MHRQVIVLSVLLVSALLISA-----A---------------------------------------------
Nicrophorus-3    ----MHRQAIVLSVLLVSALLTSA-----A---------------------------------------------
Oryctes-1        ----MEHLPVVSCVLLLLCSICVFTEQA----------------NQADTRLKLHTASPFWFGPRLGRNKRNPFS-
Oryctes-2        ----MDRSLYTFCALLLIFYTVIA---D-----------SSNEKTVDEYAKRKQISPLWFGPRLGRSKRNQHF-
Aleochara        ----MDRLVLFICVLLITIQTISS---------S------DQQEATGVDGQTNKKYARIWFEPRMGRRKRHPT--
Harmonia         ----MNRFIWFNCILLLFNTFDGSAVSL-NDYE--------LVEFRKPHHKKHYPAFYSWEGSTMK EKREPID-
Coccinella       ----MERFVWFNCVLLLLYTIDGSAVSL-NDYE--------LVEFHKPYHKRYYPSFYSWEGANIK-DKAEPIE-
Dendroctonus-1   ----MIRISVLNSFLLLIAVLYYATDSVDS--------------FDPAGDHERKESSPMWFGPRIGRKKRNPKD-
Dendroctonus-2   ----MGFSVTVICVAIFFLILRANPSASIEHGNNINGDPSETEINSRGSSLSQENYSPAWMDRKFGKQKINDLL-
Hypothenemus-1   ----MSNISVFNCFVLLAILLYYTTEIAES--------------LDAPN--DRKTSQAMWFGPRIGRKKRNPTD-
Hypothenemus-2   -------MLRVTCIILVFTTVISMS----STFVL--------SNNIESDATNTAVEKFPWINSRKS------KT-
Anoplophora-1    ----MAKVTLLSFMVFLTLALYFNGVLTA----N-------SDELIDAKRRDGEKKYSPLWFGPRIGRRKRNPTE-
Anoplophora-2    ----MDRIIIVACASLIILNIYSHMVLATHDYDS--------SLYSSKYREIDSKAAPLWFSSNLGRRKRNLKN-
Leptinotarsa-1   ----MSKLSVFTFAAIFIMLYFKSVASA---------------EVDEKRKDEAAKYSPFWFGPRIGRRKRNLID-
Leptinotarsa-2   ----MDRIVLVSCVVILIGNFCFELILASQRFDG--------SVETKMKEEEESKMSPAWFSPRLGRRKRNSKE-
Aethina-1        ----MKQSTKINFALIFMSLIILEICCAESHHGN------LSADKPEHEKHA----SELWHGPKLGRKKRNPSD-
Aethina-2        ----MTRFTLVNCAVLILIYLELAL----CSQPT------NVVAALDKQRDDEVKLDNLWFGPRLGRRKRNDSE-
Aethina-3        ----MRQSTRINCALVFISLIVLEVYCTGSHNYE------NLPDKSGKKTGYEKYISHLWSGARLGRKKRNLDD-
Aethina-4        ----MKQLTRINVALIFMSLVVLEVCCTESQHGN------LSADKSEKKTYH--DKHASQSLPKLGRKKRNPSD-
Aethina-5        ----MSQLAL TCTILMA--MCLHIVLAAPSN-----------D VTTDKRNQKTANQMWFGPRLGRRKRTPTP-
Hycleus          ----MDRINLVNCAVLCIIVLYFGIVLAVPHYE-------SAQD-------EGGDKENFWFGPRLGRKKRNQSN-
Tenebrio         ----MERIILVNLAVLCVAILLSEVVLSVPHYG-------SHQVSVRRERNDDKKQSYMWFGPRLGRKKRNSN--
Tribolium        ----MERFILINWTVLCVAVLFFETVLSTPHE-----------SSVPNERNDDSKETYFWFGPRLGRKKRNSSN-

76
Pogonus          NEDITPGLYYNKDDLHAIAELIQENP-WILANIPD----------MQK-------RQMYFTPRLGRDLSEENSNS
Ignelater-1      EE TYELSSLDKEQLESLLGNIQEPPEWTAYTISD----------GKR-------N SNFVPRLGREIIEIAEPD
Ignelater-2      EENNYRTPSFDREQLESLMDTVQEPP-WTLVTINE----------GKR-------HTVNFTPRLGRESGEENEPD
Ignelater-3      EE THELSSLNKEQLESLLDNIQEPPGWTAYTISD----------GKP-------N SNFVPRLGRELIEETDPD
Photinus-1       EDNYRIPAPLDKEQLEALIEAVEEPP-WTVLTFNE----------GKR-------HTVNFTPRLGRESGEEEAE-
Photinus-2       ---KLMNFPADNEQVETMIEATQEHP-WAIFTFGG----------KKR--------SVSFIPRLGRELGDENME-
Photinus-3       ------------------------DE-RNEFFIGG----------KKR--------GVSFIPRLGRELGDDNAE-
Aquatica         DDAFTISSPLEKEQMEAFLKALQEPP-WT LTFNE----------GKR-------HTVNFTPRLGRESSEEENE-
Nicrophorus-1    DEL-LKNTNLDREQLVALLEMLQESP-WAVVALNE----------GKRH------NKMNFTPRLGRESGEDLAGS
Nicrophorus-2    --------------------FDGQND-YR-SMFN---K-------GKP-------SIYDFVPRLGRESGEDMNAY
Nicrophorus-3    --------------------IDGRND-YQ-SMFT---K-------GKP-------SIYDFVPRLGRDSGEDVNAY
Oryctes-1        PDT-YSLSILTCEDVEE---YVKVIP-WDLVTKCMISE-------GKRDI---KSRMNAYIPRLGRESGEEFISS
Oryctes-2        DDL-EILLASECDEIDEYIRSIPQSM-FERCSINEDKR-------DKP-------KSKQFSPRLGRDSYEDYFGM
Aleochara        MEY-YKTPFMDHQQLETFLEFMQQSP-WADFTLPPNEN-------AKR-------YDVNFVPRLGRESGEEMGNA
Harmonia         DEY-LKFPDLDRK---------SFSS-WLLR-MND--------------------AGRIFPSRINRESNDNELSF
Coccinella       DEY-VKYSDLDRK---------SFSS-WLLR-MND--------------------AGRIFPNRINRESNENELSF
Dendroctonus-1   NEI-FIN--SPDQPTVNLLDMLRETP-MVVLAFSEA----------F--------KQHNFVPRLGRESGESLNGY
Dendroctonus-2   ---------RDKNKMVHILETLDEYL-CSIFTGNDG--------------MQKRDGHLYYTPRLGRNVEVVDNAR
Hypothenemus-1   PKT-YIN--DKNQPSMDLYEALRESP-LVVVAVNEA----------N--------KQHNFTPRLGRESGETLPNW
Hypothenemus-2   DVVWF----TGKIKVSKLLEYLQEYP-WTVIALDSRSG------------IEKRSNSVNFTPRLGRNLDEEGYSA
Anoplophora-1    NIF-KT---LDREDLDA---YALKNP-WPLMTFNN----------GKR--------QMDFTPRLGRDSMEDQEER
Anoplophora-2    SV--------TQHELIDLWEALAKSA-TALVTIDGNDEFVCRSMPNNRFYLVEGVDKSEFNPRLGRKIE EIPGN
Leptinotarsa-1   ESY--EN--ADNENVEELLDFIRKSP-WTIMLFNH----------GRG-------HIVNYNPKFSKESPDGINEA
Leptinotarsa-2   ETY-R----SSEQELADLLEAFEDSP-LAFIAVNGNEKFICKNIPGKR---------SNFIPRLGRNVEDDFSGS
Aethina-1        DLF-REE--LEQKEQANLFDMLQDTP-WTVVAVGD----------GKR-------HVSSFTPRLGRELQDDFGSN
Aethina-2        DH--YNY--LNAEQIQNILEAIEQSP-YTIVILNP-----------TKR------HTVNFAPRLGRDSGEDD---
Aethina-3        EVF-REE--QKE QLDNLLQILQDNP-WAIVAMTD----------GKR-------HVSSFTPRLGREVDDDFGSN
Aethina-4        DVF-REE--MEQKEQANLFDMLQDTP-WAVVAVSD----------GKR-------HVSSFTPRLGRELEEDFGSN
Aethina-5        AEGYYDY--LEADEIQSILEAIEGSP-YSLVVMNP-----------NRH------KSLSFAPRLGRESGEES---
Hycleus          DET-YKN--SETEQLATLMDAVQDSP-WAILAF---K--------GKR-------HTLSFTPRLGRESGEELTN-
Tenebrio         IDP-YRN--REREQLATLLDVIQDSP-WAIVAV---N--------GKR-------HVVNFTPRLGRESGEEFVSS
Tribolium        DDL-YQD--MQKEELVSLTDALQDVP-WAIIAVNDLLE-------GKR-------HVVNFTPRLGRESGEEFVNN
```

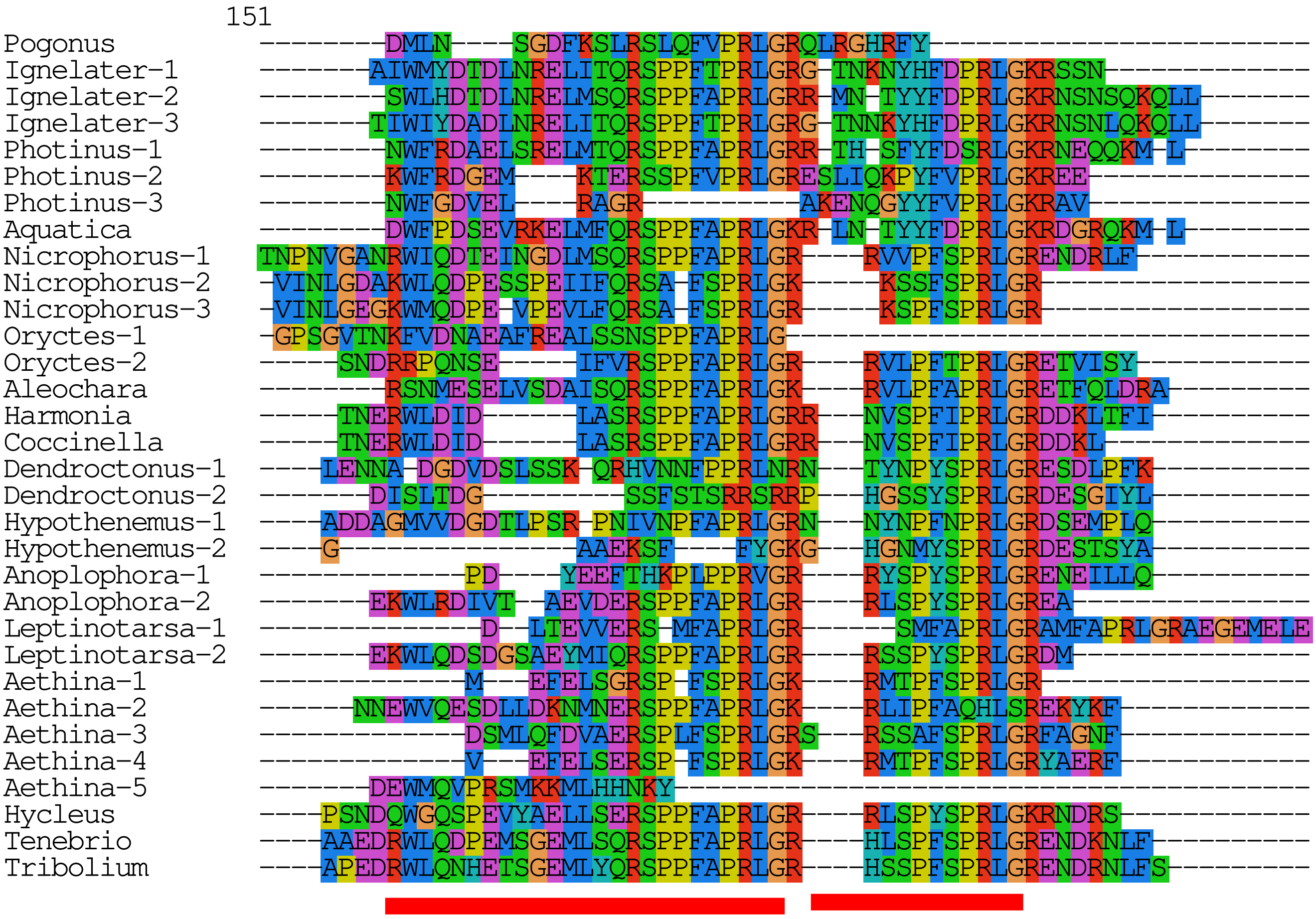

Figure S18. Alignment of pyrokinin precursors.

```
             1
Pogonus      MWFPLSTV-AALCMLLDHFDAK-IYDKELEMAFRQRTRSDWERLWHQEKHARCRESIVKHMKWACEKDIYRITRRSD
Ignelater    MWLPVSTV-AAICVIFDAADATISRDNELEDIFRQRTRSDWEEAWHREKYTRCREMLLQHLYWACEKDIYRLTRRSQ
Photinus     MWLPVSTV-VVLCVVLD---GTVSKDNDLENIFRQRSSSDWQEAWHHEKFTRCRDTLIQHLYWACEKDIYRLSRRSE
Aquatica     MWLPVSTV-AVLCVILDLTKATISKDNDLENDFRQRSKSDWQDAWHREKFTRCRDTLIQHLYWACEKDIYRLTRRSE
Nicrophorus  MWLPVSTV-AALCLLFDGVKAR-GMDKEMDLIFRDRSNTEWKEAWHKERYTRCRETLIRHLYWACEKDIYRITRRSE
Aleochara    MWLPQSTLFTTLCLLLVEA----LNNRELEMKFQLRTPGEWEREWHQERYSRCRDSITRHLRWACEKDIYKLKRAEK
Oryctes      MFFPVSTV-AALCVLVKVADGSSASSNELNSIFRGRTKSEWEEAWHREKFVRCRESLMSHLYWACEKDIYRITRRSK
Coccinella   MWLPLPT-FAVLCVLMDVYEST-QVDNELELIFKQRSHSDWREAWHREKYVRCRETLIKHLFWACENDIYRISRRRP
Harmonia     MLVPFLTLAVVFCVLLNVYECT-QVDNELELIFKQRSHSDWREAWHREKYVRCRETLIKHLFWACENDIYRINRRRP
Anoplophora  MWLPVSTI-AAICVLFDACETS-NSNNDLEIAFRERTDSDWKNAWHKEKYSRCRETLIRHLYWACEKDIYRLTRRSD
Leptinotarsa MWFPVSTI-AAVCVLIDVSDGM-RSENDLELVFKERTHSDWRNAWHQEKHSRCRGDLVKHLFWACEKDIYRLTRRSD
Aethina-1    MKFPVSTI-ATLWLLLDITDGA-MPDRELDLIFRDRSKTDWQNAWHKEKHTRCRERLIGHLYWACEKDIYKLTRRTD
Aethina-2    MWFPVSTI-AAIFVLFDVSDTM-VPDNDLELIFRTRSKSEWQTAWHKEKYSRCRDTLVRHLYWACEKDIYRLTRRTD
Hycleus      MRFPVSTI-ATIFVLFDL---A-SSNNDLELVFRDRSRLDWENVWQNEKHKRCRESLIRHLYWACEKDIYRLTRRSD
Tenebrio     MWFPVSTI-AALCVLLDVSDTT-RSDNELELVFRDRSQSDWEEAWHKEKYTRCRETLIKHLYWACEKDIYRLTRRSD
Tribolium    MWLPVSTI-AALCVLLDMSDTT-RSENELELVFRDRSQSDWEEAWHKEKYTRCRETLIKHLYWACEKDIYRLTRRSD

            78
Pogonus      FT-----------------NIHEMFPWIQETRAKRMIRLRRSGDKR-RSSITNECCSSAGCTWEEYAEYCPTNKRIT
Ignelater    QPKN-VNFF-------DNEKQYLNFPWLTPKQAQQIVRSRR-----GGSSITSECCSSTGCTWEEYAEYCPTNKRHT
Photinus     KPRV-I-KL-------GERYEYDKPPWISVMRARQLVRSRR-----GGSSITSECCSSSGCTWEEYAEYCPTNKRHT
Aquatica     RNNL-K-PY-------KEENNSFKFPWISAMAAKQIIRSRR-----GGSSITSECCSSTGCTWEEYAEYCPTNKRHT
Nicrophorus  HPMQL---A-------NEDLESLSFPWISDEEAHFMVRTKRAANRNGGSSITAECCSRKGCTWEEYAEYCPTNKRYT
Aleochara    PDIYRLKRIENELEDEDVQEYESFYPYYLPETAMAMLRTKRGLSKRSG-IITQECCSPKGCTWEEYAEYCTQNNRKV
Oryctes      PAVQQIDFE-------TGKHMVVDFPWIPKKHALVYLRYRRGYSARSSHSITSECCRSSGCTWEEYAEYCPINKRYV
Coccinella   EKRFN-----------NIFDEDTKYPWIPEKRAKLFIRSRRGINRRTG-SITSECCKSSGCTWEEYAEYCPTNKRYT
Harmonia     AKRSD-----------NIFDEDVKYPWIPEKKAKIFLRSRRGMNRRTG-SITSECCKSSGCTWEEYAEYCPTNKRYT
Anoplophora  PS----YKY--------YNDNELDYPWLVPNKAKRLLRFRRNINRRSGSSITNECCKSSGCTWEEYAEYCPTNKRYT
Leptinotarsa PRYKSYDDY-------DNDDIEVDFPWKSPRRAKRILRFRRSIDRRSGSSITNECCKSTGCTWEEYAEYCPTNKRYT
Aethina-1    PTFKN---Y-------FMESDELNYPWLIPSKAKSLLQFKRGIK-RSGSSITSECCMSTGCTWEEYAEYCPNNKRHT
Aethina-2    PYFMV-----------VLWRTSDSFPWISPTKAKRYLRFKRDVK-RSGPSITSECCKSTGCTWEEYAEYCPTNKRHN
Hycleus      PTFI---------------NDKEQFPWLSVEGAKSFLRFRRASSRRGGSSITAECCKSTGCTWEEYAEYCPTNKRHT
Tenebrio     QNG--FNNY--------INNVDEEFPWMAPKKAKRLLRFRRGVNRRAGSSITSECCKSSGCTWEEYAEYCPTNKRYT
Tribolium    Q-S--YNNY--------ITNTDEEFPYLAPKKAKRLLRFRRGVNRRAGASITSECCKSSGCTWEEYAEYCPTNKRYT

           155
Pogonus      PFV--------
Ignelater    VYVD-------
Photinus     VFVD-------
Aquatica     VFVN-------
Nicrophorus  NYL--------
Aleochara    PHKRYEYISDY
Oryctes      -----------
Coccinella   SYV--------
Harmonia     SYV--------
Anoplophora  SYI--------
Leptinotarsa SYV--------
Aethina-1    LYSSY------
Aethina-2    SVLNSYL----
Hycleus      PFVSRF-----
Tenebrio     SYV--------
Tribolium    SYV--------
```

Figure S19. Alignment of relaxin precursors.

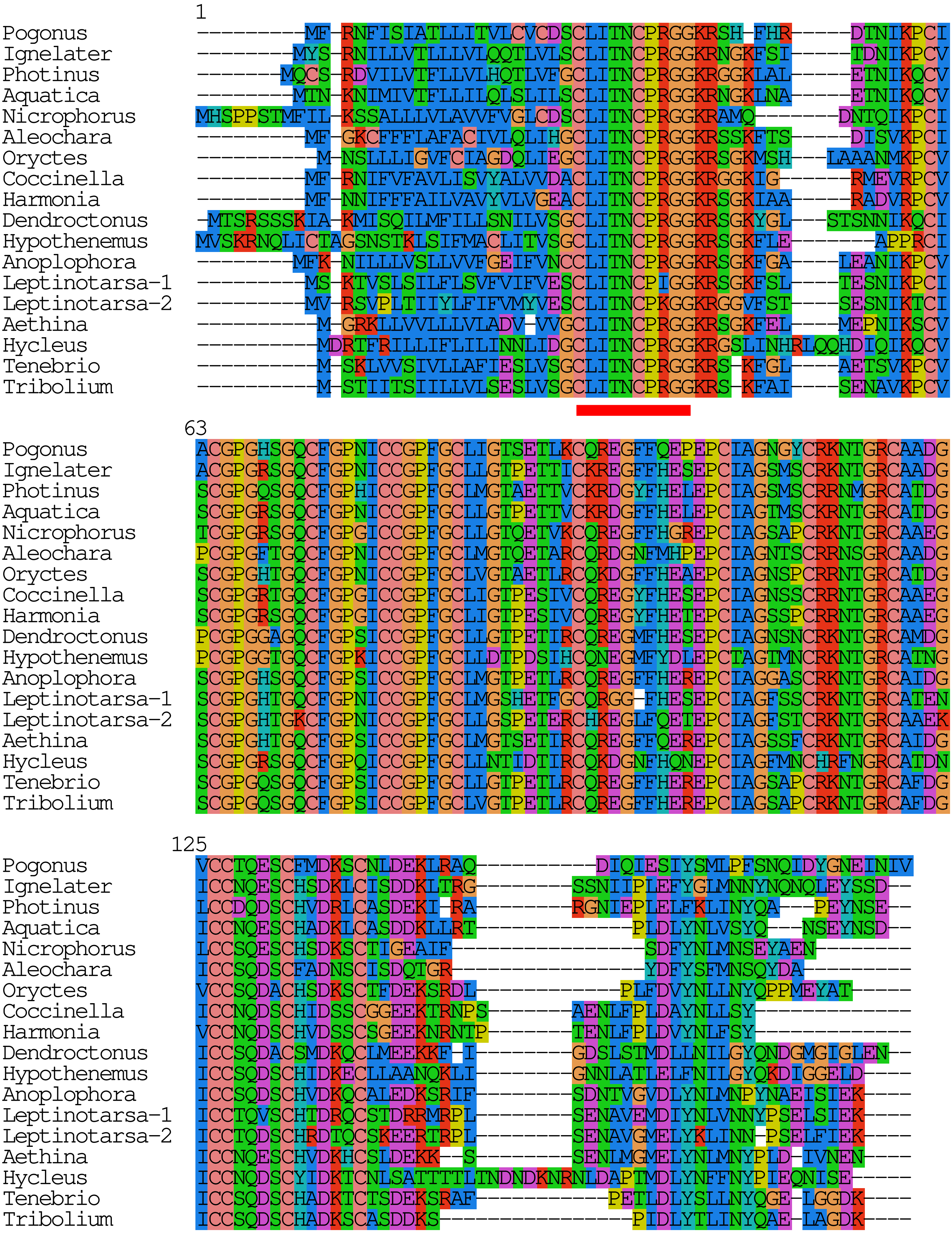

Figure S20. Alignment of vasopressin precursors.

```
                1
Pogonus         -MKSKGFEVIY---C--TLFVLSVISMCIARPQQSINDLSPQAVGSGTGGGRLSESAAVRNLI
Ignelater       MSSNQTAQLLARSFL--IIVVVGVVSVS-ARAAHFGDSNQVNPDQE-SAA---------N-L-
Photinus        -MPQQIMQFLMRSIL--LLLVLATVSTSARSTTHFGETNQINQEQDGSSG---------G-L-
Aquatica        -MSNQAAQVFVRFFL--IIALMTTISVS-ARSAHFEEASEVNSEPDGPQA---------N-I-
Nicrophorus-1   -MVCNMVSLISRSVL--VMLIV--SSLVSARPQHFGDSSQGLSAEDLAG------------L-
Nicrophorus-2   -MVCNMVSLISRSVL--VMLIV--SSLVSARPQHFGDSSQVLGEQESDGG---------NAL-
Aleochara-1     ---------MMRNFILLLAVAVASFALVSARPQKYIDSLSQAGDG--VDD--VA------DNQ
Aleochara-2     ---------MMRNFILLLAVAVASFALVSARPQKYIDSLSQSAPL--SALTPLAEADSNTNEV
Oryctes         ----------------------------------------PSSDQDTSNM---------V-D-
Coccinella      -MNTDLVRI----KS--FLLLLIVVGAVAAKPSHFSDNQVTPDAGDRSDI-------------
Harmonia        -MSSDLVRL----TS--FLLLCLLVASIAAKPSHFPDNQVTPD-GDRSDM-------------
Dendroctonus    -MQYEFPQVISRVF---FAILIMSLVAVNAKPPHYQENT--QGLNADQE-------STLNNV-
Hypothenemus    -MQLEFSQVIPRLFL--FAIFFVSLASVMGKPPNFQETASPQVFNNDPD-------PVINNN-
Anoplophora     --MQDLSQFF-SKYV--FILLVVTLIFVNARPSHLSESSQ--LL-P----------DQESNM-
Leptinotarsa    -MMQGFLHLFMKAF---LILLLVAALTT--SNARHLGENSLPENSQ-FMV------DPDNNQ-
Aethina         -MSSEIPVFLT-KAL--LILIVVSVAVANARPSHFGENNQAV-----SEQ------ESNNNI-
Hycleus         -----MSQPNNLGPV--VVFLVALLASTKARPSHFADNQL-LPETD-------------TNL-
Tenebrio        -MSAQVPHYLFRTLL--VL-FVATLAVSSARPNHFGDPNQVVAE-G--DA---------NNL-
Tribolium       -MAAQLPRYLTKTLF--IF-LIATLVVANARPNHFGDASQVVGEPA--DG---------NNL-

                64
Pogonus         DTDDD-LTALK-MQLLA---QQLA-EYNAVP---------QLPDYAA------------DRF-
Ignelater       -VDSG-LTPLQ-LEMLA---QRLS-EYNNQI---------PQVPAAW------------DRL-
Photinus        -IDSG-LTPLQ-LEMLA---QRLS-EYN-QI---------PPIPAAW------------ERL-
Aquatica        -VENG-LTPLQ-LDLLA---QRIS-ELN-QI---------PPIPIAW------------ERL-
Nicrophorus-1   -L----EDPMV-LAYLR---KDVE-RILDAA---------GSSDYEW------------DTM-
Nicrophorus-2   -GSPG-FSDLQ-LEMLA---HRLA-EYNR------------DLPVGW------------ERM-
Aleochara-1     LTDMQ----F---ELIA---QQLAN-----L-N-SRP----QF----------------AAAM
Aleochara-2     LPPMA-LLQLGLWQMLD---DRFDDDFSP-YSH-SRSLRQRQHDGHLKQALLAQSTRSQNGIF
Oryctes         -PSAG-WTNEQ-MEQLL---QHLA-ELN-RV---------PQYPYGW------------ERL-
Coccinella      -MDPGLINAWRRYDFLM---RRLAADYDNGN---------MANDNPL------------DRL-
Harmonia        -IDPGLLNAWRRYDLMM---RRLTADYDNAN---------MANENPL------------ERM-
Dendroctonus    -LDSG-LRPWQ-LELFA---QRMA-ELNQ-LSQMNNNLNGQEY-GTF------------DRAL
Hypothenemus    -LDSV-FRPWQ-LEFIA---QRMA-ELNQ-LGQMNN-LNGQEYGGGF------------ERAL
Anoplophora     -IDSG-YKQWQ-MEMLA---QKLA-ELNQ-LGNGEY---------GL------------ERSL
Leptinotarsa    -YEPG-MKQWQ-MELIA---QKLA-ELNS-P-------------IGF------------QRDL
Aethina         -LDGG-LKPWQ-YEQLLAQAQRLA-ELNG-NDQDA---------FGF------------ERAL
Hycleus         -LETN-FKPWH-LELLA---QRLS-EMNLPAAQQMNNINNNIDDYGW------------ERAL
Tenebrio        -LDSG-LKPWQ-LEMLA---QRLS-EIS--Q---------TGGDYGW------------DKS-
Tribolium       -LDSR-LKPWE-LEMLV---QRLS-EISS-Q---------TGGDFAW------------DKS-

                127
Pogonus         -------VLSKRQQR-FRQCYFNPISCFRK
Ignelater       ----ARNPEVKRQSR-YRQCYFNPISCFRK
Photinus        ----ARNPEVKRQSR-YRQCYFNPISCFRK
Aquatica        ----VRNPEVKRQRR-YQKCYFNPISCFRK
Nicrophorus-1   ----D--MPSKRQTN-RLKCYFNPVSCF--
Nicrophorus-2   ----IRSPEMKRQSR-FRQCYFNPVSCFKK
Aleochara-1     DKHLFRLPEMKRASR-FRQCYFNPVSCFRK
Aleochara-2     QSGLPG-MPVKRQGK-RLKCYFNPVSCF--
Oryctes         ----IRGPEMKRQSR-FRQCYFNPVSCFKK
Coccinella      ----FRNPEMKRQGRSYRQCYFNPISCFKK
Harmonia        ----FRNPEMKRQGRSYRQCYFNPISCFKK
Dendroctonus    RS-G---NEIKRQSR-YRLCYFNPVSCFKK
Hypothenemus    RSER---NEIKRQSR-YRLCYFNPVSCFKK
Anoplophora     RS-G---NEIKRQSR-YRLCYFNPVSCFKK
Leptinotarsa    RS------ETERKRR-FRACYFNPVSCFKK
Aethina         RS-R---SDVKRQSR-YRLCYFNPVSCFKK
Hycleus         RERGIRSPESKRQSR-YRQCYFNPISCFKK
Tenebrio        ----IRSPESKRQSR-YRQCYFNPISCFRK
Tribolium       ----IRLPEAKRQSR-YRQCYFNPISCFRK
```

Figure S21. Alignment of allatostatin CCC precursors.

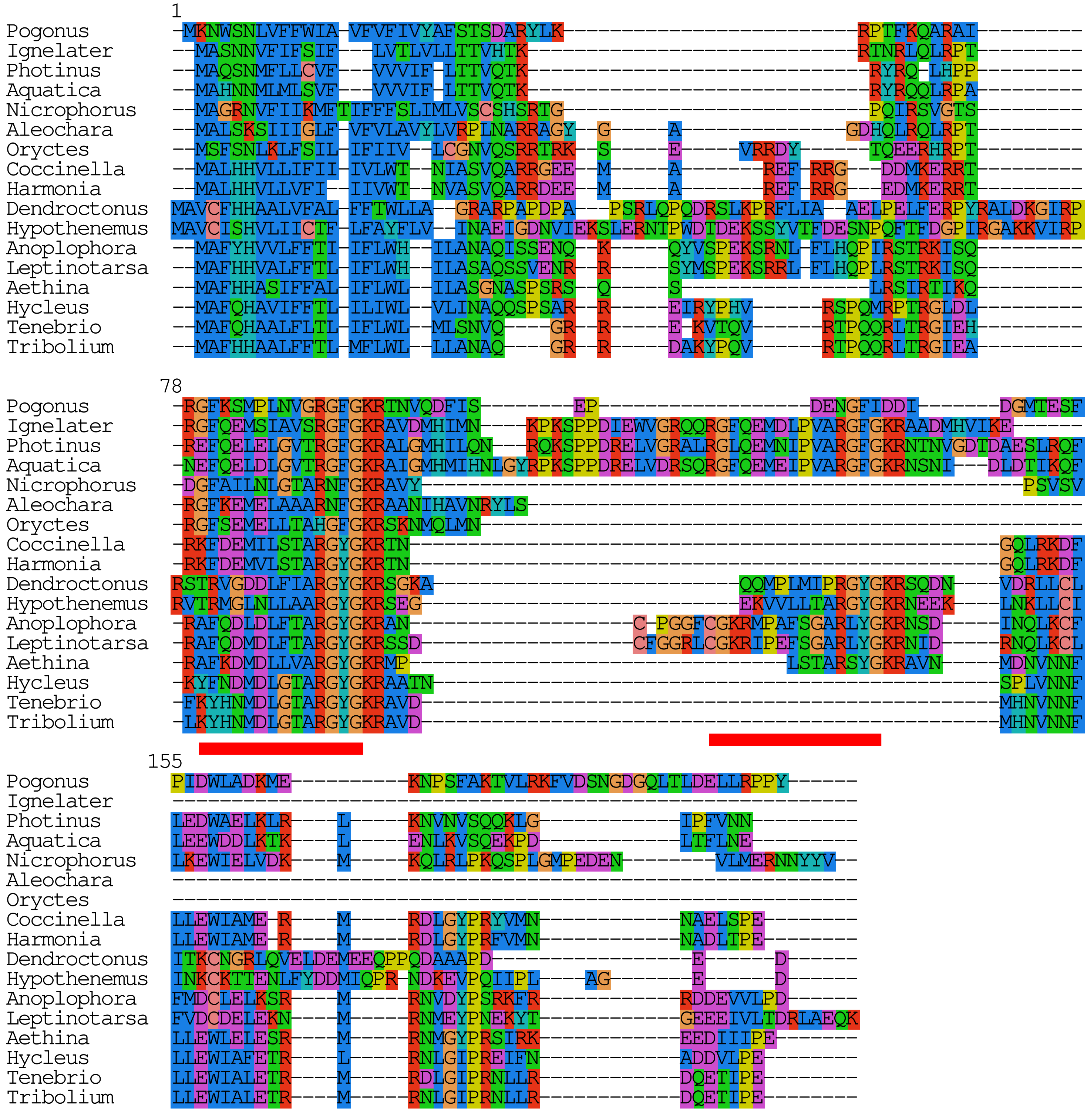

Figure S22. Alignment of allatotropin precursors.

Pogonus
MKTLIILMVISFAFATPYQYIRNRQYQIKHFKNSNQIARTHELPNSLQTLFKSFLLKRCSNLGNDDCSNGYLPE
IGNDDDYLGSGNTPGKRCSNLGNDDC

Ignelater
MNGIILLFGIVLSLDYTWGSPTKRCANMFDEGCINGGVGGAGRDEEWIKDPGNTPGKRCANMFDEGCINGGIGG
SGRDEEWIKDPGNTPGKRCANMFDEGCINEGIGGAGSDEEWIKNPGNTPGKRNLLFKNIPRLYSRSVHKNHQGS
TRRFG

Photinus, pseudogene: has no signal peptide and is not expressed
QIRPKCANLFDESCLNKNLIDSALDEQWLSNGHVPGKRNLNDEGKYTFL

Aquatica
MVKYLVSFLIISISLGSKYVNSARIKRCANLFGEDCINGGTIGGGPDQLWITGNGKQKFLHNLPKVNPQKCVNA
FDTNCINNRRLQEQSNQAWSTYPESTYRKENIQDKKVLNMLYLILKGHSIPTKRCANLMDESCINGGIVGSGDD
EEWLTGDGSPGRR

Nicrophorus
MIRFMVLFILVLGTIDAKPKPCHHRDQACLENQVAIHRPPGIFKSLSDFFNRVDIVTSKRASCSNLYDEGCING
GLPGAGDDEDWLNGNGLGKRASCSNLYDEGCINGGLPGAGDDEDWLNGNGLGKRAGHKDWMNKSGIGKRASCSN
LYDEGCINGGLPGAGDDEDWLNGNGLGKRASCSNIYDEGCINGGLPGAGNDEDWLNGNGLGKRASCSNIYDEGC
INGGLPGAGNDEDWLNGNGLGKRALCSNIYDEGCINGGLPGAGNDEDWLNGNGLGKRASCSNLYDEGCINGGLP
GASEDQAWLTGNGLGKRVNIPQPGYNKNNFKGNKNSKTPIMY

Aleochara
MIRIITLPVVFSAVFLISDGAPCANLFDEGCTYRLLKESAMDQGWLRKNHLGKRTFQEHTQIGIDDFFHRLHNI
LNDRCNNFFDINCIEPNIENHQRVDVNPDAIRAFALQLHEDILNRCAQNAMDKNCNFIQNFTPKDDDDKWIA

Oryctes
MKLLTLILLSVGTINGGYIGKKYCPGLFGDSCASGGLDGSADDEDWLNKPNNPGKRCIANVFDGSCSYGATLPG
AAADEYWKQHGSPGKRCSNVFDESCSNGAVVNGGSDEDFLNGNGNPGKRCSNLFDESCSNAGLIGAGDDESWLN
GGGSPGKRCSNLFDESCSNGGLIGAGDDENWLNGGGSPGKRYLPKQWYKAWGPGGVKTAKRHVSDKVHESLLRK
MAKIRHM

Coccinella
MRKQQFFLMLVLTIHLSQLIECRSYSAAILEEVLRRNSHQNLSPEDVHLFNELYNRLAMPVRKRCANMFDEGCS
NIGIHGSGSDEDWIKVNSPGKR

Harmonia
MKFLFALIMTTIYLFLLVESRSFSSLSFEEAVGRNSHPKLSREDIELFNELYRRLSLPVRKRCANMFDEGCSNI
GIHGSGSDEDWLKDNSPGKR

Dendroctonus
MNRIITLLAISVALSAAMSVPNVYHKTSNSLKQEQLAALINQLLREKQVYQEVKRECLRMGPLGCLNDPRKHLL
TMYNPGKRCANFGDDGCLSGGVQGAGSDSDWLASGFTPGKRCANFGDEGCVTGGVIGAASDSDWIGGGFSPGKR
CANLGDEGCANGGVPGAAADTDWIDGGYSPGKRCANLGDEGCVTGGVSGAGSDSDWISGGFSPGKRCANLGDEG
CANAGVGGAGSDADWLSGDFNPGRR

Hypothenemus
MKCIFIVLAVSVCLSFVRSFPNNPDTYRQSTQLKQKKLSEIIEDLMRDKERYRGSIREYDGCGSAGVPGAGSDS
DWIGGGFTPGKRCANFGDDGCANGGIPGANADQDWLDGGFSPGKRCANLGDEGCANGGVEGAGSDADWLNGNFN
PGRKR

Anoplophora-1
MNFLLAITAVLLGTSQGMYLQRNPHHQQAVARQSRNEPTVLKSLGDFFHRMHAAATKRCAYLLDESCNNGGIPG
AGSDNDWLNQGFNPGKRGLNLFEEGAAYNGLSGSGADSDWLNGGFNPGKRCVNTMDESCSNGGIPGSGSDSDWL

DGGFNPGKRSLNLFEEGIVNKGVSGAAADNDWLNGGFNPGKRCANTMDESCGNGGIPGSGEDRDWLDDGSANPG
KRRLNGIEGVPGSGNDNDWLHGDNTPGKR

Anoplophora-2
MKCLLAITVVLLGSSQGFYVERNPHQQLAIARQSRNQPTIFQSLGDLFHRMHATATKRCAYLLDEACNNGGIPG
AGSDSDWLTQGFNPGKRSSKVLEEEPDNDLNPEPDDDRPNGDFNLGKHCVNAMDESCDNEEIPGAGPERDWLHR
GFHPGKPGLHPFEEDNDVPVAAADGGWLSSFSIVCPNFMDVCGNVGGKRVPGSGADRDWLEEGGGNPGKRRLNG
IPGSGKDNDWLHGDNTPGKR

Leptinotarsa
MNTSTLFLIVLLGFTTAVPLQQWHANKYSSEYHKPNQRGLFSSLGKLFNPSSNKRILNSRGGFSSGERSLNLFD
DSVANSKISGSGSDSDWINGGFSPGKRCANLMGESCNNGGVPGSGSDDDWIHGGFSPGKRSLNLFDDGAANSKI
SGSGSDTEWIDGGFSPGKRCANLMDESCSNGGVPGSGSDDDWIHGGATPGKRSLNLFDDGAANSKISGSGSDSE
WIHGGFSPGKRSLNLLNYASNSKIPGSGSDSDWLNLDGFNSGRRGIKSSEKIGHVFGG

Aethina
MRGIIVVLAMVLAVTLARYVRRHPADVAMTRAKIHLKRNINDPELFKQLGDFFHRMHSASIRCSPGLLDEGCSN
GGIPGSGTDADWINNYGPGKRCVNILSESCDNGGIPGAGSDDDWIHDNSPGKRCANILDEGCNNGGVPGSGSDA
DWINNNSPGK

Hycleus-1
MKAIFIIVLVFLSLANCYYLAPHANRPVKVVPLRNLNDLLYRLNGLALRRCANLFDESCANGGMSGAGADDDWL
NGNTNPGKKRCANLFDESCSNGDINGAGSDDDWLNGDTNPGRR

Hycleus-2
MKFVKILLILLLTSTYAIDIHQSNRKSLKLLKKLINSENYGFLGRLKRRHCANLYDESCINVHLPLAKIDEDWL
RKGFNPGK

Tribolium-1
MKTAFLLLFVLMPAVLCYYVHPNLHYFPARVNYRSAPAKSWGALFHRLQLASKRCANTFDESCINDVINGAGSD
EAFLNGGDNPGKRCVNTFDESCSNGDINGAGSDDDWLHGDDTPGRR

Tribolium-2
MRPVLVLVMIFSVSYARYLEPYNYGYPRPLVDFLNRLSLSDKIKRCGNTFDESCANLPIIGASSDESWLAHSSP
GKRCANVWGESCINGGIIGGGSDQSWLQGDDNPGRR

Tenebrio-1
MKAAFVLLAIALPAAYCFYLQPNYHVPARLGTRNAPVYKSLGDLFHRLHLASKRCVNTVDESCINGGGNDAGND
EDFLNGGDTPGKRCANLYDESCSNGGINGAGADDDWLHGGNNPGRR

Tenebrio-2
MKLSLLIFAVALSTACGLYMYPKHRPRDEEVFQSLASFFNQLGRRANMKRCANTFDESCLNGPIGGATSDENWL
SNGSPGKRCSNIFGSSCVDGGTAGAGADEDFLGGGGGPGRR

Figure S23. Sequences of the various Calcitonin B genes from Coleoptera. Mature peptide sequences are in light blue the conserved C-terminal proline in dark blue, Gly residues that expected to be transformed into C-terminal amide in green and convertase cleavage sites and dibasic amino acid residues anticipated to be removed by carboxypeptidases in red. Signal peptides are highlighted in yellow.

```
              1
Pogonus-1     --MQ--KLNNG-SVIMRTTALILGVFFLCVATLDAAPTPRLIESFKDSVTSIDKCENYLNTKLGQKIKLPVDTIQET
Pogonus-2     --MQ--KLNNG-SVIMRTTALILGVFFLCVATLDAAPTPRLI-----------------------------------
Ignelater     --MKLPTIG-SNG-IMWLVILVAGTLLLISSS---------------------------------------------
Photinus      --MKLPSIT-NNG-IMWFAVILAASSILLSST---------------------------------------------
Aquatica      --MKVPTSA-NNG-IMWLAVAVATSVLLVSST---------------------------------------------
Nicrophorus   --MKSPAFA-FRS-SFVLFLVLAGMLIFLTTS---------------------------------------------
Aleochara     MRFVMQTQTRANG-LS-LVVILLGVFLLAATQVSHAA----------------------------------------
Oryctes       --MK-SIMNGSNT-IAVAVLFMAGVLIFMATT---------------------------------------------
Coccinella    --------MVTHS-VAIMLLIVPGLLLRIQNA---------------------------------------------
Harmonia      --------MVLNS-MSFLVFIIPGLLLRIDNV---------------------------------------------
Dendroctonus  MTMKYSSSSGNYG-SL-IMLALAALLVLELTT---------------------------------------------
Hypothenemus  --MNTSSPNNRSSCKSIILMALAILLVYELASSVAEAYPARVVEQ--------------------------PNLLFY
Anoplophora   --MKPSMVNSS-T-TSVLVLVLVGIVLFVTTT---------------------------------------------
Leptinotarsa  --MRSSMIH-SNG-TTIFMILLVGIVLFEITT---------------------------------------------
Aethina       -----------MQ-RPIFVLLVIGIVLLQVTT---------------------------------------------
Hycleus       --MKPSMNNNNNG-RAFFIFFIIGMVLFEATT---------------------------------------------
Tenebrio      --MKPSMIH-SNG-VSLLVLLAAGIILFEATT---------------------------------------------
Tribolium     ------MVP-NNG-ISLLMLLVAGMILFQATT---------------------------------------------

             78
Pogonus-1     LYMYNSYEQCSIVYALLKIMSPSYYSDSLTNDESQR-----LGILEMLAQLGQNMIR---DDLENSKRGLGFGMDRG
Pogonus-2     -------------------MSPSYYSDSLTNDESQR-----LGILEMLAQLGQNMIR---DDLENSKRGLGFGMDRG
Ignelater     -----------SEAAP---PLPSYYSPLSLEGQNPEYI---LQTLARLRQAIIAE-----EDLENSKRGLDLGLGRG
Photinus      -----------VEAAP-SAPSPGYYAPISVEGQNPEYI---LQALVRLRQALNAE-----EDLENTKRGIDLGLGRG
Aquatica      -----------VEAAP-SAPSSGYYPPLSFEGQNPEYI---LQALVRLRQALNAE-----EDLENTKRGIDLGLGRG
Nicrophorus   ----------TCYAAPHNSRYMGYYGSN-QDGQNPEYL---LQTLARIRQAIIAE-----EDLENSKRGLDLGLGRG
Aleochara     ----------PHYPNTLQEYYQNYPRSLDMPNANPEYLFNSIEKLAQLRQQLM-N-----ADLENTKRGIDLGLGRG
Oryctes       ----------TSAAPH-IFSNPGYYSSLSYENQNPEYL---LQTLARLRQALLAE-----EDLENSKRGLDLGLGRG
Coccinella    ---------------HAASLLPRSPEEDLISRDDPEYI---IRVFRQFRTYYNNIPDDEDDSWTDSKRGLDLGLGRG
Harmonia      ---------------NSASILNSLPEEKIFARENPEYL---LRMYPRLRSTYNIVPDEDDDSWTDSKRGLDLGLGRG
Dendroctonus  ----------TFA-SAYPARYSAYYASIPLEE----------QLIERIKQAFIED-----NDLENSKRGIDLGLGRG
Hypothenemus  KTHFHSNCFPIEVNNECVFSYPAYYSPMGLEEQNPNYVN-LQQAIERIRQAFMQN-----NEIENTKRGIDFGLGRG
Anoplophora   ----------NAAPHSPKFYYPAYYSPLSFDGQNPEYL---LQAIARLREALIND-----NDLENTKRGLDLGLGRG
Leptinotarsa  ----------TSAAPR-QASYPGYYS---LEAQNPDYL---LQTIARLRQALIND-----DDLENSKRGLDLGLGRG
Aethina       ----------TYAAPHPARYYPGYYSPMQMEGANSEYL---LQTIARLRQALNA------DDLENSKRGLDLGLGRG
Hycleus       ----------TTFAAPHSSSYPGYYSPLSMEGQNPEYL---LQTIARLRQALIAD-----DDLENSKRGLDLGLGRG
Tenebrio      ----------TYAAPHSSRYYPGYYSPLSMEGQNPEYL---LQTIARLRQALIAD-----DDLENSKRGLDLGLGRG
Tribolium     ----------TYAAPHSPRYYPGYYSPISMEGQNPEYL---LQTIARLRQALISD-----DDLENSKRGLDLGLGRG

            155
Pogonus-1     YSAQRAAQHLMGLHAATWAGGPGRKRSGEEVKRMRIGMGMDRGYSGSQAAQYLIGMDAANRIGKRMDSNASNGEPEE
Pogonus-2     YSAQRAAQHLMGLHAATWAGGPGRKRSGEEVKRMRIGMGMDRGYSGSQAAQYLIGMDAANRIGKRMDSNASNGEPEE
Ignelater     FSGSQAAKHLMGLAAANFAGGPGRRRRSEEEA---------------------------------------------
Photinus      YSGSQAAKHLMGLAAANFAGGPGRRRRSVDDA---------------------------------------------
Aquatica      YSGSQAAKHLMGLAAANFAGGPGRRRRSVVDA---------------------------------------------
Nicrophorus   FSGSQAAKHLMGLAAANFAGGPGRRRRSDEDA---------------------------------------------
Aleochara     FSGSQAAKHLMGLAAANFAGGPGRRRRSVSVQEQYANMGDELGV---------------------------------
Oryctes       YSGSQAAKHLMGLAAANFAGGPGRRRRSDENA---------------------------------------------
Coccinella    YSGSLAAKQRMGLASANFAGGPGRRRRNGLEV*--------------------------------------------
Harmonia      YSGSLAAKQRIGWAAANFAGGPGRRRRNGYEA---------------------------------------------
Dendroctonus  FSGSQAAKHLMGLAAANFAGGPGRRRRSDGEA---------------------------------------------
Hypothenemus  FSGSQAAKHLMGLAAANFAGGPGRRRRSDDSA---------------------------------------------
Anoplophora   FSGSQAAKHLMGLAAANFAGGPGRRRRSDDEDA--------------------------------------------
Leptinotarsa  FSGSQAAKHLMGLAAANFAGGPGRRRRSDDDEA--------------------------------------------
Aethina       FSGSQAAKHLMGLAAANFAGGPGRKRRSDDEE---------------------------------------------
Hycleus       FSGSQAAKHLMGLAAANFAGGPGRRRRSDEDA---------------------------------------------
Tenebrio      FSGSQAAKHLMGLAAANFAGGPGRRRRSEEEA---------------------------------------------
Tribolium     FSGSQAAKHLMGLAAANFAGGPGRRRRSEEEA---------------------------------------------
```

Figure S24. Alignment of DH31 precursors.

```
                      1
Pogonus-3/4a       ---------------KRGLGFG-MDRGYSAQRAAQHLMGLHAATWAGGPGRKR--------
Ignelater-2        ---------------KRGLDLG-LGRGFSGSQAAKHLMGLAAANFAGGPGRRRR-------
Photinus           ---------------KRGIDLG-LGRGYSGSQAAKHLMGLAAANFAGGPGRRRR-------
Aquatica           ---------------KRGIDLG-LGRGYSGSQAAKHLMGLAAANFAGGPGRRRR-------
Nicrophorus        ---------------KRGLDLG-LGRGFSGSQAAKHLMGLAAANFAGGPGRRRR-------
Aleochara          ---------------KRGIDLG-LGRGFSGSQAAKHLMGLAAANFAGGPGRRRR-------
Oryctes            ---------------KRGLDLG-LGRGYSGSQAAKHLMGLAAANFAGGPGRRRR-------
Coccinella         ---------------KRGLDLG-LGRGYSGSLAAKQRMGLASANFAGGPGRRRR-------
Harmonia           ---------------KRGLDLG-LGRGYSGSLAAKQRIGWAAANFAGGPGRRRR-------
Dendroctonus-3     ---------------KRGIDLG-LGRGFSGSQAAKHLMGLAAANFAGGPGRRRR-------
Hypothenemus-3     ---------------KRGIDFG-LGRGFSGSQAAKHLMGLAAANFAGGPGRRRR-------
Anoplophora-2      ---------------KRGLDLG-LGRGFSGSQAAKHLMGLAAANFAGGPGRRRR-------
Leptinotarsa-2     ---------------KRGLDLG-LGRGFSGSQAAKHLMGLAAANFAGGPGRRRR-------
Aethina-3          ---------------KRGLDLG-LGRGFSGSQAAKHLMGLAAANFAGGPGRKRR-------
Hycleus-3          ---------------KRGLDLG-LGRGFSGSQAAKHLMGLAAANFAGGPGRRRR-------
Tenebrio-3         ---------------KRGLDLG-LGRGFSGSQAAKHLMGLAAANFAGGPGRRRR-------
Tribolium-3        ---------------KRGLDLG-LGRGFSGSQAAKHLMGLAAANFAGGPGRRRR-------
Ignelater-1        ------------KRNIKSVDFG-MGRGHSGTLEAQYRMALESAFSPNGPGRKKR-------
Hycleus-2          -------------KRYLQLSFG-LDRGSSGNFENFTRCLQLIASSPYGPGRRKR-------
Tenebrio-2         ---------------KRFVSFG-VDRGSSGKVENLTRCLAFRASSPCGPGRKKR-------
Tribolium-2        ---------------KRFLSFG-VDRGSSGKVENLTRCLAFRASSPCGPGRRKR-------
Pogonus-1/2        ------------RLRRTIPDFG-LSRGYSGNLEAAHRLGYDRAMDPQGPGKR---------
Aethina-2          --------------KRISLDLG-TDSSHPAKLQNDILNMNELLRNYG--------------
Hycleus-1          --------------KRARPDLG-MDRGFSGRLQNSQLVAKYYGDLRG--------------
Tenebrio-1         --------------KRGGPDLG-MDRGYSGKLQNSQLLAKYYGDLRG--------------
Tribolium-1        --------------KRVRPDLG-MDRGYSGKLQNSQLLAQYYGQLRG--------------
Aethina-1          ---------------KRGIDFG-INRGFPPLWKKCMLTKMNALKTYG--------------
Leptinotarsa-1     ---------------KRVIDLGTQSRGYSGKMENMYLTNADMLRRIGKR------------
Pogonus-3/4b       --------------KRMRIGMG-MDRGYSGSQAAQYLIGMDAANRIGKR------------
Dendroctonus-2b    RVRKQSLVNCAEIMSKAQLDFG-TRQSDAGSIENCLMTLRTELNHLGK-------------
Hypothenemus-2     ---RRPRINCSQFFSKGGLDFG-TRQNDPGSIENCLRTIRTELHHFGK-------------
Anoplophora-1      ---------------KRKTDFG-TDRGYSGQQEHNMRMNKYLLEYVG--------------
Hypothenemus-1     --------------LKRQADFG-SQRGHTGRQEYALRYWSHLG------------------
Dendroctonus-1     ---------------KRTADFG-SQRDHTGQREYALRYWTHLG------------------
Dendroctonus-2a    ---------------KRTADFG-SQRDHTGQREYALRTITEKPELVPESNTMEPRVRKQSL
```

Figure S25. Alignment of DH31 and other putative neuropeptides encoded by transcripts from Coleoptera DH31 genes. Numbers behind the names indicates the transcript number (some species seem to have only one transcript). When this number is followed by a letter it indicates the putative peptide on that particular transcript. For example Pogonus-3/4a indicates DH31 itself that is coded by two transcripts in Pogonus and a refers to the first putative neuropeptide on this transcript. Pogonus-3/4b refers to the second putative neuropeptide on this transcript. Note that sequence conservation is very strong for DH31, but much less for the other putative neuropeptides encoded by these genes.

```
               1
Pogonus        -----MRVPVMYLVCAATLLVSS-RAAPVDTDSNDPETSMG--IGSAASINGQDPSETL-AYLLPRLSTRYRPTEQW
Ignelater      -----MRVPV-YLVCVALILCVNCE---------EQTNYY---GRRIQPVDLTADQETV-TYLLPKLTAKYRPNNEW
Photinus       -----MRVPV-YFVCAALILYVKSE---------DRTN Y---DRRIEPLDLTADQETV-SYLLPKLTAKYRPNTEW
Aquatica       -----MRVPV-YFVCAALIFYVKGE---------DLSN Y---DRRIQPLDLTADQETV-SYLLPKLTAKYRPNTEW
Nicrophorus    -----MNVPV-YIVCAALIVAVNSE---------ENP LF---GRENEPMD    REAM-GYILPKLMPRYRAISNK
Aleochara      -----MRVPV-YIVCAALIVAVNSE---------ENY YP---GRQQE       ADAL-GYILPRLMAKYRPMRPW
Oryctes        -----MRMPV-YLVCAALAVAVKSE---------ELPSLY---GRQLEPLEVNSEQDAL-RYIFPRLTSRFRASKNW
Coccinella     -----MRWSI-YLACAALVISIKAD--------ENSVDYY---GSFLEPINIAAEQETLNNYLLPKVQPKYKP--EW
Harmonia       -----MHWPV-YLAFAVLVIATKAE--------DNSADYY---GSFLEPINIAPEQETLNNYLLPKIQPKYKP--DW
Dendroctonus   -----MRAPI-YLVCAALVVAVRSE---------PNENYM-----GLESVNVAADPETI-NYLLPKLAAKYRLNSDW
Hypothenemus   -----MRAPL-YLVCAVLAWTAHGD------DQGQQQNYM-----DLEPVDVAADPETV-NYLLPKLTAKYRPNNEW
Anoplophora    MGAHIMRMPV-YLVCAAALVA  VK------GE EGTNY---FGRQLEPVDVAADQETI-SYLLPKLAAKYRPSNDW
Leptinotarsa   -----MRAPL-FVLCAALVVAIRSE------DTSAVNTNF--FNRQLEPINMAADQETI-SYLLPKLAAKYRPNSDW
Aethina        -----MRASF-NLLCAATLMFALASCAELNNEATGTGNYYGDAGRSLEPLNVAADPETI-NYLLPRLTAKYRPNNDW
Hycleus        -----MRVPL-YYLLWAILIVTVKT------NG-ETSNYY--GGRFLEPIDVAADQETV-SYLLPKLTAKYRPNSDW
Tenebrio       -----MRVPV-YLVCAALVVA--VK------SE-DH---N--YGRLLEPIDVAADQETV-SYLLPKLTAKYRPNNEW
Tribolium      -----MRVPV-YLVCAALVVV--VR------SE-ERTNYY--GGRYLEPVDVAADQETV-SYLLPKLAAKYRPNSEW

              78
Pogonus        NTMAEQEFYLDNNGF--------PEQE-----------PE---ERFKRA------GNGPSLSIVNPIDVLRQRLLLE
Ignelater      NGVTDPRFYVLTEME-SNDIDY---QEPL--------AGEG---RIKRQGL----NNGPSLSIVNSLDVLRNRLLLE
Photinus       NGVKDPRFYVLTEAE-SNDIDF---QEPL--------IGEG---RIKRQGL----NGGPSLSIVNSLDVLRNRLLLE
Aquatica       NGVKDPRFYLLTEME-KNDIDY---QEPL--------IG-----RIKRQGL----NNGPSLSIVNSLDVLRNRLLLE
Nicrophorus    QWMAPYY---------ADSNNNNELD---F----------GQEARNKRA----GLIDGPSLSISNSLDVLRNRLLLE
Aleochara      AYYADLD---------ANTIDTIPQQLPQFQRTLESQSQEDDRDKRKRAGLSLREVNGPSLSISNSLDVLRNRLLLE
Oryctes        DDSRYYD--IPTDYQ-LNTIDEIPFSQ----------EGRIKRANFNDKPSSVKQFRGPSLSIVNSLDVLRNRLLLE
Coccinella     RRLGGTGLYLVTDDL-NTDSER---LN-----------PET---RIKRAEF---MRPRGSLSIVNSLDALRNKLVME
Harmonia       RRLGDPSVYLMSDDL-NSDSER---LRDV--------PSET---RIKRAEF---LRPRGSLSIVNSLDALRNKLVME
Dendroctonus   RGVSDPKFYVLTEME-SNDIEN---QEPT--------AEEDLNLRTKRQSRIG-RNAGGSLSIVNSLDVLRNRLLLE
Hypothenemus   RTVTDPRFYVLTEMIGSNDIDN---QIGY--------FDENA--REKRQS SRIGDAGGSLSIVNSLDVLRNRLLLE
Anoplophora    SGVTDPRFYVLTEME-SNDIDN---QNDL--------GLDS---RIKRTGR--LGEQGGSLSIVNSLDVLRNRLLLE
Leptinotarsa   AGVTDPRFYLLTEME-SNDFDN---QETS--------NLET---RTKRAGR--IRESGGSLSIVNSLDVLRNRLLLE
Aethina        TGVTDPRFYLLTEME-SNDIDN---QNNL--------SPEA---RSKRAGR--LGEPGASLSIVNSLDVLRNRLLLE
Hycleus        SGVTDPRFYVLTEME-SNDIDH---QNGL--------RS-------KRAGGIGGGEAGASLSIVNSLDVLRNRLLLE
Tenebrio       SSVTDPRFYVLTEME-SNDIDN---QNKL--------GPEL---RSKRAGA--LGESGASLSIVNSLDVLRNRLLLE
Tribolium      SGVTDPRFYVLTEME-SQDIEN---QNKL--------SPES---RSKRAGA--LGESGASLSIVNSLDVLRNRLLLE

             155
Pogonus        IARRQMRESQKQVAENRKLMKTIGKRGRFLRQPHDMRRPNPLGFDNM---
Ignelater      IARKKQLEG---MNRNRNLLSSVGKRATFGHRGALG YDNNL--------
Photinus       IARKKQLEG---MNRNRNLLSSVGKRAILGHRGLVP-TFDNF--------
Aquatica       IARKKQLEG---MNRNRNLLSSVGKRAVFGHKDVIA-GYDNF--------
Nicrophorus    ISRKKALLG---ANYNRNMLCRIGKRFEN-NKRRCE-DSEKYVEDRFMV-
Aleochara      MSRKKELEG---ASRNRNILCKIGKRAFD-GKRRCE-EPEKYVDDKLGEA
Oryctes        ISRKKALEG---ANRNRNYLCKIGKRSPLFDQKQCE-DDNIKAFL---P-
Coccinella     MARKKTQQN---AERNRQFLQSFGKRSYAVHR NID-RFNNM--------
Harmonia       IARKKTKQN---AERNRQFLQSFGKRSYPVHRRSMD-RFNNM--------
Dendroctonus   IARKKAKEG---ANRNRQLLMNFGKRAFIQRIPRAY-FQRQI--------
Hypothenemus   IARKKAKEG---ANRNRQLLMNFGKRAYLPTRRLFV-NRKI---------
Anoplophora    IARKKAKEG---ANRNRQMLMNLGKRAFLQR PAGV-YDNKM--------
Leptinotarsa   IARKKAKEG---ATRNRQMLMSLGKRAYFSRRLYGD-DE-----------
Aethina        IARKKAKEG---ASRNRQILMHLGKRAFYHR NTGI-FDNKV--------
Hycleus        IARKKAKEG---ATRNRQILLSLGKRAFLQSRNNGR-YDNNV--------
Tenebrio       IARKKAKEG---ANRNRQILLSLGKRAFLQSRASGT-YDNNV--------
Tribolium      IARKKAKEG---ANRNRQILLSLGKRAFLQSRASGT-YDNNV--------
```

Figure S26. Alignment of DH47 precursors.

```
              1
Coccinella    -----MRWSIYLACAALVISIKADEN---------SVDY--YGSFLEPINIAAEQETLNNYLLP
Harmonia      -----MHWPVYLAFAVLVIATKAEDN---------SADY--YGSFLEPINIAPEQETLNNYLLP
Dendroctonus  -----MRAPIYLVCAALVVAV--RS-------EP-NENY----MGLESVNVAADPETI-NYLLP
Hypothenemus  -----MRAPLYLVCAVLAWTA--HGD----DQGQ-QQNY----MDLEPVDVAADPETV-NYLLP
Anoplophora   MGAHIMRMPVYLVCAAALVAV--KGE----E----GTNY--FGRQLEPVDVAADQETI-SYLLP
Leptinotarsa  -----MRAPLFVLCAALVVAI--RSE----DTSAVNTNF--FNRQLEPINMAADQETI-SYLLP
Aethina-1     -----MRASFNLLCAATLMFALASCAELNNEATGTGNYYGDAGRSLEPLNVAADPETI-NYLLP
Aethina-2     -----MRASFNLLCAATLMFALASCAELNNEATGTGNYYGDAGRSLEPLNVAADPETI-NYLLP
Hycleus       -----MRVPLYYLLWAILIVTVKTNG----ET---SNYY--GGRFLEPIDVAADQETV-SYLLP
Tenebrio      -----MRVPVYLVCAALV-VA-VKSE----DH------N--YGRLLEPIDVAADQETV-SYLLP
Tribolium     -----MRVPVYLVCAALV-VV-VRSE----ER---TNYY--GGRYLEPVDVAADQETV-SYLLP

             65
Coccinella    KVQPKYKP--EWRRLGGTGLYLVTDDLNTDSERL-APYRPNLRKRYYESYIDD-------RNVR
Harmonia      KIQPKYKP--DWRRLGDPSVYLMSDDLNSDSERL-EPYRSNIHKRYYESYVDD-------RNVR
Dendroctonus  KLAAKYRLNSDWRGVSDPKFYVLTEMESNDIENQTPEQPRRIRNKRYNNY-------------S
Hypothenemus  KLTAKYRPNNEWRTVTDPRFYVLTEMIGSNDIDNQGRKPKRIRSKRY-NY-------------T
Anoplophora   KLAAKYRPSNDWSGVTDPRFYVLTEMESNDIDNQ-ITKPKKIRNKRYNYDLN-----------R
Leptinotarsa  KLAAKYRPNSDWAGVTDPRFYLLTEMESNDFDNQ-VANNRRIRNKRYDFDVYEPRQVRYDFGEE
Aethina-1     RLTAKYRPNNDWTGVTDPRFYLLTEMESNDIDNQV-SHIRK-RAD-----------------DR
Aethina-2     RLTAKYRPNNDWTGVTDPRFYLLTEMESNDIDNQN-HMGGKLKKR-----------------QN
Hycleus       KLTAKYRPNSDWSGVTDPRFYVLTEMESNDIDHQ-V--GRNVQRR------------------S
Tenebrio      KLTAKYRPNNEWSSVTDPRFYVLTEMESNDIDNQ-MPSERSIQRR------------------S
Tribolium     KLAAKYRPNSEWSGVTDPRFYVLTEMESQDIENQ-VPSERSIQRR------------------S

            129
Coccinella    PSLSIKAPIDALRKAMLHKNWERNVQKNRELLQRL-----
Harmonia      PSLSIKAPIDALRKAMMYKNWEKNVQRNRELLQRL-----
Dendroctonus  PTLSVQAPIQALRNLYDRSNYQQRISQNRRLINQLNSS--
Hypothenemus  PTLSVQAPIQALRNLYDRSTYNQRAFQNRQLIRQLTSS--
Anoplophora   PSLSIQAPIQSLRNAYAKEKLRQRLEDNRRFLANLVDIQ-
Leptinotarsa  PSLSINANIQNLRQAYAKTKLRERLAANRKFLENLRDSQ-
Aethina-1     AVLSIQAPVRALYDSMQKNKMRKLLLANRQLLNNLRDQYN
Aethina-2     HVLSFQEPRHMLNRHLEKQKHERQSELNRNFLNNLVDYY-
Hycleus       PTISIAAPIDVLRKTWQQEHARKQMERNREFLSRLT----
Tenebrio      PTISITAPIDVLRKTWEQERARKQMVKNREFLNSLN----
Tribolium     PTISITAPIDVLRKTWAKENMRKQMQINREYLKNLQ----
```

Figure S27. Alignment of DH37 precursors.

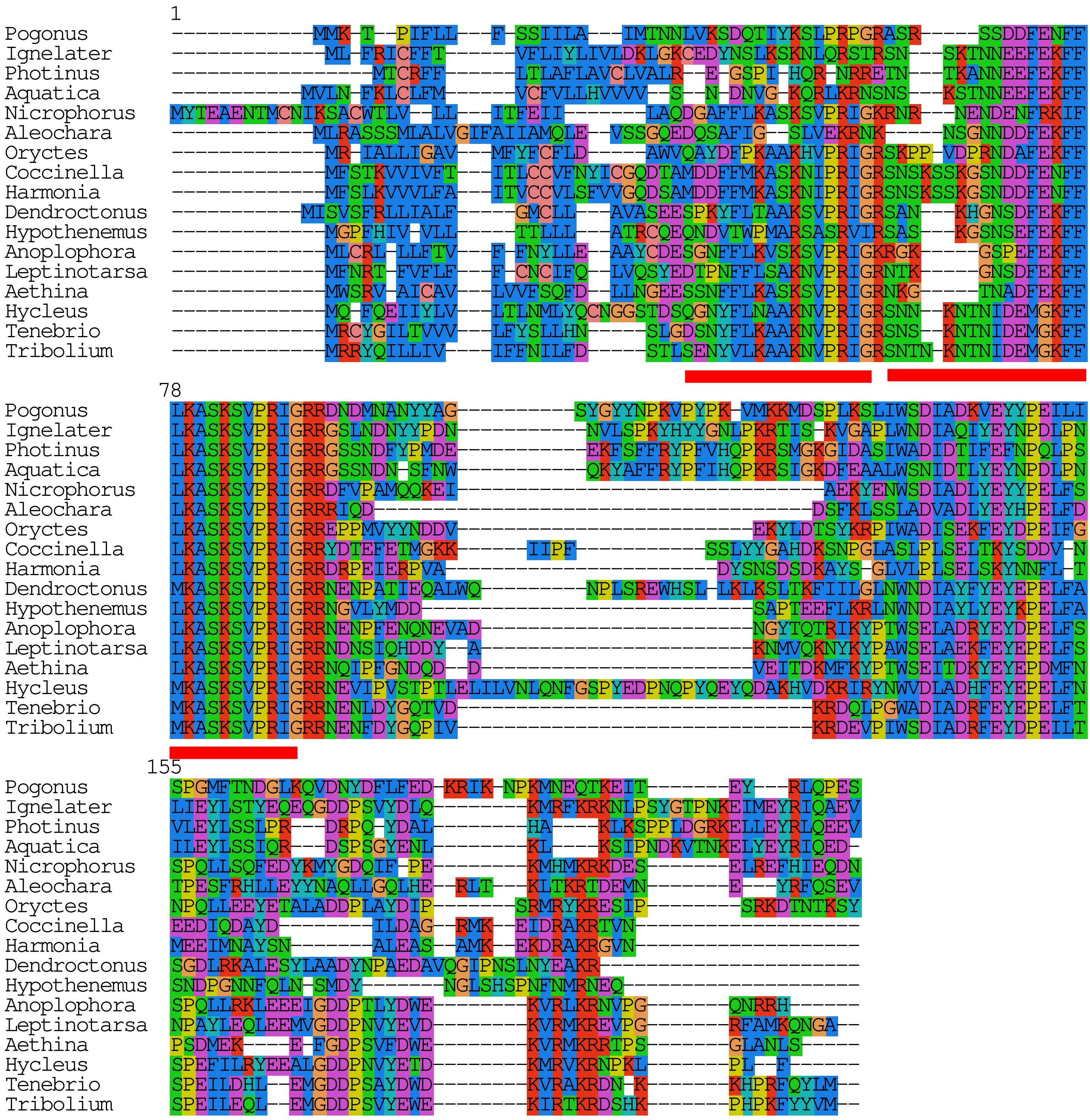

Figure S28. Alignment of ETH precursors.

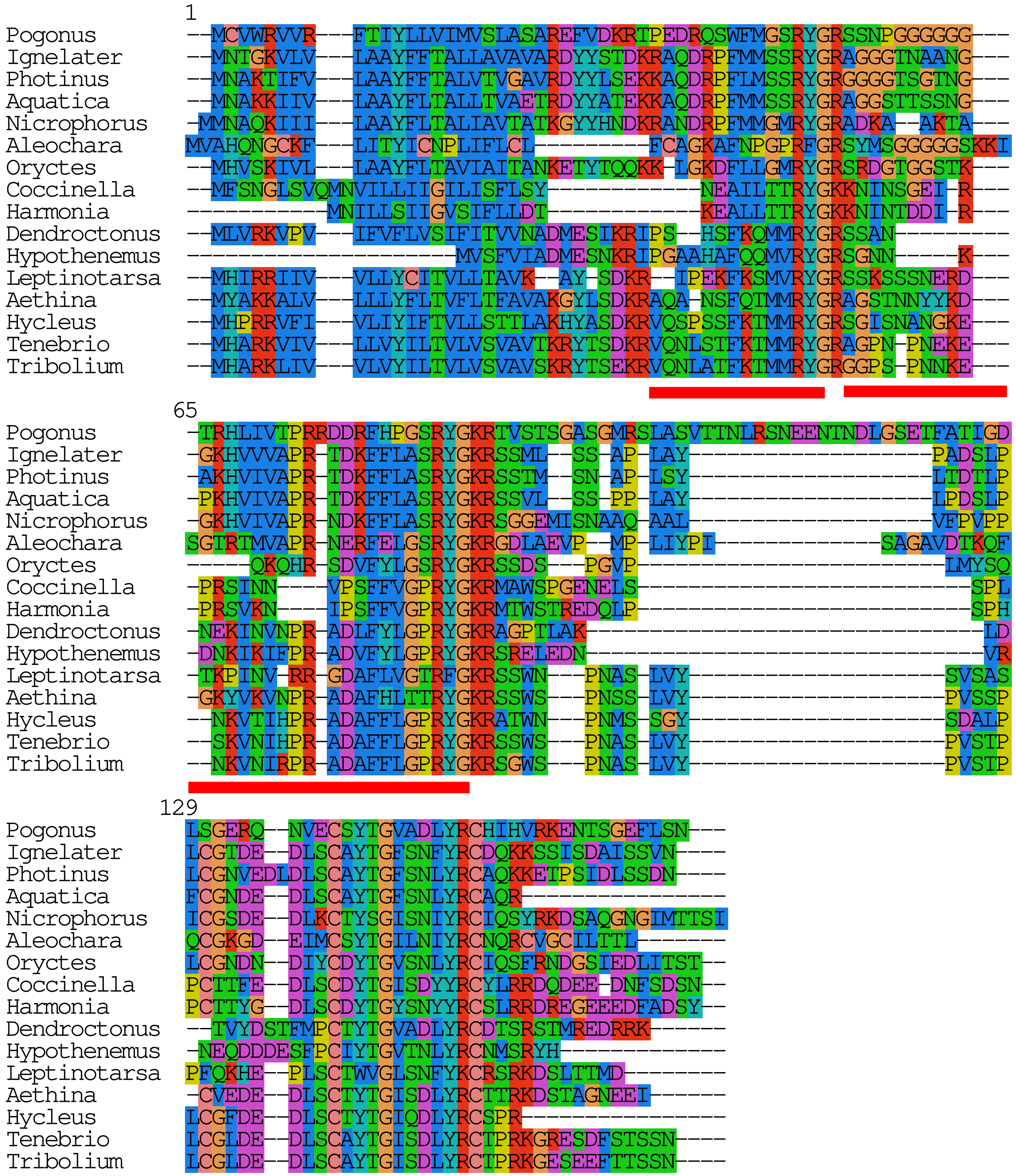

Figure S29. Alignment of RYamide precursors.

```
Pogonus       MGCRNILRIILLTIIIC-VLLSHRQHTVKGTP-T--NIGDNNHQTRNRPN-RFGT-RLTSQYVR
Photinus      MFQARLPTGLFAISSIYLLLFLNHSQNANSFVSA-LNNMEALHR-PHRPLPAASA-RLAIHYKK
Aquatica      MFMVKGRSRVLFILSAL-ILFIIHNQNVNSA--T-AAAIDGSHR-QHRPIPRVSA-TIPLRFNK
Ignelater     MSSIRILTGILIILSAF-ILLLHQKQHIQGVSAA-TVNMENNHR-PHRPVPRVAA-RLPVHYSK
Nicrophorus   MSQ-TYFMMKLLLVAMC-LILMACNDGASAGPAGASVPTEANRRIRSRPFLRVSP-RTIGQYAR
Aleochara     MICKGIIRS-FLLCFIF-LVVCNHMQRVSAAASANVQPNENSHRLRPRPYLRIPQ-RAFNQYAR
Oryctes       MVVHKIFTVFVIIFFVY-TLKLDNSQFVAGA--PSTNTLESIHRHRSKPLLRLLP---KSQYPK
Coccinella    --MAKVMTAIFFILSVY-LLFIHQLHIVSSAIPSY-----------SNRLEKLSLGKFKNRRIN
Harmonia      --MAKIMTGIFCILSIY-LLLIHQIQIVASTIPSR---------KLFGRFDRLHLGEISNRKVH
Dendroctonus  --MKNAITSYFLIIAICFFLILCQMNHIIAESSN---GNVELP--RNRPSGLLKYPYRLVQYRR
Hypothenemus  --MNHLLTNPFVLISICLLITCHCLTITSNNNIPGGLPR-------TLSTNTVYHQRAHTQYNR
Anoplophora   --MKSFFTGMFLLTSLY-LFLTYQFGFTSAT--TGGPTNQEVHRPRARILGRFA--RPITQYAR
Leptinotarsa  MVLRSLFTGFFIVSSLY-LLLTYQFGLSLGA--PGRGQN--NHRSRNRGFGRHPI---SPQYAQ
Aethina       --MRSLLTGMFLITSLY-LLFIHQLQSV--Q--PAHVPSADLHRVRARPINRFNK-PLLNQLAR
Hycleus       ---MKISTGFFILSSIY-LICINQLTIVSAS--TGSSPNLENRRQRPRPFVRVPLGRPPVQYAR
Tenebrio      MGMKSVFTGIFIISSMY-LLFIHQFHNASAA--PGNVNNLDSHRPRGRPFTRLTP-RTSNQYAR
Tribolium     MGMKSFFTGVFLISSVY-LLFIHQFQNVSAA--PGNANNVDSHRLRARPFARLTP-RT--QYSR

              65
Pogonus       TRVEP--D---FI-LDDDDLFDLTKRQS---DDYGHMRFGKR-E--FDDYGHMRFGRSGTD---
Photinus      VHP-EL-S---DI-LDEEDLFDLSKRLV--TDDYGHLRFGKRGED-FDDYGHLRFGRGGTENK-
Aquatica      IRTETL-S---DL-FDDDDLFDLSKRLV--TDDYGHLRFGKRGED-FDDYGHLRFGRNGRDKEL
Ignelater     IRTESL-S---DF-LDDDDLFDLSKRQV--ADDYGHLRFGKRGED-FDDYGHLRFGRSGGEKK-
Nicrophorus   FKP-DLGD---FI-VDDDDFFELNKRQN--SDDYGHLRFGKR-ED-FDDYGHLRYGRSGDSSRR
Aleochara     LKADE-------F-LDDDDFFEMNKRQT--SDDYGHLRFGKR-DE-FDDYGHMRFGRSGSDTNN
Oryctes       IKA-DFSD---FI-VDDDDLFELSKRQT--SDDYGHLRFGKRGDEQFDDYGHMRFGRSGNSD--
Coccinella    QRSDFLFN---DF-IDEEDLDMTNKRQI---DDYGHMRFGKRGEDQFDDYGHMRFGRSF-----
Harmonia      PKSDFIFN---DF-IDDEDLETNNKRQV---DDYGHMRFGKRGEDQFDDYGHLRFGRSF-----
Dendroctonus  LKPHAIIN---DFDEDDDDVFNLSKRQQTNSDDYGHLRFGKR-EEQFDDYGHMRFGRNGGERK-
Hypothenemus  LKPESFFVPNDLVLLDDDDLFDLSKRLPTASDDYGHFRFGKR-DPVFDDYGHMRFGRGGAD---
Anoplophora   LKPEPYLN---DF-IDDDDLIEFSKRQT--SDDYGHLRFGKRGEESFDDYGHMRFGRSGTDQ--
Leptinotarsa  MKNEPFVS---DF-IDADDFFEISKRQQNNPDDYGHMRFGRKGEEISDDYGHMRFGRSDIENK-
Aethina       IK-EPVDA---FI-VDEDDLFDLAKRQT--SEDYGHLRFGKR-DEQFDDYGHMRFGRGGD----
Hycleus       AKSEPFGD---FI-VDDDDLFELSKRQT--SDDYGHLRFGKRGEDSFDDYGHMRFGRSGSD---
Tenebrio      IKPEPFNE---FI-VDDDDLFELSKRQT--SDDYGHLRFGKRGEEPFDDYGHMRFGRSGGDK--
Tribolium     IKAEPFNE---FI-VDDDDLFELSKRQT--SDDYGHLRFGKRGEEPFDDYGHMRFGRSGSD---

             129
Pogonus       -
Photinus      -
Aquatica      S
Ignelater     -
Nicrophorus   -
Aleochara     -
Oryctes       -
Coccinella    -
Harmonia      -
Dendroctonus  -
Hypothenemus  -
Anoplophora   -
Leptinotarsa  -
Aethina       -
Hycleus       -
Tenebrio      -
Tribolium     -
```

Figure S30. Alignment of Sulfakinin precursors.

```
              1
Pogonus       -----MWIWILLAALINQ-SFISSSAAEPIGSEYQEEAPDDSMYLYLEERSVKDN---------
Ignelater     ML----RLIIVVVLIHISSAIPMFDDS---YD--------DEPFLYMDSRPSKYR---------
Photinus      MH----RLMILILLVQLSSIEVFSEDA---YD--------EDPLTFLESHISKYR---------
Aquatica      MY----RLVVVALLILLSSLEVISEES---YD--------EDPVLFLEPFSS------------
Nicrophorus   ---MSTMLNLLFLLAALEPIWAYASPE-EGYLLPQTSGEDDDLYLYVDTRSESEQ---------
Aleochara     ----MAMITLIIGILLVQ-ALRFASTAEEAFLGYPLQTEEDEGYLYMDSRADPGY---------
Oryctes       MLAHP-ITALLFHFLIMT--TAYSANS---IYQQPSKTKGEQLFMNLRSNYDTERSTRRVFAQN
Coccinella    -----MYISALLVLVLTQYTWAYNDDN---YPFSESYDP---T-LISAEDY-ADQ---------
Harmonia      -----MYISGLLVLVLTQYTWAYNDDN---YPFSDNYDP---N-IIPFEDY-TDQ---------
Dendroctonus  ----MGLSVILMALLLLQSALSLTDDSFEGYEIFGPDEE-----------ADME----------
Hypothenemus  ----MGLSSVFLLLFLLRFAVSINDDSIEGFELFVPEDE-----------SDLE----------
Leptinotarsa  -----MLPIFLFIFFLVQSSFANLEES---YHETPDSYS-----YYPDDLDSDD----------
Anoplophora   -----MIIIPIISILLIQSSLAFLEDS---YPETFDSNTY------PEDLFDND----------
Aethina       MFFF-----PLVILFLVQSTWGYVDET---YYPGENVDNY----LYSEEVTENEN---------
Hycleus       MSPIPLICIILLLLHHHHTVWCYNNDD---LYQQFPPDPYDSI-YYSIE-DYFDE---------
Tenebrio      ---------------------------------------------------QPPP---------
Tribolium     MVPF----AILILTLIIQLASGYNNED---FYSDNF--------------DGFEE---------

             65
Pogonus       -----------T---------------DDIEARRRSAL--------DKNFMRFGRGGNTNDLY-
Ignelater     -----------PS--------------R--------LLQDKDA------FYRVSRAHEDSYA--
Photinus      -----------PY--------------K--------TFHDKDRGFPDFNYLRYSRGRSESIL--
Aquatica      ---------------------------------------------------RYTRSRSESLL--
Nicrophorus   ---------------------------RSSEPSRRSTL--------YKNFARLGRGDLPANY--
Aleochara     -----------SPSML-----------KQGETRRRSTL--------DKNFARFGRSGLGKSKLS
Oryctes       PIRFGGRRSDIPPLVGKAMEYEAMLDNEVGDRERRAFSKGNHMRFGRANFLRFGRSRSNTNN--
Coccinella    ---------------E-----------DIFEPA--EKR--------ANNIPRY-------EH--
Harmonia      ---------------E-----------DIFEPA--EKR--------SESLGRY-------EQ--
Dendroctonus  ---------------------------P--YMDRQSRG--------NDKFLRFGKAFWDYES--
Hypothenemus  ---------------------------S--YIDRQTRN--------NDKFLRFGKAFWDYSD--
Leptinotarsa  ------------------------------NEM--EKP--------NNHFLRLARSDGNFD---
Anoplophora   ---------------------------NDIEVQ---RR--------SNHFLRFGRNNGKYEN--
Aethina       -----------PNPPQ--------------RRN---NN--------NNNFLRFGRSEK--KY--
Hycleus       -----------PDEIY-----------RHFY-N---DK--------NDNNDRIATIIKRHDN--
Tenebrio      -----------PPPAS-----------SVLTPL---LR--------GGYHRRVRG--T--NY--
Tribolium     -----------PSEEV-----------SDMEVR---RR--------NSNFLRFGR--S--GP--

            129
Pogonus       -LQK--HILDPDDVDYNDE---------YNRDQR---TSNKNGNFIRFGRGKAGDFIRFGRNPT
Ignelater     PLI---DEIDTEFEDFNDE---------FARPTRS---QDKNDNFVRFGRGN-SNFVRFGRDSY
Photinus      PFM---DDLESEYDD-EEQ---------FARPTRS---QDKNDNFVRFGRGN-SDFIRFGRDPY
Aquatica      PLM---DDLESEYDD-DDQ---------FARPTRS---QDKNDNFVRFGRGN-SDFIRFGRDPY
Nicrophorus   --------------EMEEG---------YDRPTRV--LGDKSDQFIRFGRGN-SDFLRFGRD--
Aleochara     YLNEDNRLKDMMYMDEDADETLTNNGDSGRFLERNSRSGERNDQFIRFGRGK-SDFVRFGRADS
Oryctes       -------------NSNRND---------YNRLTR-----TNNSQFLRFGRGR-SDFLRFGRSEE
Coccinella    ---------R---RSSNDP---------DPRPTRGAKM-LRSDYFVRFGRSK-QDFLRFGRDPN
Harmonia      ---------R---RSSNDP---------DPRPTRGAKM-LRSDYFVRFGRSK-QDFLRFGRDPP
Dendroctonus  --GN--E-----APYSDKA---------DQRPPRTG---------------------SLPDNPQ
Hypothenemus  --AG--N--DAAAMYIDKE---------APRPSRTGRQ-GNNEHFIRFGRSN-KDFLRLSGNPQ
Leptinotarsa  ---------------EYDD---------EDGPIIGKK-NLKNDHFIRFGRSSHDRCLRLGRDLE
Anoplophora   ---------NEVGNDDYED---------FARPTRSGRI-EKNDHFIRFGRGKHQEFLRFGRDPQ
Aethina       ---------DTDYEDYIED---------FARPTRSGRT-DKNDHFIRFGRAG-QDFLRFGRSQV
Hycleus       ---------SLSDLSYDSK---------FPRPTRSGAIGDKNDHFIRFGRSK-QDFLRFGRRST
Tenebrio      ---------DVEYDDFNED---------FARPTRSGKT-EKNDHFIRFGRSK-QDFLRFGRDQH
Tribolium     ---------NYEYEDYGED---------FARPTRSGKI-EKNDHFIRFGRSK-QDFLRFGRNQP
```

```
              193
Pogonus       Y-LRDIRGRDQNMIRFGRSVPLKRSK--------RQVNDM-YSQEEEKR-RTNNMLRFGR---N
Ignelater     KH------KNQDFVRFGRSISESEMP------KKRSRRDV---DESKKRNDRDNFLRFGRGGGD
Photinus      KYGREMRANDQNFLRFGRNAPENDTE------KKRSKRSV---DVQDKR-GSDSFLRFGKA-GD
Aquatica      KYNREMRGNDQNFLRFGRSVD---SN------KKRSKRSV---ETQNKR-GSDNFLRFGKS-GD
Nicrophorus   ---------KGHFLRFGRSGSTVKS--------VRNKRSA--NFEKRPE-RNSNFLRFGR---N
Aleochara     H-S-----KNRDFIRFGRSTRSIKGKPVNKQEKERKKREA----EYYKK-SGSNFVRFGRADRN
Oryctes       Y-S-------------------------------RKKRDTSDVNEEEKR-GNNNFMRFGKRYND
Coccinella    Q-Q--RFSRSRDYLRFGRSPPPPATE------NGRYKRDA--VFDPEVK-RNSNFLRFGR---N
Harmonia      H-Q--RLSRSRDYLRFGRSLPTPGE-------HERSKREA--TFEPDTK-RNSNFLRFGR---N
Dendroctonus  Q-R--SARDKSSFLRFGRSISEGSKK-------KRDKRQA-AASADALK-RHENYLRFGRT-AS
Hypothenemus  Q-R--STRDKSSFLRFGRSIDEDAKK-------KREKRA----VSETFK-RHENYLRFGRS-SN
Leptinotarsa  K-R--THD-KGFHLRFGRSIQEK----------PRSKRQA--TPDKVPK-RTDAYLRFGK---S
Anoplophora   R-R-----DKSAYLRFGKNEMPIFKE-------YRHKRDTFS--DEADK-RSDSFLRFGR---N
Aethina       R-L--HRDRD-THLRFGRSAPVQEQK-------SRAKRDT-AMQEEYKR-GGSNFLRFGR---N
Hycleus       -------DNDNNYIRFGRNVNNEDRK-------HRNKRDASSIYPDYKR-SSSNFLRFGR---N
Tenebrio      R-V--VRDRSGNYLRFGRSVPNTEEK-------VRNKRDT---YSEFKR-GGSNFMRFGR---N
Tribolium     K-A------TTNYLRFGRRN---------------------------KR-DTSNFLRFGR---N

              257
Pogonus       GNFMRFGRMPPIFKNDKDIDAQDD--QNHQNE-------SPVTREKIFPSDQQLSIPANYWPVL
Ignelater     DSFIRYGRNPDGIKINADDTLP---VPTKSN-----FIHGQSAHYPIA-INGRLLHSSPLFSIL
Photinus      DSFIRYGRNQQKTKLNLEEEFP---MPTVSRPLPPSFELEGTLRFPLV-LNDRILHKFPLLTIL
Aquatica      DSFIRYGRNKNEIKKQIDDLYP---LPTVSHPFVNRFGSDPLLYYPMI-FNEKLLHRFPLLKVL
Nicrophorus   DNFMRFGRDPEVVYNQKHNQV---------------------------------------LQML
Aleochara     DNFMRFGRNMNANYLQQPHVAS----ING-----------TDSLCKLLEQMQGRQSQALLLRML
Oryctes       EDYLEDFQGEEAKKQ----------------------------------NLHDKLENAFFLKLL
Coccinella    SNFLRFGRNKDDPMQG----VE----LTQE-------------DLKMLGRLRQ-FYESPLWRLF
Harmonia      SNFLRFGRTKEDHSQH----LE----LTQE-------------DLKLLSQLRQ-FYESPLSRLF
Dendroctonus  SNFMRFGRNLGAQGGLSEEELI---RPS-------------------LE-----SRNRHLLDYL
Hypothenemus  SNFLRFGRTLR--------------LPS-------------------YE-----SRNKYLINYL
Leptinotarsa  SNFMRFGRNHQTTPLSHNNADERLVLVNSAAI--------DHLPPRSFEELKV-PANSPLALLL
Anoplophora   SNFMRFGRDPTNYLANPGNMLS----T---------------------LELLK-MQEQTLANVL
Aethina       SNFLRFGRNPDGVLAMPTSETE----ITHAQ------------INELLGKIKQ-MHDSPLLRLL
Hycleus       SNYLRFGRDNEILGTVPSY----------------------------YNRHQN-LNRGPLVQLL
Tenebrio      SNFLRFGRNNEMTATSD------------------------------PEKVQQ-LQESPLVQLL
Tribolium     SNFLRFGRNNESS------------------------------------------YESPLVQLL

              321
Pogonus       QNFAASLRYTSGKQKCV-----DVTNCPPRQG----
Ignelater     RKIEEEMRQSN-------------------------
Photinus      KKLEEV-RQNH-------------------------
Aquatica      KKLEDEVRQSH-------------------------
Nicrophorus   MDLLRKQKEQ--QKQQQQQDEDIQA-----------
Aleochara     IDAMARNRLQNELQQLQQQQH-GFE--GEFKGHKET
Oryctes       SQISCNMDQ----INCD-----------DRLD----
Coccinella    VQLRLEGQER-----WK-----SDV-----------
Harmonia      AQLEFQGQRR-----CE-----SDV-----------
Dendroctonus  KSLLRQSDGRISHLN---------------------
Hypothenemus  RNLLRQSLENGSRKN---------------------
Leptinotarsa  AQLMARRQEQ--GQRWE-----TFL-----------
Anoplophora   AQLMLHKKEE--NKPCT-----RAA-----------
Aethina       TDLAINARQD--GKKFS-----S-------------
Hycleus       EDLIENLRKE--HGKHA-----DGSA----------
Tenebrio      SELLEHIKKG--QDKNR-----IV------------
Tribolium     SSLLKK---E--ENKQR-----IV------------
```

Figure S30. Alignment of FMRFamide precursors.

```
                    1
Pogonus             MACSFFNVLRFFWLVICIFA-VVVKPDEDPDDSKISMCELSLAQ-LLLLPP--DSMQVQALKRELFRYLEEK
Ignelater           MAYFFSPKILISCGI-LWFT-LLIGIKSEERENSLSACEIEKLLKPILSPPDERTIQAQALRQRLINLLRQG
Ignelater-s.v.      MAYFFSPKILISCGI-LWFT-LLIGIKSEERENSLSACEIEKLLKPILSPPDERTIQAQALRQRLINLLRQG
Photinus            --MRCPPKFLFCCGI-LWFL-LFGEIKSED--TPISSCEIERLLKPILSPPEDRTIQAQALRMRLMNLIRQG
Aquatica            MLCFFPSKILFCCGI-LWLL-FFDAIKSEDPESPLSTCEIERLLKPILSPPDDRTIQAQALRNRLINLIRQG
Nicrophorus         ---MAK------VPI-VLLL-AVFIIRAHCQ----EYMDLDEIFARIFCPEKATTMQAQALRHDIIGKIASK
Aleochara           MG-----TLTVGVVV---LA-LSCA-LAPQLASAQSDEEINSMLEKLFAPESESSMQSRALRREVLRRLYDA
Oryctes             --------------------------INAQEE----CDTNLENMLTSLVSPDEPQTIQLQSLRNELIRRIKEA
Coccinella          MVSGFRKPFAV-----VFLSVFIIMVQSDET----CNMDFERTLEILFAPQEQPSPQIRSLRRYLISIFHNM
Harmonia            MVSRFPLKLLL-----VFLSVYVCMVQSDEP----CNLDFERTLQTLFAPQEQPSPQIRSLRRYFISILHSM
Dendroctonus        MGNLRLINLSV-WCVLVGCALIDNKVKSDNS----CDLDIPESLRILLDPQETDSIQVQALRRQLLRKLQL-
Dendroctonus-s.v.   MGNLRLINLSV-WCVLVGCALIDNKVKSDNS----CDLDIPESLRILLDPQETDSIQVQALRRQLLRKLQL-
Hypothenemus        MGSLGLIQLSM-WCILVGFALIDNKVNSDNS----YDFDTAEALRILLLPEENDSIQVQALRRQLLKQLSNL
Anoplophora         MSFLSQ--TVVSLAA-LVLV-GSYGVNSDNT----CDIDVENTLRTLLSPQEN-SMQVQALRRDVLRKFQEA
Anoplophora-s.v.    MSFLSQ--TVVSLAA-LVLV-GSYGVNSDNT----CDIDVENTLRTLLSPQEN-SMQVQALRRDVLRKFQEA
Leptinotarsa        MAAF--EPIFISVVL-FISA-MLYEVLPDKS----CDIDIEKTLGTLLNPQDNPSIQVRALQIELLKKLEEA
Aethina             ---MRKP-IVGFWCI-LIVD-AVLLQVNSEN----CDGEIENALRKLLSPSEYPTLQLRALRRQLLHKMQQA
Aethina-s.v.        ---MRKP-IVGFWCI-LIVD-AVLLQVNSEN----CDGEIENALRKLLSPSEYPTLQLRALRRQLLHKMQQA
Hycleus             -----MGSFIVRIGV-LMVA-LIVTVKSDD-----YDFDIENALRTILTPQDYPTIQLQALRRSIFNRLLDR
Hycleus-s.v.        -----MGSFIVRIGV-LMVA-LIVTVKSDD-----YDFDIENALRTILTPQDYPTIQLQALRRSIFNRLLDR
Tenebrio            MAVFGAPKFLIGTGI-LMSA-LFFMVKSDET----CDIEIENTLRTLLSPQEFPTIQQQALRKDLLRRFQEA
Tribolium           -MLFGAPKFFFGTGV-LVFA-LIFVVKSDES----CDIEIENTLKTLLTPQDYPSMQQQALRKDLLRRFQQA
Tribolium-s.v.      -MLFGAPKFFFGTGV-LVFA-LIFVVKSDES----CDIEIENTLKTLLTPQDYPSMQQQALRKDLLRRFQQA

                   73
Pogonus             LEEDEL--------------SKRSFASLVRDGNLPYVPKRSIEALARSGYLKSATDDF--------------
Ignelater           FEEQVDNG----------IEEKRSISSLARWNDLPM--KRNLQALARAGYLRSLPDDD----------EIDD
Ignelater-s.v.      FEEQVDNG----------IEEKRSISSLARWNDLPM--KRNLQALARAGYLRSLPDDD----------EIDD
Photinus            LDEQVDNG----------LEEK-------GWDDMAT--KRSLQSLAREGYLRPS--EE----------SADD
Aquatica            LDEQIGNE----------LDDKRSISALARWNDMPM--KRNIQSLAREGYLRSN--ED----------NEDD
Nicrophorus         LAREGINEDEDLVRKVDEMNKKKESYDDDYYRMAAF-GKRNLAALARAGYIRTLPDED-------------N
Aleochara           IEREQLDDDL--------ANYKRSINSLARLGTIPG--KRNLAALAKGGYIRTIPDSSTEQ---------EQ
Oryctes             KQREELENEL--------LQYKRSLSSLARWNNMPG--KRNLESLARAGYIRTLPTTN----------E---
Coccinella          VARAEE---------------------IEGWNTYVY-PKRSLEALARAGYLHTLPDDE-----------DNQ
Harmonia            VARAEE---------------------AEGWNTYVY-PKRSLEALARAGYLHTLPDEE-----------DNQ
Dendroctonus        DCHNL-----ELAELEDAKNY-KSLASLAGWGNIPAEHKRNLEALARAGTWYKNPPEI-----------DQS
Dendroctonus-s.v.   DCHNL-----ELAELEDAKNY-KSLASLAGWGNIPAEHKRNLEALARAGTWYKNPPEI-----------DQS
Hypothenemus        LQPNV-----EFAEQPDDGNYRRTLASLDLWNDLPEEHKRNIEALARSGMACRMFPEL-----------EP-
Anoplophora         LDRLDQEEDE--------RNLKRSLSSLAAWNNLPE-HKRNLEALARAGYLKTLPDED-------------D
Anoplophora-s.v.    LDRLDQEEDE--------RNLKRSLSSLAAWNNLPE-HKRNLEALARAGYLKTLPDED-------------D
Leptinotarsa        AEKQEE------------DENKRALVALAKYNDLPE-HKRNLEALARAGYIKTLPDEE-------------D
Aethina             LEKFEE-EED--------PNLKSSLSALARWNNLPE--KRNLESLARAGYIKTLPDVE-------------D
Aethina-s.v.        LEKFEE-EED--------PNLKSSLSALARWNNLPE--KRNLESLARAGYIKTLPDVE-------------D
Hycleus             FDNGNIDV-----------DIGNDVGVIDRWN------KRNLEALARAGLVRTLPITQQDNNDDNNEHDNDE
Hycleus-s.v.        FDNGNIDV-----------DIGNDVGVIDRWN------KRNLEALARAGLVRTLPITQQDNNDDNNEHDNDE
Tenebrio            LEKANL-DDD--------MNYKRSLSSLAQWDNLPD--KRNLEALARAGYVRTLPSQD-------------D
Tribolium           LDRADD-EDE--------MNYKRSISSLAQWGNLPG--KRNLEALARAGYIRTLPNPD-------------E
Tribolium-s.v.      LDRADD-EDE--------MNYKRSISSLAQWGNLPG--KRNLEALARAGYIRTLPNPD-------------E

                  145
Pogonus             TDDDVKRSIANL--AKNGQLPTFQRPEDEQTQTKRGIESLARNGDLHGK---RK-LENLFENLYGKRNIASL
Ignelater           NDPNLKRSIQYL--VKNGQLPD-YRS----EESKRGIQALARNGELHNT---RQDLEELLDELYEKRNIGSL
Ignelater-s.v.      NDPNLKRSIQYL--VKNGQLPD-YRS----EESKRGIQALARNGELHNT---RQDLEELLDELYEKRNIGSL
Photinus            GD--LKRSIQSL--ARMNQFPV-Q-S----DDLKRSISSLARNGELINN---RREMEELLDEINAKRNIGSL
Aquatica            TDLSLKRSIQSL--ARLNQFPE-YQT----EPPKRSISSLARNGELFDG---RRNLEDIIDELYEKRNIGSL
Nicrophorus         ----GKRSIANL--AKNGQLPN-YQN----DAEKRGIESLARNGELHNK---RE-IEDLIDELYEKRNIANL
Aleochara           EEEQDKRSLESL--VRAGQMPNVAYD----AESKRGIESLARNGELHGK-----------------RNLGSL
Oryctes             --PDYKRNFMTTRSVRNTQSPV--------------------------------------------------
Coccinella          TDSNDKRSLSAL--AKNGQFPLHQFR---------DEESYKRSSGPPNSGDISKEIQKMLDDLYNN------
Harmonia            TDSNDKRSLSAL--AKNGQLPVHRLR---------DEESFKRSGTPVSTNDINKEMQKMLDDLYNN------
Dendroctonus        EDANYKRSMEVL--LKNGPAPLMPES---LENQKRGIESLARNGDFLRR---QP-FQSFLNSQDYKRNIASL
Dendroctonus-s.v.   EDANYKRSMEVL--LKNGPAPLMPES---LENQKRGIESLARNGDFLRR---QP-FQSFLNSQDYKRNIASL
Hypothenemus        QDSTYKRSIEIL--AKNGQHPLVRQP---IENQKRGIEALARNGDLHRP---PN-YQIQQGNMDYKRNLANL
Anoplophora         GDSNYKRSIANL--AKNGQLPMP--S----DGQKRGIESLARNGDLRPK---KD-IQELLDEMYGKRNIGSL
Anoplophora-s.v.    GDSNYKRSIANL--AKNGQLPMP--S----DGQKRGIESLARNGDLRPK---KD-IQELLDEMYGKRNIGSL
Leptinotarsa        ADANYKRSIANL--AKNGQLPTR--K----EDQKRGIESLARNGDLHQN---RE-MLEPQQDAYLA---RSL
Aethina             ANDSFKRSIANL--AKNGQLPHH--------DDKRGIQSLARNGEIHTK---PTSRQDQLDEELYKRNVASL
Aethina-s.v.        ANDSFKRSIANL--AKNGQLPHH--------DDKRGIQSLARNGEIHTK---PTSRQDQLDEELYKRNVASL
Hycleus             DDGNFKRSLSSL--VRNGQLPH---------QNKRGIQSLARNGELIQH---QRNNFDD-------------
Hycleus-s.v.        DDGNFKRSLSSL--VRNGQLPH---------QNKRGIQSLARNGELIQH---QRNNFDD-------------
Tenebrio            EDPNYKRSLATL--AKNGQLPT-YQN----NDSKRGIESLARNGELTTR---RE-IQELLDELYNKRNVGSL
Tribolium           EDPNNKRSLSTL--AKNDQLPTTFQN----NESKRGIESLARNGELHNR---RD-IQELLDELYNKRNVGSL
Tribolium-s.v.      EDPNNKRSLSTL--AKNDQLPTTFQN----NESKRGIESLARNGELHNR---RD-IQELLDELYNKRNVGSL
```

```
                    217
Pogonus             ARGFNYPV-----AG-KRYIGALARGGDLRYSSSDDDFDKRNIASIVRNGKRNVQSLVRTNNLPGGKRYYLF
Ignelater           ARDFNFPS-----YG-KRFLGSLAKSGDLSSRFNG---GKRNLASIMRNGKRNIASLVRSNNLPSGG----Y
Ignelater-s.v.      ARDFNFPS-----YG-KRFLGSLAKSGDLSSRFNG---GKRNLASIMRNGKRNIASLVRSNNLPSGG----Y
Photinus            ARDFNFPS-----YG-KRFLGSLARSGDLS-RFES---GKRNIASIVRNGK---------RNLPE-G----Y
Aquatica            ARDFNFPT-----YG-KRFIGSLARNGDLNGHFSG---GKRNLASVVRNGKHSAATLARTNNLPE-D----Y
Nicrophorus         ARSYSFPY-----G--KRYIGSLARSGELNRF---------------------------------------H
Aleochara           ARSYSLPP-----YG-KRYLGALMKN----------------------------------------------
Oryctes             --DGQFTY-----YD--------------------------------------------------------L
Coccinella          --------------------------------------------------------------IAR------D
Harmonia            --------------------------------------------------------------LAQ------V
Dendroctonus        ARAYSFPVGSTAGFG-KRSIGSIARNGNLPYF----------------------------------------
Dendroctonus-s.v.   ARAYSFPVGSTAGFG-KRSIGSIARNGNLPYF----------------------------------------
Hypothenemus        ARTYNFPVSNGQSYG-KRSLGSLAKNGELPFR----------------------------------------
Anoplophora         ARGFNLPS-----YG-KRSLSSLAKSGDLNYQ---------------------------------------R
Anoplophora-s.v.    ARGFNLPS-----YG-KRSLSSLAKSGDLNYQ---------------------------------------R
Leptinotarsa        VKNYNFPY-----ST-KRSLSSLAKSGDLYSR---------------------------------------Q
Aethina             ARSYNFPV-----FNQKRSLSSLARAGDLPN-----------------------------------------
Aethina-s.v.        ARSYNFPV-----FNQKRSLSSLARAGDLPN-----------------------------------------
Hycleus             ----TSDF-----YGSKRNIASLARSGDLNLG----------------------------------------
Hycleus-s.v.        ----TSDF-----YGSKRNIASLARSGDLNLG----------------------------------------
Tenebrio            ARNFNFPT-----YG-KRFLGSLVRNGDSQYS---------------------------------------G
Tribolium           ARNFNFPS-----YG-KRYLASLVRNGELKYS---------------------------------------G
Tribolium-s.v.      ARNFNFPS-----YG-KRYLASLVRNGELKYS---------------------------------------G

                    289
Pogonus             DELKRNIASLARTMGSRYTGKRAVPTAALLRQDGLMKPVDHHHYDDDSSKSSDNDNDRHENDSTDKRNIAAL
Ignelater           DEYKRNIASLARE-GGKFVGKRNV--AALLRQDNYLNERHHQPESEEENQTEK-PDNDLEETENEKRNLASV
Ignelater-s.v.      DEYKRNIASLARE-GGKFVGKRNV--AALLRQDNYLNERHHQPESEEENQTEK-PDNDLEETENEKRNLASV
Photinus            DDYKRNIASLARG-GGKFVGKRNV--AALLRQDNYVNEQNRH-DTDS---THS-PDVEGEEATESKRNLPSI
Aquatica            DEYKRNIASLARD-GDKFVGKRNV--AAMLRQDNYLNEQLRH-QSEDYTHTEK-PVATDEVNNESKRNLASI
Nicrophorus         -NDKRNVASLARG-GNLLYGKRNV--AALLRQDKIHGPNDRS-----YDDMMK-SDAERDSGNGDKRNLASI
Aleochara           --------------SGGFITKRNV--AALLRQDKIHGPNDRSSHMDESTGLGL-SLGKHSVDQGGKRNVGAA
Oryctes             PMYKRNVASLARS-GMKINGKRNV--AALLRQDNYLNSIREGRG---DLQ-----TERPSENYEDKRNIASI
Coccinella          GNTKRNLASIARD--GGFAGKRNA--AALLKNDRYLSHML--------------------NQVDSKRNIASL
Harmonia            ETTKRNIASIARD--GGFLGKRNV--AALLKNDRYLSNML--------------------N---GKRNIASV
Dendroctonus        -YGKRNIQSLARD-G--ALGKRSA---------------------------------DYMERPADKRNIQSI
Dendroctonus-s.v.   -YGKRNIQSLARD-G--ALGKRSA---------------------------------DYMERPADKRNIQSI
Hypothenemus        -YGKRNIQSLARD-G--VIGKRSI---------------------------------DSDDSPLDKRNIQSL
Anoplophora         TQYKRNIASLARD--GKFTGKRAM--PAL-REDEYSVSLH---------------RLPQAEGMEEKRNLQSI
Anoplophora-s.v.    TQYKRNIASLARD--GKFTGKRAM--PAL-REDEYSVSLH---------------RLPQAEGMEEKRNLQSI
Leptinotarsa        SSAKRNIAALARE-GM-IPGKRYG--GSQI----YYNS-----------------EIPFN------------
Aethina             -FYKRNIAALARD-GA--LGKRLE--DSYF---------------------------TEDGTENIKRNIASI
Aethina-s.v.        -FYKRNIAALARD-GA--LGKRLE--DSYF---------------------------TEDGTENIKRNIASI
Hycleus             ---KRNIGALARD-GIRVNGKRNLP---------------------------------------------SI
Hycleus-s.v.        ---KRNIGALARD-GIRVNGKRNLP---------------------------------------------SI
Tenebrio            ---KRNIASLARE-GGRFVGKRNV--AAMLRQDNYLNGQK-----------SN-EKVEGPELDNEKRNLASI
Tribolium           ---KRNIASI--------------------------------------------------------------
Tribolium-s.v.      ---KRNIASI--------------------------------------------------------------

                    361
Pogonus             KA-QMKS--SKFKRST-------ENENETSSTRSKRQADYWD-PSEEYPVPVYQNNNV-FDYEELIHSL-T-
Ignelater           KA-GYK---SKFKRSI-------PYVEMARGGRNKRQADYYDFQNEEYPQPVYQNQNNNMDYEELMHAI-N-
Ignelater-s.v.      KA-GYK---SKFKRSI-------PYVEMARGGRNKRQADYYDFQNEEYPQPVYQNQNNNMDYEELMHAI-N-
Photinus            KA-GYK---PKFKRST-------PYTGDY-TLRSKRETDYYDGSNEEYLQPVYQNQNS-REYDEILQAL-A-
Aquatica            KA-GYK---PKYKRSA-------PFTD-----RNKREADYYDVPNEEYIQPVYQNQNN-REYEELLNAL-Y-
Nicrophorus         KA-GYK---QPFKRSV-------ET-------RKKRQADYYE-INDEYASPVLQNQNV-FEYEELIKAL-T-
Aleochara           KANGYKP---PSKRSIHLPFLQEPSQEEFYGLKPKRDI---DYFTDEYAMPVYQNQNE-HDYEEMMKAF-T-
Oryctes             KA-QYK---PKFKRSV-------VDD-----KRAKRETDYYDMVNEEYPSPVYQSLFD---YSDD-KDL-D-
Coccinella          KA-SYK---PRYKREL-------------------SNADDYE-------DEEYQNIQNVDGYEKLVEELL--
Harmonia            KA-SYK---PRYKREM-------------------NDLDYYE-------DEDYQNLQNIDRYEKLVEEIL--
Dendroctonus        KAQ----------------------------QRNKRQADYFENE------LVYQMPA---DYEDILQDLAAS
Dendroctonus-s.v.   KAQ----------------------------QRNKRQADYFENE------LVYQMPA---DYEDILQDLAAS
Hypothenemus        K------------------------------QRNKRQADYFENE------MVYQIPI--TDYEDYLQEL-SS
Anoplophora         KA-QYK---PKFKRSA-------DETE----KRNKREADYFDVENGEYPSPVYQSPNI-YDYEDWLQDL-T-
Anoplophora-s.v.    KA-QYK---PKFKRSA-------DETE----KRNKREADYFDVENGEYPSPVYQSPNI-YDYEDWLQDL-T-
Leptinotarsa        ------------------------E----------LERMNNHYENGHYFSPNDQNIDY-N----FIQDL-NA
Aethina             KA-QYN---PKFKRSA-------SRT-----------KREIDYDNLEYSSPVYQNTNV-FDYEEMIKEL-T-
Aethina-s.v.        KA-QYN---PKFKRSA-------SRT-----------KREIDYDNLEYSSPVYQNTNV-FDYEEMIKEL-T-
Hycleus             KA-QLLPPNRQFKREL----------------NTNRQKRQIDYDNTEYPSPVYQNQNV-YDYEELMKAL-T-
Hycleus-s.v.        KA-QLLPPNRQFKREL----------------NTNRQKRQIDYDNTEYPSPVYQNQNV-YDYEELMKAL-T-
Tenebrio            KA-QYS---GKFKRAV----------------RSKRQTSYYDDEGGELPSPVYQNQNV-DDYEELVKAL-T-
Tribolium           KA-QYP---G-----T----------------RSKRQASYYDGEGGEFSLPVYQNQNV-DDYEELVKAL-A-
Tribolium-s.v.      KA-QYP---G-----T----------------RSKRQASYYDGEGGEFSLPVYQNQNV-DDYEELVKAL-A-
```

```
                    433
Pogonus             -GEYPNQEKRFLGAVARQGGWYRSP--------SRFR-PPSKRHIGSLARLGWLPAFRTARRFNRSGRSANE
Ignelater           -GIYPNAEKRFLGSVARS-GWPSSL--------VKYR-NPDKRHIGSLARLGWLPSFRTARRFNRSGRSTNY
Ignelater-s.v.      -GIYPNAEKRFLGRLPQM-GKPKTT--------PTPK-VRRRRISGFY------------------------
Photinus            -EAYPNNDKRFLGSVARS-GWPSSL--------VRYR-NPDKRHIGSLARLGWLPSFRSARRFNRSGRSTNY
Aquatica            -GMYPNNEKRFLGSLARS-GWPTSL--------LKYK-NPDKRHIGSLARLGWLPSFRSARRFNRSGRSTNY
Nicrophorus         -GEYPNTDKRFLGSVAKS-GWFRPSAS------SRFTSRIDKRHIGSLARLGWLPSFRSVRRFNRSGRSSN-
Aleochara           -GEYPA-EKRFLGSVAKS-GWFRPR--------------IDKRHIGSLARLGWLPAFRQIRRTSA-------
Oryctes             -DEYQSFDKRFL---AKS-GWFRQS--------NRFV-PPNKRHIGSLARLGWLPSFRTARRFNRSGRSSSS
Coccinella          -EGQR-AQKRFLGSIAKS-GWYDSPRS----SSMVR-SSPGKRHIGSLARLGWLPTIRNVRRFSRSGRSSS-
Harmonia            -EGQQQPQKRFLGSIARN-GWYNSPRS----SSIRSTSNEGKRHIGSLARLGWLPTIRNVRRFSRSGRSSS-
Dendroctonus        QDAYPVEDKRFLGSVAKS-GWFRPV----SRSYSSWVSLPEKRHVAALARLGWLPTYRSVRRFNRSGRSYLR
Dendroctonus-s.v.   QDAYPVEDKRFLGRIPQM-GKPKTTAIPKSRRYH--------------------------------------
Hypothenemus        QGSYPVEDKRFLGSVAKS-GWFRPT----SQGYSSFVSLPGKRHVAALARLGWLPSYRSLRRFNRSGRSYLV
Anoplophora         -GAYPNAEKRFLGSIART-GWFQPSNS----RYSRFV-SPDKRHIGSLARLGWLPSFRNVRRFNRSGRSTTS
Anoplophora-s.v.    -GAYPNAEKRFLGRLPQM-GKPKTTPA----PKSRRY-H---------------------------------
Leptinotarsa        AQGPSNYEKRFLGSVAKT-GWFQSSLS--SAKDPEFF-KTEKRHIGSLARLGWLPSLRQIRRFNRSGRSTSA
Aethina             -GEYPEPEKRFLGSVART-GWFRPATS----RMSRYN-SPDKRHIGSLARLGWLPIRRYERGFNRSGRSTNL
Aethina-s.v.        -GEYPEPEKRFLGRLPQM-GKPKSTVS----PKVRFP-----------------------------------
Hycleus             -GVYPNTEKRFLGSLARS-GWFRSTAP--STS-RYYQNLPEKRHIGALARLGWLPAFRNVRRFNRSGRSSIY
Hycleus-s.v.        -GVYPNTEKRFLGRLPQM-GKPKTTPP--PKT-RFH------------------------------------
Tenebrio            -GIYPNTDKRFLGSVAKT-GWFRPT--------SRYR-SPEKRHIGALARLGWLPTLRNVRRFNRSGRSTRG
Tribolium           -AAYPNNDKRFLGSVARS-GWFRPN--------SRYR-SPEKRHIGALARLGWLPTLRNVRRFNRSGRSTTL
Tribolium-s.v.      -AAYPNNDKRFLGRLPQM-GKPKTT--------P----SPKTRFH---------------------------

                    505
Pogonus             PVLTEYIDDNVRSYPADGSAEIDTQLSEEPLDEAAKRYLLLPAIDNILLRKLYSNTQNSNQD--LL------
Ignelater           EEC-----GVCRSAEDV-------------------------------------------------------
Ignelater-s.v.      ------------------------------------------------------------------------
Photinus            EEC-----PPCRETTSDGEAEDDPFAQN----AENKRFLLIPAANHLLLRKFPMHTFHN-------------
Aquatica            GEC-----GICRETAADGEAEDDSFSES----PEDKRFLLIPAANSMFLRKLPVRS----------------
Nicrophorus         -EC-------YREASADGQTKEYAFP-EDKLPVDDKRFLLQPAVDRILLQRVLMQPRNH-------------
Aleochara           ---------------PDGQVEDYPVAEDHL-QVIPARHFGPP-T-GLLLDDF-----SDNVEGYIVQRAVQS
Oryctes             CDS-----RF-------------AFPYSR-------------------------------------------
Coccinella          --------RSCREASSDGQNQDYFIT-DNAVPLDNKRFLPVPAMWKVPLQTFNY------------------
Harmonia            --------GRCREASSDGQDEDYFLT-DNT--VDNKRFLPVPAMWKVPLQTFNY------------------
Dendroctonus        SN---TEDIVCRSSQ---------------------------------------------------------
Dendroctonus-s.v.   ------------------------------------------------------------------------
Hypothenemus        TT---GDDNLCRKDSTNGKTEDDSYSKGPTIPLENKRYLLQPAVDNILLRKVFRHPRTIF------------
Anoplophora         DDI-------CREVAPNGQTEDYASAKEPPLSLEDKRYLLQPAVDNILLRKVFMHPRSH-------------
Anoplophora-s.v.    ------------------------------------------------------------------------
Leptinotarsa        ENT-------CRTITSDGQTKVNTNPQHPLITLLDARQKYQPKENVFYLRKMFHHPRSH-------------
Aethina             -DE-------CRALTADGEAEIDSFAKS--------TFPLNIPINNLMVRSHPRHPNAL-------------
Aethina-s.v.        ------------------------------------------------------------------------
Hycleus             NINNIDDNEICR------------------------------------------------------------
Hycleus-s.v.        ------------------------------------------------------------------------
Tenebrio            -EG-------CRETSSDGQAEDDAVA-ENSLSLGDKRFLLQPAVDKILLRKIFMHPRTHPFLSDLS------
Tribolium           -EG-------CREAATDGQTEDDAFA-EDQISLEDKRFLLQPAVDQILLRKIFMHPRTHPAIN---------
Tribolium-s.v.      ------------------------------------------------------------------------

                    577
Pogonus             --
Ignelater           --
Ignelater-s.v.      --
Photinus            --
Aquatica            --
Nicrophorus         --
Aleochara           RA
Oryctes             --
Coccinella          --
Harmonia            --
Dendroctonus        --
Dendroctonus-s.v.   --
Hypothenemus        --
Anoplophora         --
Anoplophora-s.v.    --
Leptinotarsa        --
Aethina             --
Aethina-s.v.        --
Hycleus             --
Hycleus-s.v.        --
Tenebrio            --
Tribolium           --
Tribolium-s.v.      --
```

Figure S32. Alignment of NPLP1 precursors; s.v., splice variant.

```
               1
Pogonus        MMQYAM--------KALAGGLVWAALVSCYCAQATESHDGEHGS------MHMKTPQQDDLQMDSGD-LQMVKRAWN
Ignelater      -MRYAA--------SASVGTLVSAALLACCLKASYTVASSDDPS------MVPKSLED---SNSQLE-TSMSKRDWN
Photinus       -MRYVV--------SASAGSLVWAVVFLFCLKDSYILAASEESP------LSQKSDDD---LNSQLE-SSMLKKDWN
Aquatica       -MRHVV--------SVSVGTLLWTV-LIFSLKARYTCAASEEGL------LGQKPDDD----TSQLE-GSMIKKDWN
Nicrophorus    -MRSAV--------LVIVGAIVCISMLPFSMQAAIDVGSDPDIG------IPK----------ESDE-MQMEKRDWN
Aleochara      -MTYAM--------LSTSRTLLGVAAIFCCLKIGMAVSGNDVGA-------P---KAE---LDPNDQ-MDMEKRGWT
Oryctes        ----MM--------SSNVGKLLWAALLACYLHAGYSIAVSDDSS------LAKS-SDD---TNQQME-SAMNKRDWH
Coccinella     -MRYVF--------ASASHKFLGAAVLAFYLQVNFAYTLPDDAT------ILLKSEDD----NLQTD-TDMVKKNWD
Harmonia       -MRHFF--------ASISHKLLGAALFAFYLQASFAYTLPEEEA------V----DDN----LKQMD-SDMMKRNWN
Dendroctonus   ----MFG-LWPMLRMRICDIFLYSMLIAICLEAAVLA-DV----ANDVPMPPRKSLEE----NPQLE-NDYSKRGWK
Hypothenemus   ----MIG-RWPT-------YFLYVTAYVTCLRAAAISNDV----PMIMAAVPQKSLNE----NPQVD-NDELKRGWK
Anoplophora    -------MRYVPTPTVSLGKMFWAALLACCLQISVVVLADDS--------SAAKSGDE----NLQAEG---------
Leptinotarsa   --------MRYQSTNVNSAKILWATLLACYLQACLTFVVADN--------ADSRGQDD----NLQVDGDEVNKRDWN
Aethina        -MRHVSGVLVAGGQSGGGSIWLWSVVLACCLEAALLPAVVAEDIAPVAPISRSDAIDD----NPQL--NEISKRDWH
Hycleus        -MRDAT-IHSTTTKTASSTRFLCVIFLIYYMEIGLVAALSDE--------IPLQSNND----NPQVD-DEMSKRDWN
Tenebrio       -MKDAV--------AAVAAKMLGLVLLACCLQASITRALSDE--------TPMKSTND----NPQMD-DEMSKRDWN
Tribolium      -MRDAV------------APVLGAVLLTC-YSLQATLALSDE--------TPLKSSND----NPQIE-DEMSKRDWN

              78
Pogonus        SM---PQGAWG--KRAWNNMGATWGKRLA--------N--SYGAADKRAWQKFQGGWGKRWGPEDEW--SLSEN---
Ignelater      R----NLHSWG--KRAWQNLQGGWGKRD-----SSYPL--YYPYADKRAWQNLQGAWGKRLAPEDEY--AMKQLASL
Photinus       R----NLHSWG--KRAWSNLQGGWGKRD-----SSYPL--YYPYNDKRAWQNLQGGWGKRYSSDDEY--AAKLA-AL
Aquatica       R----NLHSWG--KRAWSSLQGGWGKRD-----SS-----YYPYNGKRAWQNLQGGWGKRYAPDDEY--ASRLA-AI
Nicrophorus    R----NLHSWG--KRAWQNLQGGWGKRDLWGPSSSFPL--YV--TEKRAWQNLQGGWGKRFAPEDEY--AMKQLATL
Aleochara      KGNPSMMHSWG--KRAWQNLQGGWGKRDLWGPSNSYPL--YI--TDKRAWQNLQGGWGKRFATEEEY--AAKLAALL
Oryctes        K----SLPLWG--KRAWQSLQGGWGKRDAWDDS-------LYPAAEKRAWQNLQGGWGKRYAPDDIY--TLRLAAAL
Coccinella     K----NLKMWG--KRAWSNLHGGWGIKRSIPEDSEQD----IVIADKRSWENLRSGWGKRNDFMEDV---LSQEPVM
Harmonia       K----NLKMWG--KRAWSNLHGGWGVKRSISDDFGQD----DLVTEKRAWENFRNGWGKRRVPLEDL---TPQELSL
Dendroctonus   T----DLPMWG--KRGWSNLHSGWGKRDSDSDEEL-DG--YPSGMEKRAWQRLQGGWGKRYIPTEEDMV-IRQLA--
Hypothenemus   T----DFPLDGTNKRGWNNLRTNWGKRDIGDDD-L-D------LYQKRSWQKLQGSWGKRTYPSDEDDD-MVHQWPV
Anoplophora    ----------L--KRQWERMQGGWGLKSTLPLNDQIG-Y-STPDREKRAWQNLQGVWGKRFAPSEDDL-TIQELASI
Leptinotarsa   K----ELHMWG--KRGWSSIHGGWALKNNIPSLMNQQAYPPIPIVEKRSWNNLQGGWGKRSAYEEDD--AIEDISSM
Aethina        K----ELKIWG--KRAWNGLHGGWGKRSVPNYDDQMMPYDSPSLVEKRAWNRLQNGWGKRMAPDDEL--AVNQLASM
Hycleus        K----DLHIWG--KRGWNNLHGGWGLKRSIPATTWNE---QATTPDKRAWQNFQNGWGKRFLPNDDEYAASRQLSAL
Tenebrio       K----DLHIWG--KRGWNNLHEGWGRKRSAPS--WGD---Q-PAMEKRAWQNLHSGWGKRFAPEDE-YA-IRQLAAM
Tribolium      K----DLHIWG--KRGWNNLHEGWGRKRSVPA--WEE---Q---QEKRAWQSLQSGWGKRFAPEDE-YA-IRQLAAM

             155
Pogonus        ---VD-TEPQQYDD-YAAV-APS-GGDFEDLIDEDKRAWRAIN-GAWGKRAHGWDSFRGSWGKRD--PAWNNLKGLW
Ignelater      IEQEE-QQQQQEPQ-YSEY----GLGSDSDINTDDKRAWKGLN-GGWGKRSDSWGNFRGSWGKRD--PAWTNLKGIW
Photinus       LE-----QEDQEPQ-YNEF----GNLPETDANDAEKRAWKGLN-GGWGKRSDSWGNFRGSWGKRD--PAWTNLKGIW
Aquatica       LE-----QEEQEPQ-YNEF----GPASDIDLNDGDKRAWKGLS-GGWGKRSDGWGNFRGSWGKRD--PAWTNLKGIW
Nicrophorus    LEQED-QGIQGDAP-YADY----AQITEMDANDDEKRAWKNLNNAGWGKRS-EWGNFRGSWGKRD--PAWTNLKGIW
Aleochara      AD-EE-QLQQQLAD-YNSI-IGEGDAADVENNEEEKRGWRSLNSAGWGKRA-QWGNFRGSWGKRD--PAWTNLKGIW
Oryctes        AEQEE-QEPQLT----SDL----SNDADFEPIDEEKRSWKSLSGTGWGKRS-DWTSFRGAWGKRD--PAWTNLKGMW
Coccinella     TRLE------------TDY----PDSDNLEYNEDEKRSWRQLN-HGWGKRN-KWSEFRGAWGKRRPDPAWNNLKGIW
Harmonia       LENE------------LQY----TDFDNLEENDDEKRSWNQLN-NGWGKRN-KWSEFRGSWGKRSPSPAWNNLKGIW
Dendroctonus   -AALSEDNNENTIDYYP-------DQDFDSSHEVNKRNWRGFS-DGWGKRSDKWDKFRGSWGKRQ--PAWNNLKGLW
Hypothenemus   PDPDSIANDNNVLDYYVYYFNPEQVGDFDGGHEVNKRNWRSFP-EGWGKRSNKWDKFRGSWGKRQ--PAWNNLKGLW
Anoplophora    LEQDS-KEAPP----VDD--------NEF-DIHEDKRSWDKFV-DGWGKRN-KWEKFRGSWGKRE--PAWSNLKGIW
Leptinotarsa   LEPST-RY-------KIPT----DSDDDFIVHDKVKRNWSKFT-DGWGKRS-KWDNFKGTWGKRQ--PAWSNLKGIW
Aethina        FERDP-QMEQP----YTEF----GPEAEIDGIEEEKRNWQQFN-SGWGKRN-NWNKMRGAWGKRE--PAWSNLKGMW
Hycleus        LDLDN-VNQLY----SSEY----GPDFDL-YNDDEKRNWGQFH-GGWGKRS-KWEKFRGSWGKRE--PAWSNLKGIW
Tenebrio       LEPQ-----------YDEY----NPEGDLDVNDDEKRNWGQFH-GGWGKRS-KWDNFRGSWGKRE--PAWSNLKGIW
Tribolium      LDSQ-----------YDDY----NPE--IETNDDEKRNWGQFH-GGWGKRS-KWDNFRGSWGKRE--PAWSNLKGIW

             232
Pogonus        GKRSIQDRISS
Ignelater      GKRNYPDRIAQ
Photinus       GKRSFPDRIAQ
Aquatica       GKRSYTDRITQ
Nicrophorus    GKRSPSERRSQ
Aleochara      GKRSFQDKDAQ
Oryctes        GKRSYSDRIYQ
Coccinella     GKRNYNDNVMI
Harmonia       GKRSYSEKAVL
Dendroctonus   GKRSVSE----
Hypothenemus   GKRSISE----
Anoplophora    GKRSLYQYAN-
Leptinotarsa   GKRSEPEQFLN
Aethina        G----------
Hycleus        GKRSNPDQIAQ
Tenebrio       GKRSVQDQIAQ
Tribolium      GKRSGEK----
```

Figure S33. Alignment of allatostatin B precursors.

```
             1
Pogonus      MR---HSRLICPILMAIF-SLINASENVKRAPSGFQGVRGKKSIQDTYPETAPATGNSNTASSG
Ignelater    --MRRTLL-VISIISAALGGVCAADQPKRRAPSGFTGVRGKKSIPDAAYSDTSQS------L--
Photinus     --MWRIIL-MASLLSASIEKIQTADQVKRRAPSGFTGVRGKKSIPDSTYSDTTQI------L--
Aquatica     --MWKTAL-TISLILATLDNVCTTDQTKRRAPSGFTGVRGKKSIPETAYSDTSQI------I--
Nicrophorus  ---MRVPN-ISIVLATVWCLVSTAAEEQKRGPSGFTGVRGKKSFEDEDFEMRD-----------
Aleochara    ---MRVSH--ITLLLAGICI-VTASESHKRVPSGFTGVRGKKSIPNSEYALIQQE---------
Oryctes      MKYFLFSI-RLYLAIIWTVDIAIAIEQEKHAPPGSAFASETSETTLPARNGNNHI---------
Coccinella   MRFSSTA---TFTFSLMLCL-ARSEDHHKRAPSGFTGVRGKKSIVEDPKGAIFFEDESDDEDGG
Harmonia     MRISSTA---TFTFSLILCL-ARSEDHHKRAPSGFTGVRGKKSIVENAKNAIFFEEDSDEDDAT
Dendroctonus MKSIRPFT-TWISISLLLSTCCLADEHHKRVPSGFTGVRGKKSIPDEAYSADLDNSNSIEQQTE
Hypothenemus MKFLHQFTSSITLLILIVSSKAEEEDGTKRAPSAFTEVRGKQFISDEAFNEVLNNKDADQQA--
Anoplophora  ----MTLLSIQILLFTVLCILTTKSEDHHT-FSSLTLVRGRKSRDDSIYDSINKFNYSANIL--
Leptinotarsa -----------MAFFIIVCLCMMLINATLGTPYGFVAVRGKKADPFYDISRIDMRDLPMELFDE
Oreina       -----------MAFILLLYVCIISFDSIIGTPYGFVAVRGKKANSIYDP-RQDMQELSPEIFDE
Aethina      MQTRRGLP--FLLLLTSLCWCVLAEDHHKRVPSGFTGVRGKKSIPEGAYDEQDAPEASK-----
Hycleus      MHPTTSLN-ITIVMVLICCNLCRAEDHHKRIPTGFTGTRGKKSIQYTTTDETVNN--QFNGI--
Tenebrio     MHSTTIT---TAVVLATIYVVCTAEEHHKRAPSGFTGVRGKKSIPDSAYSTGNAESDATSEL--
Tribolium    MHSTTIT---TAVVLATIYVVCAAEDHHKRAPSGFTGVRGKKSIPDSAYSTGNSDSDSIPEL--

            65
Pogonus      ---------VASAENDILDEMSHA-MEKRAPSAGFHGMRGKKIPITFDNYPYPVMSINDMMLLP
Ignelater    ------------DDLSETAGDGNA-LNKRAP-SGFMGMRGKKPFPD---------PDGFPEDMI
Photinus     ------------NDPAFSSGE-DG-LDKRAP-AGFMGMRGKKPFPE---------SNSLPEDIE
Aquatica     ------------ADPSESV-LNNV-VDKRAP-SGFMGMRGKKLFPD---------SETYNED--
Nicrophorus  -----------------------I-EDKRAP-SGFLGMRGKKPFEG---WT----SLDYPEVGI
Aleochara    -----------------------L-EEKRAP-AGFLGMRGKKPFNS---WL---------EEKR
Oryctes      -----------------DNDNTQY-LAKRVP-SGFFGMRGKKPFEG---WI----SSQEDDIYN
Coccinella   GRGEGQQVAIPEVPYQFAGRPE-GDVSKRAPSMGFVGMRGKKPWAMDSGFY-----EE--DRMP
Harmonia     K-VATNQAPLPVASLQYPSGLV-GDIDKRAPSMGFVGMRGKKPWSLDPRFY-----VEDSDRLP
Dendroctonus Y--------TPSSDTEAPASFNYI-VEKRAP-SGFFGMRGKKPWNF----L---QSSNYPEELY
Hypothenemus -------------TVAPEISDNYV-LEKRSP-MGFFGTRGKKPSDW----------LGYNEEMY
Anoplophora  ----------KTKDKPLSVSETLETSVRIAP-SGSITNSENKPFAYMQLWK-----GTYPDMLY
Leptinotarsa Y-----QSMRPYENKL-------L-AEYLAR----LESFQERQ------WD-----GASTDGQY
Oreina       H-----QDVLPYDNKL-------L-AEYLAK----LDSDQARP------WD-----GF--NAQY
Aethina      -------------TLEEPHPEQQK-VEKRAP-SGFMGMRGKKPYSYGEPWE---GSYQFQDAVY
Hycleus      ------------EDDDDGGNDSLN-IEKRAP-SGFMGMRGKKPFPF---YD---EDNIYPDGIY
Tenebrio     -------------KA-DVVSDVGA-VNKRAP-SGFMGMRGKKPYPV---WE-----GTYPDEVY
Tribolium    -------------KAVDIVSDLGA-VDKRAP-SGFMGMRGKKPFSL---WE-----GTYPDGVF

           129
Pogonus      KRAPMGFMGMRGKKEMEMVG----DEYDKRAPNMGFLGMRGKKMSDED--ESLSDYYMEKRAP-
Ignelater    KRAPSGFLGMRGKKDIDLDS---SSEYDKRAP-SGFMGMRGKKDFEDD--VDTYNSYMDKRAP-
Photinus     KRAPSGFMGMRGKKEN-------LDDYDKRAP-SGFMGMRGKKEYDAD--Y--YGNDFVDKRA-
Aquatica     KRAPSGFMGMRGKKDNDLNN--IVEDYEKRAP-SGFMGMRGKKDFDN--------EYAEKRAP-
Nicrophorus  KRAPSGFQGMRGKKGLDYV-----EVLEKRAP-SGFLGMRGKKEYDDM---------FEKRAP-
Aleochara    PSGPSGFLGMRGKKDDLSED---EGEVYKRAP-NGFFGMRGKKEFEPF--VNE----YEKRAP-
Oryctes      KRAPTGFFGLRGKKDMDYVM-----DFEKRVP-SGFLGLRGKKFYDYMGFDDDETPFYEKRVP-
Coccinella   KRAPNGFFGMRGKKDDDADL---TYDMEKRVH-SGFFGMRGKKDEDPS--EPYSFYMSEKRAPS
Harmonia     KRAPNGFFGMRGKKGDDSDS---AYDMEKRMS-SGFFGMRGKKDDDLS--E-ASLYMSGKRAPS
Dendroctonus KRAPMGFVGMRGKKDSLEDDEVTWDEPEKRAQ-MGFFGMRGKKLWTDY--------QGEKRAP-
Hypothenemus KRAPMGFVGMRGKKESLDY---FNEPSLKRAR-MGFFGMRGKRYFVEP------QYQEEKRAP-
Anoplophora  KRARSGFVGMRGKKDSGFVE------F-------------------------------------
Leptinotarsa KRTPYRLMGVRGKRSASSSDEMNTN---------------------------------------
Oreina       KRTPNGFVGVRGKRAASSLAGINII---------------------------------------
Aethina      KRAPSGFMGMRGKKDNELFG--YD-EYPKRAP-SGFMGMRGKKEYDPF------EYYNEKRAP-
Hycleus      KRAPSGFMGMRGKKDIEFPN--FDEEYGKRAP-SGFMGMRGKKANDQ----DQDNDFYYKRAP-
Tenebrio     KRAPSGFMGMRGKKDMEFTN--YGDEYDKRAP-SGFMGMRGKKEYDSL--SNQYDGYFEKRAP-
Tribolium    KRAPSGFMGMRGKKDMEFAN--YD-EYIKRAP-SGFMGMRGKKDYDSS--SSQ----LDKRAP-
```

```
             193
Pogonus      MGFMGMRGKKEYSP-FM-EEEKRAPADGFFGMRGKKQPARSSFFGMRGKKYPYEFRGKFVGVRG
Ignelater    SGFMGMRGKKTFES-IA-EEMKRAQMSGFFGMRGKKQPSRSSFFGMRGKKYPYEFRGKFVGVRG
Photinus     RGFMGMRGKKSIDE-IN-EEVKRAVLSGFFGMRGKKQPARSSFFGMRGKKYPYEFRGKFVGVRG
Aquatica     NGFMGMRGKKSYED-MV-EEMKRAVMSGFFGMRGKKQPSRSSFFGMRGKKYPYEFRGKFVGVRG
Nicrophorus  SGFLGMRGKKDLETVLL-PEESKRAPNGFFGMRGKKPSRSAGFFGMRGKKYPYEFRGKFVGVRG
Aleochara    SGFLGMRGKKNDE--IDSVELKRAPSSGFVGMRGKKQPGRSGFFGMRGKKYPYEFRGKFVGVRG
Oryctes      SGFFGLRGKREFEDEIE-DDKRAAPENRFFGMRGKKMPARNGFFGMRGKKYPYEFRGKFVGVRG
Coccinella   MGFVGMRGRKSYDDTP--DEFEKRAPSGFFGMRGKKQ---WGQFALRGKKIPYQFRGKFVGVRG
Harmonia     MGFVGMRGKKR-----------------------------WGPFELRGKKIPYQFRGKFVGVRG
Dendroctonus MGFVGMRGKKDFENPIGYYEDIKRAPSGFFGMRGKKHP--SGFFGMRGKKYPYEFRGKFVGVRG
Hypothenemus MGFFGMRGKKDFEDSYY---DNKRAPNGFFGVRGKKQP--YGFFGMRGKKYPYEFRGKFVGVRG
Anoplophora  ---------------------------------GKKQ--SSEFFGMRGKKYPYEFRGKFVGVRG
Leptinotarsa ----------------------------------------------------------------
Oreina       ----------------------------------------------------------------
Aethina      MGFVGMRGKKSYEDEFV---EDKRAPQGFFGMRGKKLQRTSGFFGMRGKKYPYEFRGKFVGVRG
Hycleus      MGFVGMRGKKPT--------DDKRAPSGFFGMRGKKQPRQSGFFGMRGKKFPYELRGKFVGVRG
Tenebrio     MGFVGMRGKKEYD-ELM---EEKRAPSGFFGMRGKKMPRQSGFFGMRGKKYPYEFRGKFVGVRG
Tribolium    MGFMGMRGKKDYD-EIA---DEKRAPSGFFGMRGKKMPRQAGFFGMRGKKYPYQFRGKFVGVRG

             257
Pogonus      KKDSSENAIPEFEYNPSA------DSYATDLDMNDLMMLLSEGQDGP-TWEKRKGPSGFLGMRG
Ignelater    KKSSN-EVGDYGYLNGNLGNDHPLSHLSEGLDLNQLMLLLTENSDA----SLGNG-NEYFEDRV
Photinus     KKSSN-EIGDYGYLNGHLDNEEQLAKFAEDLDLNQLMLLLTDNNGNQ--WD----SSYLEQDRV
Aquatica     KKSSN-EVGDYGYLNGHVDNEMEVPQLTNDFDLNQLLQYLNENNGD---WSHDNANSYAGQEMV
Nicrophorus  KKMSN-EAVPPPEYYEEF----GK--LGQGLDINQLMLLLTENDGYNLAKET---GQDY-----
Aleochara    KKSSE-EAI-------------PMQRLTNDLDFNQLMLLLTENEQYNSPETYYVPENGNSV---
Oryctes      KRLNE-IGAPEYVNSE------EVPRYGGDMDLNQLMLLLNEKSSEQLHD--------------
Coccinella   KKTMSNNGN--------FE-----TEPNYNSDMNTLLMQLLEDEDLH-----------------
Harmonia     KKTSYDSGN--------VE-----TEPYLDSDMNRLLMQLLEDEEMRRTEADEV----------
Dendroctonus KKSSE-MAIPSYYYQDST------TDRLSDPDWNQLMLLLTGRNEFDRSH--------------
Hypothenemus KKSQI-TVPKYIYEEPNV------DKLISDLDYNQLMLLLTEQDESRQ----------------
Anoplophora  KKISHE-LPDKV--NV------DLNSWRKELQSNQLMLVLTEQNEPGGV---------------
Leptinotarsa ----------------------------------------------------------------
Oreina       ----------------------------------------------------------------
Aethina      KKDMT-DAD--LYRNDYL----ARLGNVPNLDMNQLLLLLTEKNDEYEANQIDVADDENRIAEK
Hycleus      KKAPN-QAV------IGE----PI--YEQELDLQQLLYLLTNQPDNGDFWNG--IDENSQ----
Tenebrio     KKASD-NPESDYYNNVDL----NT--LGQDLDLNQLMLLLTENDGASDIWNG--NNEVGQYSQK
Tribolium    KKASP-----DDYYNVDL----DT--VGQELDLNQLMLLLTENEGESDIWNG--NNEVGQYSQK
```

Figure S34. Alignment of Tachykinin precursors.

```
              1
Pogonus       MGNMYF---AVLFLT--IAALCVLSTSLYLEKRVGN-------AANFLGKLRMDPKKRPFCNAF
Ignelater     ---MITSTFFIVAVTVA-MLHYQETRALYLPKR-------ADQFSR---EDIVDPKKRPFCNAF
Photinus      ---MVSSILFVISVSIV-MLH-QDGLALFLPKR-------VDPLTR---TGITDPKKRPFCNAF
Aquatica      ---MVNSTMFLITITII-VLQ-HESSAFFLPKR-------VDPVSR---ADMADPKKRPFCNAF
Nicrophorus   ---MVSTTLFIVVVGF--AFLVIDTESIFLPKR-------ANGYEG--RDSIIDPKKRPFCNAF
Aleochara     MSVSPAFTVIMFVTTV--VVLLADVESVFLPKR-------LNGYDG--KDMVLDPKKRPFCNAF
Oryctes       ---MT--SVFFITILVASSLLLAYTHAVFLPKRDG-----VNYLA---KESLIDPKKRPFCNAF
Coccinella    ---MLPTKTIVFS-TIIASVFIGEAYFYVLQKKVSDVFKGGVYTIEERLEDNMDPKKRPFCNAF
Harmonia      ---MLPTKSIVFS-TIIVSVFISHAQLYVMQK------KGGVYTIEERVDDNMNTEKRPFCNAF
Dendroctonus  ---MTALTLISVVLYSTAMILIIELDALFIPKR-------MDPVLSARERIILDPKKRPFCNAF
Hypothenemus  ---MTALAVLFIALYSTAMTVIMGSEGLFLPKK-------LSPYINTREKIIFEPKKRPFCNAF
Anoplophora   --MTTSSAFFIICACMVAVLLVSEAEAMFLPKR-------GSAPYPTRERVFIEPDKRPFCNAF
Leptinotarsa  -MSTVSSMLIVICGCAVVC-LLSEVGSLYLPKK-------GLAPYHTRERVFLEPEKRPFCNAF
Aethina       ---MTTLTLWIICGCVI---YMFQVDAAYLPKR-------GPAPFNTKERI-LDPKKRPFCNAF
Hycleus       ---MTAFRYLIIF---VILAVVIEIDAVFLPRR-------LDFNLK--DK-IVSPKKRPFCNAF
Tenebrio      ---MTTATFFVICIAAV---LTVETRSLFLPKS-------LGQNLAARERV-VEPKKRPFCNAF
Tribolium     MKNMTTAKLFVICIFAA---LAIETHSRFLPKS-------ISKNLGATERV-LEPKKRPFCNAF

              65
Pogonus       TGCGRKRSNLPPLASNLF-------------DQEPPEMENLGTLLDLSAEPAVEDLSRQIMSEA
Ignelater     TGCGRKRSNLPALTNRG-----------------EPVEESIGSLLELSAEPAVEDLSRQIMSEA
Photinus      TGCGHKRSNLPALTNRG-----------------EPVEESIGSLLELSGEPAVEDLSRQIMSEA
Aquatica      TGCGHKRSNLPALTNRG-----------------EPVEETIGSLLELSAEPAVEDLSRQIMSEA
Nicrophorus   TGCGRKRSNLPALSM------------------QGDNDETIDSLLELSAEPAVEDLSRQIMSEA
Aleochara     TGCGRKRSNVPAYPAQP----------------QGYTDENIGSLLELSAEPAVEDLSRQIMSEA
Oryctes       TGCGRKRANIPPLMEIQQQLQQSQQMHRSQSPKHRVLDDSVAGLLELNAEPGIEDLSRQILSEA
Coccinella    TGCGRKRSNLPALTSDG-----------------EEIDDSISALLELNAEPAVENLSRQIMSEA
Harmonia      TGCGRKRSNLPALTSDG-----------------EEIDDSISALLELNAEPAVENLSRQIMSEA
Dendroctonus  TGCGRKRSNLPALFSDG-----------------QEMENSINSLLELSAEPSVEDLSRQIMSEA
Hypothenemus  TGCGRKRSNLPALSSDG-----------------QEVDNSIDSLLELSAEPAVEDLSRQIMSEA
Anoplophora   TGCGKKRSNLPALTDKG-----------------EEMDETINSLLELSAEPAIEDLSRQIMSEA
Leptinotarsa  TGCGKKRSNLPALADNG-----------------EEMDESINSLLELSAEPAVEDLSRQIMSEA
Aethina       TGCGRKRSNLPALSDQG-----------------EEIDESIGSLLELSAEPAVEDLSRQIMSEA
Hycleus       TGCGRKRSNLPALTDQG-----------------EVIEDSIGSLLELSAEPAIEDLSRQIMSEA
Tenebrio      TGCGRKRSNLPALTDQG-----------------EIIDESLGSLLELSAEPAVEDLSRQIMSEA
Tribolium     TGCGRKRSNLPALPEQS-----------------EVVDENLGSLLELSAEPAVEDLSRQIMSEA

             129
Pogonus       KLWEAIQEAHREIMRQRQETSDGEEIPAVNPLAGATSGVGVANGKCALPSCIAI
Ignelater     KLWEAIQEANVELARRKQESG-VEN--PDN---EAGTPTRSATASCALPPCYI-
Photinus      KLWEAIQEANVELARRKQESA--ES--LDN---DPSSPARSATASCALPPCYIK
Aquatica      KLWEAIQEANVELARRKQESA--ES--LDN---NPGSAARSASASCTLPPCYIK
Nicrophorus   KLWEAIQEANMEILRRKQESA--EG--YEE---EPATPARSATASCALPPCYI-
Aleochara     KLWEAIQEANMELLRRKQESL--EG--ADD---ELPTPARSATASCALPPCYI-
Oryctes       KLWEAIQEANIELRKEQEAMDN-------Q---ELTQDVRNMPNSCAVPPCYAA
Coccinella    KLWEAIQEANMELNRHRQKTKE-------N---ISQKNKIQVAGTCALPPCYTI
Harmonia      KLWEAIQEANMELNRHRQNSQG-------N---QQQKPGIQGAGTCAIPPCYI-
Dendroctonus  KLWEAIQEANLELKKHKNRPMLPSD--FSD---DVSMADRGAISTCALPPCYI-
Hypothenemus  KLWEAIQEANLELNRQRQGQPVNKQNKNDE---TSSDSDRGAIATCALPPCYIT
Anoplophora   KLWEAIQEANVELNRRKQQNS-VES--TED---DVAVPAKSATASCALPPCYI-
Leptinotarsa  KIWEAIQEANVELNRRRQNPRLLDG--EQN---DAPVPAKSATASCALPPCYI-
Aethina       KLWEAIQEANVELNRRKQGL--SDI--P-E---DDAVPARSATASCALPPCYI-
Hycleus       KLWETIQEANLELNRRKQQSSVENS----D---EDVVPAKSATATCALPPCYI-
Tenebrio      KLWEAIQEANMELHRRRQES--AES--AED---DVAVPPRSAAASCALPPCYI-
Tribolium     KLWEAIQEANMELHRRRQES--AES--SEE---DAAVPARSATASCALPPCYI-
```

Figure S35. Alignment of CCAP precursors.

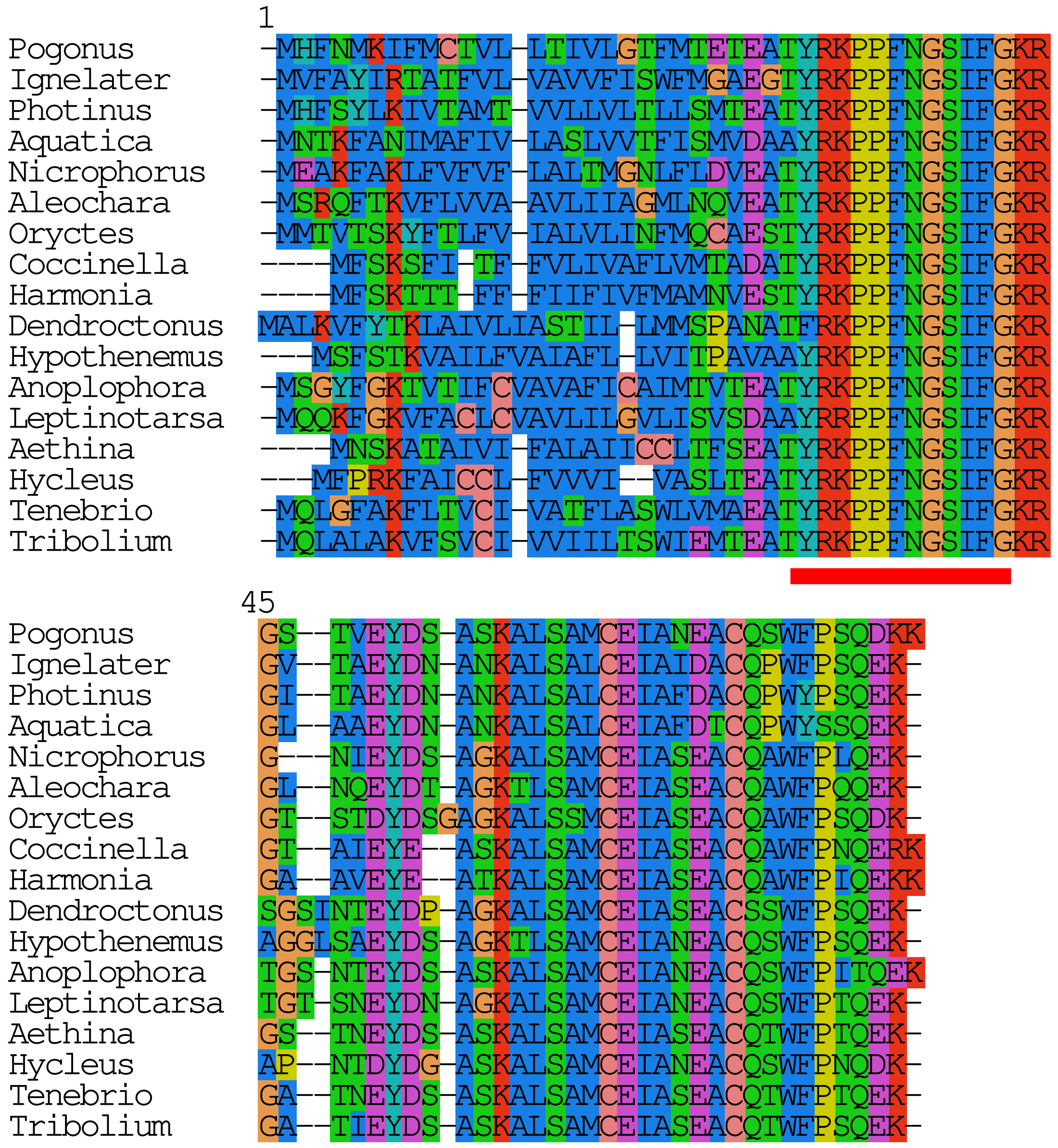

Figure S36. Alignment of SIFamide precursors.

```
             1
Pogonus      -MFGCWILIVATVVACVTMS----SGSLARDTWEKPGCHKVGHTRKISIPGCVEFRMTTNACRG
Ignelater    -MLAFWLLLAVT-STNVLLT----STVTAKDAWQRPGCHKVGHTRKISIPNCVEFHMTTNACRG
Photinus     -MLASWLLLAVI-FPNVLLS----STVIARDAWQKPGCHKIGHTRKISIPNCVEFHMTTNACRG
Aquatica     -MQKYWIFLVLI-FLNVLV-----ATVIAKDAWQKPGCHKIGHTRKISIPNCVEFHMTTNACRG
Nicrophorus  MLLVWWYLVAAFSVTDALLLQPMTAAAVAKETWQKPGCHKIGHTRKISIPNCIEIKITTNACRG
Aleochara    -MLTWWFLIAAISLSNAFLS----TSVVARDTWQKPGCHKVGHTRKISIPDCVEFHMTTNACRG
Oryctes      -MLACWLLIAVISLSNALLY----NTVTARDAWEKPGCHKVGHTRKISIPDCVEFPMTTNACRG
Coccinella   -MLVSWFLINLLPILNAFLI----NTAMETDGWRKPGCHKVGHTRKISIPDCVEFKMTTNACRG
Harmonia     -MLISWFLLNLLPLLNAFLI----NTAMETDGWKKPGCHKVGHTRKISIPDCVEFKMTTNACRG
Dendroctonus -MLAYYFLFLVLPFSYGTVLA-----PERDRDWHKPGCHKVGHTGKVNIRDCVSFDITTNACRG
Hypothenemus -MLVYYFLFWLILFSNGGL-------SDTDLDWQKPGCHLVGHKQTINLRGCISFDITLNACRG
Anoplophora  -MLAFWFLVIVVSFSNAFLV----TSVNAKDAWYAPGCHKVGHTRKISIPNCVEFYMTTNACRG
Leptinotarsa -MPPFWFLFVAVSSSIDFIP----GSANAGEAWRAPGCHKVGHTRKISIPNCVEFHMTTNACRG
Aethina      -MLAFWLLMTALSFSNAFLV----SSVNARDAWMKPGCHKVGHTRKISIPDCVEFSMTTNACRG
Hycleus      -MLACWLLFTILSLSHAFLV----TTVNAKDSWQRPGCHKVGHTRKISIPDCVEFHMTTNACRG
Tenebrio     -MLACWLLFTVLSLSDAFMV----TTVNARDAWQKPGCHKVGHTRKISIPECVEFHMTTNACRG
Tribolium    -MLACWLLFTLLSLSDAFMV----KAVTARDAWQKPGCHKVGHTRKISIPECVEFHMTTNACRG

            65
Pogonus      FCESWAIPSGPKA-NPEQPVTSVGQCCNIMDTEDVTARVLCVDGVKMLTFKSATNCACYHCKKD
Ignelater    FCESWAVPSGPKA-SPTQPVTSVGQCCNIMDTEPVEARVLCVDGVRTLTFKSAVTCSCYHCKKD
Photinus     FCESWSVPSGPKT-TPTQPVTSVGQCCNIMDTEPVEARVLCVDGVRTLTFKSAVSCSCYHCKKD
Aquatica     FCESWSVPSGPKA-SPTQPVTSVGQCCNIMDTEPVEARVLCVDGVRTLTFKSAVTCSCYHCKKD
Nicrophorus  YCESWAVPSSTNI-SWTQPVTSVGECCNIMDSELQEVQVFCVEGVRTLTFKSAISCSCYHCRKY
Aleochara    YCESWAVPSGPKA-SPTQPVTSVGQCCNIMDTEPVEARVLCVDGVRTLTFKSAVSCSCYHCKKD
Oryctes      YCDSWAVPSGPKT-SPTQPVTSIGQCCNIMETEPLEARVLCIDGFRTLEFRSATRCSCYYCKKD
Coccinella   YCESWAVPTHHKV-NPAQPITSIGQCCNIMETEPVEARVLCVDGIRTLTFKSATSCSCYHCKKD
Harmonia     YCESWAIPSHQKN-NPAQPITSIGQCCNIMETENVEARVLCADGIRTLTFKSAISCACYHCKKD
Dendroctonus FCESWAIPSDPQNAQISQPVTSIGTCCNIMETEPIEVRVLCKEGIRTLVFKSAVSCACYHCKKD
Hypothenemus YCESYALPSDPRNTQITQPVTSVGTCCNIMESESYEARVLCVDEVRHLVFKSAVTCACYHCKKD
Anoplophora  YCESWAVPSSPKT-TPSQPVTSIGQCCNIMETEPAEVKVLCVDGIRTLTFKSAVTCSCYHCKKD
Leptinotarsa FCESYAVPSLPKI-TPTQPVTSIGQCCNIMETEPVEARVLCVDGMRTLTFKSAVTCSCYHCKKD
Aethina      FCESWAIPSGMKT-SPTQPVTSVGQCCNIMDTEPIEAKVLCIDGVRTLTFKSAVSCSCYHCKKD
Hycleus      FCESWAVPSGPKA-TPNQPVTSVGQCCNIMETEPFEARVLCVDGVRTLTFKSAVSCACYHCKKD
Tenebrio     FCESWAVPSGPKA-TPTQPVTSVGQCCNIMETEPVEARVLCVDGVRTLTFKSAVSCSCYHCKKD
Tribolium    FCESWAVPSGPKA-TPTQPVTSVGQCCNIMETEPVEARVLCVDGVRTLTFKSAVSCSCYHCKKD
```

Figure S37. Alignment of GPA2 precursors.

```
              1
Pogonus       ---MVGYFSWITVAFIC-----I-----------QATIALDSAETLECHRRLYTYRVTQSDDQG
Ignelater     ----MFSLFWIFMAA-TSICG-TQQVIEAGTGP------IDAASTLDCHRRLYTYRVTKADENG
Photinus      ----MFLGIGILLTV-ASISG-TQKIVNADLER------VDPINTLDCHRRLYTYRVTQSDENG
Aquatica      VAAAMLILFGIFLTA-FSICR-TQHIIEAGIET------IDPATTLDCHRRLYTYRVTQADENG
Nicrophorus   MSWMS---AAILALLLMSIVDCQKSLVEADASAGG--TSMESSRTLECHRRLYSYKVTQTDENG
Aleochara     MKVEVILSLLLLAGNVEK-IRAQISLIEAV----PEPINKEMKDPLDCHRSSYTYRVTQSDKNG
Oryctes       ----MF-RFWMSCLALAAVGRCQMNVIDAGL----EPIY---ASTLECHRKIYTYYVSQSDVNG
Coccinella    MQYLISWNICLFMI-TT--SVV--SQIDVDSLIPPEE-------DLECRERVYTFQVTQTDENG
Harmonia      MKYLISWNICFFII-ST--SVDCQSIIDVESLIPPEE-----PESLECRERIYTFQVTQTDING
Dendroctonus  ---MFFQ-LVFFLLYIS-TMIDTQSIIEAASFEE-----EVENSTLKCHKRLYNYSVSQVDKNG
Hypothenemus  ---MIFQ-LLFFFMYIG-SFVRTQSIIDGIALDN-----VNQKTTLKCHKFLHKHDVTQLDKNG
Anoplophora   ---MINILVWMTLTLCV--VVKGQSIIEANTELQEEEPLKDAAITLECHRRLYTYRVTQTDENG
Leptinotarsa  --MMITILLCLAVTL----NSARGVIINADSVLEGEQPLANAGKTLSCHRKLYTYLVTQQDENG
Aethina       ---MSIPPLWLTLGVCS--LVIGQSIIKAPPEF------VEVSETLDCHKRMYTYHVTQTDENG
Hycleus       ---MKSYQFLLIILSNS--LINGQSIIDVGLEP------LDASNTIECHRRLYTYRVTQTDENG
Tenebrio      ---MLSVQVWILVCLSA--LVSSQSIIEAGLEP------LDASGTIECHRRMYTYRVTQTDDNG
Tribolium     ---MLGVQVWLFVGLSA--LVRCQSIIEAGLEP------LDASGTIECHRRMYTYRVTQTDDNG

             65
Pogonus       KQCWDTLSVMSCWGRCDSNEISDWRFPYKRSYHPVCVHAGRSKAVAKLRHCEAGVAPGTDRYVY
Ignelater     KQCWDTLSVLSCWGRCDSNEISDWRFPYKKSSHPVCVHYGRNKAVATLRHCEEGALPSAAKYEY
Photinus      KECWDTLSVLSCWGRCDSNEISDWRFPYKKSLHPVCVHHGRNKALAILRHCEEGALPSAARYEY
Aquatica      KQCWDTLSVLSCWGRCDSNEISDWRFPYKKSLHPVCVHYGRNKVVAILRHCEEGASPSASRYEY
Nicrophorus   KMCWDIIQVWACWGRCDSNEISDWRFPYKKSSHPVCMHYGRNKAVANLRNCEDGALPSAAKYEY
Aleochara     RQCWDTLTVMACWGRCDSNEISDWRFPYKKSSHPVCVHYGRRQSVVELRHCEEGAGEEARRYEY
Oryctes       RQCWDTLSVWACWGRCDSNEIVDWRFPYKKSSHPVCVHYGRTRAVATLRHCEEGAHPSAARYEY
Coccinella    KQCWDALSTVACWGRCDSNEISDWRFPFKKSNHPVCIHYGRNRNVVTLRHCEEGALPEAARYEY
Harmonia      KQCWDALSTIACWGRCDSNEISDWRFPYKKSNHPVCIHYGRNRNVVTLRHCEEGALPEAAHYEY
Dendroctonus  KKCWDILSVMACWGRCDSNEIVDFRFPYKKSVHPVCVHYGRNKAFVILRNCEEGALPSAARYEY
Hypothenemus  KACWDTLSVMACWGRCDSKEMADYRFPYKKSFHPVCVHYGRRKVFVILRNCQEGALPSAARYEY
Anoplophora   RQCWDTISVMACWGRCDSKEISDWRFPYKKSTHPVCVHYGRNRAFVTLRHCEEGVLKGTDHYEY
Leptinotarsa  KQCWDKVSVWACWGRCDSNEISDWRFPFKRSNHPVCVHYGRNRVFVTLRHCEDGIVEGTDHYEY
Aethina       RSCRDKVSVMACWGRCDSNEIADWRFPYKKSNHPVCVHYGRKRSEVILRNCDEGVMPLTRIYQY
Hycleus       KQCWDTLSVWACWGRCDSNEISDWRFPYKKSNHPVCVHYGRNRSIVTLRHCEEGALPTASRYEY
Tenebrio      KQCWDTLSVMACWGRCDSNEISDWRFPYKKSNHPVCVHYGRNRSVVTLRHCEEGADPTTARYEY
Tribolium     KQCWDTLSVMACWGRCDSNEISDWRFPYKKSNHPVCVHYGRNRSVVTLRHCEEGANPSAARYEY

            129
Pogonus       LEAEACRCQPCTSSDTSCEGVRYRGHRNQAMTLLSLDSEDGAFGNDEENIIE
Ignelater     LEAAGCRCQACSSSDTSCEGLRYRPHRSHPDLLGFGPQ--------------
Photinus      LEAAGCRCQACSSSDTSCEGLRYRPHRSQPDLIGFGNM--------------
Aquatica      LEAAGCRCQACSSSDTSCEGLRYRPHRSQPDIYGFGSK--------------
Nicrophorus   MEAGGCRCQFCFSADTSCEGLRYTTHRSH-----------------------
Aleochara     LEAAGCRCQLCSSTDTSCEGLRYRPHRSHPDQLGFRF---------------
Oryctes       LEAAGCQCQLCSSSDTSCEGLTYRSHRSHPDLIGFGLH-K------------
Coccinella    LEAAGCKCQICSSSDTSCEGLRYRPQRSHPQSLGFQLI--------------
Harmonia      LEAAGCKCQICSSSDTSCEGLRYRPQRSHPQSLGFQLS--------------
Dendroctonus  YEAADCKCQQCSSLDTSCEGLRYRSQRSEPDR-GFKRIIRGREVDIY-----
Hypothenemus  YEAADCKCQQCTSLDTSCEGLRYRAHRSGF--GGFRRLVRGGEEFF------
Anoplophora   LEADDCKCQLCSSSDTSCEGLRYRPHRSHPDSLGFRYNFKK-----------
Leptinotarsa  LEADDCKCQVCSSSDTSCEGVRYRPHRSDPDRLGFL----------------
Aethina       LEADECKCQQCSSSDTSCEGITYRPHRSHPDSLGFKIN--------------
Hycleus       LEAAGCKCQLCSSSDTSCEGLRYRPQRSHPGSLGFRIN--------------
Tenebrio      LEAAGCKCQQCSSSDTSCEGLRYRPQRSHPASLGFRIN--------------
Tribolium     LEAAGCKCQQCSSSDTSCEGLRYRPQRSHPASLGFRIN--------------
```

Figure S38. Alignment of GPB5 precursors.

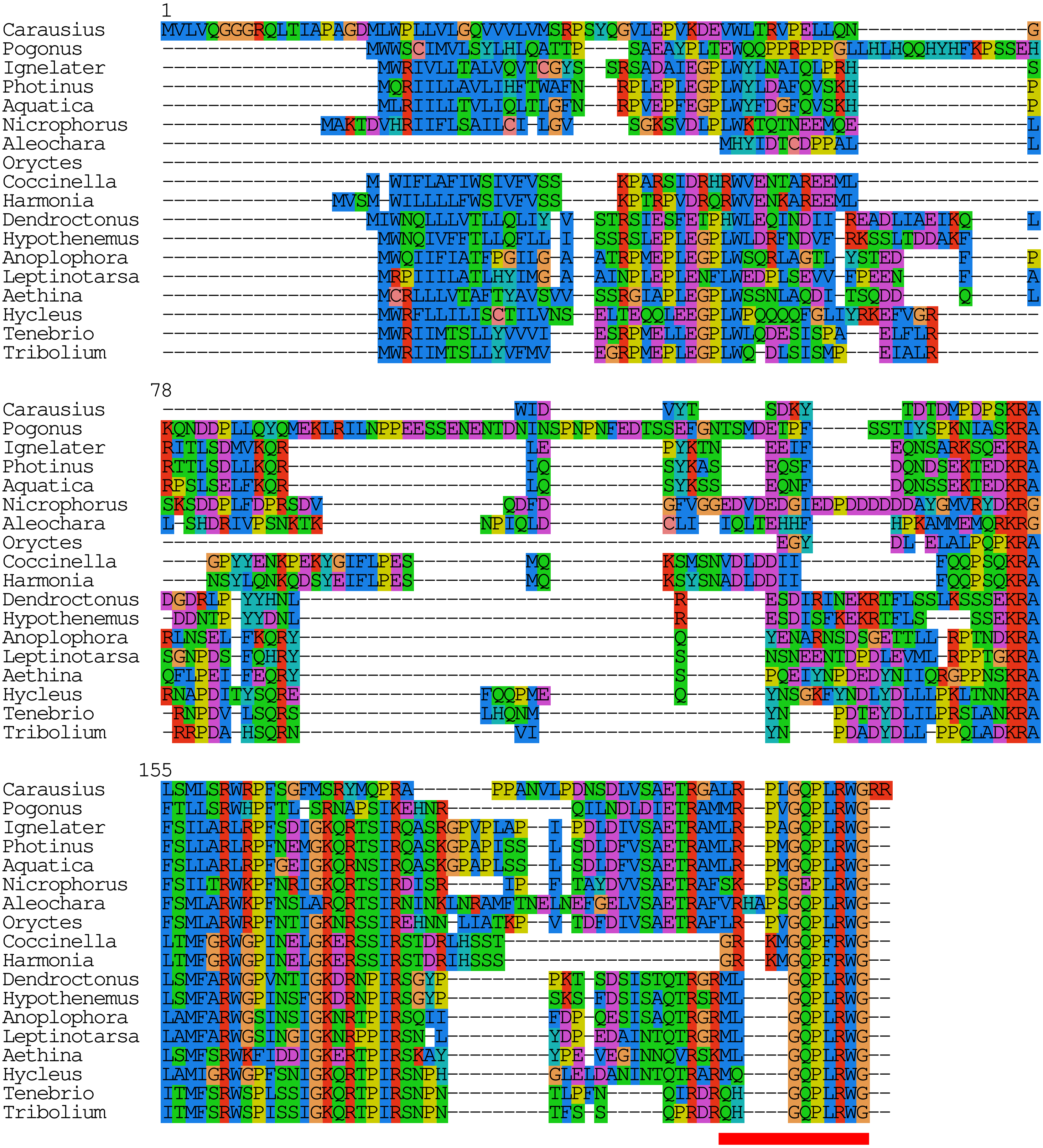

Figure S39. Alignment of Hansolin precursors.

```
                                1
Pogonus                         ---MQKNY---LLVTVVLCLASNSWSAHLI---TPKNCLPCILAANSI
Ignelater                       ----MIIIFALVLVTTVQSYPAGQILY----------------SKDL-
Photinus                        ---MKIITLLIFIVTVTHAYPPGQFLY----------------SDNL-
Aquatica                        ---MKIIIILV-FVTFAYSYPPGQILY----------------SDNL-
Nicrophorus-splice-variant-1    MISLRRQFLVQLLALLVTGQP-----IHLTE--DEMNKDIGIITT---
Nicrophorus-splice-variant-2    MISLRRQFLVQLLALLVTGQP-----IHLTE--DEMNKDIGIITT---
Aleochara                       ------------------------------------------------
Oryctes                         ------------------------------------------------
Coccinella                      ----MIVLLGIILVSRVHCRSFIEGLSEAGVIPQINPTYI-ITSDSM-
Harmonia                        ----MIVLLGIILVLRVDCRSFIQGY------PRVHPGYI-IRSDSI-
Dendroctonus                    ---MNLVVLIIVF---SIGNIRETFARSVVVSSAIGVQDSEAADARIS
Hypothenemus                    ---MILSLFIAIF---SVGHVRQSMAGTLTLVPKHGVADL------LL
Anoplophora                     ---MVLVLVAII-FLALCGGLVKSFPARLAMEAGPGE---RVFQKGLS
Leptinotarsa                    ----------------------------KVEATTLGE---QDFEK---
Aethina                         ---MIVILVGLICVAGTYGAIAT----DFD---AYFNTMSQPRKGDLG
Hycleus                         ----MRITLV-ILILVICGSLFNNITTALP---TIFNTLP--------
Tenebrio                        ----MRIAFGVIFVTGIFGGFFGDNASASP---VHHHHVI---SKDLD
Tribolium                       ----MRIAFGVIFVTGIFGGFFAENVFALP---VARHHVI---TKDLD
Tribolium-splice-variant        ----MRIAFGVIFVTGIFGGFFAENVFALP---VARHHVI---TKDLD

                                49
Pogonus                         KRSEENAMDSLLEVANEEEDDSL----------ERRYILDMDKIHEQA
Ignelater                       SNSVDEVYGSYFTLSDNRKDNNEVNNHPYSQPYLNKMIIPP--PSPQQ
Photinus                        SNSVDEVYDSYLSVSDDDKDD---VNPPPNS-LLNQLIENE----N--
Aquatica                        SNSVDEIYDTYLSVADDDKED---INPPPPEPYLNKLIEET--INL--
Nicrophorus-splice-variant-1    TSSGNQIRNSILQYSDEKIADELQ----YVSQSINRLYSEESYPWNG-
Nicrophorus-splice-variant-2    TSSGNQIRNSILQYSDEKIADELQ----YVSQSINRLYSEESYPWNG-
Aleochara                       ---GEQ-------------------------QRMNSLYRNL-LIFNR-
Oryctes                         ----------------DDEDYNNNILHHKFYAKLQRYYLDK--LQNQQ
Coccinella                      -DKINEEYDTYIDLKDNDAESNNF----DFISEEERFFSNG--RQF--
Harmonia                        -GKIKEEYDAYIDLKDNEDDSNEY----DILSEEERFISNG--HQF--
Dendroctonus                    QNDQKQISGNYISYLDNQA----------------ALLVDQ--IQQYK
Hypothenemus                    PLEPTSFGKNHLFIFNQQEPSRIQNDIAYFDKREGQMNVKK--LNNYR
Anoplophora                     SDSVNEVYDSYYAISKKNEENEET----NVIARVQRVDLNK--IKYSH
Leptinotarsa                    --KPAIILNDYYLFDN---NQEEL----DVFSQIHQINSHN--FRLSH
Aethina                         KTTPDRIQETYSLANRE-EIREE--------KTTSSLFLNK--LKEVY
Hycleus                         ----KTNNELYLSVSDKYSDNSNRLD--KMSARRRQVTLHQ--PKFLQ
Tenebrio                        DTTADKMNKPYISISHDRADNSDK---ADVITRVQKVFVDS--PKSNV
Tribolium                       L---DEMYKSYTSISNDRDDYNDK----V----TSRVYLDS--PQISV
Tribolium-splice-variant        L---DEMYKSYTSISNDRDDYNDK----V----TSRVYLDS--PQISV

                                97
Pogonus                         RAAAFDALIKLHDQSDNVAIIHRHKKQRHDKRFLNCKFATLPVRHARA
Ignelater                       SEDLAKSEP--------------ESRRVERTAY-------LH------
Photinus                        --ANLISPA--------------ETQRVEQTSI-------VH------
Aquatica                        --ESLINPE--------------DAQRVERTAF-------VH------
Nicrophorus-splice-variant-1    ------------------------VSPF-KPEV-------AP------
Nicrophorus-splice-variant-2    ------------------------VSPF-KPEV-------AP------
Aleochara                       ------------------------VADMLENDY-------LQ------
Oryctes                         A---ITNLE--------------D-SEIQRTAY-------LL------
Coccinella                      -------GR--------------ERDREWIQTY-------IL------
Harmonia                        -------GT--------------DRDRELIQTY-------IL------
Dendroctonus                    N----KELS--------------KEVQSRIEEF-------IM------
Hypothenemus                    -TIAMEPLP----------A---LVRDRIEDSF-------V-------
Anoplophora                     ------------------------IYDINGDTY-------IL------
Leptinotarsa                    ------------------------FYNLRGRTV-------LV------
Aethina                         K---FDSLG--------------RPQKIEERAY-------LL------
Hycleus                         -----HALK--------------PKKLDEETAY-------LV------
Tenebrio                        ----SKNGK--------------Q-QKTKQTAY-------LL------
Tribolium                       ----SKNGK--------------Q-QKTKQTAY-------LL------
Tribolium-splice-variant        ----SKND----------------------------------------
```

```
                                   145
Pogonus                            AEAREGQKGGKRRAAVSDLSLCHFKICNMGRKRN--QRFIHSFNEYDN
Ignelater                          ---IQTK-QKDKRYA-SYLTLCHFKICNMGRKRT--TRYFHVISSLDN
Photinus                           ---VQTR-EKNKRYS-SYLTLCHFKICNMGRKRT--TRYFQVMKTLDN
Aquatica                           ---VQTQ-EKNKRYS-SYLTLCHFKICNMGRKRT--TRYFQMMRSLDN
Nicrophorus-splice-variant-1       ---GKIRRQRKKRFLVTDLTLCHFKLCNMGKRKT--TRTFQKISRASS
Nicrophorus-splice-variant-2       ---GKIRRQRKKRYL----TLCHFKLCNMGKRKT--TRTFQKISRASS
Aleochara                          ---KHRNKRNDPKSYVSDAAPCYFKLCNLGKRRA--------------
Oryctes                            ---IKTMKQRDKRYTSSYLTLCHFKICNMGRKRT--TRYFHMIKSLHD
Coccinella                         ---VKTMRQRNKRYT-SYLTLCHFKICNIGKKRR--TRYIHGMQSEKG
Harmonia                           ---VKTLRQRNKRYS-SYLTLCHFKICNMGKKRR--TRFVHGMESSRE
Dendroctonus                       ---LKQQSNNQRQKRRPRLTPCLFKICNMGRKRQ--HHVLCTYKS---
Hypothenemus                       ---FLKMKTTRERSKRSHLMPCYYKICNMGRKRL--------------
Anoplophora                        ---VKAFRHRNKRFT-SYHTLCPFKICNMGRKRTRTRRYIEPIRPVGD
Leptinotarsa                       ---LKTLKHRNKRYT-NYLTLCPFKICNMGRKRMRAKRSNNKIKRSSD
Aethina                            ---VKTLKHRNKRYS-SYLTLCHFKICNMGRKRN--TRYKSFNDMEND
Hycleus                            ---VNRM-QRDKRYI-SYLQLCHFKICNMGRKRS--ARYFHMIRKLDD
Tenebrio                           ---VKTMRQRNKRYI-SYLTLCHFKICNMGRKRT--TRYFHMIRRLDN
Tribolium                          ---VNTMSQRNKRYI-SYLTLCHFKICNMGRKRT--SRYFHMIRQLDD
Tribolium-splice-variant           ------------------LTLCHFKICNMGRKRT--SRYFHMIRQLDD

                                   193
Pogonus                            -----
Ignelater                          GT---
Photinus                           KT---
Aquatica                           KT---
Nicrophorus-splice-variant-1       PNWRI
Nicrophorus-splice-variant-2       PNWRI
Aleochara                          -----
Oryctes                            DHT--
Coccinella                         V----
Harmonia                           NRI--
Dendroctonus                       D----
Hypothenemus                       -----
Anoplophora                        NDET-
Leptinotarsa                       GRYSS
Aethina                            LEYYK
Hycleus                            NES--
Tenebrio                           ES---
Tribolium                          NEA--
Tribolium-splice-variant           NEA--
```

Figure S40. Alignment of CNMamide precursors.

```
                1
Pogonus         -MRAVVVATVLCLLCSGVRVNAWGGLFNRFSPEMLANMGYGAHGGFAQPFIQRAGGEENILESFDEG--EGDDEPCY
Ignelater       -MKGLIVLIAVCVLG-K-QADAWGGLFNRFSPEMLANMGYGGHSGF-----IQRAGDEGILEEFEGE---GDDEPCY
Photinus        -MKSVIVIIAISVLT-Q-RAHAWGGLFNRFSPEMLANMGYGGHSGF-----IQRAGDEGILEEFETD---SDDDPCY
Aquatica        -MKAFVIIVAVCLLA-H-RAYAWGGLFNRFSPEMLANMGYGGHSGF-----IQRAGDEGILEEFEGE---AEDEPCY
Nicrophorus     -MKGLIALLAICLLG-R-QTEAWGGLFNRFSPEMLANMGYGGHGGGGGG--GFRTGDEGILEEFEVD---SDDDPCY
Aleochara       -MKVIIALLALCVLG-R-QTEAWGGLFNRFSPEMLANMGYGAHGG---G--LPRGADEGILEEFEAE---EDADSCY
Oryctes         -MKGLIAMVAICLLG-K-YADAWGGLFNRFSPEMLANMGYGGHGNSIA---RIGGGDEGILEEFEIN--RGDEDPCY
Coccinella      -MRSLISLLAICLVG-QQTAHAWGGLFNRFSPEMLANMGYGGHG-GFIQ--RAGEGDEGILEEYSSD--V-DEEPCY
Harmonia        -MKSLITLLALCLVG-QQTAYAWGGLFNRFSPEMLANMGYGGHG-GYMQ--RSGEGDEGILEEYTND--V-EDEPCY
Dendroctonus    -MKSLAALLAVFLVC--HQVHAWGGLFNRFSPEMLANMGYGGHGGGMM-----RDGDDNFLEEYVNEAEEEGREPCY
Hypothenemus    -MKSFAALMAVLLVV--NRVSAWGGLFNRFSPEMLANMGYGGHGAGIL-----RDEENFLDEQYAN---VEDDGPCY
Anoplophora     -MRAAIAILAFCLLG-Q-KAHGWGGLFNRFSPEMLANMGYGGHGGGFIS--RNGEGDEGIMEEFVSE--NSEDEPCY
Leptinotarsa    MMKTIVAILAFCYVG-R-EVNAWGGLFNRFSPEMLANMGYGGHGGGGFS--R-AEGDDGILEEYVSE--NGDDEPCY
Aethina         -MPTLVTILALCLVG-R-QAHAWGGLFNRFSPEMLQNLGYGGHGG-F----ARGDGDEGILEEFAKE--G-DMDPCY
HycleusITG      -MRTLILLFVVCTMS-F-ETHAWGGLFNRFSPEMLANMGYGGHS-GFIQ--RTGDGDEGILEEYASD--N-DDEPCY
Tenebrio        -MRALIILFMACLLG-H-KAHGWGGLFNRFSPEMLANMGYGGHG-GFIQ--RTGEGDEGILEEYASE--G-DEEPCY
Tribolium       -MRALIILFTACLLG-H-KAHAWGGLFNRFSPEMLANMGYGGHG-GFIQ--RTGDEDKGILEEYTNE--G-EEEPCY

               78
Pogonus         GKRCTANEHCCPGSVCVDVDGVIGSCLFAYGRRAGELCRRDSDCESGLLCAEADQGA-ARVCRPPVHQDKQYNEPCH
Ignelater       GKRCTANEHCCPGSVCVDVDGVVGTCLFAYGRRAGELCRRDSDCESGLLCAEPESGGSARVCRPPVRQDKQYSEPCN
Photinus        GRRCTANEHCCPGLVCVDVDG-AGTCLFAYGRRAGELCRRDSDCESGLLCTESESGGATRICRPPVRQDKQYSETCN
Aquatica        GRRCTANEHCCPGSVCVDVDGVVGTCLFAYGRRAGELCRRDSDCESGLLCAEPETGGSTRVCRPPVRQDKQYSETCN
Nicrophorus     RKSCTANEHCCPGQVCVDVDGVVGTCLFAYGRRVGELCRRDSDCESGLVCTESEQTSSSRICRPPVQQDKQYSEPCN
Aleochara       GRKCSANEFCCPGQICIDVDGVYGQCLFTYGRRVGELCRRDSDCESGLVCAEAEQGISTRVCRPPV-----------
Oryctes         ERPCTANEHCCPAQVCIDVDGAVGTCVYAYGRRIGELCRRDSDCESGLVCSEGDQGISARICRPPVQQDKQYSELCN
Coccinella      GKRCTANEHCCPGSVCVDVDGVVGSCLFAYGRRVGELCRRDTDCESGLVCAEAEPGTMTRVCRPPVHQEKQYSELCN
Harmonia        GKRCTANEHCCPGSVCVDVDG-VGSCLFAYGRRVGELCRRDTDCESGLVCAEAEPGTMTRVCRPPVHQEKQYSELCN
Dendroctonus    GKKCTSNEQCCPGLVCVDTETLVGSCLFAYGRRVGELCMKDSDCESGLVCAEADMGPNARVCRPPVRQDKQYSEPCH
Hypothenemus    GKKCTANEHCCPNSVCVDVDGVVGSCLMTYGRRVGELCRSDSDCESGLVCAEADSGPSARICRH---QDKQYSEPCH
Anoplophora     GRRCTANEHCCPGSVCVDVDGVMGSCLFAYGRRVGELCRRDSDCESGLVCAEADGGVSTRVCRPPVHQDKQYSEPCN
Leptinotarsa    GKRCTANEHCCPGSVCVDVDGVIGSCLFAYGRRLGELCRRDSDCESGLVCAEAEVGASTRVCRPPVHQDKQYSETCN
Aethina         GKPCTANEHCCPGSVCVDVDGVVGSCVFAYGRRVGELCRRDSDCESGLVCAEAEPDVPTRVCRPPVHQDKQYSETCN
HycleusITG      GKRCTANEHCCPGSVCVDVDGVVGSCLFAYGRRVGELCRRDSDCESGLVCAEAEQGVS-RVCRPPVHQDKQYSEPCN
Tenebrio        GKRCTANEHCCPGSVCVDVDGVVGSCLFAYGRRVGELCRRDSDCESGLVCAEAEPGVSTRVCRPPVHQDKQYSEPCN
Tribolium       GKPCTANEHCCPGSVCVDVDGVVGSCLFAYGRRVGELCRRDSDCESGLVCAEAEPGVSTRVCRPPVHQDKQYSEPCN

              155
Pogonus         MSGECDISRGLCCQLQRRHRQAPRKVCSYFKDPLVCIGPVASDQIKNSVQHTAGEKRLTG---YKRPMH--
Ignelater       MSSECDISRGLCCQLQRRHRQAPRKVCSYFKDPLVCIGPVASDQIKNSIQHTAGEKRLTGFTGFKRPMH--
Photinus        MSSECDISRGLCCQLQRRHRQAPRKVCSYFKDPLVCIGPVASDQIKNSIQHTAGEKRLTGFTAYKRPMH--
Aquatica        MSSECDISRGLCCQLQRRHRQAPRKVCSYFKDPLVCIGPVASDQIKNSIQHTAGEKRLTGFTAYKRPMH--
Nicrophorus     MSSECDISRGLCCQLQRRHRQAPRKVCSYFKDPLVCIGPVAADQIKNAIQHTAGEKRLTGLAAFKRPMH--
Aleochara       -SSECDISRGLCCQLQRRHRQAPRKVCSYFKDPLVCIGPVASDQIKNSIQHTAGEKRLTGLTNYKRPMH--
Oryctes         MSSECDISRGLCCQLQRRHRQQPRKVCSYFKDPLVCIGPVAADQVKNAVQHTAGEKRLTRFTAFKRQMH--
Coccinella      MSSECDISRGLCCQLQRRHRQAPRKVCSYFKDPLVCIGPVATDQIKNSVQHTAGEKRLTGFSSFKRPLPMH
Harmonia        MSSECDISRGLCCQLQRRHRQAPRKVCSYFKDPLVCIGPVATDQIKNSIQHTAGEKRLTGFTSFKRPLPMH
Dendroctonus    MSSECDISRGLCCQLQRRHRQAPRKVCSYFKDPLVCIGPVATDQIKNAIEHTAGEKRITGLA-FKRPIMH-
Hypothenemus    MSSECDISRGLCCQLQRRHRQVPRKVCSYFKDPLVCIGPVATDQIKNIVEHTAGEKRITGMA-YKRSMMH-
Anoplophora     MSSECDISRGLCCQLQRRHRQAPRKVCSYFKDPLVCIGPVASDQIKNAIQHTAGEKRLTGLTSYKRPMH--
Leptinotarsa    MSSECDISRGLCCQLQRRHRQVPRKVCLYFKDPLVCIGPVAPDQIKSNIQHTAGEKRLTGLSTFKRPMH--
Aethina         MSSECDISRGLCCQLQRRHRQAPRKVCSYFKDPLVCIGPVAIDQIKNAVQHTAGEKRITGMA-YKRPMH--
HycleusITG      MSSECDISRGLCCQLQRRHRQAPRKVCSYFKDPLVCIGPVASDQIKSAVQHTAGEKRLTGLATFKRPMH--
Tenebrio        MSSECDISRGLCCQLQRRHRQAPRKVCSYFKDPLVCIGPVASDQIKNTIQHTAGEKRLTGLASFKRPMH--
Tribolium       MSSECDISRGLCCQLQRRHRQAPRKVCSYFKDPLVCIGPVASDQIKSNIQHTAGEKRLTGLAGFKRPMH--
```

Figure S41. Alignment of ITG-like precursors.

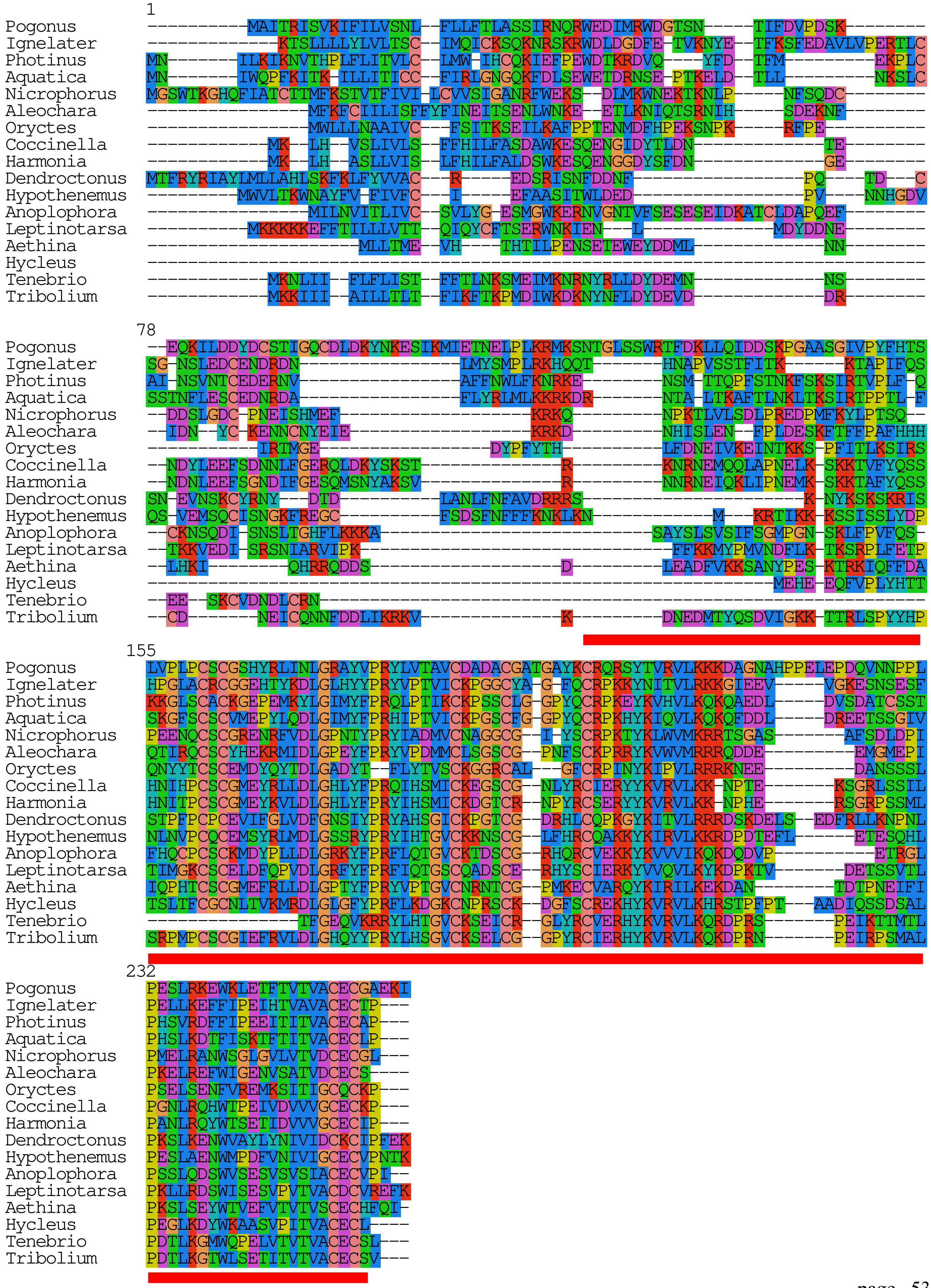

Figure S42. Alignment of PTTH precursors.

```
              1
Pogonus       MPQTQSSSKSAINSTSCVSTRTTRRLRTGNVRCAAVWLAAACALALGGNGGSGTGGSRLAIPSG
Ignelater     ----------------------MHHRSSTCVSTRGVWVCMALALVLQLAQ--------------
Photinus      ----------------------MHRRSSTSIYTKGVWVCMALTLVIQLAHC-------------
Aquatica      ----------------------MHRRSSTSLNTKGVWVCMALTLVFQLTQ--------------
Nicrophorus   -----------------------MHHRSASISTQRVWAFMALALVLQMVS--------------
Aleochara     -----------------------MHQRSTSIGTQGVWAYMAVALMLQMTAC-------------
Oryctes       -----------------------MNHRSTSVSTQFVWVCMALTLVVQIAL--------------
Coccinella    -----------------------MNYLSSKVSTTLLWVCMALVLFLSGSF--------------
Harmonia      -----------------------MNYLSSKVSTTLLWVCMAFIFFFGSSL--------------
Dendroctonus  -----------------MSQRSSQLEQRGRGTRRAAVWCLAIALVVQSARA-------------
Hypothenemus  -----------------MSQRSSQLEQRGRGTRRAAIWCLAIALVVQSARA-------------
Anoplophora   ---------------------MSHRSLKSVTNTQAVWVCMALALIFHITS--------------
Leptinotarsa  ---------------------MSNRSSSSVRGTQVAWWCMAIALILHVSS--------------
Aethina       ---------------------MSHPLSQSVRSTQIVGVCMALALVVQVVV--------------
HycleusITP    --------------MFQIPEKHMNCQSPKSISRQAIWSCMALAVLLIQVTT-------------
Tenebrio      ----------------------MSYRSSIIVNTQAVWVCMTLAVIIQAVTS-------------
Tribolium     ----------------------MNYRSSKSISTQAVWVCMVLAVVFQEITS-------------
Tribolium     ----------------------MNYRSSKSISTQAVWVCMVLAVVFQEITS-------------

             65
Pogonus       ATASPAGERT---VAVPPVHHLTK-RSFFDIQCKGVYDKSIFARLDRICEDCYNLFREPQLHSL
Ignelater     ---SSPTQRS---RPLL-SHHLTK-RAFADQQCKGVYDKSIYARLDRICEDCYNLFREPELHTL
Photinus      ----TPTQRS---RPLL-SHHLTK-RAFADQQCKGVYDKSIYARLDRICEDCYNLFREPELHSL
Aquatica      ---STPTQRS---RPLL-SHHLTK-RAFADQQCKGVYDKSIYARLDRICEDCYNLFREPEIHGL
Nicrophorus   ---GSPAQRM---SPLL-SHHLSKRGGFLDLQCKGVYESSIFAKLDRICEDCYNLFREPKLQGL
Aleochara     --DCSPAER----KPLL-THHLTK-RSFYDIQCKGVYDKSIFARLDRICEDCYNLFRDPHLHSL
Oryctes       ---SSPLQRT---HPLL-THHLMKRKIFFQLECKGQYNKSRFARLDRICDDCYHLFREPEVDRM
Coccinella    ---GSPANRS---SNLL-SHHLAK-RSFFDIQCKGVYDKSIFARLDRICEDCYNLFREPQVHSL
Harmonia      ---GNPTNRS---SGLL-SHHLAK-RSFFDIQCKGVYDKSIFARLDRICEDCYNLFREPQVHSL
Dendroctonus  ---GSPWRRGS--GPLL-THHLSK-RSFFDIQCKGVYDKTIFAKLDRICEDCYNLFREPQVHSL
Hypothenemus  ---GSPWRRGSNNGPLL-THHLSK-RSFADIQCKGVYNKQIFARLDRICEDCYNLFREPHILSL
Anoplophora   ---GSPANRS----PLL-THHLTK-RSFFDIQCKGVYDKSIFARLDRICEDCYNLFREPSIHSL
Leptinotarsa  ---GSPANRS---PELLSRHHLAK-RSFFDLQCRGVYDKSIFARLDRICVDCYNLFREPSVHSL
Aethina       ---GSPS-RS----PLL-THHLTK-RSFHAIECQGVYDKSIFARLDRICEDCYSLFREPEVHSL
HycleusITP    ---SPLMDRS----PSLLAHHLTK-RSFFDIQCKGVYDKSIFARLDRICEDCYNLFREPQLHSL
Tenebrio      ----IPTNGS----PVLLPHHFTK-RSFFDIQCKGVYDKSIFAKLDSICEDCYNLFREPQLHSL
Tribolium     ----SPAGRS----PAFLPHHFTK-RSFFDIQCKGVYDKSIFAKLDSICEDCYMLFREPQLHNL
Tribolium     ----SPAGRS----PAFLPHHFTK-RSFFDIQCKGVYDKSIFAKLDSICEDCYMLFREPQLHNL

            129
Pogonus       CRKNCFTTDYFKGCLEALQLTDEMEQIQTWIKQLHGAEPGV---------------
Ignelater     CKKSCFTTDYFKACLESLQLQDEMEQIQTWIKQLHGAEPGPV--------------
Photinus      CKKNCFTSEFFKACLDSLQLQDEMEQIQIWIKQLHGAEPGV---------------
Aquatica      CKEECFTNDYFKACLDTLQLQDEIEQIQIWIKQLHGAEPGV---------------
Nicrophorus   CKNNCFTTNIFKGCLESLQLNDEMEQVQTWIKQLHGAEPGF---------------
Aleochara     CRKNCFTTDYFKGCLEALQLTDEMEQVQTWIKQLHGAEPAV---------------
Oryctes       CRKNCFTTAIFKGCLEALQLGDELDEIQASIKELHGAEARV---------------
Coccinella    CRRNCFTTDYFKGCLDTLQLSDELQQIQTWIKQLHGADPGV---------------
Harmonia      CRKNCFTTDYFKGCLDTLQLSDELQQIQTWIKQLHGADPGV---------------
Dendroctonus  CMKNCFTTAYFKGCLETLQLTDEMEQVKDWIKQIHGAPPDE---------------
Hypothenemus  CMSNCFTTSYFNGCVESLLKLDEMPTFRKMIEYLAK--------------------
Anoplophora   CRQDCFTTKFFKGCIDTLQLSDEMEQIKSWIKQLHGADPGV---------------
Leptinotarsa  CRDNCFTSDYFKGCVDTLQLSDHIPEIMDMIKQLKGAKA-----------------
Aethina       CRKDCFTTSYFKGCLDTLQLQDEMEQVQTWIKQLHGADPKL---------------
HycleusITP    CRKDCFTTDYFKGCLDTLQLSDEEQQIQTWIKQLHGADPGV---------------
Tenebrio      CRKNCFTTDYFKGCLETLQLSDEEAQIQLWIKQIRGAELGGLGPSVSPPNTS----
Tribolium     CRKNCFTTDYFKGCIDTLQRSDEEAQIQLWIKQIRGAELGGLGTLIAPFLFSNTLT
Tribolium     CRKNCFTTDYFKGCIDTLQRSDEEAQIQLWIKQIRGAELGGLGPSASPPNTS----
```

Figure S43. Alignment of ITP-A precursors.

```
Pogonus       MPQTQSSSKSAINSTSCVSTRTTRRLRTGNVRCAAVWLAAACALALGGNGGSGTGGSRLAIPSG
Ignelater     ----------------------MHHRSSTCVSTRGVWVCMALALVLQLAQS-------------
Photinus      ----------------------MHRRSSTSIYTKGVWVCMALTLVIQLAHC-------------
Aquatica      ----------------------MHRRSSTSLNTKGVWVCMALTLVFQLTQS-------------
Nicrophorus   -----------------------MHHRSASISTQRVWAFMALALVLQMVSG-------------
Aleochara     -----------------------MHQRSTSIGTQGVWAYMAVALMLQMTAC-------------
Oryctes       -----------------------MNHRSTSVSTQFVWVCMALTLVVQIALS-------------
Coccinella    -----------------------MNYLSSKVSTTLLWVCMALVLFLSGSFG-------------
Harmonia      -----------------------MNYLSSKVSTTLLWVCMAFIFFFGSSLG-------------
Dendroctonus  -----------------MSQRSSQLEQRGRGTRRAAVWCLAIALVVQSARAG------------
Hypothenemus  -----------------MSQRSSQLEQRGRGTRRAAIWCLAIALVVQSARAG------------
Anoplophora   ---------------------MSHRSLKSVTNTQAVWVCMALALIFHITSG-------------
Leptinotarsa  ---------------------MSNRSSSSVRGTQVAWWCMAIALILHVSSG-------------
Aethina       ---------------------MSHPLSQSVRSTQIVGVCMALALVVQVVVG-------------
Hycleus       -------------MFQIPEKHMNCQS-PKSISRQAIWSCMALAVLLIQVTT-------------
Tenebrio      ---------------------MSYRS-SIIVNTQAVWVCMTLAVIIQAVTS-------------
Tribolium     ---------------------MNYRS-SKSISTQAVWVCMVLAVVFQEITS-------------

              65
Pogonus       ATASPAGERT---VAVPPVHHLTKRS-FFDIQCKGVYDKSIFARLDRICEDCYNLFREPQLHSL
Ignelater     ----SPTQRS---RPLL-SHHLTKRA-FADQQCKGVYDKSIYARLDRICEDCYNLFREPELHTL
Photinus      ----TPTQRS---RPLL-SHHLTKRA-FADQQCKGVYDKSIYARLDRICEDCYNLFREPELHSL
Aquatica      ----TPTQRS---RPLL-SHHLTKRA-FADQQCKGVYDKSIYARLDRICEDCYNLFREPEIHGL
Nicrophorus   ----SPAQRM---SPLL-SHHLSKRGGFLDLQCKGVYESSIFAKLDRICEDCYNLFREPKLQGL
Aleochara     --DCSPAERK----PLL-THHLTKRS-FYDIQCKGVYDKSIFARLDRICEDCYNLFRDPHLHSL
Oryctes       ----SPLQRT---HPLL-THHLMKRKIFFQLECKGQYNKSRFARLDRICDDCYHLFREPEVDRM
Coccinella    ----SPANRS---SNLL-SHHLAKRS-FFDIQCKGVYDKSIFARLDRICEDCYNLFREPQVHSL
Harmonia      ----NPTNRS---SGLL-SHHLAKRS-FFDIQCKGVYDKSIFARLDRICEDCYNLFREPQVHSL
Dendroctonus  ----SPWRRGS--GPLL-THHLSKRS-FFDIQCKGVYDKTIFAKLDRICEDCYNLFREPQVHSL
Hypothenemus  ----SPWRRGSNNGPLL-THHLSKRS-FADIQCKGVYNKQIFARLDRICEDCYNLFREPHILSL
Anoplophora   ----SPANRS---PLL--THHLTKRS-FFDIQCKGVYDKSIFARLDRICEDCYNLFREPSIHSL
Leptinotarsa  ----SPANRS---PELLSRHHLAKRS-FFDLQCRGVYDKSIFARLDRICVDCYNLFREPSVHSL
Aethina       ----SP-SRS---PLL--THHLTKRS-FHAIECQGVYDKSIFARLDRICEDCYSLFREPEVHSL
Hycleus       --ASPLMDRS---PSLL-AHHLTKRS-FFDIQCKGVYDKSIFARLDRICEDCYNLFREPQLHSL
Tenebrio      ----IPTNGS---PVLL-PHHFTKRS-FFDIQCKGVYDKSIFAKLDSICEDCYNLFREPQLHSL
Tribolium     ----SPAGRS---PAFL-PHHFTKRS-FFDIQCKGVYDKSIFAKLDSICEDCYMLFREPQLHNL

             129
Pogonus       CRSQCFSTQYFKGCLESLLLT--EEEQKYHQMIEYLGKRSN--
Ignelater     CKKNCFKTDYFVACLEVLELE--EEREEIVKSVKQLNHRRSRH
Photinus      CKKNCFKSPYFQTCIDTLNLG-SEAAREVYEWVKRMNKRRL--
Aquatica      CKKNCFKTPNFQACIDAFNLG-DDEKKNFYSLVKQVNRRRL--
Nicrophorus   CKSDCFSSDYFKGCLEALLLN--EEMDKFAKMVEYIGKK----
Aleochara     CRSECFSKSYFRGCVEALLLS--EEWEHLNRSVHYLGRK----
Oryctes       CRLNCFKSAYFKGCLKALLIDSVDEIKSIEEDIEITGK-----
Coccinella    CRNRCFSTRIFKGCVDSLLLS--EELPKFSKMIEYLGK-----
Harmonia      CRNRCFSTRIFKGCVDSLLLS--EELPKFSKMIEYLGK-----
Dendroctonus  CMSKCFTTEYFTGCVESLLKS--DEMPSYRKMIEYLAK-----
Hypothenemus  CMKDCFTTEYFKGCLETLQLT--DEMEQVKEWIKHIHGASPDK
Anoplophora   CRSECFSTKYFKGCVESLLMV--EDWPKFEKMIDYLAK-----
Leptinotarsa  CRSQCFSTKIFKGCVESLLEM--EEWSKLKKMIDFVGK-----
Aethina       CRNKCFSTKYFQGCVDSLLLT--DEMPEFNRTIEYLSGY----
Hycleus       CRSQCFTTKYFKGCVESLLLN--EEMPKFLKMIEYLAK-----
Tenebrio      CRSQCFSTKYFVGCVESLLLS--EEMPNFRKMIEYLSK-----
Tribolium     CRSECFSTKYFVGCVESLLLS--EEMPKFRKMIEYLSK-----
```

Figure S44. Alignment of ITP-B precursors.
